# Electrochemical Single-Carbon Insertion via Distonic Radical Cation Intermediates

**DOI:** 10.1021/jacs.5c06798

**Published:** 2025-07-14

**Authors:** Tatsuya Morimoto, Yoshio Nishimoto, Taku Suzuki-Osborne, Su-Gi Chong, Kazuhiro Okamoto, Tomoki Yoneda, Azusa Kikuchi, Daisuke Yokogawa, Mahito Atobe, Naoki Shida

**Affiliations:** † Department of Chemistry and Life Science, 13154 Yokohama National University, 79-5 Tokiwadai, Hodogaya-ku, Yokohama 240-8501, Japan; ‡ Department of Chemistry, Graduate School of Science, 12918 Kyoto University, Kitashirakawa-Oiwake-cho, Sakyo-ku, Kyoto 606-8502, Japan; § Department of Chemistry, 1555 University of Bath, Claverton Down, Bath BA2 7AY, U.K.; ∥ Institute of Advanced Sciences, Yokohama National University, 79-5 Tokiwadai, Hodogaya-ku, Yokohama 240-8501, Japan; ⊥ Department of Science, 34823 University of Toyama, 3190 Gofuku, Toyama 930-0887, Japan; # Department of Pharmaceutical Sciences at Narita, 446160 International University of Health and Welfare, 4-3 Kozunomori, Narita City, Chiba 286-8686, Japan; ∇ Department of Multidisciplinary Science, Graduate School of Arts and Sciences, 13143 The University of Tokyo, Komaba, Meguro-ku, Tokyo 153-8902, Japan; ○ PRESTO, Japan Science and Technology Agency (JST), 4-1-8 Honcho, Kawaguchi, Saitama 332-0012, Japan

## Abstract

The synthesis of polysubstituted (hetero)­aromatic compounds is essential in various fields, including pharmaceuticals, where such compounds are fundamental to many approved drugs. In this study, we present a novel electrochemical method for single-carbon insertion targeting various (hetero)­aromatic compounds, with a particular focus on pyridines. In this process, the electrochemical oxidation of pyrrole derivatives produces a radical cation intermediate, which then undergoes nucleophilic attack by diazo compounds to yield polysubstituted pyridine derivatives. Notably, the insertion position is influenced by the electronic properties of *N*-protecting groups, allowing for unprecedented *para*-selective insertion through the introduction of electron-withdrawing groups. Insights from *in situ* spectroscopy and theoretical calculations suggest the involvement of distonic radical cation intermediates, facilitating carbon-atom migration on the aromatic ring and enabling insertion at different positions. This study expands the chemical toolkit for synthesizing polysubstituted (hetero)­aromatic compounds and introduces a new concept for single-carbon insertion chemistry.

## Graphical abstract

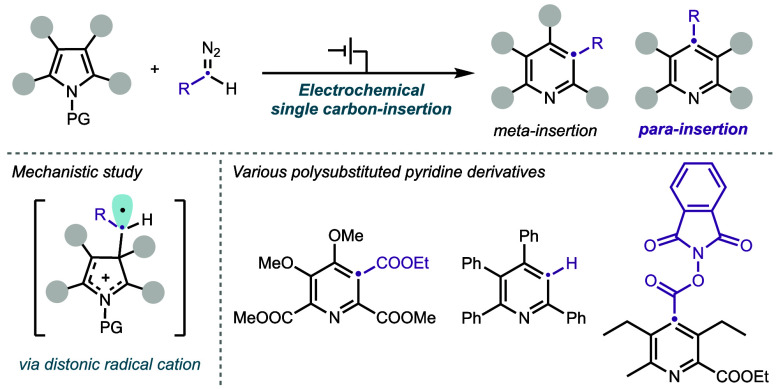

## Introduction

Polysubstituted (hetero)­aromatic compounds play a crucial role in diverse fields, particularly in pharmaceuticals.^1–8^ Approved drugs such as Netupitant,^1^ Esomeprazole,^4^ Pyridoxine,^8^ and Opicapone^7^ contain benzene and pyridine rings with more than three substituents (Figure 1A). To synthesize these compounds, researchers have developed multiple methods, including coupling reactions,^9–11^ C–H functionalization,^12–14^ and cyclization reactions.^15^

**Figure 1:**
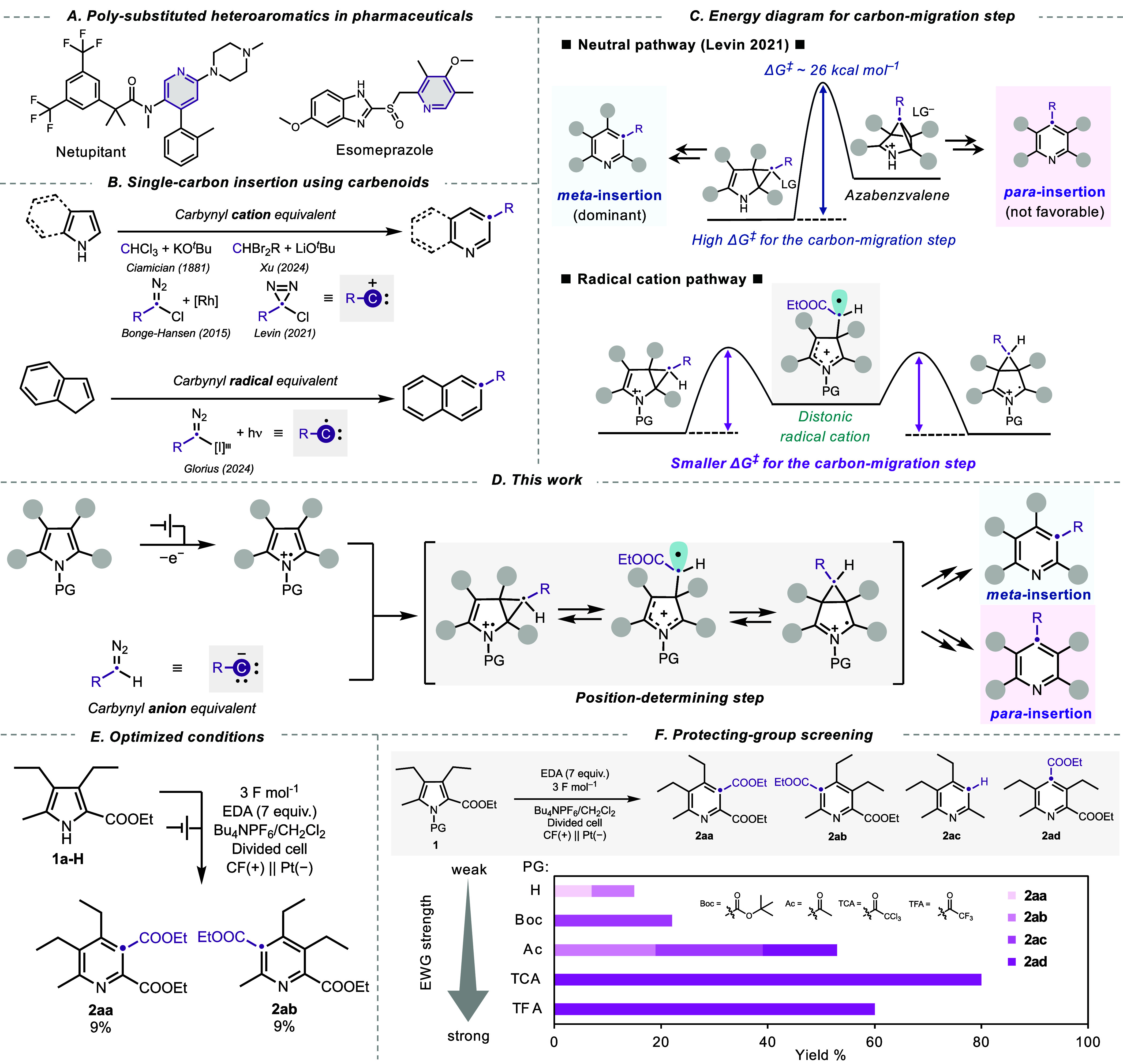
Overview and conceptual framework of the present study. (A) Representative examples of poly substituted (hetero)­aromatics found in pharmaceuticals. (B) Previously reported types of carbene equivalents.^18,20–23^ (C) Hypothetical energy profile for single-carbon insertion into pyrrole via neutral- and radical cation pathways. (D) Electrochemical single-carbon insertion reaction proposed in this study. (E) Electrolysis of **1a-H** under optimized conditions. (F) Evaluation of various *N*-Protection groups (PGs).

Single-carbon insertion offers an alternative approach for modifying polysubstituted (hetero)­aromatic compounds, gaining prominence in the realm of skeletal editing within synthetic organic chemistry. This method significantly alters the structure of the parent skeletons.^16–19^ A common technique for single-carbon insertion involves the reaction of halogenated carbene-equivalents, specifically carbynyl cations, with cyclic compounds (Figure 1B, top). A classic example of this approach is the Ciamician–Dennstedt rearrangement, which transforms pyrrole into pyridine using chloroform as a carbon source and KO*^t^* Bu as a base.^20^ Recently, Xu et al. introduced a similar reaction using 1,1-dibromoalkane derivatives and LiO*^t^* Bu to incorporate diverse functional groups.^18^ Alternatively, carbenoids stabilized by N_2_-groups have also been employed as a carbon source in this reaction manifold. Bonge–Hansen and co-workers reported the transformation of indoles to quinolines using α-halo-ethyl diazoacetate (α-halo-EDA) in the presence of a Rh catalyst.^21^ Levin developed a robust ring expansion strategy for pyrrole- and indole-derivatives using α-chlorodiazirines as a carbon source, demonstrating an excellent substrate scope.^22^ While this reaction is limited to benzylic carbon insertion, the use of α-chlorodiazirines allows for carbon insertion without necessitating potent bases or transition metal catalysts, proving highly advantageous for late-stage functionalization.^22^ As a mechanistically distinct reaction, Glorius recently reported a ring expansion reaction of indenes under light irradiation using a hypervalent iodine reagent as a carbon source, with a proposed carbynyl radical as the reaction intermediate (see Figure 1B, bottom).^23^

Despite the rapid growth of the single-carbon insertion methodology for synthesizing (hetero)­aromatic compounds, controlling the insertion position remains a significant challenge. For instance, ring expansion of pyrrole to pyridine is solely reported for *meta*-insertion, where a carbon atom is inserted into the *meta*-position of a nitrogen atom.^18–22^ Levin et al. observed rare *para*-insertion as a minor product in the reaction of α-chlorodiazirines and a pyrrole derivative bearing bulky substituents at α-positions.^22^ Their density functional theory (DFT) simulation revealed that *para*-insertion is less favored than *meta*-insertion due to the high energy barrier (Δ*G* ^‡^ > 26 kcal mol^–1^) from transannular cyclopropane to azabenzvalene, a crucial intermediate for the carbon-atom migration step (Figure 1C, top); thus, they reported that the direct 3,4-cyclopropanation via the zwitterion could cause *para*-insertion. In other words, the high energy required for the carbon-atom migration step limits the scope of insertion positions. We hypothesized that carbon migration might proceed with lower activation energy via a radical cation intermediate owing to destabilization and bond-weakening resulting from the removal of an electron from the bonding orbital.^24^ Consequently, the C–C bond of the cyclopropane ring cleaves to generate a distonic radical cation as an intermediate instead of azabenzvalene under ambient conditions, thereby introducing the potential for unprecedented selectivity in carbon-insertion reactions (Figure 1C, bottom).

In this study, we present a novel single-carbon insertion approach based on a radical cation intermediate generated via electrochemical oxidation (Figure 1D). We employ α-H diazo ester as the carbon source, which formally acts as a carbynyl anion equivalent following denitrogenation and deprotonation. While the nucleophilic reactivity of α-H diazo ester, such as ethyl diazoacetate (EDA), toward radical cation species, is documented,^25,26^ the oxidative single-carbon insertion and ring expansion with α-H diazo ester have not been previously reported. Although electrochemical ring-contraction and ring-expansion reactions involving nitrogen insertion have been demonstrated,^27–29^ electrochemical ring-expansion reactions via single-carbon insertion have been reported only once, and only for a single specific compound. This example, dating back to 1972, involved a reaction of 2,3,4,5-tetrakis­(4-methoxyphenyl)­pyrrole, where nitromethane solvent served as the carbon source to afford *meta*-insertion product.^30^

## Results and Discussion

### Preliminary Reaction and Protection-Group Screening

First, we conducted the electrolysis using EDA and ethyl 3,4-diethyl-5-methyl-pyrrole-2-carboxylate (**1a-H**). The optimization details are summarized in the Supporting Information (Tables S1 and S2). Importantly, the reaction proceeded solely in the weakly coordinating electrolyte, suggesting that the formation of the highly electrophilic and noncoordinated radical cation species is crucial for the desired reaction. Under the optimized conditions, the isomeric *meta*-insertion products, **2aa** and **2ab**, were obtained with a total yield of 18% (Figure 1E).

We then conducted screening of *N*-protecting groups (PG, PG = Boc, Ac, TCA, and TFA) (Figure 1F). Depending on the PG type, Three structural isomers, **2aa, 2ab**, **2ad**, and the decarbonylated product, **2ac**, were obtained. Overall, the total yield of insertion products increased with stronger electron-withdrawing properties of the PG. Selective formation of **2ac** was achieved by introducing the Boc-group. Interestingly, **2ac** was the exclusive decarboxylation product, and no other structural isomer was observed. Introducing the Ac-group resulted in a mixture of **2aa**, **2ab**, and **2ac**. Notably, using TCA as the PG led to an 80% yield with the selective production of the *para*-insertion product, **2ad**. Similarly, TFA yielded only **2ad** as the sole product, indicating a preference for electron-withdrawing PG for *para*-insertion.

### Selectivity Study

Next, the selectivity of the insertion position was investigated across a broad range of substituted pyrroles (Figure 2A). For **1a-TFA**, it is noteworthy that the reaction proceeded smoothly even at a scale of up to 1.15 mmol. In addition, we successfully obtained *para*-insertion products in moderate to high yields from seven pyrroles containing esters (**1a**–**e**), ketones (**1f**–**g**), or imines (**1h**) at the α-position bearing TFA as *N*-protecting group. Although the substrate containing an aldehyde was not directly compatible with the reaction conditions (see Supporting Information), the ring expansion of the imine-substituted pyrrole with −BF_2_– protecting group (**1h**) yielded the corresponding pyridine (**2h**), which can be hydrolyzed to afford the α-formyl product.

**Figure 2:**
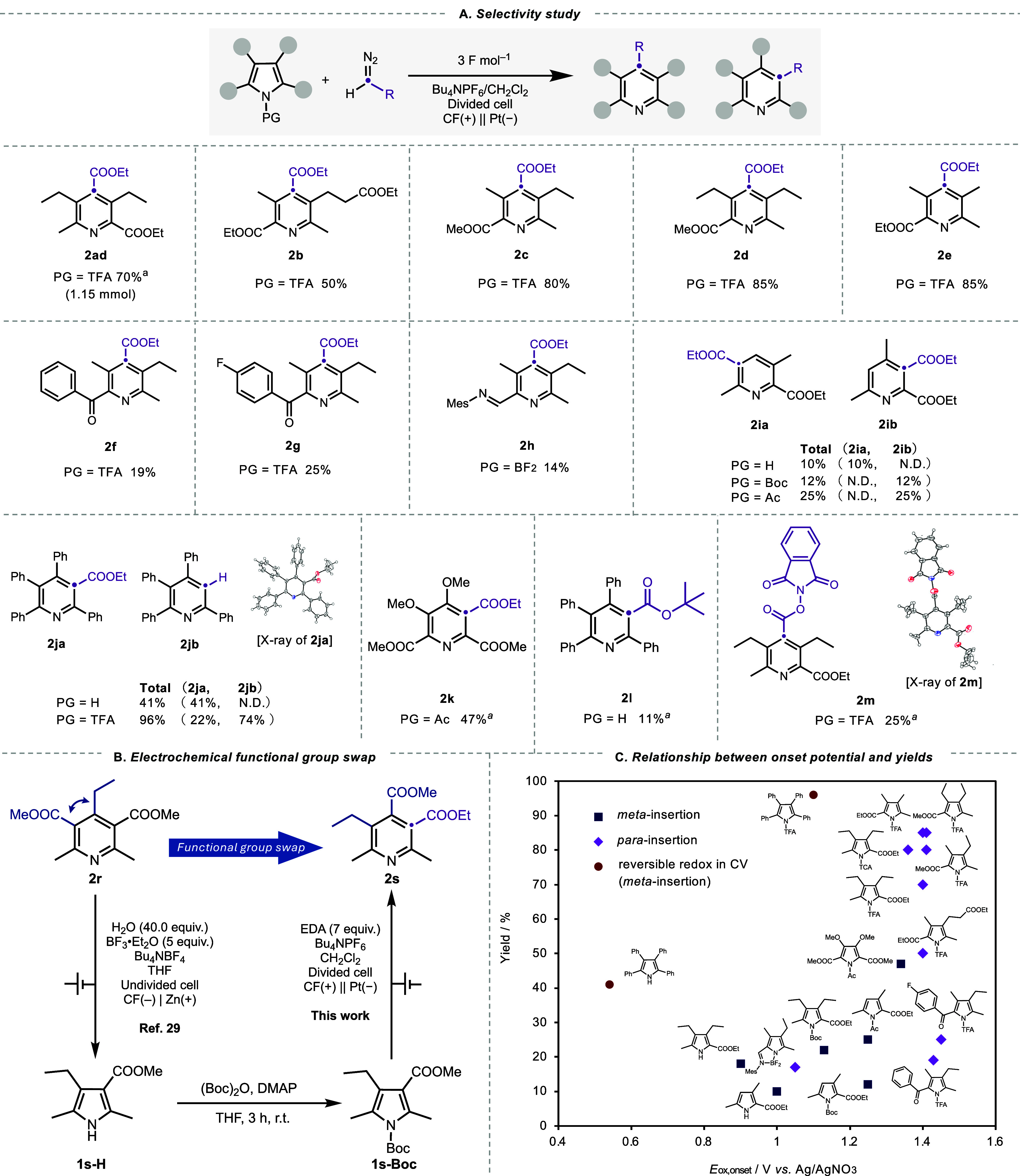
(A) Study on the insertion position with various pyrrole-derivatives. (B) Electrochemical functional group swap.^29^ (C) Correlation between oxidation onset potential (*E*_ox,onset_) and product yields. Yields were determined by ^1^H NMR using benzaldehyde as an internal standard. *^a^* Isolated yield.

The reaction was also applicable to trisubstituted pyrroles (**1i-H**, **1i-Ac**, and **1i-Boc**), yielding the corresponding pyridines (**2ia, 2ib**). Intriguingly, while the reaction of **1i-H** afforded *meta*-insertion product at less hindered site (**2ia**), **1i-Ac** and **1i-Boc** enabled insertion at sterically more demanding positions to produce **2ib**. This result again highlights the critical influence of electronic modulation by the *N*-protecting groups on insertion site-selectivity. 2,3,4,5-tetraphenylpyrrole (**1j-H** and **1j-TFA**) afforded carbon insertion products in moderate to high yields. In particular, **1j-TFA** selectively afforded *meta*-insertion products (**2ja** and **2jb**) in a total yield of 96%. Such high selectivity for *meta*-insertion, despite the use of TFA as protecting group, was attributed to the low oxidation potential of **1j-TFA** compared to other TFA-protected pyrrole-derivatives (vide infra). Regarding the compatibility of diazo ester, *tert*-butyl α-diazoacetate was also applicable to this reaction, and its reaction with **1j-H** yielded *meta*-insertion product (**2l**). Redox-active diazo esters were also compatible with this reaction. The reaction of **1a-TCA** with 1,3-dioxoisoindolin-2-yl 2-diazoacetate yielded *para*-insertion product (**2m**), which could be useful for subsequent reactions such as photocatalytic borylation.^31^

An additional selectivity study was conducted on the pyrrole obtained by the electrochemical ring-contraction reported by Cheng et al. (Figure 2B).^29^ Using tetra-substituted pyridine (**2r**) derived from the Hantzsch ester as a substrate, we performed a sequential transformation involving ring contraction via cathodic reduction, Boc protection, and ring expansion via anodic oxidation. This process afforded compound **2s** in 27% yield, featuring a successful electrochemical functional group swap of the alkyl ester at the 3-position and the ethyl group at the 4-position.

The limitations of the ring expansion of pyrrole are presented in Figure S40. In particular, electrochemical single-carbon insertions were unsuccessful for substrates with no substituents at 2- or 5-position, likely due to the high spin density at these positions, which decreases the stability of the corresponding radical cation intermediate. Examples of electrochemical single-carbon insertions into other five-membered rings (**1n**–**p**, **3a**–**d**) are also summarized in the Supporting Information.

To gain deeper insights, we analyzed the correlation between the oxidation potential and the yield. Figure 2B illustrates the relationship between yields and oxidation onset potential (*E*_ox,onset_) determined by cyclic voltammetry (CV) measurements (see Figures S5–S32 in Supporting Information). The results indicate that starting materials with higher *E*_ox_ values tend to yield the insertion product in higher yields. This outcome is attributed to the increased electrophilicity of the resulting radical cation species owing to the higher *E*_ox,onset_, thereby enhancing its reactivity with EDA. This finding aligns with our initial observation that a weakly coordinating electrolyte is favorable for this reaction. Substrates such as **1j-H** and **1j-TFA** exhibited reversible redox behavior in the CV measurements, correlating with exceptionally high yields (Figure 2C, red dots). These results suggest that radical cations with either higher electrophilicity or longer lifetimes readily react with EDA, leading to higher yields.

Importantly, the positional outcome of the carbon insertion was also found to be dependent on *E*_ox,onset_. Specifically, *meta*-insertion products—including both ester-substituted and decarbonylated species—were typically observed at lower *E*_ox,onset_ values (Figure 2C, blue squares), whereas *para*-insertion products were preferentially formed at higher *E*_ox,onset_ values (Figure 2C, purple diamonds). Notably, *para*-selectivity emerged when the onset potential exceeded approximately 1.4 V *versus* Ag/AgNO_3_. This trend suggests that electron-withdrawing *N*-protecting groups fine-tune the electronic structure of the pyrrole ring, enabling *para*-selective insertion. An exception to this trend is substrate **1h-TFA**, which exhibits *para*-selectivity despite having a relatively low *E*_ox,onset_ values. We attribute this anomaly to the unique electronic and conformational influence of BF_2_-chelated imine-type protecting group, which distinguishes it from typical carbamate-based protecting groups.

### Mechanistic Study

Next, mechanistic studies were conducted. Initially, CV measurements were performed on **1a-H** and EDA. The results revealed that the onset potential of **1a-H** was 0.9 V, while that of EDA was 1.6 V (Figure 3A). Furthermore, the onset potentials of the other substrates utilized in this study were all below 1.6 V (Figure 2B and Supporting Information Section S4.2), suggesting that the reaction progresses through the oxidation of substrates such as pyrroles and cyclopentadienes.

**Figure 3:**
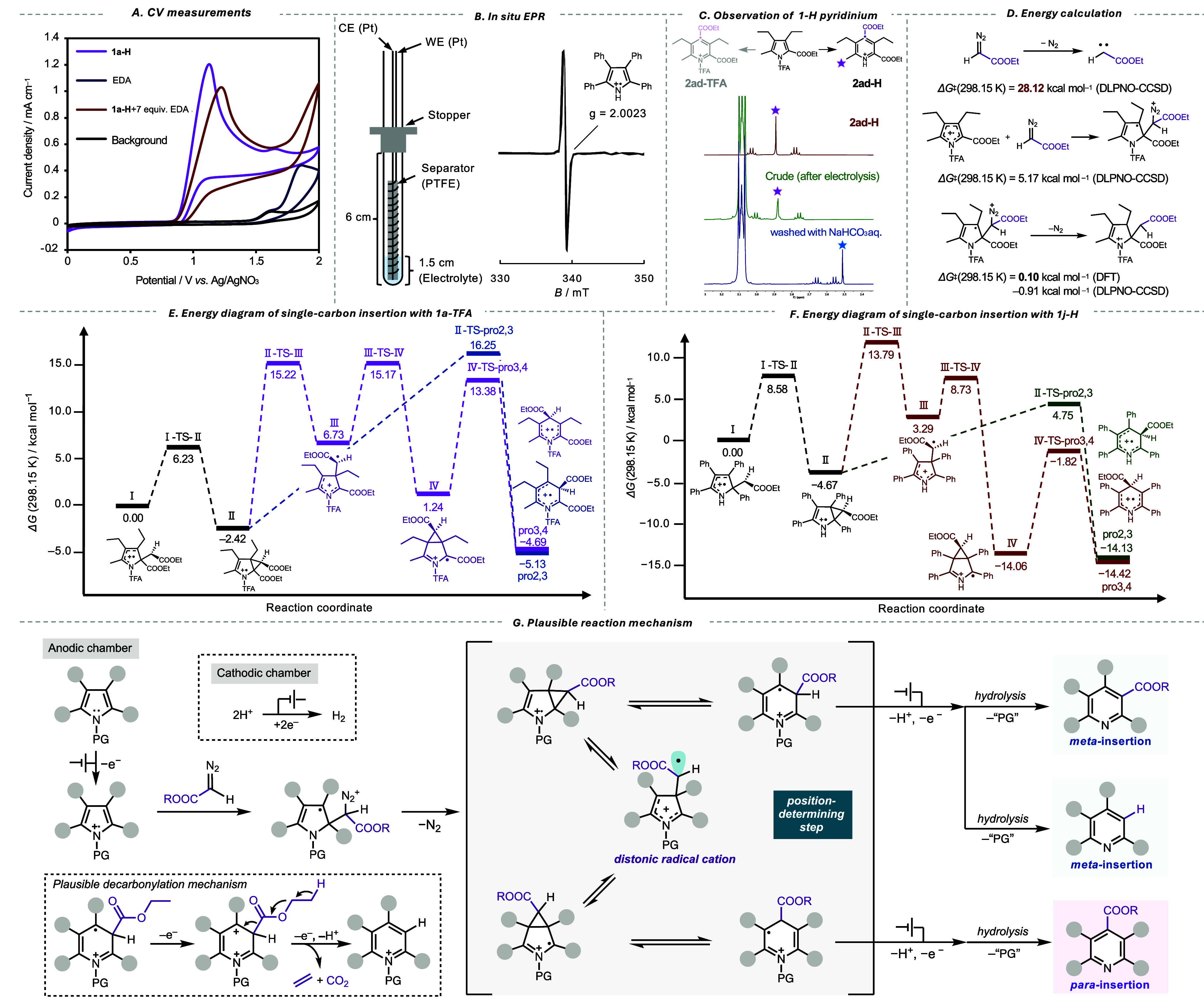
Mechanistic study. (A) Cyclic voltammograms (CVs) of **1a-H** and EDA. (B) *In situ* electron paramagnetic resonance (EPR) spectra for detecting radical cation species. (C) Product analysis using (*N*-H pyridinium species) **1a-TFA**. (D) Energy diagram for the N_2_-release step. (E) Energy diagram for the carbon migration step of **1a-TFA**. (F) Energy diagram for the carbon migration step of **1j-H**. (G) Plausible reaction mechanism.

To study the radical cations produced through anodic oxidation, we conducted *in situ* EPR measurements with custom electrochemical cells (Figure 3B). The EPR measurements were conducted during electrolysis with **1d-H** as the substrate, leading to the successful detection of the radical cation signal. This finding confirms the involvement of the radical cation as an intermediate in the reaction.

We then examined the structure of the postreaction product to investigate the mechanism of protecting group removal. Following the reaction using **1a-TFA** as a starting material, ^1^H NMR analysis of the crude product was conducted (Figure 3C). A singlet peak corresponding to the methyl group was detected, with a chemical shift consistent with *N*-H pyridinium (**2ad-H**), rather than *N*-TFA-pyridinium (**2ad-TFA**). Washing the crude mixture with saturated NaHCO_3_ *aq.* led to a peak shift toward a higher magnetic field, consistent with the chemical shift of the insertion product, **2ad** (see Supporting Information Section S3.6). Oxidative insertion of EDA into **1a-TFA** is anticipated to produce **2ad** with TFA protection (**2ad-TFA**) in the form of pyridinium salt. However, the presence of such an intermediate was not definitively confirmed, possibly due to its unstable nature, leading to hydrolysis into pyridine, followed by protonation via EGA.

Further mechanistic study was conducted through theoretical calculations based on density functional theory (DFT) and the coupled-cluster method (Figure 3D–F). First, the denitrogenation step was simulated. The computation indicated that 28.12 kcal mol^–1^ is required to directly form a carbene from EDA, suggesting that N_2_ release from EDA barely occurs under ambient conditions (Figure 3D). In contrast, the nucleophilic addition of EDA to the radical cation requires only 5 kcal mol^–1^ of energy, while the release of N_2_ from the EDA-bound radical cation species requires minimal energy (Figure 3D, −0.91 kcal/mol at DLPNO–CCSD and 0.10 kcal/mol at DFT). Therefore, it can be reasonably inferred that EDA reacts with radical cations species as a nucleophile, leading to subsequent N_2_ release. The calculations also revealed that EDA addition at the 2-position of the pyrrole ring is energetically favorable (Supporting Information Section S4.4.1).

Figure 3E shows the energy diagram for the carbon-migration step of **1a-TFA** at the DLPNO–CCSD/def2-TZVPP­(D)//CAM-B3LYP-D3­(BJ)/6–31­(+)­G­(d,p) level of theory (see Supporting Information Section S4.4 for details). The thermal stability of the precursor for the *meta*-insertion product, **pro-2,3**, exceeded that of the *para*-insertion precursor, **pro-3,4**. Despite the higher stability of **pro-2,3**, the experimental observation of selectively obtaining the *para*-insertion product implies that the selectivity for the insertion position is under kinetic control rather than thermodynamic control. The oxidation of **pro-2,3** and **pro-3,4** is expected to occur at a lower energy level than that of the initial material, a pyrrole derivative, owing to the unstable nature of the radical cation intermediate. This instability leads to rearomatization, forming pyridine and promoting the second oxidation step. The rapid progression of the second oxidation step indicates kinetic control of the reaction. Further analysis revealed that a Δ*G* ^‡^ of 18.67 kcal mol^–1^ is necessary for the ring-opening of the cyclopropane formed at the 2,3-position to complete a *meta*-insertion (**II-TS-pro2,3**). In contrast, the formation of **II** from **IV** via a distonic radical cation (see SI, Figure S37), which leads to the *para*-insertion intermediate, requires a lower activation energy of Δ*G* ^‡^ = 17.64 kcal mol^–1^. Based on the experimental result, which shows selective formation of the *para*-insertion product, along with theoretical calculation, it is suggested that the selectivity for the insertion position is governed by kinetic control rather than thermodynamic control.

Figure 3F shows an energy diagram of **1j-H**, displaying a *meta*-insertion product with a relatively high yield. The thermal stability of the *para*-insertion product precursor exceeded that of *meta*-insertion, indicating kinetic control of the reaction. The maximum Δ*G* ^‡^ from **II** to **pro2,3** of the *meta*-insertion product was calculated to be 9.42 kcal mol^–1^. In contrast, the maximum Δ*G* ^‡^ for *para*-insertion to yield **pro3,4** was calculated to be 18.46 kcal mol^–1^. This observation indicates that the formation of *meta*-insertion products is significantly faster than that of *para*-insertion products. Again, the reaction is kinetically controlled. Similarly, the energy diagram of **1a-H** (the unprotected form of **1a-TFA**) demonstrates a faster formation of the *meta*-insertion product compared to the *para*-insertion product (refer to Figure S36 for details). A comparison between the two energy diagrams (Figure 3E,F) revealed that the Δ*G* ^‡^ for the process of **pro2,3** from **II** is 9.42 kcal mol^–1^ for **1d-H**, while that for **1a-TFA** is 18.67 kcal mol^–1^. These differences suggest that the cyclopropane ring formed at the 2,3-positions is less likely to cleave with **1a-TFA** than with **2a-H**, possibly due to differences in electron density affecting the bond cleavage ease of the cyclopropane ring at the 2,3-positions.

Based on these findings, we propose the reaction mechanism (Figure 3G). First, radical cations of the substrates are formed through single-electron oxidation at the anode. Subsequently, nucleophilic attack of diazo esters, followed by N_2_ elimination, yields cyclopropane-containing intermediate species. The reversible transformation of these species occurs through the generation of distonic radical cations, facilitating carbon-atom migration under ambient conditions. Depending on the electronic and steric properties of the substituents, particularly the *N*-protecting group, the insertion occurs at either the *meta*- or *para*-position. For certain substrates, the reaction proceeds with a concomitant decarbonylation event at the *meta*-position. Although the decarboxylation mechanism is still unclear, a plausible decarboxylation pathway is shown in Figure 3G. The insertion position is determined by kinetic control, where Δ*G* ^‡^ depends on the electronic structure of the aromatic ring controlled by PG. Subsequent one-electron oxidation, deprotonation, and hydrolysis produce pyridines as the final product.

## Conclusions

In conclusion, we have demonstrated the electrochemical ring expansion reaction using α-H diazo esters as a carbynyl anion equivalent. This approach enabled efficient single-carbon insertion into a range of polysubstituted pyrroles, affording structurally diverse pyridine derivatives. Importantly, the insertion position was controlled through electronic perturbation by the *N*-protecting group (PG), and unprecedented *para*-selective insertion was achieved by introducing an electron-withdrawing protecting group to the pyrrole derivatives. *In situ* spectroscopy and theoretical calculations supported the reaction mechanism involving a distonic radical cation intermediate. This study introduces a novel concept for single-carbon insertion chemistry, expanding the chemical toolbox for synthesizing polysubstituted (hetero)­aromatic compounds.

## Supplementary Material



## Notes

Description of the materials and experimental procedure, voltammetry, details of computation, crystallographic information, and NMR spectra (PDF)

## Abbreviations

Boc: *tert*-butoxycarbonyl
Ac: acetyl
TCA: trichloroacetyl
TFA: trifluoroacetyl
NMR: nuclear magnetic resonance
EPR: electron paramagnetic resonance
NHPI: *N*-hydroxyphthalimide.
