# Supplementary material for: Electrochemical Single-Carbon Insertion via Distonic Radical Cation Intermediates
Source: J Am Chem Soc. 2025 Jul 14;147(29):25635–41. doi: 10.1021/jacs.5c06798 (PMC12291465; doi:10.1021/jacs.5c06798)
Supplement: Supplementary file 1 [file ja5c06798_si_001.pdf]

Supporting Information

Electrochemical Single-Carbon Insertion via Distonic Radical Cation Intermediates

Tatsuya Morimoto,[1] Yoshio Nishimoto,[2] Taku Suzuki-Osborne,[3] Su-Gi Chong,[4] Kazuhiro Okamoto,[5] Tomoki Yoneda,[6] Azusa Kikuchi,[1] Daisuke Yokogawa,[7] Mahito Atobe,[1,4*] Naoki Shida[1,4,8*]

[1] Department of Chemistry and Life Science, Yokohama National University, 79-5 Tokiwadai, Hodogaya-ku, Yokohama 240-8501, Japan

[2] Department of Chemistry, Graduate School of Science, Kyoto University, Kitashirakawa-Oiwake-cho, Sakyo-ku, Kyoto 606-8502, Japan

[3] Department of Chemistry, University of Bath, Claverton Down, Bath, BA2 7AY United Kingdom

[4] Institute of Advanced Sciences, Yokohama National University, 79-5 Tokiwadai, Hodogaya-ku, Yokohama 240-8501, Japan

[5] Department of Science, University of Toyama, 3190 Gofuku, Toyama 930-0887, Japan

[6] Department of Pharmaceutical Sciences at Narita, International University of Health and Welfare, 4-3 Kozunomori, Narita City, Chiba 286-8686, Japan

[7] Department of Multidisciplinary Science, Graduate School of Arts and Sciences, The University of Tokyo, Komaba, Meguro-ku, Tokyo 153-8902, Japan

[8] PRESTO, Japan Science and Technology Agency (JST), 4-1-8 Honcho, Kawaguchi, Saitama 332-0012, Japan

Contents

1. General Considerations ***4***

2. General Experimental Procedure ***5***

2.1 Interior parts of the reactor **5**

2.2 Electrochemical ring expansion through carbon atom insertion using a divided cell 7

3. Substrate Preparation Procedure ***21***

3.1. Preparation of *N*-Boc-pyrroles **21**

3.2. Preparation of *N*-Ac-pyrroles **25**

3.3. Preparation of ethyl 3,4-diethyl-5-methyl-1-(2,2,2-trichloroacetyl)-1*H*-pyrrole-2-carboxylate (1a-TCA) **27**

3.4 Synthesis and Characterization of *N*-TFA-pyrroles **28**

3.4.1 Preparation of *N*-TFA-pyrroles **28**

3.4.2 Preparation of 2,2,2-trifluoro-1-(2,3,4,5-tetraphenyl-1*H*-pyrrol-1-yl) ethan-1-one (1j-TFA) **32**

3.5. Synthesis of 2-aroyl-3–5-dimethyl-4-ethyl-1*H*-pyrroles (1f) **33**

3.6. Preparation of 2-(4-fluorobenzoyl)-3–5-dimethyl-4-ethyl-1H-pyrrole (1g) **33**

3.7. Preparation of 5-Ethyl-1,1-difluoro-2-mesityl-4,6-dimethyl-1*H*-pyrrolo[1,2-c][1,3,2]diazaboryl-2-ium-1-uide (BF_2 complex of 2-iminopyrrole) (1h) **33**

3.8. Preparation of 2-cyclopropyl-3-(4-fluorophenyl)-1*H*-indole (1q) **34**

3.9. *N*-H-pyridinium salt **36**

3.10. Hydrolysis of diethyl 3,5-diethyl-6-methylpyridine-2,4-dicarboxylate (2ad) **37**

3.11. Synthesis of NHPI esters (2u) **38**

3.12. Preparation of dimethyl 4-ethyl-2,6-dimethylpyridine-3,5-dicarboxylate (Addit). **39**

3.13. Synthesis of methyl 4-ethyl-2,5-dimethyl-1H-pyrrole-3-carboxylate (1s-H) **39**

4. Supporting Data ***40***

4.1 Reaction condition optimization **40**

4.2 General procedure for the CV experiments **42**

4.3 General procedure for the *in situ* electron paramagnetic resonance experiments **52**

4.4 Results of energy calculations **54**

4.5. Single-crystal X-ray diffraction **57**

4.6. Expansion of synthetic utility **59**

4.7. Limitations **62**

5. Supporting References ***63***

6. NMR spectra ***65***

7. Coordinates for compounds and transition states ***144***

7.1 Coordinates for Δ*G*‡ (298.15 K) of the addition reaction of the ethyl diazoacetate (4.4.1)......144

7.2 Coordinates for transition states of dissociation of N_2 after the addition reaction (Figure 5D) 151

7.3 Coordinates for transition states on the energy diagram of 1a-TFA......153

7.4 Coordinates for transition states on the energy diagram of 1j-H 170

7.5 Coordinates for transition states on the energy diagram of 1a-H......190

1. General Considerations

Reagents and dry solvents were obtained from commercial sources and used without further purification. Reactions were conducted under an inert atmosphere using the Schlenk technique unless stated otherwise. ^{1}H and ^{13}C nuclear magnetic resonance (NMR) spectra were recorded on JEOL ECA500 (^{1}H: 500 MHz, ^{13}C: 126 MHz), JEOL ECX400 (^{1}H: 400 MHz, ^{13}C: 100 MHz), and Bruker DRX 500 (^{1}H: 500 MHz) spectrometers with $CDCl_3$ or CD_3CN as the solvent. The chemical shifts for ^{1}H and ^{13}C NMR spectra are reported in δ (ppm) relative to internal tetramethylsilane and the deuterated solvent, respectively. Multiplicities are denoted as singlet (s), doublet (d), triplet (t), quartet (q), or multiplet (m).

Electrospray-ionization time-of-flight mass spectra (ESI-TOF MS) were acquired using a Bruker Daltonics microTOF focus II, Exactive Orbitrap, and Nano Frontier LD.

Single crystal X-ray diffraction data were collected with a Rigaku XtaLAB PRO diffractometer using multi-layer mirror monochromated Cu-Kα radiation (λ = 1.54184 Å). The structures were solved employing a dual-space algorithm, SHELXT (SHELX2014), and refined through a full-matrix least-squares method (SHELXL).[1,2]

Electron paramagnetic resonance (EPR) spectroscopy measurements were conducted using a JES-FA200 X-band electron spin resonance spectrometer (JEOL). The experimental magnetic field was calibrated with an Echo Electronics EFM-2000AX proton NMR gauss meter.

Cyclic voltammetry (CV) measurements were conducted with the VSP-3A (Biologic). A three-electrode system, including a glassy carbon (GC) disk as a working electrode (φ = 3.0 mm), a Pt plate as a counter electrode (20 mm × 20 mm), and a $Ag/AgNO_3$ reference electrode (containing 10 mM AgNO3 and 0.1 M Bu_4NClO_4 in acetonitrile), was used for the CV measurements. Constant current electrolysis was performed using the galvanostat HABF-501A (Hokuto Denko).

The flow synthesis was conducted using a commercially available flow cell system (VF2, EC frontier). Images of the flow electrolysis setup are depicted in Section **2.1**. This cell consisted of a working-electrode compartment and a counter-electrode compartment, divided by a porous glass. The surface area of the carbon felt is 1900 cm^{2}. A Pt wire was inserted into the column for electrical connection. A Pt spiral wire was positioned in the counter-electrode compartment as a counter electrode. The electrolysis was conducted by applying a constant current of 10 mA.

2. General Experimental Procedure

2.1 Interior parts of the reactor

2.1.1 Interior parts of the divided cell and electrodes

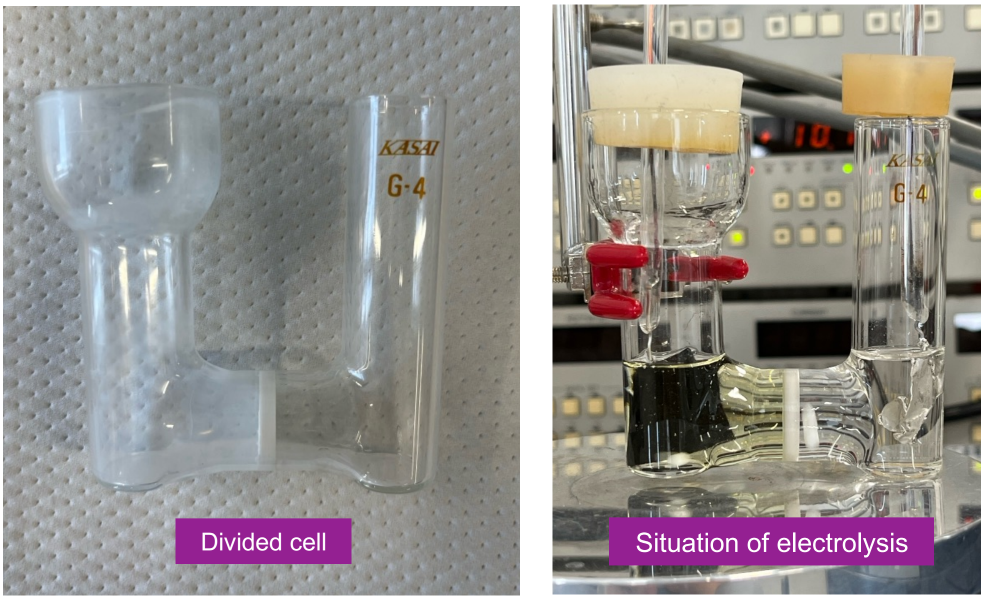

FigureS1. Schematic of the divided cell and the electrolysis setup.

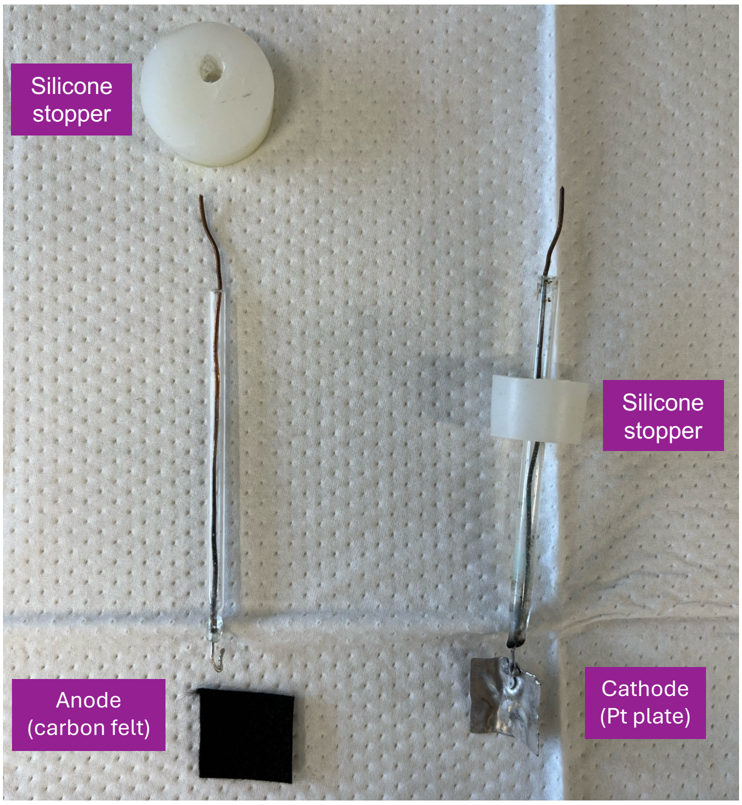

FigureS2. Structural configuration of the electrodes used in the divided cell.

2.1.2 Interior parts of the flow reactor

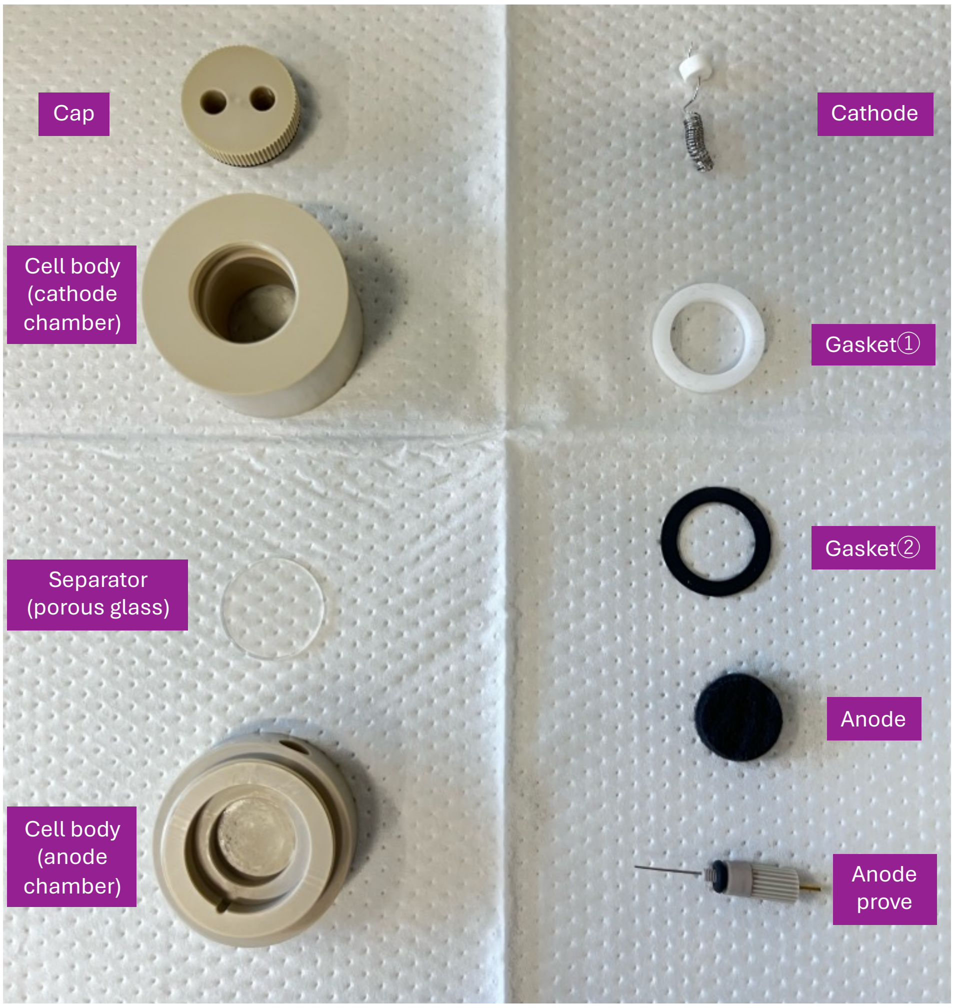

FigureS3. Internal components of the flow reactor

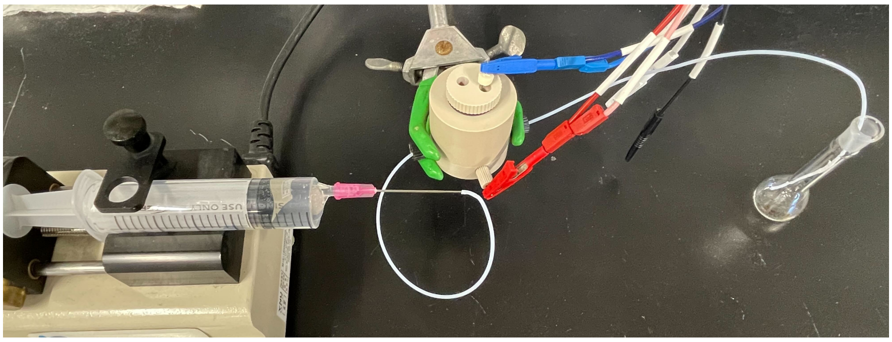

FigureS4. Schematic representation of the flow electrolysis setup within the reactor.

2.2 Electrochemical ring expansion through carbon atom insertion using a divided cell

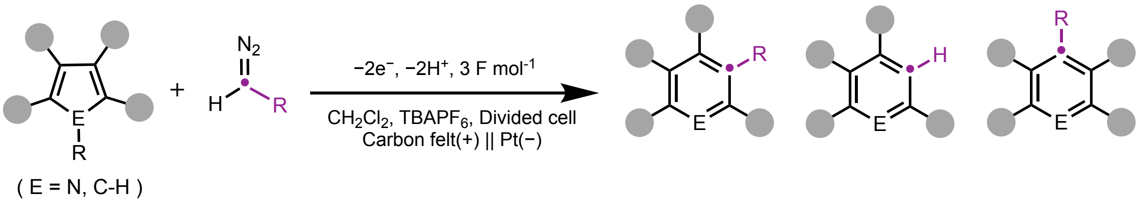

Pyrrole, imidazole, indole, cyclopentadiene, or indene derivatives (0.15 mmol, 15 mM) and 7 equiv. diazo compounds (1.05 mmol, 105 mM) were dissolved in a 10 mL electrolyte solution consisting of 0.1 M Bu_4NPF_6/CH_2Cl_2. The electrolysis was conducted in a two-electrode setup within the divided cell. Carbon felt was employed as the anode (2 cm × 2 cm), and a Pt plate served as the cathode (2 cm × 2 cm). A constant current of 10 mA was applied during electrolysis with vigorous stirring. Following electrolysis, the electrolyte solution was collected, and the solvent was evaporated under reduced pressure. The resulting mixture was washed with aq. $NaHCO_3$, and the organic layer was dried over Na_2SO_4. After filtration, the solution was concentrated under reduced pressure. To eliminate the electrolyte (Bu_4NPF_6), the crude material obtained was purified via silica gel flash column chromatography using diethyl ether, and the yield was determined by ^1H NMR with benzaldehyde as an internal standard. Purification of the crude material was repeated as necessary using silica gel column chromatography.

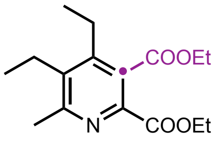

Diethyl 4,5-diethyl-6-methylpyridine-2,3-dicarboxylate (2aa)

Using 3,4-diethyl-2-ethoxycarbonyl-5-methylpyrrole (**1a-H**) (31.4 mg, 0.15 mmol) and 7 equiv. ethyl diazoacetate (containing ≥13 wt. % dichloromethane, 127 μL), compound **2aa** was synthesized in a 6% yield as a colorless oil (2.2 mg, 7.5 μmol).

^1H NMR (500MHz, $CDCl_3$, ppm): δ = 4.42 (q, J = 7.2 Hz, 2H), 4.41 (q, J = 7.1 Hz, 2H), 2.74 (q, J = 7.6 Hz, 2H), 2.67 (q, J = 7.6 Hz, 2H), 2.64 (s, 3H), 1.40 (t, J = 7.1 Hz, 3H), 1.38 (t, J = 7.2 Hz, 3H), 1.21 (t, J = 7.6 Hz, 3H), 1.17 (t, J = 7.5 Hz, 3H).

^{13}C NMR (126 MHz, $CDCl_3$, ppm): δ = 168.46, 165.45, 158.07, 148.74, 142.60, 139.33, 130.24, 62.25, 61.84, 23.46, 23.01, 21.98, 15.28, 14.36, 14.17, 13.85.

HRMS (ESI-TOF) for $C_{16}H_{24}NO_4^+$ $[M+H]^+$: calculated = 294.1705, observed = 294.1707.

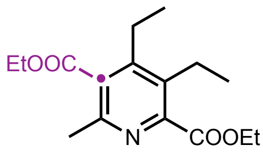

Diethyl 3,4-diethyl-6-methylpyridine-2,5-dicarboxylate (2ab)

Using 3,4-diethyl-2-ethoxycarbonyl-5-methylpyrrole (**1a-H**) (31.4 mg, 0.15 mmol) and 7 equiv. ethyl diazoacetate (containing ≥13 wt. % dichloromethane, 127 µL), compound **2ab** was synthesized in a 5% yield as a colorless oil (2.2 mg, 7.5 µmol).

^{1}H NMR (500 MHz, $CDCl_3$, ppm): δ = 4.45 (q, J = 7.1 Hz, 2H), 4.43 (q, J = 7.1 Hz, 2H), 2.77 (q, J = 7.5 Hz, 2H), 2.50 (s, 3H), 1.41 (t, J = 7.1 Hz, 3H), 1.40 (t, J = 7.2 Hz, 3H), 1.21 (t, J = 7.5 Hz, 3H), 1.19 (t, J = 7.6 Hz, 3H).

^{13}C NMR (126 MHz, $CDCl_3$, ppm): δ = 168.88, 167.37, 152.14, 150.32, 149.44, 133.61, 131.68, 62.01, 61.78, 23.50, 22.58, 21.46, 15.76, 15.24, 14.33, and 14.30.

HRMS (ESI-TOF) for $C_{16}H_{24}NO_4^+$ $[M+H]^+$: calculated = 294.1705, observed = 294.1703.

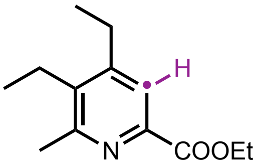

Ethyl 4,5-diethyl-6-methylpicolinate (2ac)

Using 1-(*tert*-butyl)-2-ethyl-3,4-diethyl-5-methyl-1*H*-pyrrole-1,2-dicarboxylate (**1a-Boc**) (46.4 mg, 0.15 mmol) and 7 equiv. ethyl diazoacetate (containing ≥ 13 wt. % dichloromethane, 127 µL), compound **2ac** was obtained in a 17% yield as a colorless oil (5.6 mg, 19.5 µmol).

^{1}H NMR (500MHz, $CDCl_3$, ppm): δ = 7.77 (s, 1H), 4.43 (q, J = 7.1 Hz, 2H), 2.69 (q, J = 7.5 Hz, 2H), 2.67 (q, J = 7.7 Hz, 2H), 2.63 (s, 3H), 1.40 (t, J = 7.1 Hz, 3H), 1.24 (t, J = 7.6 Hz, 3H), 1.12 (t, J = 7.6 Hz, 3H)

^{13}C NMR (126 MHz, $CDCl_3$, ppm): δ = 165.81, 157.19, 151.56, 144.82, 139.66, 123.29, 61.77, 25.28, 22.73, 21.82, 14.54, 14.42, 13.59.

HRMS (ESI-TOF) for $C_{13}H_{20}NO_2^+$ $[M+H]^+$: calculated = 222.1489, observed = 222.1485.

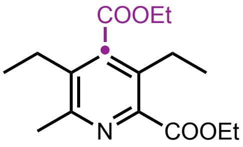

Diethyl 3,5-diethyl-6-methylpyridine-2,4-dicarboxylate (2ad)

Using ethyl 3,4-diethyl-5-methyl-1-(2,2,2-trifluoroacetyl)-1*H*-pyrrole-2-carboxylate (**1a-TFA**) (45.8 mg, 0.15 mmol) and 7 equiv. ethyl diazoacetate (containing ≥13 wt. % dichloromethane, 127 μL), compound **2ad** was obtained in a 55% yield as a light-yellow oil (24.2 mg, 82.5 μmol).

^{1}H NMR (500MHz, $CDCl_3$, ppm): δ = 4.43 (q, *J* = 7.1 Hz, 2H), 4.43 (q, *J* = 7.1 Hz, 2H), 2.73 (q, *J* = 7.5 Hz, 2H), 2.58 (q, *J* = 7.6 Hz, 2H), 2.58 (s, 3H), 1.40 (t, *J* = 7.1 Hz, 3H), 1.39 (t, *J* = 7.2 Hz, 3H), 1.20 (t, *J* = 7.5 Hz, 3H), 1.16 (t, *J* = 7.6 Hz, 3H).

^{13}C NMR (126 MHz, $CDCl_3$, ppm): δ = 168.13, 166.67, 155.03, 146.38, 143.43, 135.46, 132.89, 61.88, 61.78, 24.18, 23.60, 22.08, 15.79, 14.33, 14.30, 14.04.

HRMS (ESI-TOF) for $C_{16}H_{24}NO_4^+$ $[M+H]^+$: calculated = 294.1700, observed = 294.1696.

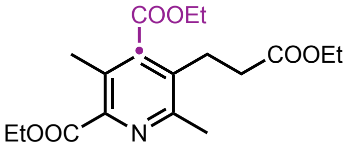

Diethyl 5-(3-ethoxy-3-oxopropyl)-3,6-dimethylpyridine-2,4-dicarboxylate (2b)

Using ethyl 4-(3-ethoxy-3-oxopropyl)-3,5-dimethyl-1-(2,2,2-trifluoroacetyl)-1*H*-pyrrole-2-carboxylate (**1b-TFA**) (56.6 mg, 0.15 mmol) and 7 equiv. of ethyl diazoacetate (containing ≥13 wt.% dichloromethane, 127 μL), compound **2b** was obtained in a 28% yield as a colorless oil (14.7 mg, 42.0 μmol).

^{1}H NMR (500MHz, $CDCl_3$, ppm): δ = 4.44 (q, *J* = 7.1 Hz, 2H), 4.43 (q, *J* = 7.1 Hz, 2H), 4.15 (q, *J* = 7.1 Hz, 2H), 2.94 – 2.84 (m, 2H), 2.59 (s, 1H), 2.55 – 2.48 (m, 2H), 2.37 (s, 1H), 1.41 (t, *J* = 7.1 Hz, 3H), 1.39 (t, *J* = 7.1 Hz, 3H), 1.26 (t, *J* = 7.1 Hz, 3H).

^{13}C NMR (126 MHz, $CDCl_3$, ppm): δ = 172.16, 167.80, 166.46, 155.00, 147.13, 144.28, 132.02, 127.29, 62.09, 61.94, 60.87, 33.93, 26.21, 22.12, 15.90, 14.32, 14.27, 14.23.

HRMS (ESI-TOF) for $C_{13}H_{20}NO_6^+$ $[M+H]^+$: calculated = 352.1751, observed = 352.1755.

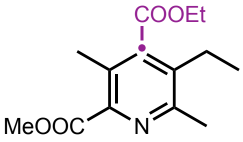

4-Ethyl 2-methyl 5-ethyl-3,6-dimethylpyridine-2,4-dicarboxylate (2c)

Using methyl 4-ethyl-3,5-dimethyl-1-(2,2,2-trifluoroacetyl)-1H-pyrrole-2-carboxylate (**1c-TFA**) (41.5 mg, 0.15 mmol) and 7 equiv. of ethyl diazoacetate (containing ≥13 wt.%

dichloromethane, 127 μL), compound **2c** was obtained as a colorless oil in a 63% yield (25.0 mg, 94.5 μmol).

^{1}H NMR (500 MHz, CD_3CN, ppm): δ = 4.41 (q, *J* = 7.1 Hz, 2H), 3.87 (s, 3H), 2.60 (q, *J* = 7.6 Hz, 2H), 2.51 (s, 3H), 2.31 (s, 3H), 1.35 (t, *J* = 7.1 Hz, 3H), 1.14 (t, *J* = 7.6 Hz, 3H).

^{13}C NMR (126 MHz, CD_3CN, ppm): δ = 168.65, 167.70, 155.82, 147.04, 144.67, 136.18, 127.42, 62.77, 52.86, 24.45, 21.88, 15.78, 14.35, 14.26.

HRMS (ESI-TOF) for $C_{14}H_{20}NO_4^+$ $[M+H]^+$: calculated = 266.1387, observed = 266.1394.

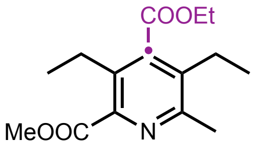

4-Ethyl 2-methyl 3,5-diethyl-6-methylpyridine-2,4-dicarboxylate (2d)

Using methyl 3,4-diethyl-5-methyl-1-(2,2,2-trifluoroacetyl)-1H-pyrrole-2-carboxylate (**1d-TFA**) (43.7 mg, 0.15 mmol) and 7 equiv. of ethyl diazoacetate (containing ≥13 wt.% dichloromethane, 127 μL), compound **2d** was obtained as a colorless oil in a 60% yield (25.2 mg, 90.3 μmol).

^{1}H NMR (500 MHz, $CDCl_3$, ppm): δ = 4.43 (q, *J* = 7.2 Hz, 2H), 3.95 (s, 3H), 2.76 (q, *J* = 7.5 Hz, 2H), 2.58 (q, *J* = 7.6 Hz, 2H), 2.58 (s, 3H), 1.39 (t, *J* = 7.1 Hz, 3H), 1.20 (t, *J* = 7.1 Hz, 3H), 1.16 (d, *J* = 7.1 Hz, 3H).

^{13}C NMR (126 MHz, $CDCl_3$, ppm): δ = 168.07, 166.72, 155.01, 145.39, 143.67, 135.86, 133.80, 61.80, 52.84, 24.21, 23.66, 22.07, 15.75, 14.29, 14.01.

HRMS (ESI-TOF) for $C_{15}H_{22}NO_4^+$ $[M+H]^+$: calculated = 280.1543, observed = 280.1544.

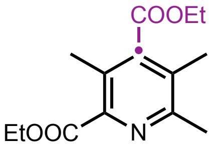

Diethyl 3,5,6-trimethylpyridine-2,4-dicarboxylate (2e)

Using methyl ethyl 3,4,5-trimethyl-1-(2,2,2-trifluoroacetyl)-1H-pyrrole-2-carboxylate (**1e-TFA**) (41.6 mg, 0.15 mmol) and 7 equiv. of ethyl diazoacetate (containing ≥13 wt.% dichloromethane, 127 μL), compound **2e** was obtained as a colorless oil in a 58% yield (23.1 mg, 87.0 μmol).

^{1}H NMR (500 MHz, $CDCl_3$, ppm): δ = 4.43 (q, *J* = 7.1 Hz, 2H), 2.53 (s, 3H), 2.38 (s, 3H), 2.23 (s, 3H), 1.40 (t, *J* = 7.1 Hz, 3H), 1.39 (t, *J* = 7.2 Hz, 3H).

^{13}C NMR (126 MHz, $CDCl_3$, ppm): δ = 168.18, 166.61, 155.36, 146.11, 144.03, 129.80, 127.17, 61.87, 61.84, 22.89, 16.38, 16.00, 14.37, 14.32.

HRMS (ESI-TOF) for $C_{14}H_{20}NO_4^+$ $[M+H]^+$: calculated = 266.1387, observed = 266.1390.

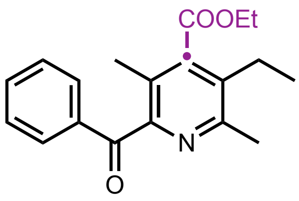

Ethyl 2-benzoyl-5-ethyl-3,6-dimethylisonicotinate (2f)

Using 1-(2-benzoyl-4-ethyl-3,5-dimethyl-1H-pyrrol-1-yl)-2,2,2-trifluoroethan-1-one (**1f-TFA**) (48.5 mg, 0.15 mmol) and 7 equiv. of ethyl diazoacetate (containing ≥13 wt.% dichloromethane, 127 µL), compound **2e** was obtained as a colorless oil in a 13% yield (6.1 mg, 19.5 µmol).

^{1}H NMR (400 MHz, $CDCl_3$, ppm): δ = 7.89–7.85 (m, 2H), 7.61–7.56 (m, 1H), 7.48–7.43 (m, 2H), 4.45 (q, J = 7.1 Hz, 2H), 2.64 (q, J = 7.5 Hz, 2H), 2.56 (s, 3H), 2.22 (s, 3H), 1.41 (t, J = 7.1 Hz, 3H), 1.23 (t, J = 7.6 Hz, 3H).

^{13}C NMR (101 MHz, $CDCl_3$, ppm): δ = 195.37, 168.33, 154.21, 153.02, 143.42, 136.26, 134.12, 133.72, 130.80, 128.57, 125.45, 61.87, 24.17, 21.94, 15.19, 14.38, 14.26.

HRMS (ESI-TOF) for $C_{19}H_{22}NO_3^+$ $[M+H]^+$: calculated = 312.1594, observed = 312.1596.

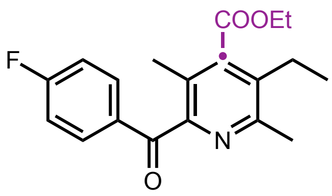

Ethyl 3-ethyl-6-(4-fluorobenzoyl)-2,5-dimethylisonicotinate (2g)

Using 1-(3-ethyl-5-(4-fluorobenzoyl)-2,4-dimethyl-1H-pyrrol-1-yl)-2,2,2-trifluoroethan-1-one (**1g-TFA**) (51.2 mg, 0.15 mmol) and 7 equiv. of ethyl diazoacetate (containing ≥13 wt.% dichloromethane, 127 µL), compound **2g** was obtained as a colorless oil in a 12% yield (5.9 mg, 18.0 µmol).

^{1}H NMR (400 MHz, $CDCl_3$, ppm): δ = 7.95–7.88 (m, 2H), 7.15–7.09 (m, 2H), 4.45 (q, J = 7.1 Hz, 2H), 2.64 (q, J = 7.9 Hz, 2H), 2.56 (s, 3H), 2.23 (s, 3H), 1.41 (t, J = 7.1 Hz, 3H), 1.23 (t, J = 7.5 Hz, 3H).

^{13}C NMR (101 MHz, $CDCl_3$, ppm): δ = 193.61, 168.21, 167.43, 164.88, 154.17, 152.54, 143.55, 134.31, 133.56, 133.47, 132.70, 125.69, 115.80, 115.59, 61.87, 24.14, 21.90, 15.16, 14.34, 14.20.

HRMS (ESI-TOF) for $C_{19}H_{21}NO_3^+$ $[M+H]^+$: calculated = 330.1500, observed = 330.1503.

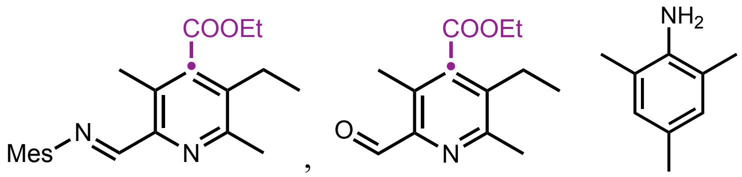

2h **2h'** **2h''**

Ethyl 3-ethyl-6-formyl-2,5-dimethylisonicotinate (2h)

Using 5-Ethyl-1,1-difluoro-2-mesityl-4,6-dimethyl-1*H*-pyrrolo[1,2-c][1,3,2]diazaboryl-2-ium-1-uide (**1h-BF$_2$**) (47.4 mg, 0.15 mmol) and 7 equiv. ethyl diazoacetate (containing ≥13 wt.% dichloromethane, 127 μL), compound **2h** was obtained as a brown oil in a 13% yield (6.9 mg, 19.5 μmol). As confirmed through NMR analysis, this compound contains 13% of **2h' and 2h''**.

^1H NMR (500MHz, $CDCl_3$, ppm): δ = 8.41 (s, 1H), 6.89 (s, 2H), 4.47 (q, J = 7.2 Hz, 2H), 2.69 – 2.57 (m, 8H), 2.28 (s, 3H), 2.13 (s, 6H), 1.43 (t, J = 7.1 Hz, 3H), 1.22 (t, J = 7.6 Hz, 4H).

^{13}C NMR (126 MHz, $CDCl_3$, ppm):δ = 168.76, 165.30, 154.66, 149.03, 148.93, 144.11, 133.84, 133.18, 128.87, 127.90, 126.61, 61.79, 24.17, 21.93, 20.87, 18.58, 17.72, 16.60, 14.23.

HRMS (ESI-TOF) for $C_{22}H_{29}N_2O_2^+$ $[M+H]^+$: calculated = 353.2224, observed = 353.2223.

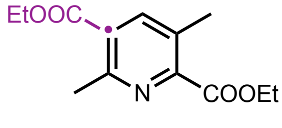

Diethyl 3,6-dimethylpyridine-2,5-dicarboxylate (2ia)

Using ethyl 3,5-dimethyl-1H-pyrrole-2-carboxylate (**1i-H**) (25.1 mg, 0.15 mmol) and 7 equiv. ethyl diazoacetate (containing ≥13 wt.% dichloromethane, 127 μL), compound **2ia** was obtained as a colorless oil in a 7% yield (2.6 mg, 10.5 μmol).

^1H NMR (500MHz, $CDCl_3$, ppm): δ = 8.07 (s, 1H), 4.46 (q, J = 7.1 Hz, 2H), 4.39 (q, J = 7.1 Hz, 2H), 2.82 (s, 3H), 2.52 (s, 1H), 1.43 (t, J = 7.1 Hz, 3H), 1.41 (t, J = 7.1 Hz, 3H).

^{13}C NMR (126 MHz, $CDCl_3$, ppm): δ = 166.26, 166.23, 156.89, 150.03, 141.90, 131.17, 127.38, 62.07, 61.70, 24.48, 18.99, 14.39.
HRMS (ESI-TOF) for $C_{13}H_{18}NO_4^+$ $[M+H]^+$: calculated = 252.1230, observed = 294.1226.

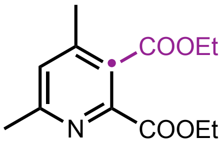

Diethyl 4,6-dimethylpyridine-2,3-dicarboxylate (2ib)

Using 1-(*tert*-butyl)-2-ethyl-3,5-dimethyl-1*H*-pyrrole-1,2-dicarboxylate (**1i-Boc**) (40.1 mg, 0.15 mmol) and 7 equiv. ethyl diazoacetate (containing ≥13 wt.% dichloromethane, 127 μL), compound **2ib** was obtained as a colorless oil in an 11% yield (4.1 mg, 16.5 μmol).
^{1}H NMR (500MHz, $CDCl_3$, ppm): δ = 7.17 (s, 1H), 4.43 (q, J = 7.1 Hz, 2H), 4.40 (q, J = 7.2 Hz, 2H), 2.60 (s, 3H), 2.38 (s, 3H), 1.43 – 1.35 (m, 6H).
^{13}C NMR (126 MHz, $CDCl_3$, ppm): δ = 167.57, 165.50, 159.26, 146.38, 146.28, 128.53, 127.39, 62.27, 61.76, 24.36, 19.15, 14.18, 14.05.
HRMS (ESI-TOF) for $C_{13}H_{18}NO_4^+$ $[M+H]^+$: calculated = 252.1230, observed = 294.1226.

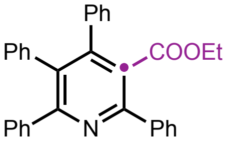

Ethyl 2,4,5,6-tetraphenylnicotinate (2ja)

Using 2,3,4,5-tetraphenylpyrrole (**1j-H**) (55.7 mg, 0.15 mmol) and 7 equiv. of ethyl diazoacetate (containing ≥13 wt. % dichloromethane, 127 μL), **2ja** was obtained as a colorless solid in a 37% yield (25.3 mg, 55.5 μmol).
^{1}H NMR (400MHz, $CDCl_3$, ppm): δ = 7.83–7.76 (m, 2H), 7.83–7.76 (m, 3H), 7.38–7.33 (m, 2H), 7.21–7.14 (m, 5H), 7.13–7.07 (m, 2H), 7.05–6.99 (m, 3H), 6.91–6.85 (m, 2H), 3.87 (q, J = 7.1 Hz, 2H), 0.83 (t, J = 7.1 Hz, 3H).
^{13}C NMR (126 MHz, $CDCl_3$, ppm): δ = 168.33, 158.11, 154.43, 148.59, 140.31, 139.74, 137.24, 136.95, 133.44, 131.20, 130.09, 129.42, 128.76, 128.74, 128.27, 128.25, 127.68, 127.57, 127.54, 127.48, 127.46, 126.62, 61.23, 13.41.
HRMS (ESI-TOF) for $C_{32}H_{25}NO_2^+$ $[M+H]^+$: calculated = 456.1958, observed = 456.1952.

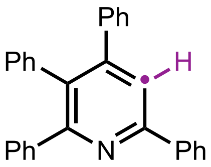

2,3,4,6-Tetraphenylpyridine (2jb)

Using 2,2,2-trifluoro-1-(2,3,4,5-tetraphenyl-1*H*-pyrrol-1-yl) ethan-1-one (**1j-TFA**) (70.1 mg, 0.15 mmol) and 7 equiv. of ethyl diazoacetate (containing ≥13 wt. % dichloromethane, 127 μL), **2jb** was obtained as a colorless solid in a 67% yield (38.5 mg, 100.5 μmol).

^{1}H NMR (500MHz, $CDCl_3$, ppm): δ = 8.21–8.16 (m, 2H), 7.79 (s, 1H), 7.52–7.47 (m, 2H), 7.45–7.36 (m, 3H), 7.25–7.19 (m, 6H), 7.16–7.12 (m, 2H), 7.11–7.04 (m, 3H), 6.95–6.91 (m, 2H).

^{13}C NMR (126 MHz, $CDCl_3$, ppm): δ = 158.12, 155.74, 150.77, 141.15, 140.00, 139.25, 138.03, 133.01, 131.58, 130.34, 129.48, 129.13, 128.83, 128.06, 127.84, 127.65, 127.49, 127.47, 127.20, 126.71, 120.46.

HRMS (ESI-TOF) for $C_{29}H_{22}N^+$ $[M+H]^+$: calculated = 384.1747, observed = 384.1740.

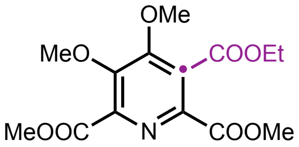

3-Ethyl 2,6-dimethyl 4,5-dimethoxypyridine-2,3,6-tricarboxylate (2k)

Using dimethyl 1-acetyl-3,4-dimethoxy-1H-pyrrole-2,5-dicarboxylate (**1k-Ac**) (42.8 mg, 0.15 mmol) and 7 equiv. of ethyl diazoacetate (containing ≥13 wt. % dichloromethane, 127 μL), **2k** was obtained as a colorless oil in a 47% yield (23.1 mg, 70.5 μmol).

^{1}H NMR (400MHz, $CDCl_3$, ppm): δ = 4.46 (q, J = 7.2 Hz, 2H), 4.05 (s, 3H), 3.99 (s, 3H), 3.99 (s, 3H), 3.95 (s, 3H), 1.40 (t, J = 7.2 Hz, 3H).

^{13}C NMR (101 MHz, $CDCl_3$, ppm): δ = 164.38, 164.27, 163.85, 158.06, 150.94, 145.81, 140.53, 129.96, 62.56, 62.38, 61.81, 53.52, 53.30, 14.17.

HRMS (ESI-TOF) for $C_{14}H_{18}NO_8^+$ $[M+H]^+$: calculated = 328.1027, observed = 328.1029.

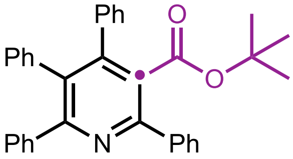

***tert*-Butyl 2,4,5,6-tetraphenylnicotinate (2l)**

Using 2,3,4,5-tetraphenylpyrrole (**1d-H**) (55.7 mg, 0.15 mmol) and 7 equiv. *tert*-butyl diazoacetate (149 mg, 1.05 mmol), compound **2l** was obtained as a white solid in an 11% yield (8.0 mg, 16.5 μmol).

^{1}H NMR (500MHz, $CDCl_3$, ppm): δ = 7.83–7.78 (m, 2H), 7.46–7.39 (m, 3H), 7.36–7.32 (m, 2H), 7.19–7.15 (m, 6H), 7.13–7.09 (m, 2H), 7.03–6.98 (m, 3H), 6.91–6.86 (m, 2H), 1.05 (s, 9H).

^{13}C NMR (126 MHz, $CDCl_3$, ppm): δ = 167.27, 157.73, 154.43, 148.36, 140.50, 139.94, 137.46, 137.13, 133.63, 131.32, 130.23, 129.82, 129.50, 129.15, 128.79, 128.28, 127.79, 127.76, 127.72, 127.64, 127.57, 127.50, 126.70, 82.59, 27.50.

HRMS (ESI-TOF) for $C_{34}H_{30}NO_2^+$ [M+H]$^+$: calculated = 484.2271, observed = 484.2277.

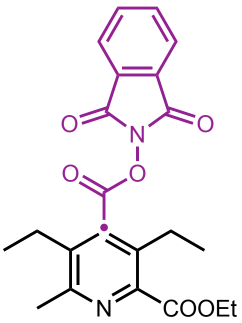

4-(1,3-Dioxo-2,3-dihydro-1*H*-inden-2-yl) 2-ethyl 3,5-diethyl-6-methylpyridine-2,4-dicarboxylate (2m)

Using ethyl 3,4-diethyl-5-methyl-1-(2,2,2-trichloroacetyl)-1H-pyrrole-2-carboxylate (**1a-TCA**) (52.9 mg, 0.15 mmol) and 7 equiv. 1,3-dioxoisoindolin-2-yl 2-diazoacetate (242.6 mg, 1.05 mmol), compound **2m** was obtained as a light-yellow solid in a 25% yield (15.2 mg, 37.5 μmol).

^{1}H NMR (500MHz, $CDCl_3$, ppm): δ = 7.96–7.93 (m, 2H), 7.85–7.83 (m, 2H), 4.48 (q, J = 7.1 Hz, 2H), 3.03 (q, J = 7.5 Hz, 2H), 2.90 (q, J = 7.5 Hz, 2H), 2.66 (s, 3H), 1.44 (t, J = 7.1 Hz, 3H), 1.32 – 1.25 (m, 6H).

^{13}C NMR (126 MHz, $CDCl_3$, ppm): δ = 166.37, 164.45, 161.92, 155.56, 146.82, 138.18, 137.21, 135.18, 134.31, 129.07, 124.32, 62.10, 24.40, 23.82, 22.09, 16.18, 14.36.

HRMS (ESI-TOF) for $C_{22}H_{23}N_2O_6^+$ $[M+H]^+$: calculated = 411.1551, observed = 411.1548.

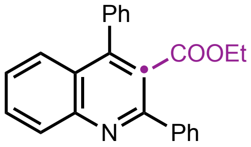

Ethyl 2,4-diphenylquinoline-3-carboxylate (2n)

Prepared from *tert*-butyl 2,3-diphenyl-1*H*-indole-1-carboxylate (**1n-Boc**) (55.4 mg, 0.15 mmol) and 7 equiv. of ethyl diazoacetate (containing ≥13 wt. % dichloromethane, 127 µL), compound **2n** was obtained as a yellow solid in a 58% yield (30.8 mg, 87.0 µmol).

^{1}H NMR (500MHz, $CDCl_3$, ppm): δ = 8.23 (m, *J* = 8.5, 1.3, 0.7 Hz, 1H), 7.79 – 7.71 (m, 3H), 7.62 (m, *J* = 8.4, 1.5, 0.7 Hz, 1H), 7.52 – 7.39 (m, 9H), 3.87 (q, *J* = 7.1 Hz, 2H), 0.81 (t, *J* = 7.1 Hz, 3H). The ^{1}H NMR spectrum was consistent with the published data.[3]

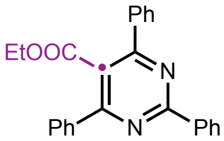

Ethyl 2,4,6-triphenylpyrimidine-5-carboxylate (2o)

Using *tert*-butyl 2,4,5-triphenyl-1H-imidazole-1-carboxylate (**1o-Boc**) (59.4 mg, 0.15 mmol) and 7 equiv. of ethyl diazoacetate (containing ≥13 wt.% dichloromethane, 127 µL), compound **2o** was obtained as a white solid in a 15% yield (8.6 mg, 22.5 µmol).

^{1}H NMR (400 MHz, $CDCl_3$, ppm): δ = 8.67–8.58 (m, 2H), 7.87–7.74 (m, 4H), 7.51 (dt, *J* = 4.9, 2.5 Hz, 9H), 4.09 (q, *J* = 7.2 Hz, 2H), 0.98 (t, *J* = 7.2 Hz, 3H). ^{1}H NMR spectrum corresponded to the reported data.[4]

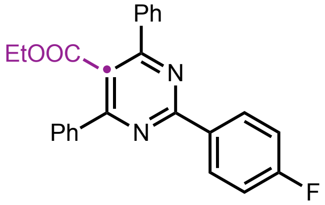

Ethyl 2-(4-fluorophenyl)-4,6-diphenylpyrimidine-5-carboxylate (2p)

Using *tert*-butyl 2,4,5-triphenyl-1H-imidazole-1-carboxylate (**1p-Boc**) (59.4 mg, 0.15 mmol) and 7 equiv. ethyl diazoacetate (containing at least 13 wt. % dichloromethane, 127 µL), compound **2p** was obtained as a white solid in a 13% yield (7.8 mg, 19.5 µmol).

^{1}H NMR (500 MHz, $CDCl_3$, ppm): δ = 8.67–8.61 (m, 2H), 7.83–7.74 (m, 4H), 7.56–7.46 (m, 6H), 7.21–7.13 (m, 2H), 4.09 (q, J = 7.1 Hz, 2H), 0.98 (t, J = 7.1 Hz, 3H).
^{13}C NMR (126 MHz, $CDCl_3$, ppm): δ = 168.51, 166.17, 164.68, 164.17, 163.04, 138.06, 133.44, 131.13, 131.06, 130.21, 128.66, 123.19, 115.72, 115.55, 62.04, 13.61.
HRMS (ESI-TOF) for $C_{25}H_{20}N_2O_2F^+$ $[M+H]^+$: calculated = 399.1503, observed = 399.1508.

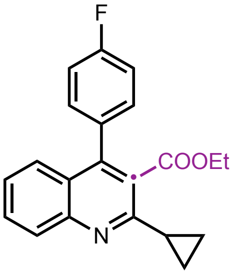

Ethyl 2-cyclopropyl-4-(4-fluorophenyl) quinoline-3-carboxylate (2q)
Prepared from *tert*-butyl 2-cyclopropyl-3-(4-fluorophenyl)-1*H*-indole-1-carboxylate (**1q-Boc**) (52.7 mg, 0.15 mmol) and 7 equiv. of ethyl diazoacetate (containing at least 13 wt. % dichloromethane, 127 µL), compound **2q** was obtained as a colorless oil in a 12% yield (6.0 mg, 18.0 µmol).
^{1}H NMR (400 MHz, $CDCl_3$, ppm): δ = 8.01–7.95 (m, 1H), 7.69–7.63 (m, 1H), 7.5–7.46 (m, 1H), 7.40–7.33 (m, 3H), 7.22–7.16 (m, 2H), 4.11 (q, J = 7.1 Hz, 2H), 2.27–2.18 (m, 1H), 1.39–1.33 (m, 2H), 1.08–1.00 (m, 5H). The obtained ^{1}H NMR spectrum corresponded to the reported data.[5]

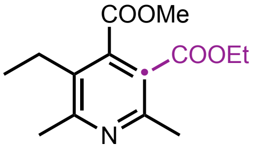

3-Ethyl 4-methyl 5-ethyl-2,6-dimethylpyridine-3,4-dicarboxylate (2s)
Using 1-(*tert*-butyl) 3-methyl 4-ethyl-2,5-dimethyl-1*H*-pyrrole-1,3-dicarboxylate (**1s-Boc**) (42.2 mg, 0.15 mmol) and 7 equiv. of ethyl diazoacetate (containing ≥13 wt.% dichloromethane, 127 µL), compound **2s** was obtained as a clear oil in a 27% yield (10.7 mg, 40.5 µmol).
^{1}H NMR (400MHz, $CDCl_3$, ppm): δ = 4.33 (q, J = 7.1 Hz, 2H), 3.89 (s, 3H), 2.65 – 2.58 (m, 8H), 1.36 (t, J = 7.2 Hz, 3H), 1.16 (t, J = 7.5 Hz, 3H).

^{13}C NMR (101 MHz, $CDCl_3$, ppm): δ = 168.21, 167.18, 159.69, 154.66, 141.17, 131.34, 122.50, 61.89, 52.62, 23.88, 23.35, 22.60, 14.34, 14.21.
HRMS (ESI-TOF) for $C_{14}H_{20}NO_4^+$ $[M+H]^+$: calculated = 266.1387, observed = 266.1386.

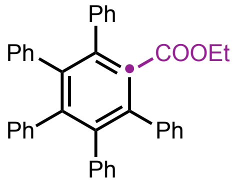

Ethyl 2,3,4,5,6-pentaphenylbenzoate (4a)

Using 1,2,3,4,5-Pentaphenyl-1,3-cyclopentadiene (**3a**) (66.9 mg, 0.15 mmol) and 7 equiv. ethyl diazoacetate (containing ≥13 wt.% dichloromethane, 127 µL), compound **4a** was obtained as a white solid in a 61% yield (48.5 mg, 91.5 µmol).
^1H NMR (500 MHz, $CDCl_3$, ppm): δ = 7.19–7.08 (m, 10H), 6.91–6.73 (m, 15H), 3.67 (q, J = 7.1 Hz, 2H), 0.71 (t, J = 7.1 Hz, 3H).
^{13}C NMR (126 MHz, $CDCl_3$, ppm): δ = 169.05, 142.44, 140.68, 140.06, 139.59, 139.36, 138.09, 135.13, 131.41, 131.28, 130.32, 127.39, 126.89, 126.82, 126.78, 125.75, 125.66, 60.77, 13.55.
The obtained ^1H, ^{13}C NMR spectrum corresponded to the reported data.[6]

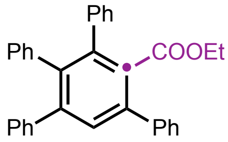

Ethyl 4',6'-diphenyl-[1,1':2',1''-terphenyl]-3'-carboxylate (4b)

Prepared from 2,3,4,5-tetraphenyl-1,3-cyclopentadiene (**3b**) (55.5 mg, 0.15 mmol) and 7 equiv. of ethyl diazoacetate (containing ≥13 wt.% dichloromethane, 127 µL), compound **4a** was obtained as a white solid in a 23% yield (15.7 mg, 34.5 µmol).
^1H NMR (500 MHz, $CDCl_3$, ppm): δ = 7.55–7.49 (m, 4H), 7.45–7.33 (m, 3H), 7.19–7.07 (m, 10H), 6.94 (m, 3H), 6.82 (m, 2H), 3.78 (q, J = 7.1 Hz, 2H), 0.77 (t, J = 7.1 Hz, 3H).
^{13}C NMR (126 MHz, $CDCl_3$, ppm): δ = 169.23, 142.87, 141.29, 140.34, 139.89, 139.36, 139.21, 138.90, 138.79, 133.63, 131.54, 131.26, 131.09, 130.34, 129.99, 129.93, 128.73, 128.48, 127.80, 127.77, 127.39, 127.28, 127.21, 126.83, 126.72, 126.11, 60.96, 13.58.
HRMS (ESI-TOF) for $C_{33}H_{26}O_2^+$ $[M+H]^+$: calculated = 455.2006, observed = 455.2003.

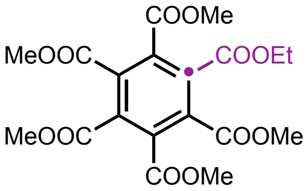

1-Ethyl 2,3,4,5,6-pentamethyl benzene-1,2,3,4,5,6-hexacarboxylate (4ca)

Using pentamethylcyclopenta-1,3-diene-1,2,3,4,5-pentacarboxylate (**3c**) (53.4 mg, 0.15 mmol) and 7 equiv. of ethyl diazoacetate (containing ≥13 wt. % dichloromethane, 127 μL), **4ca** was obtained as a colorless solid in a 21% yield (13.9 mg, 31.5 μmol).

^{1}H NMR (500 MHz, $CDCl_3$, ppm): δ = 4.34 (q, J = 7.1 Hz, 2H), 3.88 (s, 3H), 3.88 (s, 3H), 3.88 (s, 6H), 1.34 (t, J = 7.2 Hz, 3H).

^{13}C NMR (126 MHz, $CDCl_3$, ppm): δ = 165.33, 164.80, 134.21, 134.10, 133.94, 63.06, 53.62, 53.52, 13.97.

HRMS (ESI-TOF) for $C_{19}H_{21}O_{12}^{+}$ $[M+H]^{+}$: calculated = 441.1028, observed 441.1030.

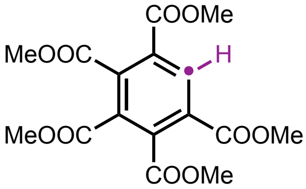

Pentamethyl benzene-1,2,3,4,5-pentacarboxylate (4cb)

Using pentamethyl cyclopenta-1,3-diene-1,2,3,4,5-pentacarboxylate (**3c**) (53.4 mg, 0.15 mmol) and 7 equiv. of ethyl diazoacetate (containing ≥13 wt. % dichloromethane, 127 μL), **4ca** was obtained as a colorless solid in an 11% yield (6.1 mg, 16.5 μmol).

^{1}H NMR (500 MHz, $CDCl_3$, ppm): δ = 8.66 (s, 1H), 3.95 (s, 6H), 3.94 (s, 6H), 3.88 (s, 3H).

^{13}C NMR (126 MHz, $CDCl_3$, ppm): δ = 166.98, 164.83, 164.26, 138.98, 134.38, 130.21, 53.57, 53.37, 53.34.

HRMS (ESI-TOF) for $C_{16}H_{17}O_{10}^{+}$ $[M+H]^{+}$: calculated = 369.0816, observed = 369.0814.

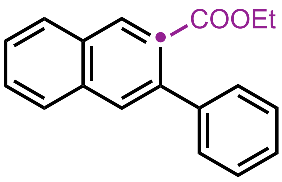

Ethyl-3-phenyl-2-naphthoate (4d)

Using 2-phenyl indene (**3d**) (28.8 mg, 0.15 mmol) and 7 equiv. of ethyl diazoacetate (containing ≥13 wt. % dichloromethane, 127 μL), compound **4d** was obtained in a 45% yield, as determined by ^{1}H NMR.[7]

3. Substrate Preparation Procedure

3.1. Preparation of *N*-Boc-pyrroles

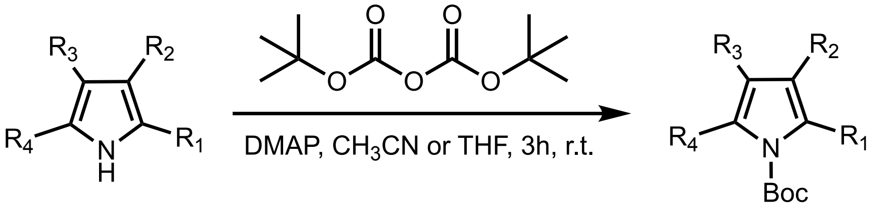

In a 5 mL round-bottomed flask, pyrroles (1 mmol) and 4-dimethylaminopyridine (DMAP) (18.3 mg, 0.15 equiv.) were added to dry CH_3CN or THF (1 mL) at room temperature under a nitrogen atmosphere. After stirring for 3 h, the reaction mixture was concentrated under reduced pressure. The crude material obtained was purified by silica gel column chromatography using hexane/ethyl acetate (95/5) to yield a spectroscopically pure product.

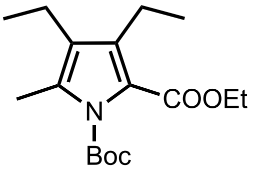

1-(*tert*-butyl) 2-ethyl 3,4-diethyl-5-methyl-1*H*-pyrrole-1,2-dicarboxylate (1a-Boc)

Using 3,4-diethyl-2-ethoxycarbonyl-5-methylpyrrole (209 mg, 1 mmol) in dry CH_3CN, **1a-Boc** was obtained in a 98% yield as a colorless oil (303 mg, 0.98 mmol).

^{1}H NMR (500 MHz, $CDCl_3$, ppm): δ = 4.29 (q, J = 7.1 Hz, 2H), 2.59 (q, J = 7.5 Hz, 2H), 2.35 (q, J = 7.6 Hz, 2H), 2.28 (s, 3H), 1.56 (s, 9H), 1.34 (t, J = 7.1 Hz, 3H), 1.12 (t, J = 7.5 Hz, 3H), 1.05 (t, J = 7.6 Hz, 3H).

^{13}C NMR (126 MHz, $CDCl_3$, ppm): δ = 161.81, 150.07, 135.74, 131.69, 124.16, 120.07, 84.08, 60.32, 27.76, 18.22, 17.08, 15.81, 15.68, 14.45, 11.68.

HRMS (ESI-TOF) for $C_{17}H_{28}NO_4^+$ $[M+H]^+$: calculated = 310.2013, observed = 310.2010.

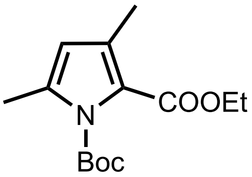

1-(*tert*-butyl) 2-ethyl 3,5-dimethyl-1*H*-pyrrole-1,2-dicarboxylate (1i-Boc)

Using ethyl 3,5-dimethyl-1*H*-pyrrole-2-carboxylate (167 mg, 1 mmol) in dry CH_3CN, **1i-Boc** was obtained in a 97% yield as a colorless oil (259 mg, 0.97 mmol).

^{1}H NMR (500 MHz, $CDCl_3$, ppm): δ = 5.75 (s, 1H), 4.29 (q, *J* = 7.1 Hz, 2H), 2.31 (s, 3H), 2.19 (s, 3H), 1.56 (s, 9H), 1.34 (t, *J* = 7.1 Hz, 3H).
^{13}C NMR (126 MHz, $CDCl_3$, ppm): δ = 161.62, 149.83, 135.74, 130.58, 121.42, 113.12, 84.28, 60.33, 27.73, 14.53, 14.16, 12.56.
HRMS (ESI-TOF) for $C_{14}H_{22}NO_4^+$ $[M+H]^+$: calculated = 268.1543, observed = 268.1539.

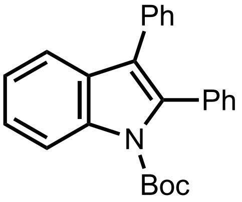

tert-butyl 2,3-diphenyl-1*H*-indole-1-carboxylate (1n-Boc)
Using 2,3-diphenyl-1*H*-indole (269 mg, 1 mmol) in dry THF, **1n-Boc** was obtained in 98% yield as a white solid (361 mg, 0.98 mmol).
^{1}H NMR (500 MHz, $CDCl_3$, ppm): δ = 8.30 (m, 1H), 7.61–7.55 (m, 1H), 7.42–7.35 (m, 1H), 7.31–7.24 (m, 8H), 7.24–7.19 (m, 3H), 1.26 (s, 9H).
^{13}C NMR (126 MHz, $CDCl_3$, ppm): δ = 150.45, 136.75, 135.88, 134.06, 133.55, 130.47, 130.29, 129.44, 128.26, 127.85, 127.58, 126.78, 124.86, 123.16, 122.05, 119.74, 115.34, 83.46, 27.63.
HRMS (ESI-TOF) for $C_{25}H_{24}NO_2^+$ $[M+H]^+$: calculated = 370.1802, observed = 370.1801.

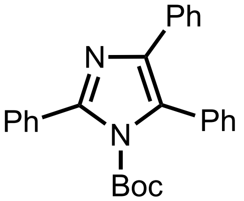

tert-butyl 2,4,5-triphenyl-1*H*-imidazole-1-carboxylate (1o-Boc)
Using 2,4,5-triphenyl-1*H*-imidazole (296 mg, 1 mmol) in dry CH_3CN, **1o-Boc** was obtained as a white solid in a 96% yield (380 mg, 0.96 mmol).
^{1}H NMR (500 MHz, $CDCl_3$, ppm): δ = 7.70–7.65 (m, 2H), 7.54–7.48 (m, 2H), 7.47–7.40 (m, 8H), 7.24–7.14 (m, 3H), 1.17 (s, 9H).
^{13}C NMR (126 MHz, $CDCl_3$, ppm): δ = 148.56, 148.00, 138.27, 133.63, 131.49, 131.36, 130.82, 129.23, 128.98, 128.70, 128.66, 128.48, 128.26, 128.20, 127.44, 127.08, 85.58, 27.10.
HRMS (ESI-TOF) for $C_{26}H_{25}N_2O_2^+$ $[M+H]^+$: calculated = 397.1911, observed = 397.1904.

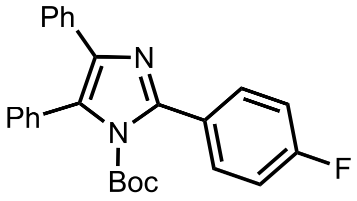

***tert*-butyl 2-(4-fluorophenyl)-4,5-diphenyl-1*H*-imidazole-1-carboxylate (1p-Boc)**

Using 2-(4-fluorophenyl)-4,5-diphenyl-1*H*-imidazole (296 mg, 1 mmol) in dry CH_3CN, **1p-Boc** was obtained as a white solid in a 96% yield (380 mg, 0.96 mmol).

^{1}H NMR (500MHz, $CDCl_3$, ppm): δ = 7.70–7.64 (m, 2H), 7.51–7.47 (m, 2H), 7.46–7.38 (m, 5H), 7.23–7.12 (m, 5H), 1.17 (s, 9H).

^{13}C NMR (126 MHz, $CDCl_3$, ppm): δ = 164.47, 162.49, 148.57, 147.16, 138.35, 133.52, 131.42, 131.11, 131.05, 130.82, 128.78, 128.73, 128.56, 128.27, 127.69, 127.66, 127.45, 127.21, 115.48, 115.31, 85.80, 27.18.

HRMS (ESI-TOF) for $C_{26}H_{24}N_2O_2F^+$ $[M+H]^+$: calculated = 415.1816, observed = 415.1817.

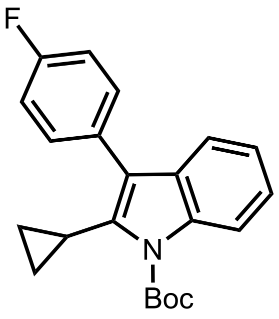

***tert*-butyl 2-cyclopropyl-3-(4-fluorophenyl)-1*H*-indole-1-carboxylate (1q-Boc)**

Using 2-cyclopropyl-3-(4-fluorophenyl)-1*H*-indole (251 mg, 1 mmol) in dry CH_3CN, **1q-Boc** was obtained as a white solid in a 99% (347 mg, 0.99 mmol).

^{1}H NMR (500MHz, $CDCl_3$, ppm): δ = 8.18–8.10 (m, 1H), 7.50–7.44 (m, 3H), 7.33–7.28 (m, 1H), 7.23–7.11 (m, 3H), 2.27–2.19 (m, 1H), 1.71 (s, 9H), 0.84–0.77 (m, 2H), 0.30 – 0.23 (m, 2H).

^{13}C NMR (126 MHz, $CDCl_3$, ppm): δ = 162.99, 161.03, 150.61, 137.80, 135.93, 131.80, 131.74, 130.29, 130.27, 128.83, 124.46, 122.83, 121.21, 119.00, 115.34, 115.17, 115.02, 83.63, 28.31, 10.08, 9.06.

^{19}F NMR (471 MHz, $CDCl_3$, ppm): δ = −115.41.

HRMS (ESI-TOF) for $C_{22}H_{23}NO_2F^+$ $[M+H]^+$: calculated = 352.1707, observed = 352.1717.

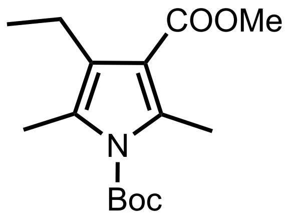

1-(tert-Butyl) 3-methyl 4-ethyl-2,5-dimethyl-1H-pyrrole-1,3-dicarboxylate (1s-Boc)

Using methyl 4-ethyl-2,5-dimethyl-1H-pyrrole-3-carboxylate (181 mg, 1 mmol) in dry THF, **1s-Boc** was obtained as a colorless oil in a 96% yield (270 mg, 0.96 mmol).

^{1}H NMR (400MHz, $CDCl_3$, ppm): δ = 3.81 (s, 3H), 2.62 (s, 3H), 2.58 (q, J = 7.4 Hz, 2H), 2.25 (s, 3H), 1.60 (s, 9H), 1.04 (t, J = 7.4 Hz, 3H).

^{13}C NMR (101 MHz, $CDCl_3$, ppm): δ = 166.38, 150.13, 136.82, 125.87, 124.49, 114.12, 84.47, 77.47, 77.15, 76.83, 50.95, 28.08, 18.46, 15.55, 14.29, 12.35.

HRMS (ESI-TOF) for $C_{14}H_{20}NO_4F^+$ $[M+H]^+$: calculated = 266.1387, observed = 266.1386

3.2. Preparation of *N*-Ac-pyrroles

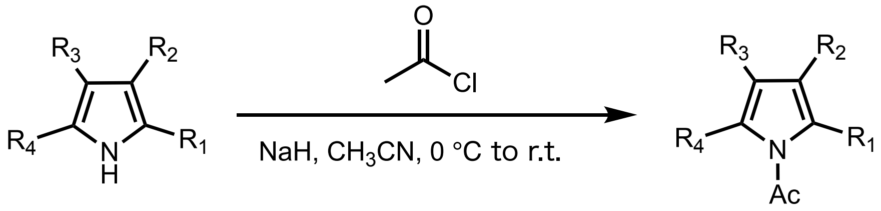

In a 50 mL round-bottomed flask, pyrroles (1 mmol) and NaH (60%, dispersion in paraffin liquid) (40 mg, 1.0 equiv.) were added to dry CH_3CN (10 mL) at 0 °C under a nitrogen atmosphere and stirred for 15 min. Acetyl chloride (107 μL, 1.5 mmol, 1.5 equiv.) was then added dropwise, and the reaction mixture was allowed to warm to room temperature. After stirring for 3 h, the reaction mixture was carefully poured into water to quench it, and the aqueous phase was extracted with ethyl acetate (EtOAc) three times. The combined organic layer was washed with water twice, brine once, and dried over Na_2SO_4. After filtration, the solution was concentrated under reduced pressure. The obtained crude material was purified by silica gel column chromatography using hexane/ethyl acetate (100/0 to 90/10) to yield a spectroscopically pure product.

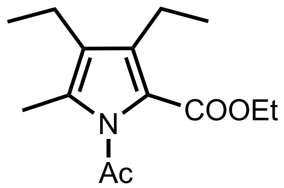

Ethyl 1-acetyl-3,4-diethyl-5-methyl-1*H*-pyrrole-2-carboxylate (1a-Ac)

Using 3,4-diethyl-2-ethoxycarbonyl-5-methylpyrrole (209 mg, 1 mmol), **1a-Ac** was obtained as a colorless oil in a 58% yield (145 mg, 0.58 mmol).

^{1}H NMR (500 MHz, $CDCl_3$, ppm): δ = 4.30 (q, J = 7.1 Hz, 3H), 2.67 (q, J = 7.5 Hz, 2H), 2.44 (s, 3H), 2.37 (q, J = 7.6 Hz, 2H), 2.23 (s, 3H), 1.35 (t, J = 7.1 Hz, 3H), 1.14 (t, J = 7.5 Hz, 3H), 1.06 (t, J = 7.6 Hz, 3H).

^{13}C NMR (126 MHz, $CDCl_3$, ppm): δ = 173.95, 161.49, 138.58, 132.67, 124.62, 118.85, 60.53, 28.53, 18.59, 17.02, 15.77, 15.67, 14.38, 11.54.

HRMS (ESI-TOF) for $C_{14}H_{22}NO_3^+$ $[M+H]^+$: calculated = 252.1594, observed = 252.1591.

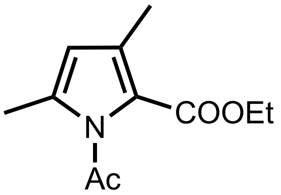

Ethyl 1-acetyl-3,5-dimethyl-1*H*-pyrrole-2-carboxylate (1i-Ac)

Using ethyl 3,5-dimethyl-1*H*-pyrrole-2-carboxylate (**1i**) (167 mg, 1 mmol), **1i-Ac** was obtained as a cloudy oil in a 17% yield (35 mg, 0.17 mmol).

^{1}H NMR (500 MHz, $CDCl_3$, ppm): δ = 5.79 (s, 1H), 4.29 (q, *J* = 7.1 Hz, 2H), 2.44 (s, 3H), 2.26 (s, 3H), 2.25 (s, 3H), 1.34 (t, *J* = 7.1 Hz, 3H).

^{13}C NMR (126 MHz, $CDCl_3$, ppm): δ = 173.58, 161.53, 136.25, 133.42, 120.29, 113.61, 60.54, 28.24, 14.46, 13.94, 13.23.

HRMS (ESI-TOF) for $C_{11}H_{16}NO_3^+$ $[M+H]^+$: calculated = 210.1125, observed = 210.1126.

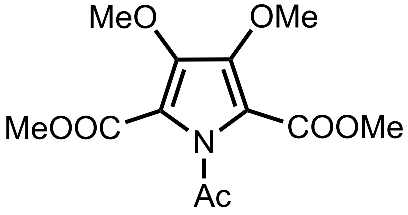

Dimethyl 1-acetyl-3,4-dimethoxy-1H-pyrrole-2,5-dicarboxylate (1k-Ac)

Using dimethyl 3,4-dimethoxy-1*H*-pyrrole-2,5-dicarboxylate[8] (**1k**) (243 mg, 1 mmol), **1k-Ac** was obtained as a colorless solid in a 70% yield (200 mg, 0.7 mmol).

^{1}H NMR (500MHz, $CDCl_3$, ppm): δ = 3.90 (s, 6H), 3.85 (s, 6H), 2.62 (s, 3H).

^{13}C NMR (126 MHz, $CDCl_3$, ppm): δ = 173.56, 160.22, 142.74, 115.01, 62.24, 52.33, 29.69, 28.88.

HRMS (ESI-TOF) for $C_9H_8N_{11}O^+$ $[M+H]^+$: calculated = 286.0908, observed = 286.0913.

3.3. Preparation of ethyl 3,4-diethyl-5-methyl-1-(2,2,2-trichloroacetyl)-1*H*-pyrrole-2-carboxylate (1a-TCA)

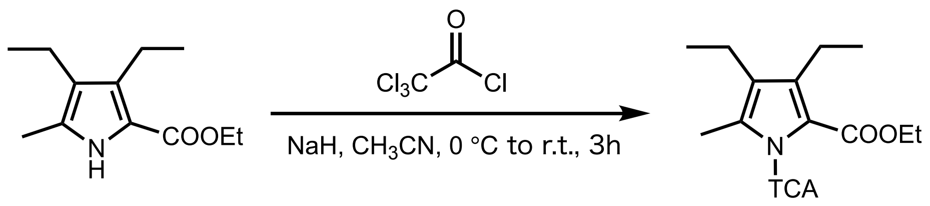

In a 50 mL round-bottomed flask, 3,4-diethyl-2-ethoxycarbonyl-5-methylpyrrole (209 mg, 1 mmol) and NaH (60%, dispersion in paraffin liquid) (40 mg, 1.0 equiv.) were added to dry CH_3CN (10 mL) at 0 °C under a nitrogen atmosphere and stirred for 15 min. Trichloroacetyl chloride (168 μL, 1.5 mmol, 1.5 equiv.) was then added dropwise, and the reaction mixture was allowed to warm to room temperature. After stirring for 3 h, the reaction mixture was carefully poured into water to quench it, and the aqueous phase was extracted with EtOAc three times. The combined organic layer was washed with water twice, brine once, and dried over Na_2SO_4. After filtration, the solution was concentrated under reduced pressure. The obtained crude material was purified by silica gel column chromatography using hexane/ethyl acetate (100/0 to 90/10) to yield a spectroscopically pure product as a light-yellow oil in 56% yield (198 mg, 0.56 mmol).

^{1}H NMR (500 MHz, $CDCl_3$, ppm): δ = 4.34 (q, J = 7.1 Hz, 2H), 2.71 (q, J = 7.5 Hz, 2H), 2.41 (q, J = 7.6 Hz, 2H), 2.26 (s, 3H), 1.35 (t, J = 7.1 Hz, 3H), 1.14 (t, J = 7.5 Hz, 3H), 1.10 (t, J = 7.6 Hz, 3H).

^{13}C NMR (126 MHz, $CDCl_3$, ppm): δ = 166.80, 161.94, 135.95, 132.27, 126.16, 121.90, 93.94, 61.33, 18.36, 17.33, 15.62, 15.56, 14.48, 10.17.

HRMS (ESI-TOF) for $C_{14}H_{19}NO_3Cl_3^+$ $[M+H]^+$: calculated = 354.0425, observed = 354.0431.

3.4 Synthesis and Characterization of *N*-TFA-pyrroles

3.4.1 Preparation of *N*-TFA-pyrroles

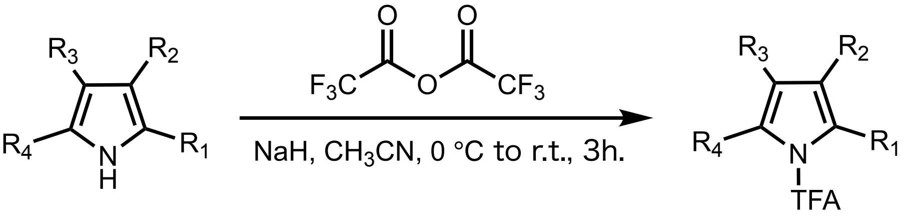

In a 50 mL round-bottomed flask, pyrroles (1 mmol) and NaH (60%, dispersion in paraffin liquid) (40 mg, 1.0 equiv.) were added to dry CH_3CN (10 mL) at 0 °C under a nitrogen atmosphere and stirred for 15 min. Trifluoroacetic anhydride (210 µL, 1.5 mmol, 1.5 equiv.) was then added dropwise, and the reaction mixture was allowed to warm to room temperature. After stirring for 3 h, the reaction mixture was carefully poured into water to quench it, and the aqueous phase was extracted with EtOAc three times. The combined organic layer was washed with water twice, brine once, and dried over sodium sulfate. After filtration, the solution was concentrated under reduced pressure. The obtained crude material was purified by silica gel column chromatography using a hexane/ethyl acetate gradient (100/0 to 90/10) to yield a spectroscopically pure product.

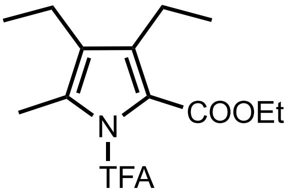

Ethyl 3,4-diethyl-5-methyl-1-(2,2,2-trifluoroacetyl)-1*H*-pyrrole-2-carboxylate (1a-TFA)

Using 3,4-diethyl-2-ethoxycarbonyl-5-methylpyrrole (209 mg, 1 mmol), **1a-TFA** was obtained as a colorless oil in a 56% yield (170 mg, 0.56 mmol).

^{1}H NMR (500MHz, $CDCl_3$, ppm): δ = 4.32 (q, J = 7.2 Hz, 2H), 2.70 (q, J = 7.5 Hz, 2H), 2.40 (q, J = 7.6 Hz, 2H), 2.18 (s, 3H), 1.15 (t, J = 7.5 Hz, 3H), 1.09 (t, J = 7.6 Hz, 3H).

^{13}C NMR (126 MHz, $CDCl_3$, ppm): δ = 161.51, 138.08, 132.46, 126.83, 120.45, 116.47, 114.18, 61.40, 18.35, 17.15, 15.48, 14.26, 10.22.

^{19}F NMR (471 MHz, $CDCl_3$, ppm): δ = −73.32.

HRMS (ESI-TOF) for $C_{14}H_{21}NO_4F_3^+$ $[M+H_3O]^+$: calculated = 324.1417, observed = 324.1422.

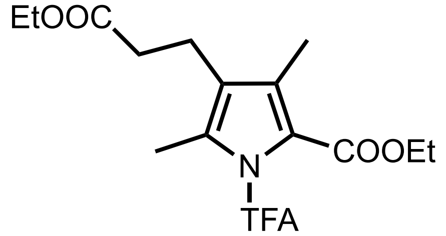

Ethyl 4-(3-ethoxy-3-oxopropyl)-3,5-dimethyl-1-(2,2,2-trifluoroacetyl)-1*H*-pyrrole-2-carboxylate (1b-TFA)

Using ethyl 4-(3-ethoxy-3-oxopropyl)-3,5-dimethyl-pyrrole-2-carboxylate (267 mg, 1 mmol), **1b-TFA** was obtained as a light-brown oil in a 53% yield (170 mg, 0.53 mmol).

^{1}H NMR (500MHz, CDCl$_3$, ppm): δ = 4.32 (q, J = 7.1 Hz, 2H), 4.12 (q, J = 7.1 Hz, 2H), 2.72 (t, J = 7.0 Hz, 2H), 2.43 (t, J = 7.1 Hz, 2H), 2.28 (s, 3H), 2.19 (s, 3H), 1.35 (t, J = 7.1 Hz, 3H), 1.24 (t, J = 7.1 Hz, 3H).

^{13}C NMR (126 MHz, CDCl$_3$, ppm): δ = 172.63, 161.63, 132.87, 131.41, 123.83, 121.23, 116.36, 114.07, 61.48, 60.66, 34.50, 19.50, 14.25, 14.21, 10.54, 10.24.

^{19}F NMR (471 MHz, CDCl$_3$, ppm): δ = −73.38.

HRMS (ESI-TOF) for $C_{16}H_{21}NO_5F_3^+$ $[M+H]^+$: calculated = 364.1366, observed = 364.1373.

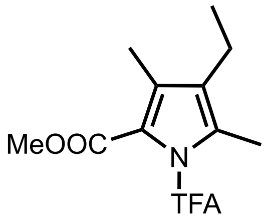

Methyl 4-ethyl-3,5-dimethyl-1-(2,2,2-trifluoroacetyl)-1H-pyrrole-2-carboxylate (1c-TFA)

Using methyl 4-ethyl-3,5-dimethyl-1*H*-pyrrole-2-carboxylate (181 mg, 1 mmol), **1c-TFA** was obtained as a colorless oil in a 62% yield (172 mg, 0.62 mmol).

^{1}H NMR (500MHz, CDCl$_3$, ppm): δ = 3.84 (s, 3H), 2.39 (q, J = 7.6 Hz, 2H), 2.27 (s, 3H), 2.18 (s, 3H), 1.06 (t, J = 7.6 Hz, 3H).

^{13}C NMR (126 MHz, CDCl$_3$, ppm): δ = 161.77, 161.65, 161.32, 132.34, 127.55, 120.59, 116.44, 114.16, 52.00, 17.33, 14.84, 10.49, 10.29.

^{19}F NMR (471 MHz, CDCl$_3$, ppm): δ = −73.25.

Owing to the unstable nature of this molecule, HRMS could not be performed.

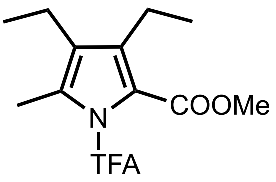

Methyl 3,4-diethyl-5-methyl-1-(2,2,2-trifluoroacetyl)-1*H*-pyrrole-2-carboxylate (1d-TFA)

Using methyl 3,4-diethyl-5-methyl-1*H*-pyrrole-2-carboxylate (195 mg, 1 mmol), **1d-TFA** was obtained as a colorless oil in 48% yield (139 mg, 0.48 mmol).

^{1}H NMR (500MHz, $CDCl_3$, ppm): δ = 3.84 (s, 3H), 2.70 (q, J = 7.6 Hz, 2H), 2.40 (q, J = 7.6 Hz, 2H), 2.18 (s, 3H), 1.15 (t, J = 7.5 Hz, 3H), 1.09 (t, J = 7.6 Hz, 3H).

^{13}C NMR (126 MHz, $CDCl_3$, ppm): δ = 161.60, 161.39, 161.27, 138.51, 132.54, 126.77, 119.80, 116.30, 114.01, 51.89, 18.16, 17.00, 15.36, 15.32, 10.20.

^{19}F NMR (471 MHz, $CDCl_3$, ppm): δ = −73.39.

Owing to the unstable nature of this molecule, HRMS could not be performed.

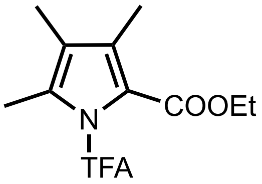

Ethyl 3,4,5-trimethyl-1-(2,2,2-trifluoroacetyl)-1*H*-pyrrole-2-carboxylate (1e-TFA)

Using ethyl 3,4,5-trimethyl-1*H*-pyrrole-2-carboxylate (181 mg, 1 mmol), **1e-TFA** was obtained as a colorless oil in 45% yield (125 mg, 0.45 mmol).

^{1}H NMR (500MHz, $CDCl_3$, ppm): δ = 4.32 (q, J = 7.1 Hz, 2H), 2.25 (s, 3H), 2.16 (s, 3H), 1.93 (s, 3H), 1.34 (t, J = 7.1 Hz, 3H).

^{13}C NMR (126 MHz, $CDCl_3$, ppm): δ = 161.69, 132.44, 132.40, 121.15, 120.79, 113.89, 61.41, 14.33, 10.76, 10.38, 9.03.

^{19}F NMR (471 MHz, $CDCl_3$, ppm): δ = −73.24.

Owing to the unstable nature of this molecule, HRMS could not be performed.

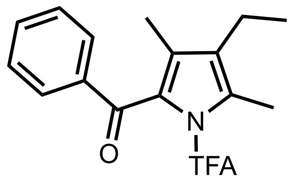

1-(2-Benzoyl-4-ethyl-3,5-dimethyl-1H-pyrrol-1-yl)-2,2,2-trifluoroethan-1-one (1f-TFA)

Using (4-ethyl-3,5-dimethyl-1H-pyrrol-2-yl)(phenyl)methanone (227 mg, 1 mmol), **1f-TFA** was obtained as a light-yellow oil in a 32% yield (104 mg, 0.32 mmol).

^{1}H NMR (500MHz, $CDCl_3$, ppm): δ = 7.72–7.68 (m, 2H), 7.60–7.55 (m, 1H), 7.50–7.46 (m, 2H), 2.40 (q, J = 7.6 Hz, 2H), 2.26 (s, 3H), 1.82 (s, 3H), 1.07 (t, J = 7.6 Hz, 3H).

^{13}C NMR (126 MHz, $CDCl_3$, ppm): δ = 186.85, 160.93, 160.60, 137.77, 135.60, 132.49, 131.81, 131.48, 131.02, 128.94, 128.63, 128.41, 128.37, 116.48, 114.19, 17.34, 14.76, 11.87, 10.28.

^{19}F NMR (471 MHz, $CDCl_3$, ppm): δ = –73.20.

Owing to the unstable nature of this molecule, HRMS could not be performed.

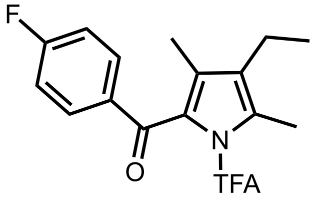

1-(3-ethyl-5-(4-fluorobenzoyl)-2,4-dimethyl-1H-pyrrol-1-yl)-2,2,2-trifluoroethan-1-one (1g-TFA)

Using (4-ethyl-3,5-dimethyl-1H-pyrrol-2-yl)(4-fluorophenyl)methanone (245 mg, 1 mmol), **1g-TFA** was obtained as a light-yellow oil in a 35% yield (119 mg, 0.35 mmol).

^{1}H NMR (500MHz, $CDCl_3$, ppm): δ = 7.78–7.71 (m, 2H), 7.17 (t, J = 8.6 Hz, 2H), 2.40 (q, J = 7.6 Hz, 2H), 2.26 (s, 3H), 1.84 (s, 3H), 1.07 (t, J = 7.6 Hz, 3H).

^{13}C NMR (126 MHz, $CDCl_3$, ppm): δ = 185.39, 166.45, 164.43, 160.80, 160.47, 135.67, 133.95, 133.93, 131.59, 131.52, 131.30, 130.81, 128.62, 116.40, 115.93, 115.76, 115.51, 114.10, 77.37, 77.12, 76.86, 17.31, 14.72, 11.94, 10.29.

^{19}F NMR (471 MHz, $CDCl_3$, ppm): δ = –73.11, –105.77.

Owing to the unstable nature of this molecule, HRMS could not be performed.

3.4.2 Preparation of 2,2,2-trifluoro-1-(2,3,4,5-tetraphenyl-1*H*-pyrrol-1-yl) ethan-1-one (1j-TFA)

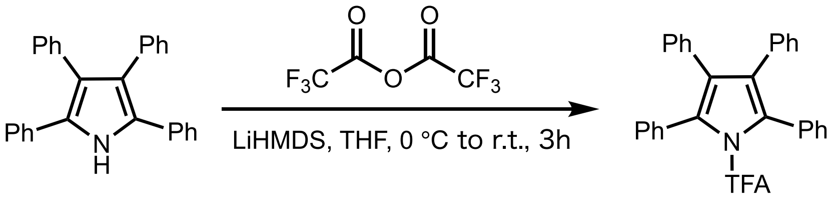

In a 50 mL round-bottomed flask, 2,3,4,5-tetraphenyl-1*H*-pyrrole (1 mmol) and lithium bis(trimethylsilyl)amide (approximately 26% in tetrahydrofuran (THF), approximately 1.3mol/L) (769 µL, 1.0 equiv.) were combined in dry THF (10 mL) at 0 °C under a nitrogen atmosphere and stirred for 15 min. Subsequently, trifluoroacetic anhydride (210 µL, 1.5 mmol, 1.5 equiv.) was added dropwise, and the reaction mixture was allowed to warm to room temperature. After stirring for 3 h, the reaction mixture was carefully poured into water to quench it, and the aqueous phase was extracted with EtOAc three times. The combined organic layer was washed with water twice, brine once, and dried over Na_2SO_4. Following filtration, the solution was concentrated under reduced pressure. The obtained crude material was purified by silica gel column chromatography using hexane/ethyl acetate (100/0 to 97/3) to yield a spectroscopically pure product (**1j-TFA**) in 52% yield (243 mg, 0.52 mmol) as a yellow solid.

^{1}H NMR (500 MHz, $CDCl_3$, ppm): δ = 7.33–7.28 (m, 6H), 7.21–7.17 (m, 4H), 7.13–7.05 (m, 6H), 6.91–6.88 (m, 4H).

^{13}C NMR (126 MHz, $CDCl_3$, ppm): δ = 133.19, 131.77, 130.77, 130.73, 130.51, 128.52, 128.38, 127.88, 127.09, 126.70.

^{19}F NMR (471 MHz, $CDCl_3$, ppm): δ = –70.69.

HRMS (ESI-TOF) for $C_{30}H_{20}NOF_3^+$ $[M+H]^+$: calculated = 468.1570, observed = 468.1566.

3.5. Synthesis of 2-aroyl-3–5-dimethyl-4-ethyl-1*H*-pyrroles (1f)

2-benzoyl-3,5-dimethyl-4-ethyl-1*H*-pyrrole (**1f**) was synthesized according to the reported procedure.[9]

3.6. Preparation of 2-(4-fluorobenzoyl)-3–5-dimethyl-4-ethyl-1H-pyrrole (1g)

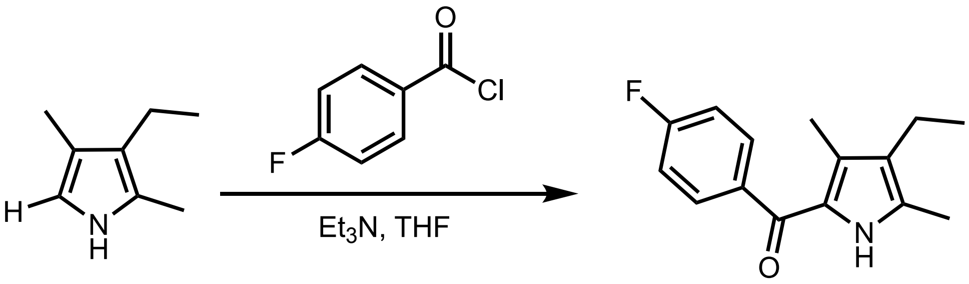

To a solution of kryptopyrrole (2.70 mL, 20.0 mmol) and Et_3N (2.83 mL, 20.4 mmol) in THF (40 mL) at room temperature, 4-fluorobenzoyl chloride (2.37 mL, 20.4 mmol) was added dropwise over few minutes. The mixture was heated to reflux for 2.5 h and subsequently allowed to cool to room temperature. The resulting triethylamine hydrochloride precipitate was removed by filtration through Celite and washed with THF (70 mL). The volatiles were removed from the combined filtrate under reduced pressure, and the residue was recrystallized from MeOH to afford 2-(4-fluorobenzoyl)-3–5-dimethyl-4-ethyl-1H-pyrrole (**1g**) as light-blue solids in 64 % yield.

^{1}H NMR (500 MHz, $CDCl_3$, ppm): δ = 8.81 (s, 1H), 7.69–7.61 (m, 2H), 7.16–7.07 (m, 2H), 2.38 (q, *J* = 7.6 Hz, 2H), 2.25 (s, 3H), 1.91 (s, 3H), 1.05 (t, *J* = 7.6 Hz, 3H).

^{13}C NMR (126 MHz, $CDCl_3$, ppm): δ = 184.23, 165.45, 163.45, 136.69, 136.67, 133.57, 130.87, 130.80, 128.29, 126.90, 125.49, 115.44, 115.27, 17.35, 15.22, 11.95, 11.59.

^{19}F NMR (471 MHz, $CDCl_3$, ppm): δ = –108.80

HRMS (ESI-TOF) for $C_{15}H_{17}NOF^+$ $[M+H]^+$: calculated = 246.1289, observed = 246.1296.

3.7. Preparation of 5-Ethyl-1,1-difluoro-2-mesityl-4,6-dimethyl-1*H*-pyrrolo[1,2-c][1,3,2]diazaboryl-2-ium-1-uide (BF_2 complex of 2-iminopyrrole) (1h)

5-Ethyl-1,1-difluoro-2-mesityl-4,6-dimethyl-1*H*-pyrrolo[1,2-c][1,3,2]diazaboryl-2-ium-1-uide (**1h**) was synthesized according to the reported procedure.[10]

3.8. Preparation of 2-cyclopropyl-3-(4-fluorophenyl)-1*H*-indole (1q)

3.8.1. Synthesis of *N*-(2-(4-fluorobenzoyl) phenyl)cyclopropanecarboxamide (1q')

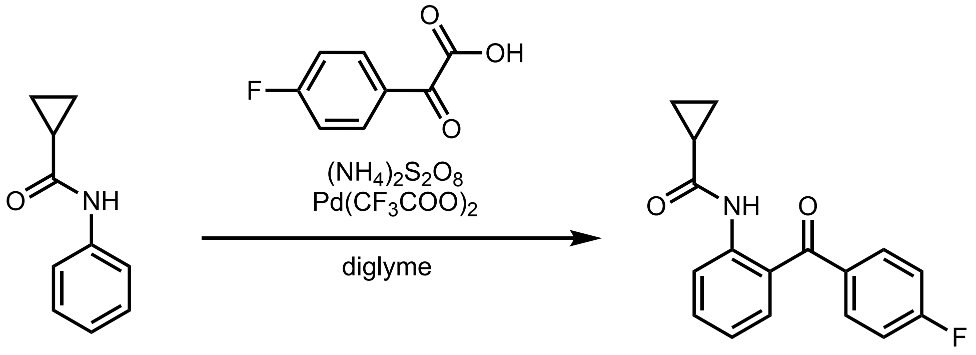

Compound **1q'** was synthesized following the reported procedure.[11]

The benzamide **1q'** (2.42 g, 15.0 mmol), 2-(4-Fluorophenyl)-2-oxoacetic acid[12] (5.04 g, 30.0 mmol), $(NH_4)_2S_2O_8$ (4.11 g, 18.0 mmol), $Pd(CF_3COO)_2$ (499 mg, 1.50 mmol), and diglyme (100 mL) were combined and stirred at room temperature for 48 h under a N_2 atmosphere. After dilution with acetone (200 mL), the mixture was filtered through a Celite pad, washed with acetone (100 mL), and subsequently evaporated under reduced pressure. The crude product was isolated by column chromatography on silica gel (diameter, 6.0 cm; height, 10 cm) using EtOAc/hexane (1:9, v/v) as the solvent (R_f= 0.23), resulting in the formation of compound **1q'** as a white solid in 67% yield.

^{1}H NMR (500 MHz, $CDCl_3$, ppm): δ = 10.92 (s, 1H), 8.71–8.53 (m, 1H), 7.81–7.71 (m, 2H), 7.59–7.49 (m, 2H), 7.21–7.14 (m, 2H), 7.10–7.03 (m, 1H), 1.66–1.60 (m, 1H), 1.11–1.07 (m, 2H), 0.90–0.85 (m, 2H).

^{13}C NMR (126 MHz, $CDCl_3$, ppm): δ = 198.13, 172.86, 166.44, 164.42, 140.65, 134.95, 134.93, 134.37, 133.19, 132.69, 132.62, 123.10, 121.93, 121.81, 115.69, 115.52, 16.58, 8.39.

^{19}F NMR (471 MHz, $CDCl_3$, ppm): δ = −105.48.

HRMS (ESI-TOF) for $C_{17}H_{15}NO_2F^+$ $[M+H]^+$ 284.1081: calculated = 284.1081, observed = 284.1079.

3.8.2. Synthesis of 2-cyclopropyl-3-(4-fluorophenyl)-1*H*-indole (1q)

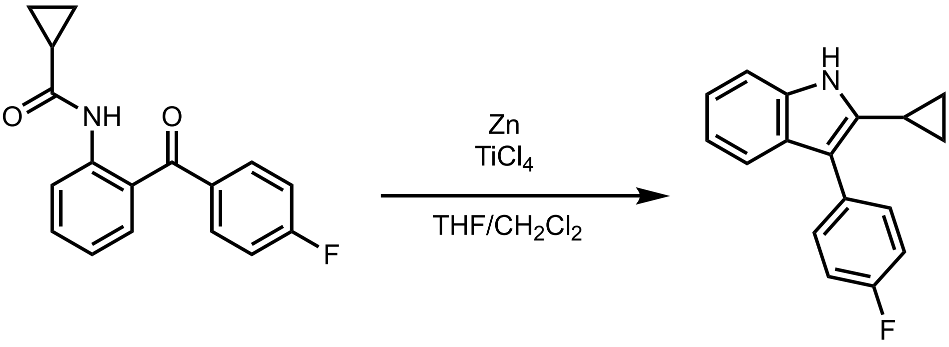

2s **1j**

To the mixture of **1q'** (2.76 g, 9.74 mmol), zinc powder (2.55 g, 39.0 mmol), and dry THF (85 mL), CH_2Cl_2 solution of $TiCl_4$ (20.4 mL, 1.00 mol/L) was added at 0 °C under a N_2 atmosphere. After stirring for 1.5 h at 65 °C, the reaction mixture was cooled to room temperature. The mixture was filtered through a Celite pad and washed with CH_2Cl_2 (200 mL). The filtrate was washed with 1 M HCl aqueous solution (250 mL × 2), and the organic layer was dried over anhydrous sodium sulfate. After filtration, the solvent was concentrated under reduced pressure. The crude product was separated by a silica gel column (diameter, 4.0 cm; height, 11 cm) using EtOAc/hexane (1:9, v/v) as the eluent (R_f = 0.27) to yield indole **1q** as white solids in 87% yield.

^{1}H NMR (500 MHz, $CDCl_3$, ppm): δ = 7.78 (s, 1H), 7.63–7.56 (m, 3H), 7.33–7.30 (m, 1H), 7.19–7.10 (m, 4H), 2.22–2.15 (m, 1H), 1.02–0.97 (m, 2H), 0.75–0.71 (m, 2H).

^{13}C NMR (126 MHz, $CDCl_3$, ppm): δ = 162.33, 160.39, 136.43, 134.81, 131.43, 131.40, 131.08, 131.02, 128.06, 121.83, 120.21, 118.53, 115.39, 115.23, 114.28, 110.63, 8.15, 7.60.

^{19}F NMR (471 MHz, $CDCl_3$, ppm): δ = −117.04.

HRMS (ESI-TOF) for $C_{17}H_{15}NF^+$ $[M+H]^+$: calculated = 252.1183, observed = 252.1187.

3.9. *N*-H-pyridinium salt

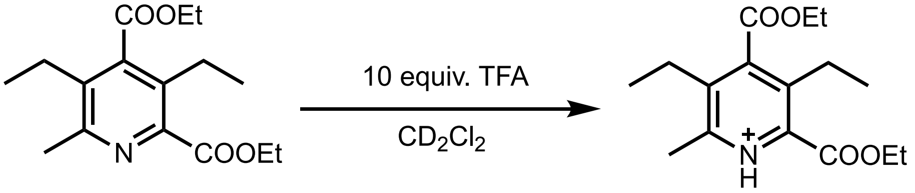

In a 5 mL round-bottomed flask, diethyl 3,5-diethyl-6-methylpyridine-2,4-dicarboxylate **(2ad)** (14.7 mg, 0.05 mmol) was added, followed by the addition of 10 equiv. of trifluoroacetic acid (TFA) (38 μL, 0.5 mmol) were added to CD_2Cl_2 (1 mL). The formation of the desired product was confirmed by ^{1}H NMR and HRMS (ESI-TOF).

^{1}H NMR (500 MHz, CD_2Cl_2, ppm): δ = 4.49 (q, *J* = 7.1 Hz, 2H), 4.49 (q, *J* = 7.1 Hz, 2H), 2.95 (q, *J* = 7.5 Hz, 2H), 2.81 (s, 3H), 2.70 (q, *J* = 7.6 Hz, 2H), 1.36 (t, *J* = 7.2 Hz, 3H), 1.35 (t, *J* = 7.1 Hz, 4H), 1.19 (t, *J* = 7.1 Hz, 3H), 1.13 (t, *J* = 7.1 Hz, 3H).

HRMS (ESI-TOF) for $C_{16}H_{24}NO_4^+$ $[M+H]^+$: calculated = 294.1700, observed = 294.1695.

3.10. Hydrolysis of diethyl 3,5-diethyl-6-methylpyridine-2,4-dicarboxylate (2ad)

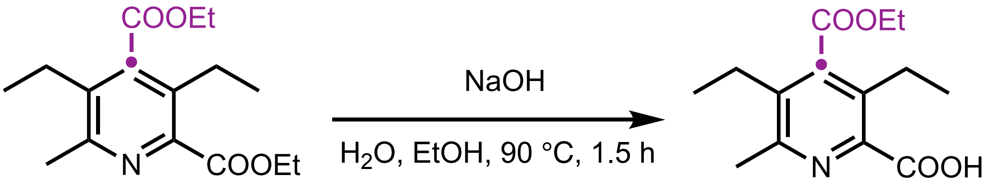

2t

In a 5 mL round-bottomed flask, 4 M (1.85 mL) diethyl 3,5-diethyl-6-methylpyridine-2,4-dicarboxylate (**2ad**) (146.5 mg, 0.5 mmol) and EtOH (2 mL) were added at 90 °C under air. The mixture was stirred for 1.5 h and subsequently concentrated under reduced pressure. The reaction mixture was then carefully poured into 1 M HCl (aq.), and the aqueous phase was extracted with EtOAc. The combined organic layer was washed with brine once and dried over Na_2SO_4. After filtration, the solvent was removed under reduced pressure. The resulting crude product was purified by silica gel column chromatography using a chloroform/methanol gradient (100/0 to 90/10) to yield a spectroscopically pure product (**2t**) in 89% (117.9 mg, 0.445 mmol) as colorless oil.

^{1}H NMR (500 MHz, $CDCl_3$, ppm): δ = 4.46 (q, J = 7.2 Hz, 2H), 3.06 (q, J = 7.4 Hz, 2H), 2.62 (q, J = 7.6 Hz, 2H), 2.58 (s, 3H), 1.41 (t, J = 7.2 Hz, 3H), 1.23 (t, J = 7.4 Hz, 2H), 1.20 (t, J = 7.6 Hz, 3H).

^{13}C NMR (126 MHz, $CDCl_3$, ppm): δ = 167.68, 163.60, 153.72, 145.73, 140.04, 138.64, 137.13, 62.10, 24.28, 23.67, 21.59, 15.37, 14.32, 13.97.

HRMS (ESI-TOF) for $C_{14}H_{20}NO_4^+$ $[M+H]^+$: calculated = 266.1387, observed =266.1383.

3.11. Synthesis of NHPI esters (2u)

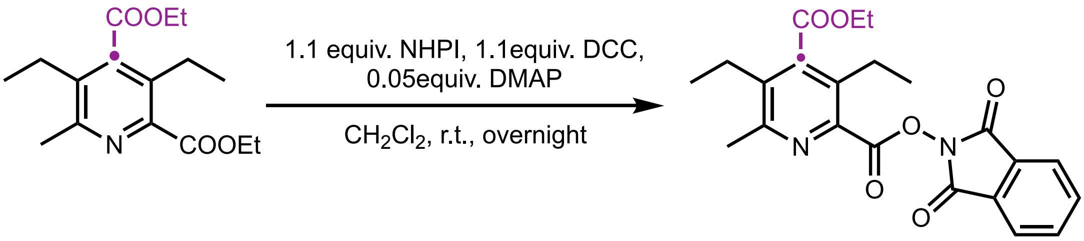

2t **2u**

In a 5 mL round-bottomed flask, *N*-hydroxyphthalimide (33.3 mg, 1.1 equiv.), DMAP (1.0 mg, 0.05 equiv.), and 4-(ethoxycarbonyl)-3,5-diethyl-6-methylpicolinic acid (**2t**) (49.3 mg, 0.18 mmol) were combined. Dichloromethane (CH_2Cl_2) (0.1 M) was added, followed by the addition of *N*,*N'*-dicyclohexylcarbodiimide (42 mg, 1.1 equiv.). The reaction mixture was stirred at room temperature under a N_2 atmosphere. After being stirred for overnight, the reaction mixture was filtered. Then, the solution was concentrated under reduced pressure. The obtained crude material was purified by silica gel column chromatography using hexane/ethyl acetate (100/0 to 80/20) to produce a spectroscopically pure product (**2u**) as light-yellow oil in 86% yield (63.5 mg, 0.155 mmol).

^{1}H NMR (500 MHz, $CDCl_3$, ppm): δ = 7.94–7.87 (m, 2H), 7.82–7.76 (m, 2H), 4.44 (q, *J* = 7.2 Hz, 2H), 2.85 (q, *J* = 7.5 Hz, 2H), 2.64 (s, 3H), 2.63 (q, *J* = 7.5 Hz, 2H), 1.40 (t, *J* = 7.1 Hz, 3H), 1.23 (t, *J* = 7.5 Hz, 3H), 1.20 (t, *J* = 7.5 Hz, 3H).

^{13}C NMR (126 MHz, $CDCl_3$, ppm): δ = 167.57, 162.02, 161.72, 155.95, 143.91, 140.52, 138.01, 136.80, 134.88, 129.15, 124.11, 61.98, 24.40, 23.76, 22.05, 15.50, 14.27, 13.92.

HRMS (ESI-TOF) for $C_{22}H_{23}N_2O_6^+$ $[M+H]^+$: calculated = 411.1550, observed = 411.1551.

3.12. Preparation of dimethyl 4-ethyl-2,6-dimethylpyridine-3,5-dicarboxylate (Addit)

Dimethyl 4-ethyl-2,6-dimethylpyridine-3,5-dicarboxylate (**2r**) was synthesized according to a previously reported procedure.[13]

3.13. Synthesis of methyl 4-ethyl-2,5-dimethyl-1H-pyrrole-3-carboxylate (1s-H)

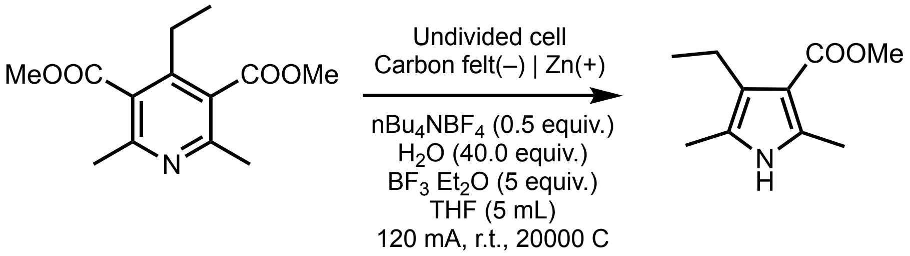

Compound **1s-H** was synthesized following a reported method.[14]

A 500 mL three-necked flask was charged with dimethyl 4-ethyl-2,6-dimethylpyridine-3,5-dicarboxylate (**2r**) (12 mmol), Bu_4NBF_4 (6 mmol), and a magnetic stir bar. The flask was equipped with rubber stoppers, graphite felt cathode, and a zinc plate anode, each connected to a copper wire. The system was evacuated and backfilled with N_2 three times. Subsequently, H_2O (40.0 equiv.), $BF_3{\cdot}Et_2O$ (5 equiv.), and anhydrous THF (150 mL) were added via syringe. The reaction mixture was stirred at room temperature under constant current electrolysis (120 mA). After a total charge of 20000 C had passed, the reaction mixture was extracted with EtOAc. The combined organic layers were washed with brine, dried over Na_2SO_4, filtered, and concentrated under reduced pressure. The resulting crude material was purified by silica gel column chromatography using a hexane/ethyl acetate gradient (100/0 to 90/10), affording **1s** as a spectroscopically pure white solid in 33% yield (717 mg, 3.96 mmol).

^{1}H NMR (500 MHz, $CDCl_3$, ppm): δ = 8.12 (s, 1H), 3.79 (s, 3H), 2.62 (q, J = 7.4 Hz, 2H), 2.44 (s, 3H), 2.12 (s, 3H), 1.09 (t, J = 7.4 Hz, 3H).

^{13}C NMR (126 MHz, $CDCl_3$, ppm): δ = 166.86, 133.98, 122.96, 121.86, 109.66, 50.46, 18.62, 16.00, 14.02, 10.45.

HRMS (ESI-TOF) for $C_{10}H_{16}NO_2^+$ $[M+H]^+$: calculated = 182.1176, observed = 182.1176.

4. Supporting Data

4.1 Reaction condition optimization

4.1.1 Electrode and solvent selection

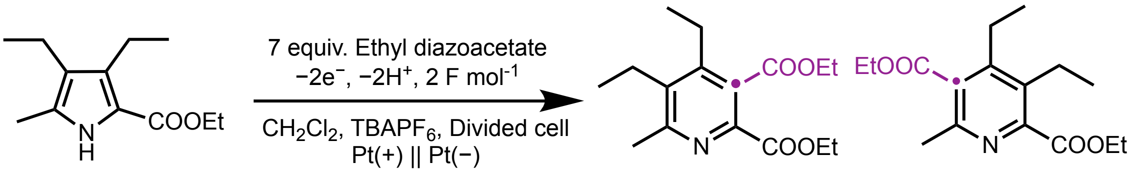

Table S1. Optimization of reaction conditions (electrode and solvent).

Entry	Working electrode	Solvent	Yield(a, b)
1	Pt	CH_2Cl_2	6% (3%, 3%)
2	Glassy carbon	CH_2Cl_2	8% (4%, 4%)
3	Pt mesh	CH_2Cl_2	13% (6%, 7%)
4	Carbon felt	CH_2Cl_2	15% (7%, 8%)
5	Carbon felt	Acetonitrile	N.D.
6	Carbon felt	TFE:CH_2Cl_2 = 1:1	5% (4%, 1%)

4.1.2 Adjustment of additional parameters

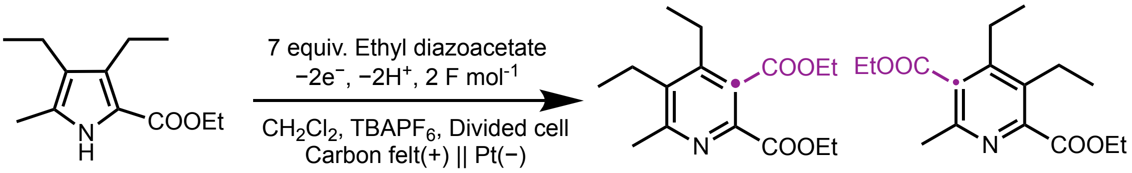

Table S2. Optimization of reaction conditions (other conditions).

Entry	variation from the standard condition	Yield(a, b)
7	TBAOTf	N.D.
8	TEAOTs	N.D.
9	5 equiv. Ethyl diazoacetate	11%(6%, 5%)
10	7 equiv. Ethyl diazoacetate	15%(7%, 8%)
11	9 equiv. Ethyl diazoacetate	14%(7%, 7%)
12	40 °C	11%(8%, 3%)
13	no current	N.D.
14	3 F mol^{-1}	18%(9%, 9%)
15	4 F mol^{-1}	14%(6%, 8%)

4.1.3 Flow reactor conditions

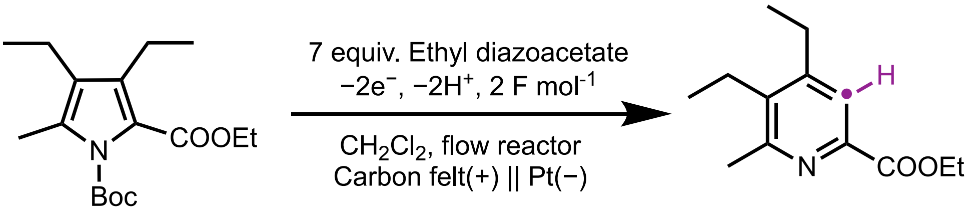

Table S3. Optimization of reaction conditions (flow reactor).

Entry	Current [mA]	Flow rate [mL/h]	Resident time [min]	Supporting electrolyte	Yield
1	5	6.2	9.6	Bu_4NPF_6	34%
2	10	12.4	4.8	Bu_4NPF_6	36%
3	15	16.1	3.2	Bu_4NPF_6	28%
4	10	12.4	4.8	$Bu_4NB(C_6F_5)_4$	44%

4.2 General procedure for the CV experiments

The VSP-3A system (Biologic) was used to conduct CV measurements. All CV measurements were conducted in the three-electrode system equipped with a glassy carbon (GC) disk as the working electrode (φ = 3.0 mm), a Pt plate as the counter electrode (20 mm × 20 mm), and Ag/$AgNO_3$ as the reference electrode.

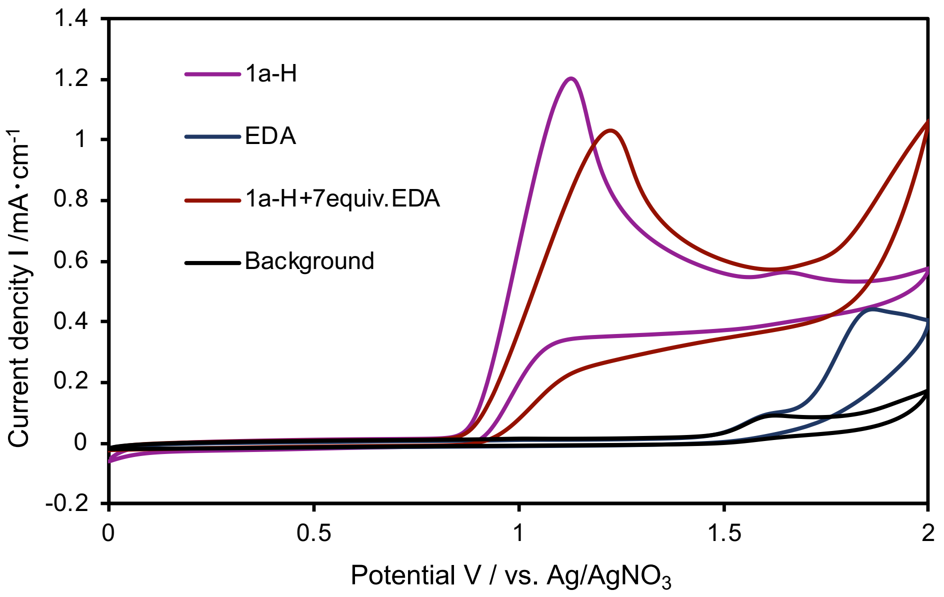

Figure S5. Cyclic voltammograms of **1a-H** and EDA.

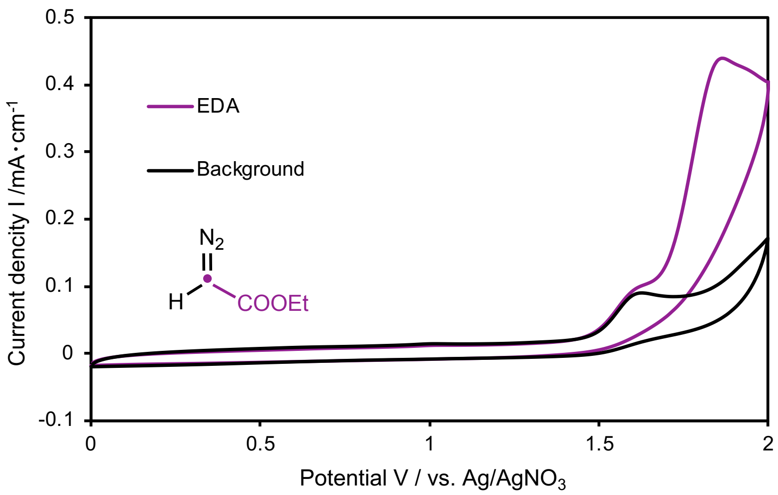

Figure S6. Cyclic voltammograms of EDA.

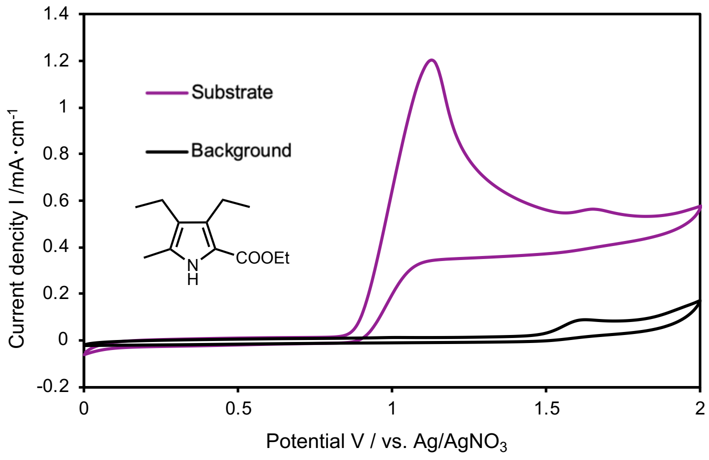

Figure S7. Cyclic voltammograms of **1a-H**.

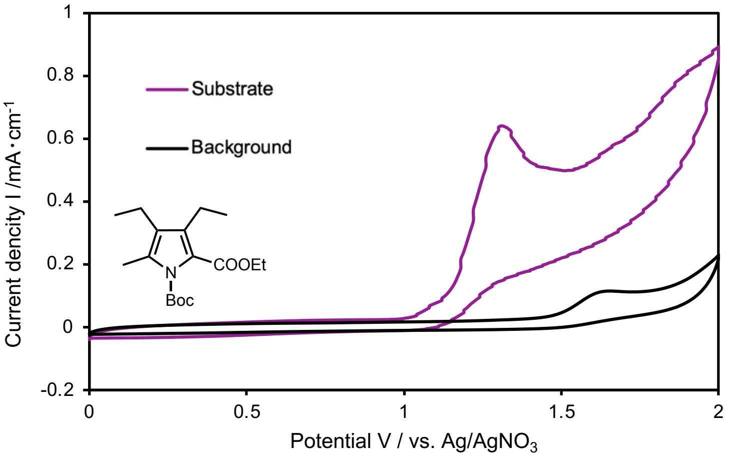

Figure S8. Cyclic voltammograms of **1a-Boc**.

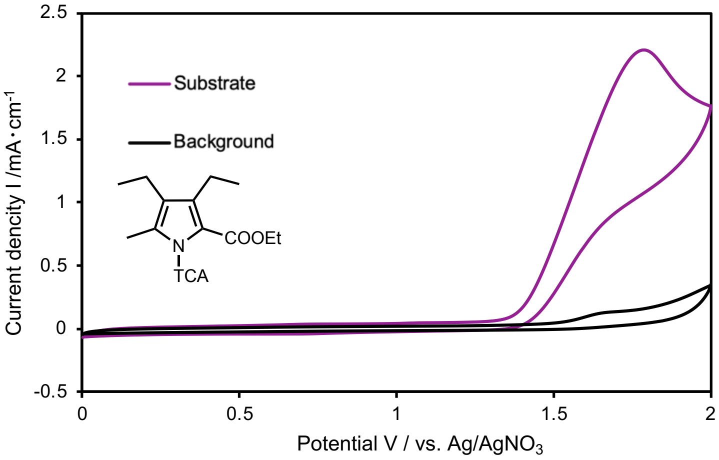

Figure S9. Cyclic voltammograms of **1a-TCA**.

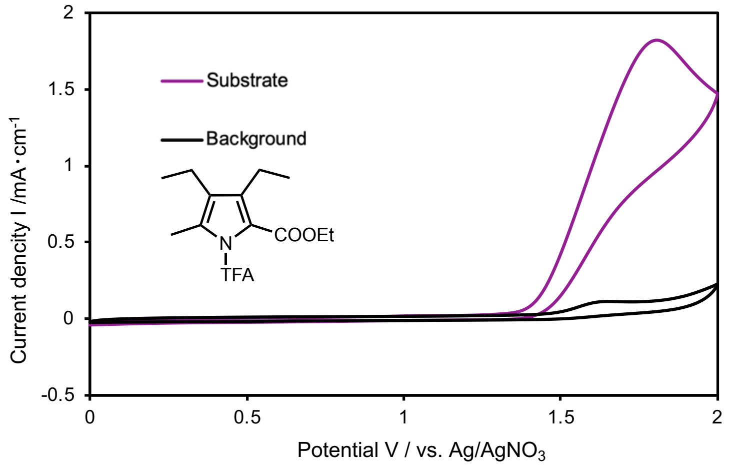

Figure S10. Cyclic voltammograms of **1a-TFA**.

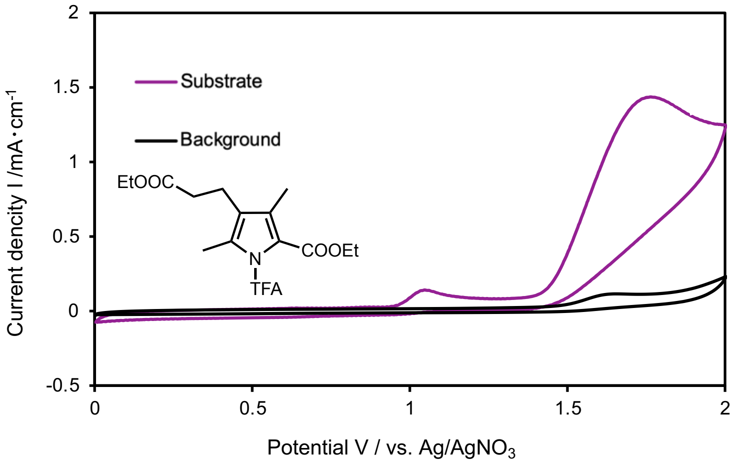

Figure S11. Cyclic voltammograms of **1b-TFA**.

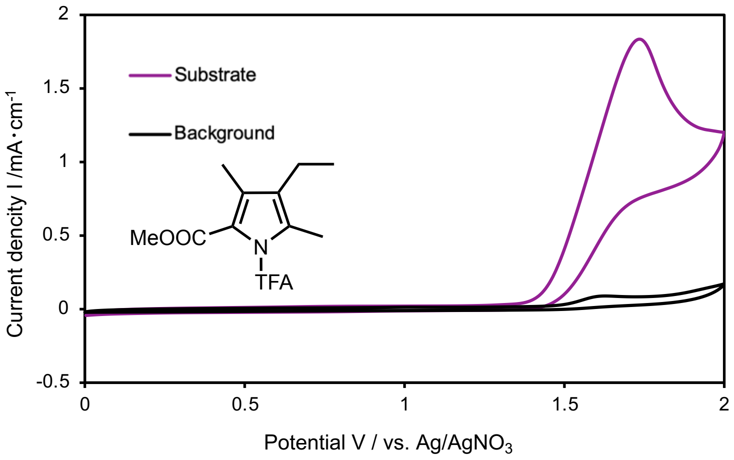

Figure S12. Cyclic voltammograms of **1c-TFA**.

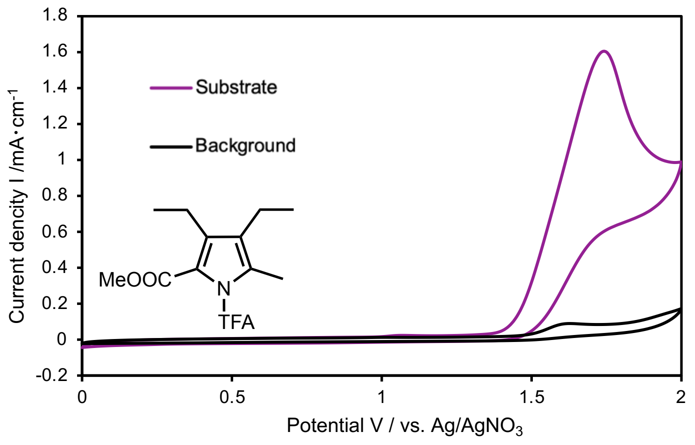

Figure S13. Cyclic voltammograms of **1d-TFA**.

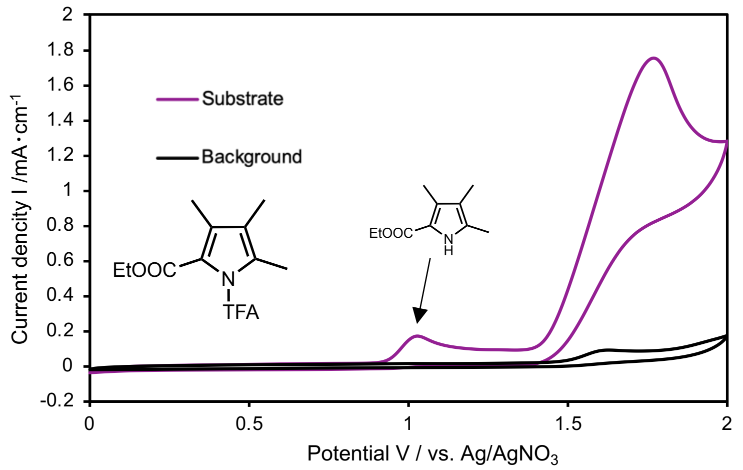

Figure S14. Cyclic voltammograms of **1e-TFA**.

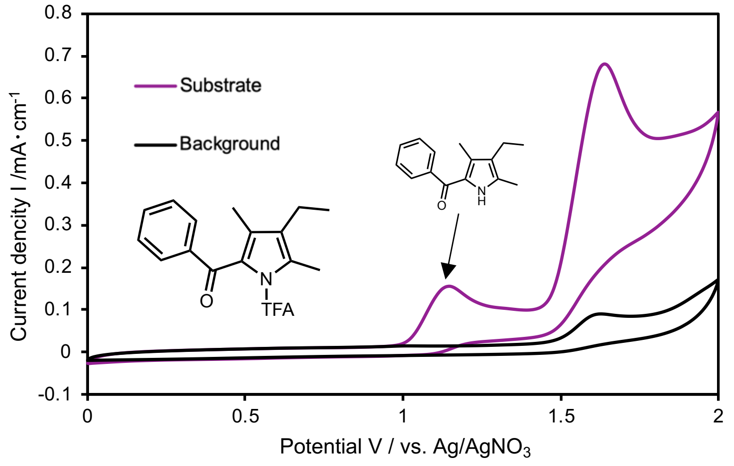

Figure S15. Cyclic voltammograms of **1f-TFA**.

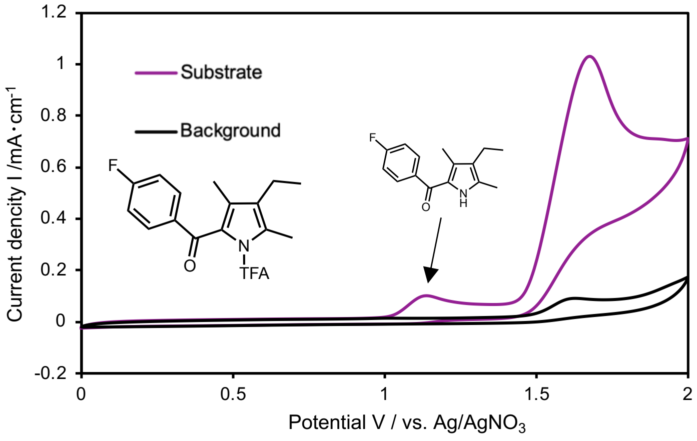

Figure S16. Cyclic voltammograms of **1g-TFA**.

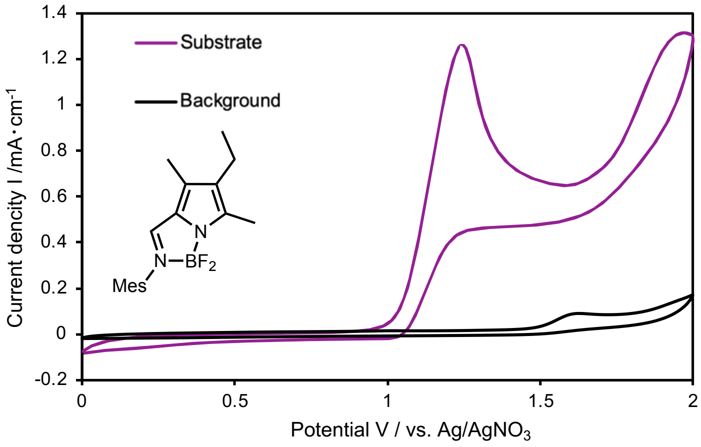

Figure S17. Cyclic voltammograms of **1h -TFA**.

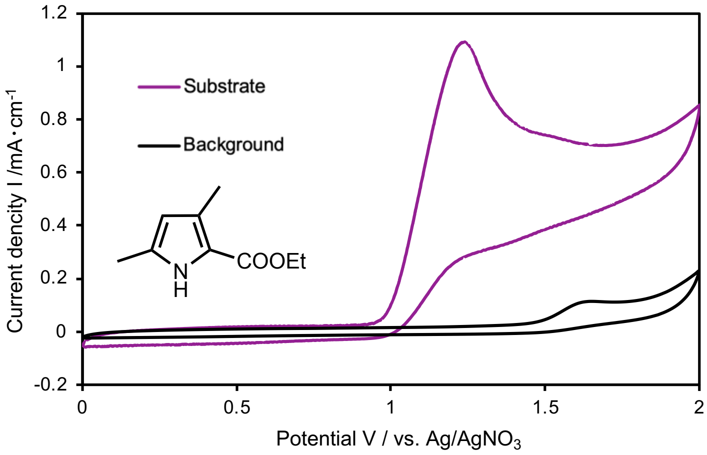

Figure S18. Cyclic voltammograms of **1i-H**.

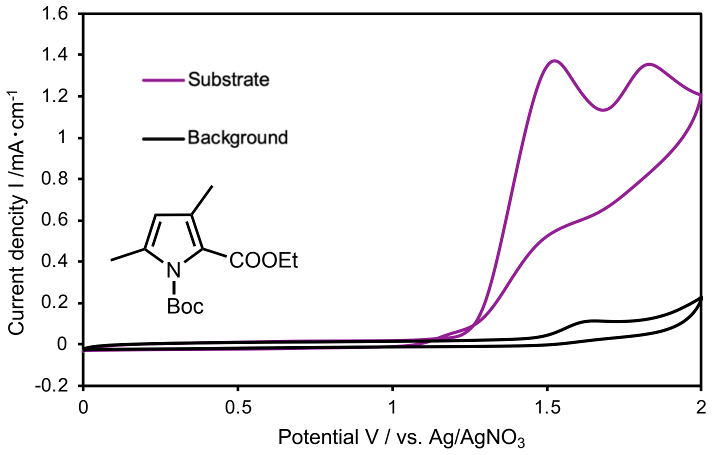

Figure S19. Cyclic voltammograms of **1i-Boc**.

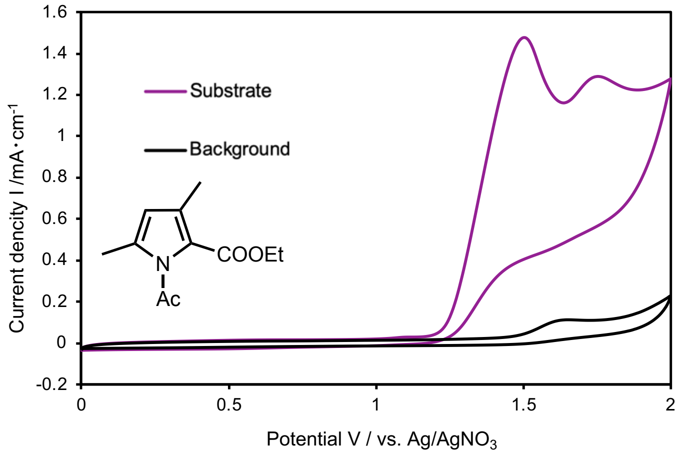

Figure S20. Cyclic voltammograms of **1i-Ac**.

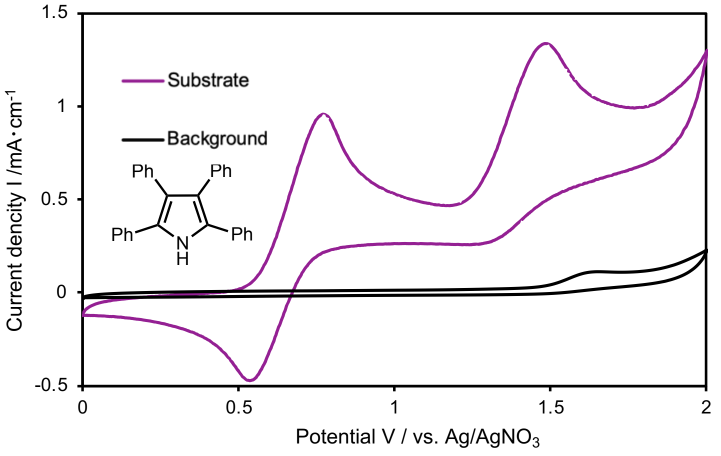

Figure S21. Cyclic voltammograms of **1j-H**.

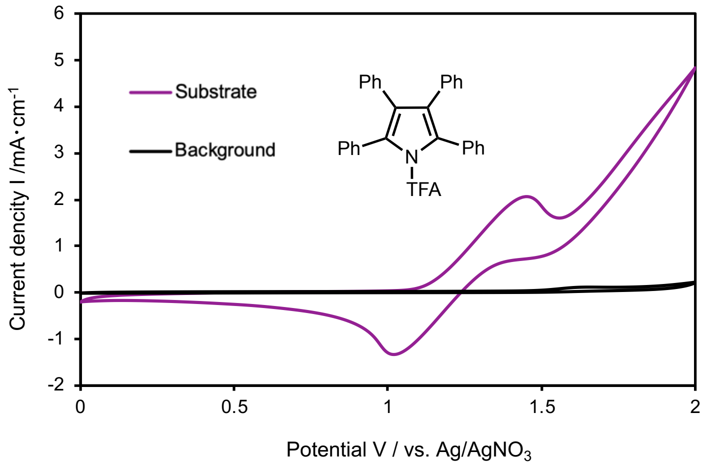

Figure S22. Cyclic voltammograms of **1j-TFA**.

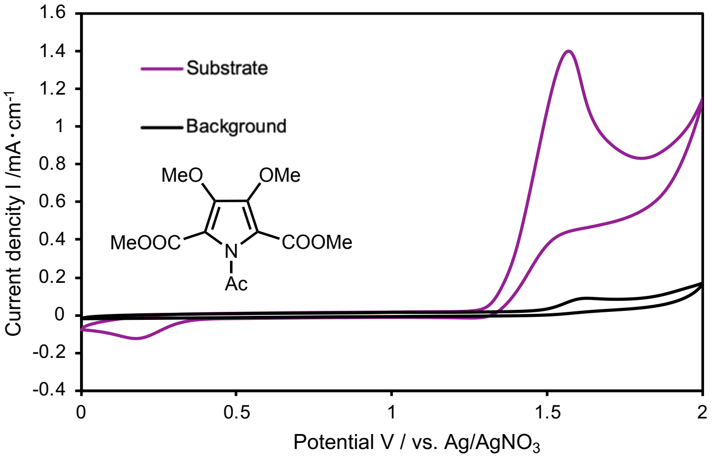

Figure S23. Cyclic voltammograms of **1k-Ac**.

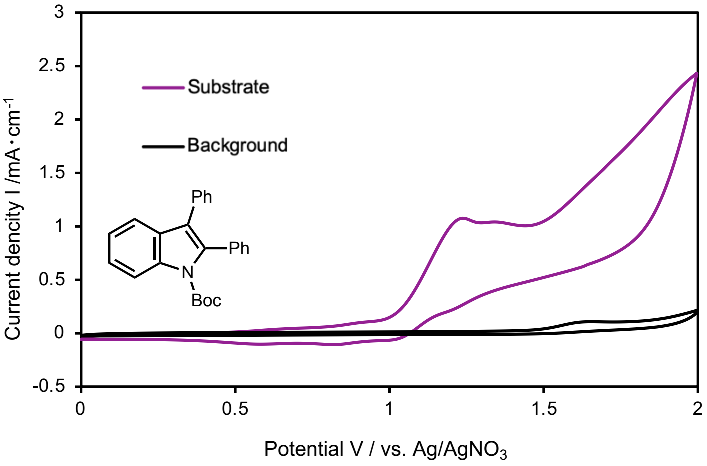

Figure S24. Cyclic voltammograms of **1n-Boc**.

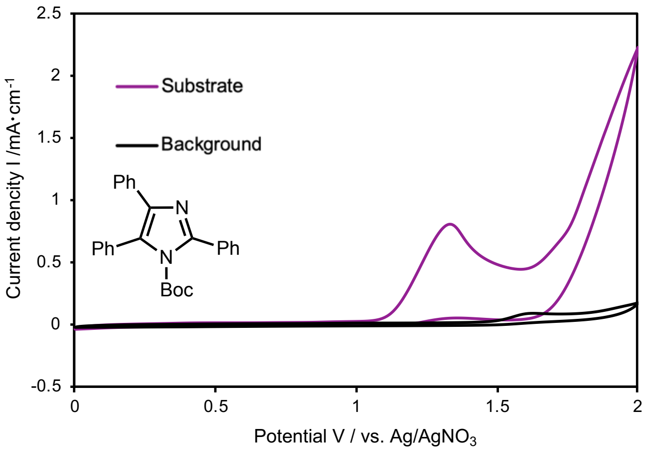

Figure S25. Cyclic voltammograms of **1o-Boc**.

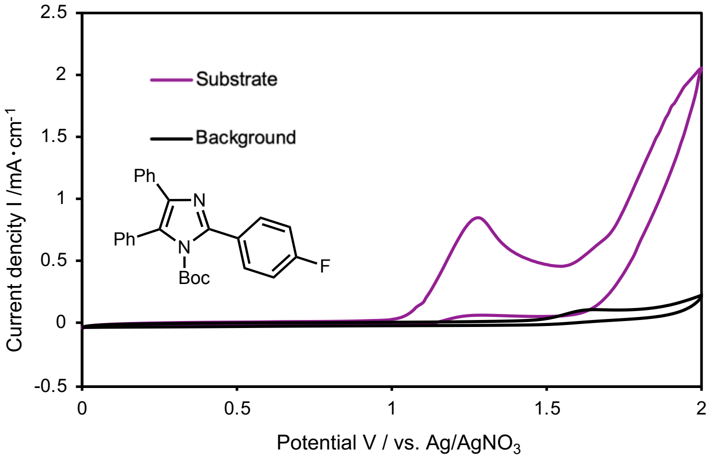

Figure S26. Cyclic voltammograms of **1p-Boc**.

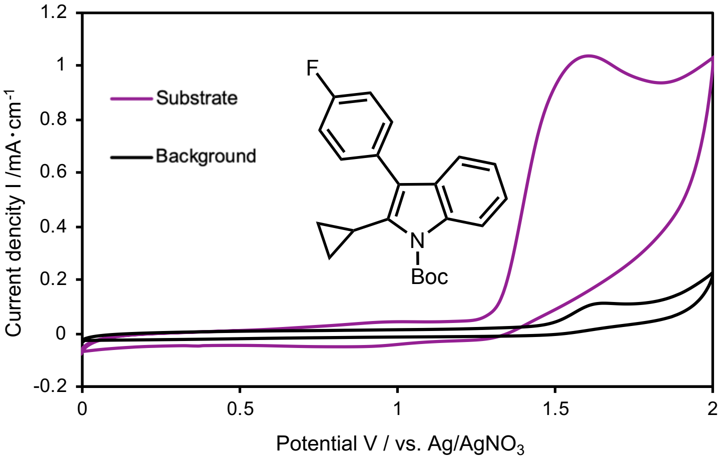

Figure S27. Cyclic voltammograms of **1q-Boc**.

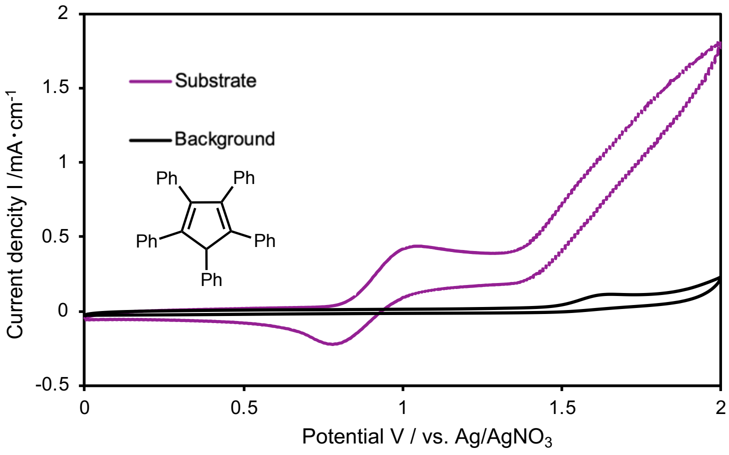

Figure S28. Cyclic voltammograms of **3a**.

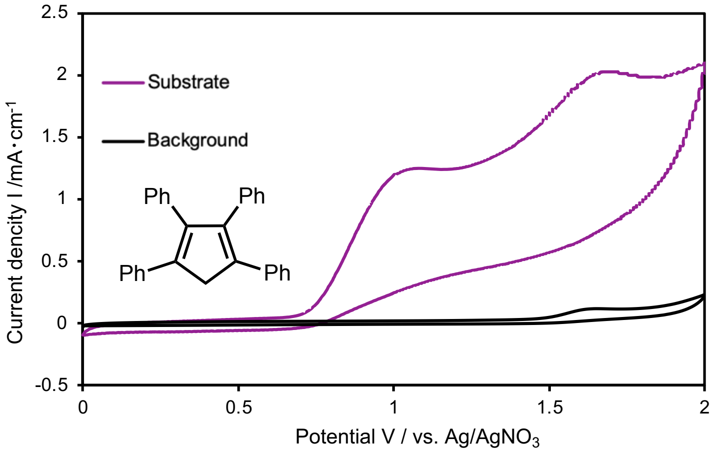

Figure S29. Cyclic voltammograms of **3b**.

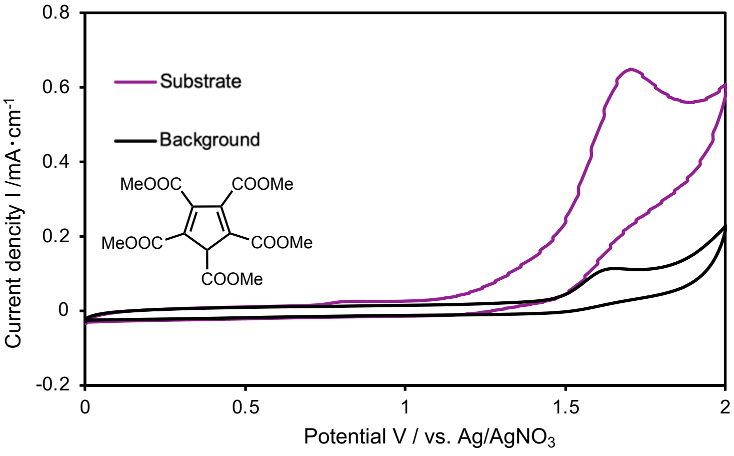

Figure S30. Cyclic voltammograms of **3c**.

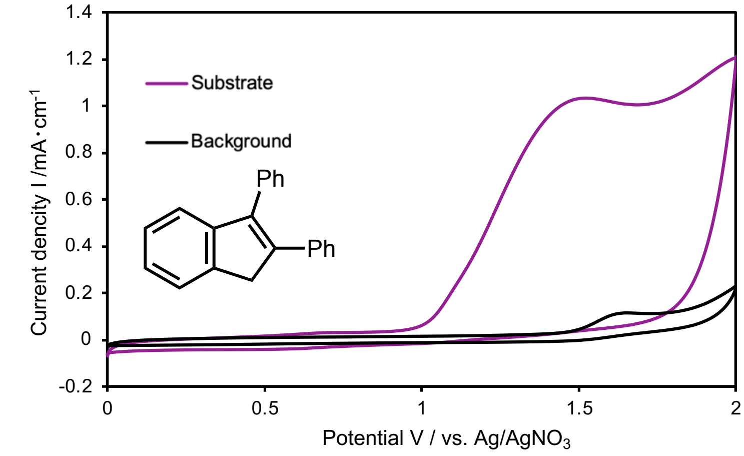

Figure S31. Cyclic voltammograms of **3d**.

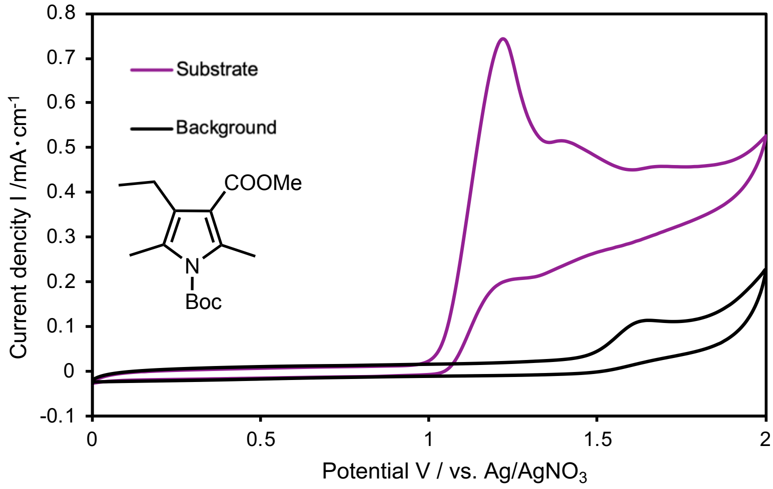

Figure S32. Cyclic voltammograms of **1s-Boc**.

4.3 General procedure for the *in situ* electron paramagnetic resonance experiments

The reaction solution was prepared by dissolving tetraphenylpyrrole (1d-H) (0.10 mmol, 10 mM) in 10 mL of an electrolyte solution consisting of 0.1 M Bu_4NPF_6/CH_2Cl_2. A portion of this solution was transferred to custom-made electrochemical cells (Figure S26), and EPR measurements were performed under constant current electrolysis at 0.01 mA. The EPR spectra were recorded at room temperature on an EPR spectrometer operated at 9484.112 MHz. The typical spectrometer settings were as follows: scan range of 25 mT, center field set to 339.070 mT, time constant of 0.03 s, sweep time of 4 min; modulation frequency of 100.00 kHz, and microwave power of 3.00 mW.

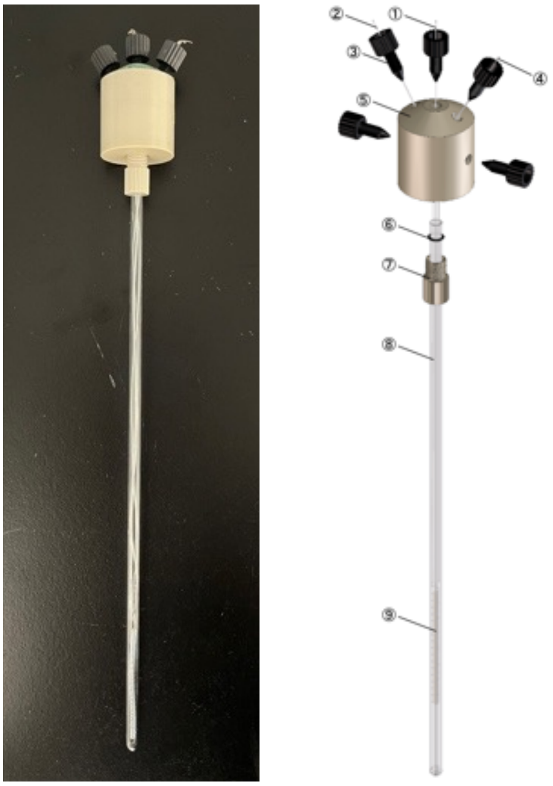

①	Pt wire
②	Pt wire
③	Tube conector
④	Ag wire
⑤	Body of reactor
⑥	O-ring
⑦	Holder
⑧	Glass body
⑨	Separator

Figure S33. Complete structure of custom-made electrochemical cells.

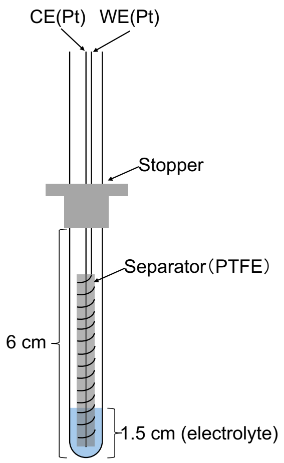

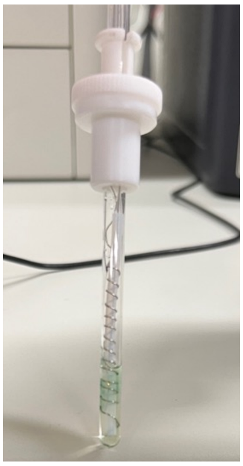

Figure S34. Schematic illustration (left) and post-electrolysis photograph (right) of the custom-designed electrochemical cell.

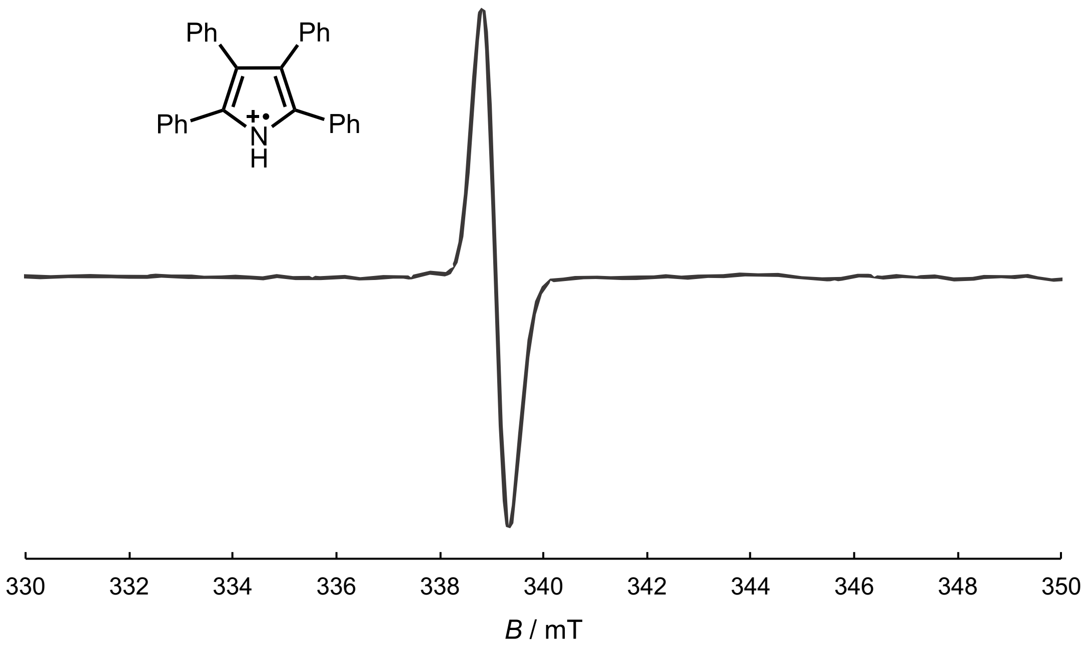

Figure S35. *In situ* EPR spectra of tetraphenylpyrrole (**1d-H**).

4.4 Results of energy calculations

Geometry optimizations and vibrational frequency analyses were performed using Gaussian 16 (Revision C.01). The calculations were based on (un)restricted density functional theory using the CAM-B3LYP functional, incorporating the D3(BJ) dispersion correction. For hydrogen and carbon atoms, the 6-31G(d,p) basis set was used, while the 6-31+G(d,p) basis set was used for nitrogen, oxygen, and fluorine atoms. Thermodynamical corrections were calculated at a temperature of 298.15 K. Single-point energy calculations were performed using ORCA 5.0.3. The DLPNO-R(O)-CCSD method was used with the def2-TZVPP basis set for hydrogen and carbon atoms, and the def2-TZVPPD basis set was used for nitrogen, oxygen, and fluorine atoms. All calculations were performed in the gas phase, with no solvent effects considered.

While the polarizable continuum model (PCM) is widely used to incorporate solvent effects, it is principally suited for homogeneous systems. In contrast, electrolytes are generally inhomogeneous, requiring explicit treatment for both cationic and anionic species, which is impractical for complex chemical reactions. Moreover, the most popular PCM, the conductor-like PCM (C-PCM), is appropriate for solvents with high dielectric constants. In contrast, for solvents with relatively low dielectric constants, the integral equation formalism PCM (IEF-PCM) is typically recommended. As shown in Table S4, the use of C-PCM compromises the prediction accuracy when IEF-PCM is used as a reference. Notably, the inclusion of solvent effects does not qualitatively change the relative order of **II-TS-pro2,3** and **IV-TS-pro3,4**. Therefore, we relied on gas-phase results. It is noteworthy that these results were obtained at the U-CAM-B3LYP-D3(BJ)/6-31(+)G(d,p) level of theory. Although DLPNO-CCSD calculations were necessary to determine the correct selectivity, IEF-PCM is not applicable to energy calculations at this level of theory.

Table S4. $\Delta\Delta G$ (in kcal mol^{-1}) at the U-CAM-B3LYP-D3(BJ)/6-31(+)G(d,p) level of theory.

	Vacuum	C-PCM	IEF-PCM
**II-TS-pro2,3**	0.00	0.00	0.00
**IV-TS-pro3,4**	−2.12	−0.56	−2.22
**II-TS-III**	+0.78	+2.31	[a]
**III-TS-IV**	−0.34	+2.62	+0.91

[a]The structure for this entry could not be optimized, likely due to numerical instability arising from the discretized tesserae used in the PCM model.

4.4.1 Δ$G^‡$ (298.15 K) for the addition reaction of ethyl diazoacetate (in kcal/mol)

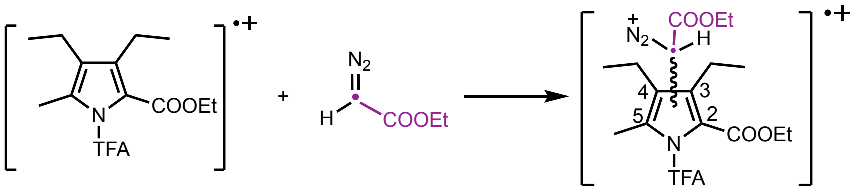

Table S5. Δ$G^‡$ of the addition reaction of ethyl diazoacetate

Addition carbon	Δ$G^‡$ (298.15 K) / kcal/mol
2	5.17
3	15.20
4	15.87
5	7.51

4.4.2 Energy diagram of 1a-H

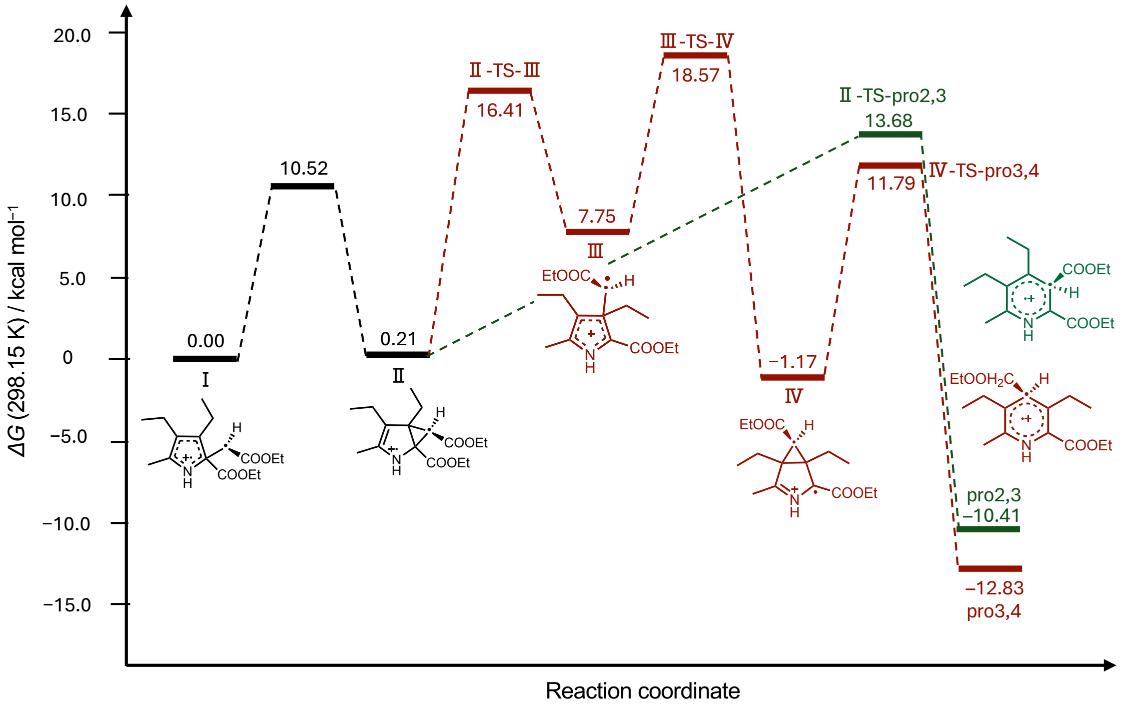

Figure S36. Energy diagram of **1a-H**.

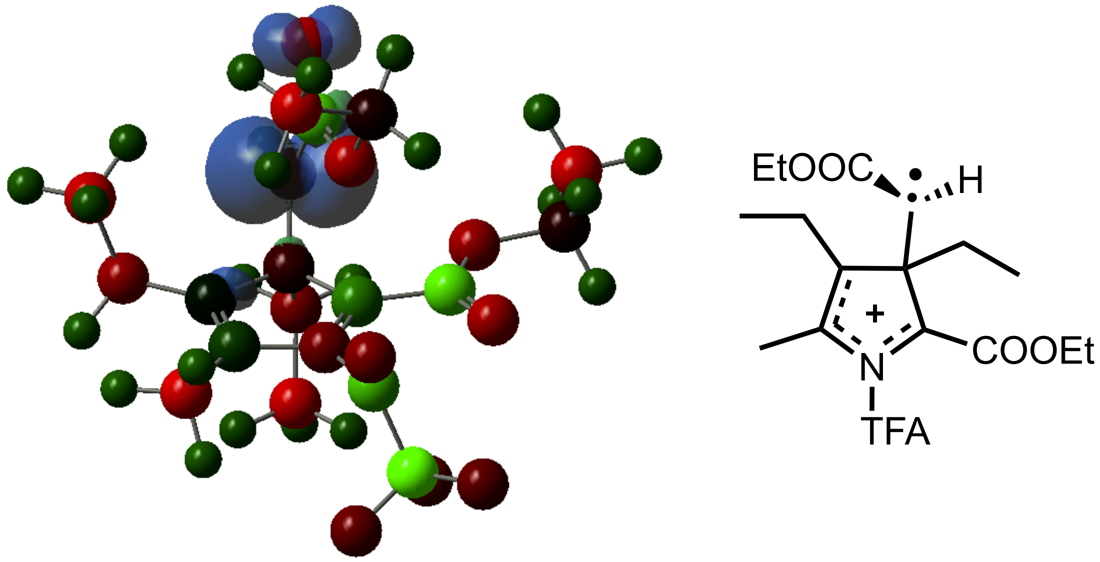

Figure S37. Three-dimensional representation and chemical structure of a distonic radical cation (**1a-TFA III**). (Spin density (isovalue = 0.005) and NBO charge distribution (color range: –1.113 to 1.113)).

4.5. Single-crystal X-ray diffraction

4.5.1 X-ray crystal structure of 2ja

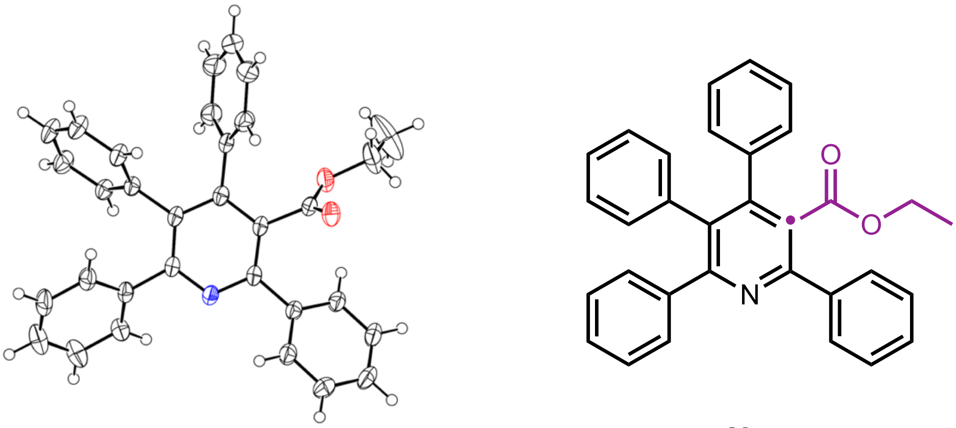

2ja

Figure S38. X-ray crystal structure of **2ja.**

Crystallographic data are available free of charge from the Cambridge Crystallographic Data Centre (https://summary.ccdc.cam.ac.uk/structure-summary-form under deposition number 2388787).

```
Bond precision:   C-C = 0.0040 A          Wavelength=1.54184

Cell:             a=14.1075(2)     b=9.2506(2)        c=18.8106(3)
                  alpha=90         beta=96.021(2)     gamma=90
Temperature:      223 K

                 Calculated                 Reported
Volume           2441.30(7)                 2441.29(7)
Space group      P 21/n                     P 21/n
Hall group       -P 2yn                     -P 2yn
Moiety formula   C32 H25 N O2               C32 H25 N O2
Sum formula      C32 H25 N O2               C32 H25 N O2
Mr               455.53                     455.53
Dx,g cm-3        1.239                      1.239
Z                4                          4
Mu (mm-1)        0.602                      0.602
F000             960.0                      960.0
F000'            962.70
h,k,lmax         17,11,23                   17,11,23
Nref             4962                       4791
Tmin,Tmax                                   0.433,1.000
Tmin'

Correction method= # Reported T Limits: Tmin=0.433 Tmax=1.000
AbsCorr = MULTI-SCAN

Data completeness= 0.966           Theta(max)= 74.058

                                                         wR2(reflections)=
R(reflections)= 0.1202( 3781)                            0.2910( 4791)

S = 1.141                  Npar= 317
```

4.5.2 X-ray crystal structure of 2m

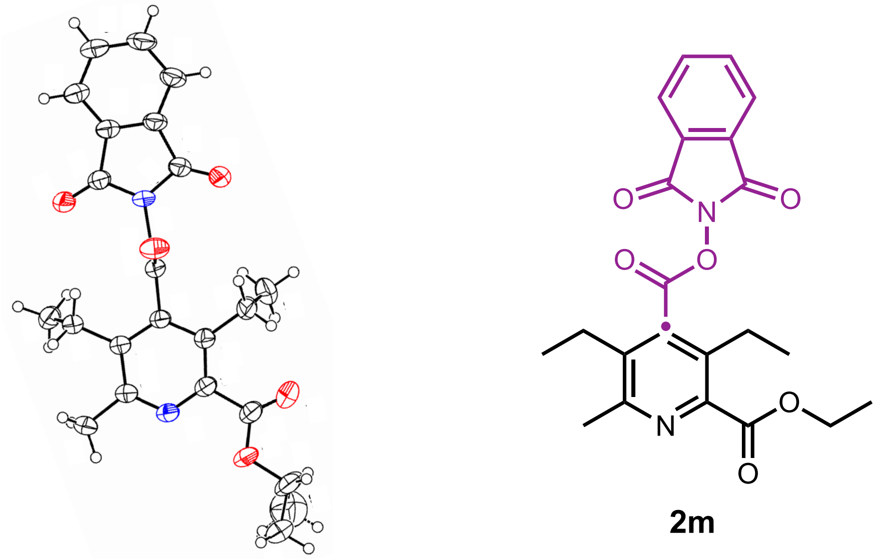

2m

Figure S39. X-ray crystal structure of **2m.**

Crystallographic data are available free of charge from the Cambridge Crystallographic Data Centre (https://summary.ccdc.cam.ac.uk/structure-summary-form under deposition number 2388788).

Bond precision: C-C = 0.0033 A Wavelength=1.54184

Cell:	a=9.1860(5)	b=9.8488(4)	c=11.5628(5)
	alpha=87.118(3)	beta=72.724(5)	gamma=86.675(4)
Temperature:	123 K		

	Calculated	Reported
Volume	996.62(8)	996.62(8)
Space group	P -1	P -1
Hall group	-P 1	-P 1
Moiety formula	C22 H22 N2 O6	C22 H22 N2 O6
Sum formula	C22 H22 N2 O6	C22 H22 N2 O6
Mr	410.42	410.41
Dx,g cm-3	1.368	1.368
Z	2	2
Mu (mm-1)	0.835	0.835
F000	432.0	432.0
F000’	433.45	
h,k,lmax	11,12,14	11,12,14
Nref	4003	3869
Tmin,Tmax	0.777,0.920	0.705,1.000
Tmin’	0.627	

Correction method= # Reported T Limits: Tmin=0.705 Tmax=1.000
AbsCorr = MULTI-SCAN

Data completeness= 0.967 Theta(max)= 73.138

R(reflections)= 0.0682(3297) wR2(reflections)= 0.2052(3869)

S = 1.076 Npar= 279

4.6. Expansion of synthetic utility

Flow electrochemistry

To enhance productivity, we utilized flow electrolysis. Boc-protected substrates were chosen for flow synthesis as they degrade slowly due to the electrogenerated acid (EGA). The use of a divided flow electrolysis cell and $Bu_4NB(C_6F_5)_4/CH_2Cl_2$ electrolyte significantly enhanced yields for **2ac** and **2db** compared to the batch system. This yield enhancement was attributed to the design of the flow cell, which sequentially removes the EGA from the reaction system (Figure S40A).

This flow system was then employed in the synthesis of a pharmaceutically relevant compound (Figure S40B). We successfully synthesized **2q**, a synthetic intermediate of pitavastatin,[15,16] through the ring expansion of indole (**1q**) without using noble metal complexes or strong bases. It is noteworthy that our reaction is compatible with cyclopropane substituents despite the formation of a radical cation intermediate. This compatibility may be due to the delocalization of the radical generated within the π-system, preventing cleavage of the C–C bond in the neighboring cyclopropane ring.

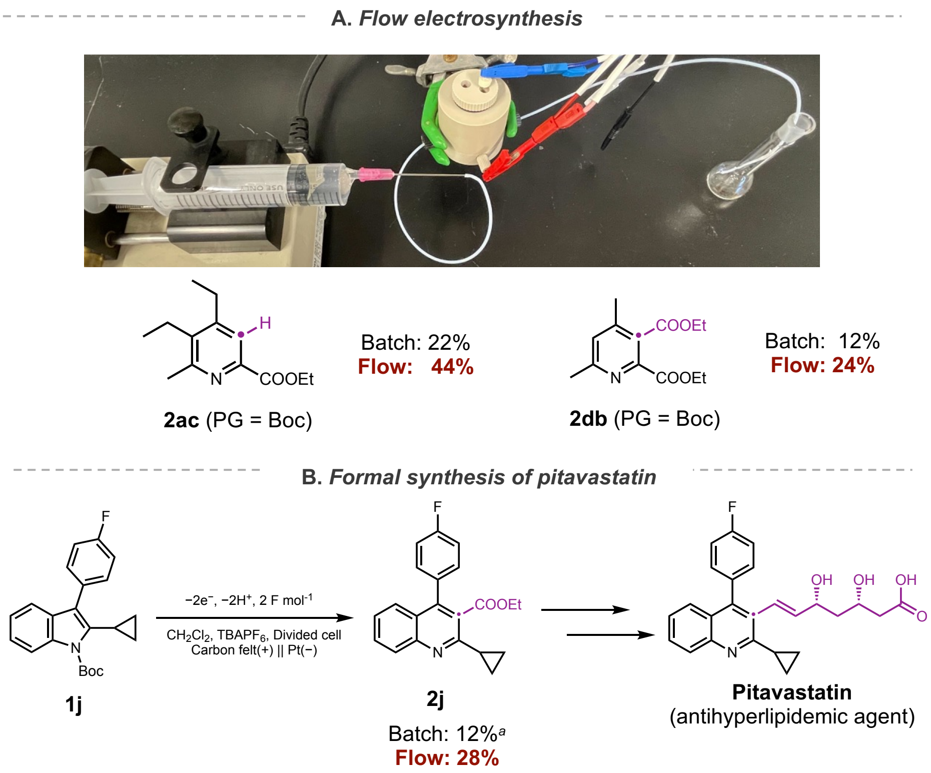

Figure S40. (A) Flow electrosynthesis for *Boc*-protected pyrroles. Conditions: substrates (1 equiv.), EDA (7 equiv.), CH_2Cl_2 (15 mM), 10 mA, 2 F mol^{-1}, 12.4 mL h^{-1}. Yields were determined by ^{1}H NMR using benzaldehyde as an internal standard. (B) Formal synthesis of Pitavastatin via electrochemical singl-carbon insertion

Electrochemical Single-carbon insertion to other compounds

The reaction with other *N*-containing five-membered aromatic compounds, such as indole- and imidazole-derivatives, afforded the corresponding quinoline- and pyrimidine-derivatives, respectively (Figure S41) (**2n-p**). Ring expansion of cyclic unsaturated hydrocarbons was also investigated. 1,2,3,4,5-Pentaphenyl cyclopentadiene (**3a**) afforded the corresponding hexa-substituted benzene derivative (**4a**) in good yield. The reaction was also effective with tetrasubstituted cyclopentadiene (**4b**), and single-carbon insertion products were observed from cyclopentadiene with multiple electron-withdrawing groups (**4ca, 4cb**). Additionally, the reaction was successful with indenes, leading to the formation of naphthalene derivatives (**4d**). Plausible reaction mechanism for single-carbon insertion into cyclopentadine rings are described in Figure S42.

From indole

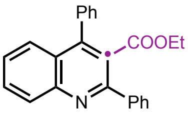

2n
PG = Boc 58%[a]

From imidazole

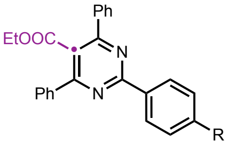

2o (R=H)
PG = Boc 21%
2p (R=F)
PG = Boc 16%

From cyclopentadiene and indene

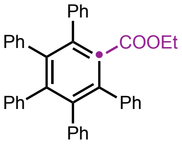

4a

68%

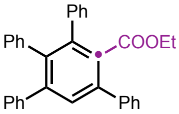

4b

23%

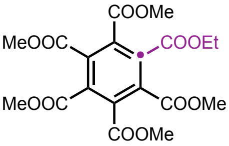

4ca

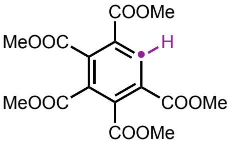

4cb

Total (**4ca**, **4cb**)
45% (30%, 15%)

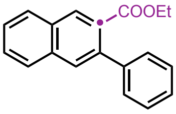

4d

45%

Figure S41. Electrochemical single-carbon insertion into indole-, imicazole-, cyclopentadiene-, and indene-derivatives.

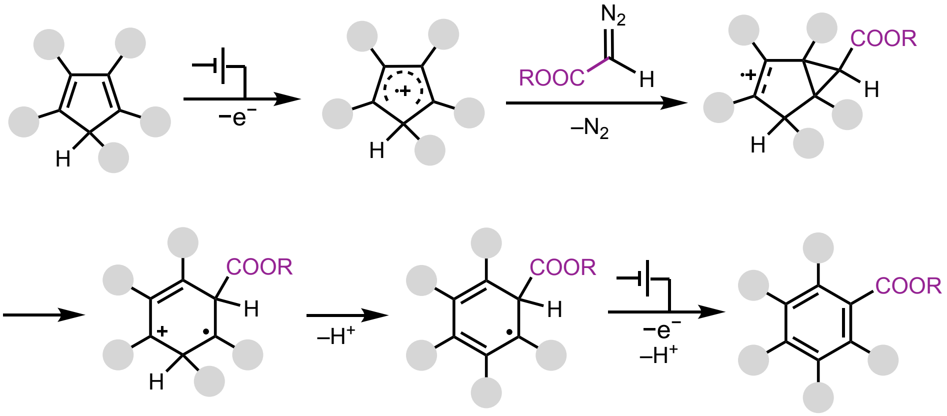

Figure S42. Plausible mechanism of electrochemical single-carbon insertion into cyclopentadiene ring.

Diversification of diester ***2ad***

To further diversify the obtained material, hydrolysis of **2ad** was performed. Interestingly, we successfully obtained **2t**, where only the *ortho*-positioned ester was selectively hydrolyzed, yielding the product in 89% yield (Scheme S1). Furthermore, **2t** was successfully condensed with NHPI to afford **2u**, where the NHPI ester is located at a different position compared to **2m**. Thus, these routes enable the complementary synthesis of pyridine-derivative bearing redox-active ester.

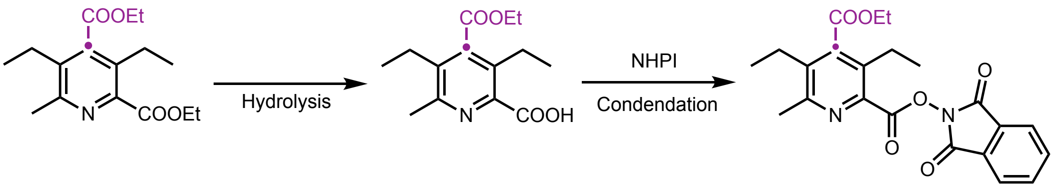

2ad | **2t** 89% | **2u** 86% (total : 77%)

Scheme S1. Hydrolysis of **2ad** and re-esterification with NHPI.

4.7. Limitations

The reaction did not proceed when fewer than two substitutions were present, or when there were no substituents at the 2- or 5-positions. Additionally, the reaction was inhibited by the presence of many electron-withdrawing substituents.

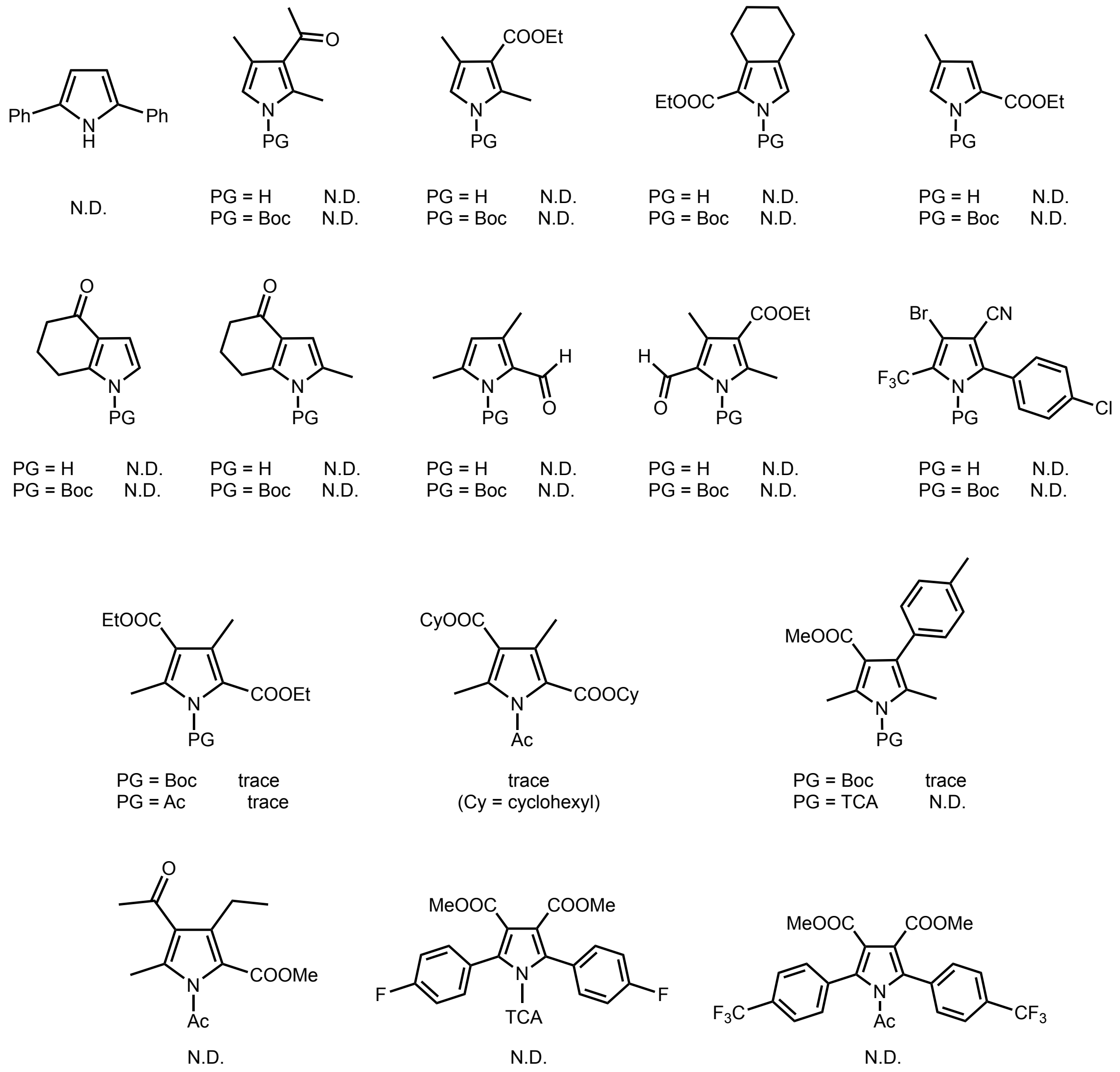

Figure S40. Substrate limitations.

5. Supporting References

(1) Sheldrick, G. M. Crystal Structure Refinement with SHELXL. *Acta Crystallogr C Struct Chem* **2015**, *71* (1). https://doi.org/10.1107/S2053229614024218, 3-8.

(2) Sheldrick, G. M. SHELXT - Integrated Space-Group and Crystal-Structure Determination. *Acta Crystallogr A Found Adv* **2015**, *71* (1). https://doi.org/10.1107/S2053273314026370, 3-8.

(3) Wang, Y.; Chen, C.; Peng, J.; Li, M. Copper(II)-Catalyzed Three-Component Cascade Annulation of Diaryliodoniums, Nitriles, and Alkynes: A Regioselective Synthesis of Multiply Substituted Quinolines. *Angew Chem Int Ed* **2013**, *52* (20). https://doi.org/10.1002/anie.201300586, 5431-5435.

(4) Kim, H.; Phan, N. H. T.; Shin, H.; Lee, H. S.; Sohn, J. H. Dehydrosulfurative Arylation with Concomitant Oxidative Dehydrogenation for Rapid Access to Pyrimidine Derivatives. *Tetrahedron* **2017**, *73* (47). https://doi.org/10.1016/j.tet.2017.10.010, 6604-6613.

(5) Suzuki, M.; Iwasaki, H.; Fujikawa, Y.; Kitahara, M.; Sakashita, M.; Sakoda, R. Synthesis and Biological Evaluations of Quinoline-Based HMG-CoA Reductase Inhibitors. *Bioorg Med Chem* **2001**, *9* (10). https://doi.org/10.1016/S0968-0896(01)00198-5, 2727-2743.

(6) Brydges, S.; Gildea, B.; Grealis, J. P.; Müller-Bunz, H.; Stradiotto, M.; Casey, M.; McGlinchey, M. J. Organic and Organometallic Derivatives of Pentaphenylbenzene, C_6Ph_5X: Correlation of Peripheral Phenyl Ring Orientations with the Steric Bulk of "x." *Can J Chem* **2013**, *91* (11). https://doi.org/10.1139/cjc-2013-0257, 1098-1111.

(7) Zeng, M. Q.; Feng, K. X.; Hu, B. L.; Tu, H. Y.; Zhang, X. G. Ni-Catalyzed Reductive Arylalkenylation of Alkynes for the Selective Synthesis of Polysubstituted Naphthalenes. *Org Lett* **2022**, *24* (29). https://doi.org/10.1021/acs.orglett.2c02068, 5386-5390.

(8) Merz, A.; Schropp, R.; Dotterl, E. 3,4-Dialkoxypyrroles and 2,3,7,8,12,13,17,18-Octaalkoxyporphyrins. *Synthesis (Stuttg)* 1995, No. 7. https://doi.org/10.1055/s-1995-3993.

(9) Kämpfe, A.; Brendler, E.; Kroke, E.; Wagler, J. 2-Acylpyrroles as Mono-Anionic O,N-Chelating Ligands in Silicon Coordination Chemistry. *Chemistry - A European Journal* 2014, *20* (30). https://doi.org/10.1002/chem.201402803.

(10) Lee, B.; Park, B. G.; Cho, W.; Lee, H. Y.; Olasz, A.; Chen, C. H.; Park, S. B.; Lee, D. BOIMPY: Fluorescent Boron Complexes with Tunable and Environment-

Responsive Light-Emitting Properties. *Chemistry - A European Journal* 2016, *22* (48). https://doi.org/10.1002/chem.201603837.
(11) Fang, P.; Li, M.; Ge, H. Room Temperature Palladium-Catalyzed Decarboxylative Ortho -Acylation of Acetanilides with α-Oxocarboxylic Acids. *J Am Chem Soc* **2010**, *132* (34). https://doi.org/10.1021/ja105245f, 11898-11899.
(12) Zheng, Z.; Wu, Y.; Lu, X.; Zhang, F. L.; Qi, M. F.; Sun, E.; Sun, B. Visible-Light-Mediated Metal-Free Decarboxylative Acylation of Electron-Deficient Quinolines Using α-Ketoacids under Ambient Air. *Tetrahedron* **2022**, *112*. https://doi.org/10.1016/j.tet.2022.132749, 132749.
(13) Zolfigol, M. A.; Choghamarani, A. G.; Shahamirian, M.; Safaiee, M.; Mohammadpoor-Baltork, I.; Mallakpour, S.; Abdollahi-Alibeik, M. 4-Phenyl-1,2,4-Triazole-3,5-Dione as a Novel and Reusable Reagent for the Aromatization of 1,4-Dihydropyridines under Mild Conditions. *Tetrahedron Lett* 2005, *46* (33). https://doi.org/10.1016/j.tetlet.2005.06.031.
(14) Liu, X.; Liu, C.; Cheng, X. Correction: Ring-Contraction of Hantzsch Esters and Their Derivatives to Pyrroles via Electrochemical Extrusion of Ethyl Acetate out of Aromatic Rings. *Green Chemistry* 2021, *23* (12). https://doi.org/10.1039/d1gc90054d.
(15) Ramanathan, M.; Liu, S. T. Cascade Annulations of Aryldiazonium Salts, Nitriles and Halo-Alkynes Leading to 3-Haloquinolines. Tetrahedron 2017, 73 (30). https://doi.org/10.1016/j.tet.2017.05.085, 4317–4322.
(16) Miyachi, N.; Yanagawa, Y.; Iwasaki, H.; Ohara,Y.; Hiyama, T. A Novel Synthetic Method of HMG-CoA Reductase Inhibitor NK-104 via a Hydroboration-Cross Coupling Sequence. Tetrahedron Letters 1993, 34 (51). https://doi.org/10.1016/S0040-4039(00)61407-7, 8267–8270.

6. NMR spectra

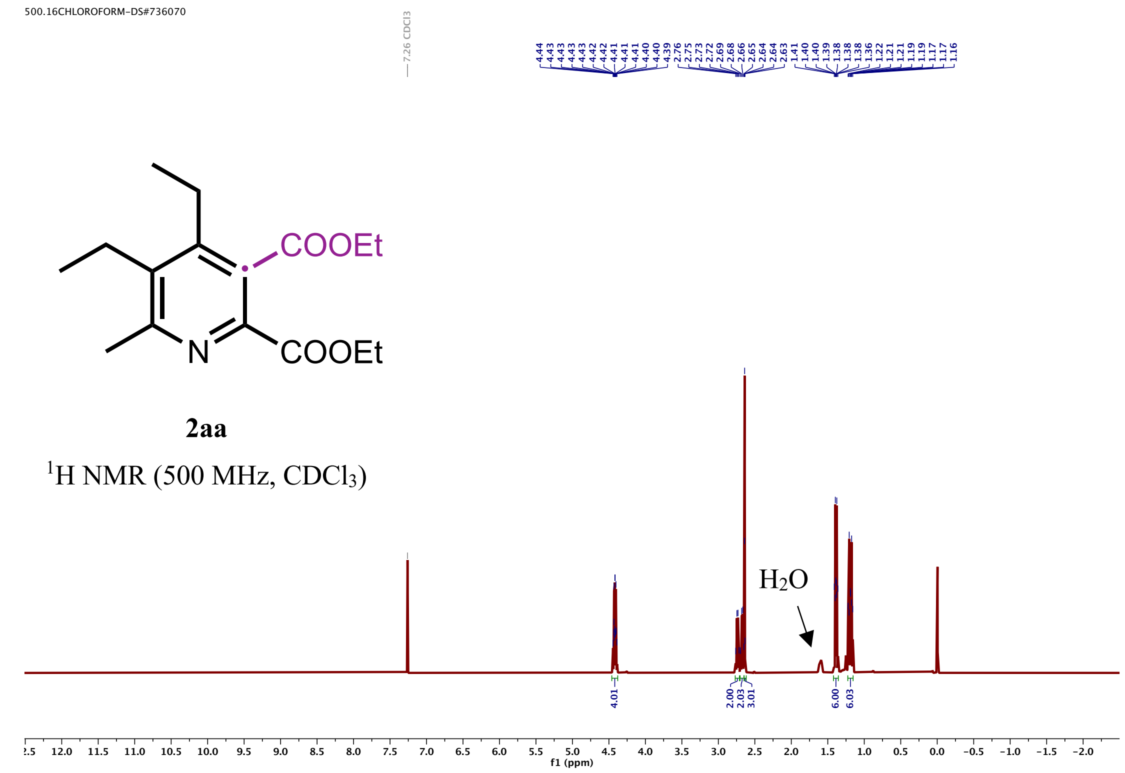

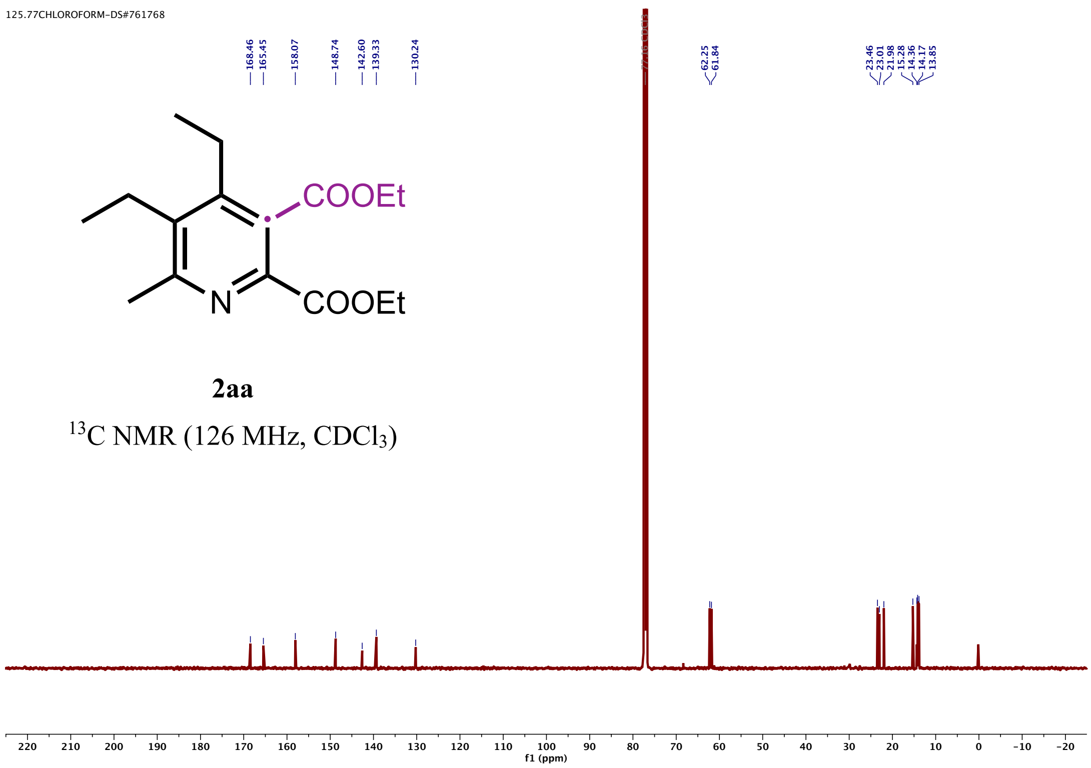

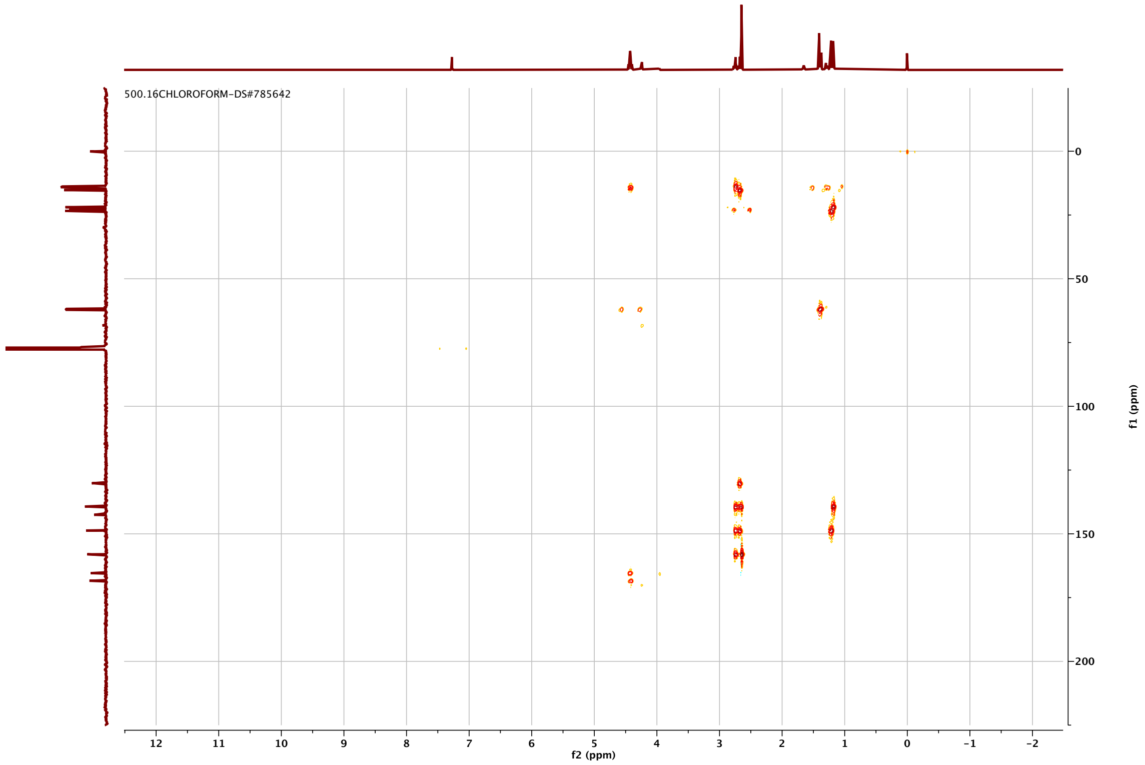

Heteronuclear Multiple Bond Coherence (HMBC) spectrum of diethyl 4,5-diethyl-6-methylpyridine-2,3-dicarboxylate **(2aa)**.

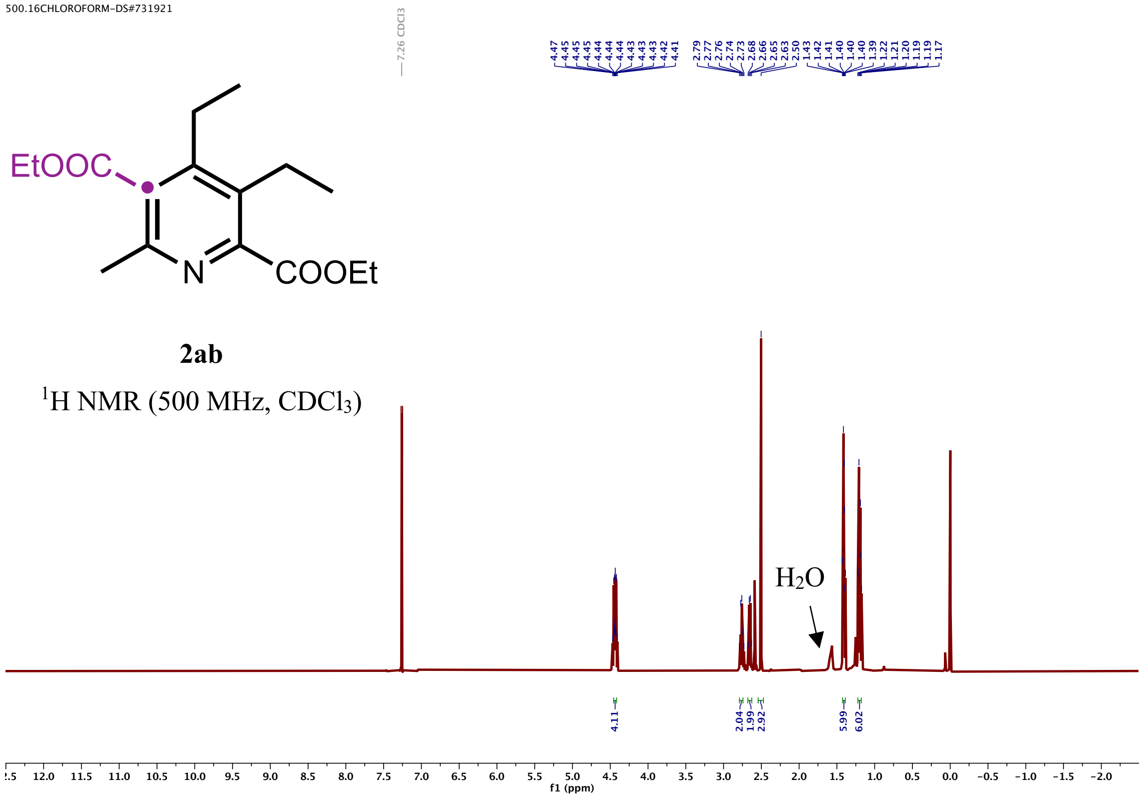
500.16CHLOROFORM-DS#731921
7.26 CDCl3
4.47 4.45 4.45 4.45 4.44 4.44 4.44 4.43 4.43 4.43 4.42 4.41
2.79 2.77 2.76 2.74 2.73 2.68 2.66 2.65 2.63 2.50
1.43 1.42 1.41 1.40 1.40 1.40 1.39 1.22 1.21 1.20 1.19 1.19 1.17
EtOOC
N
COOEt
2ab
¹H NMR (500 MHz, CDCl3)
H2O
4.11
2.04 1.99 2.92
5.99
6.02
f1 (ppm)

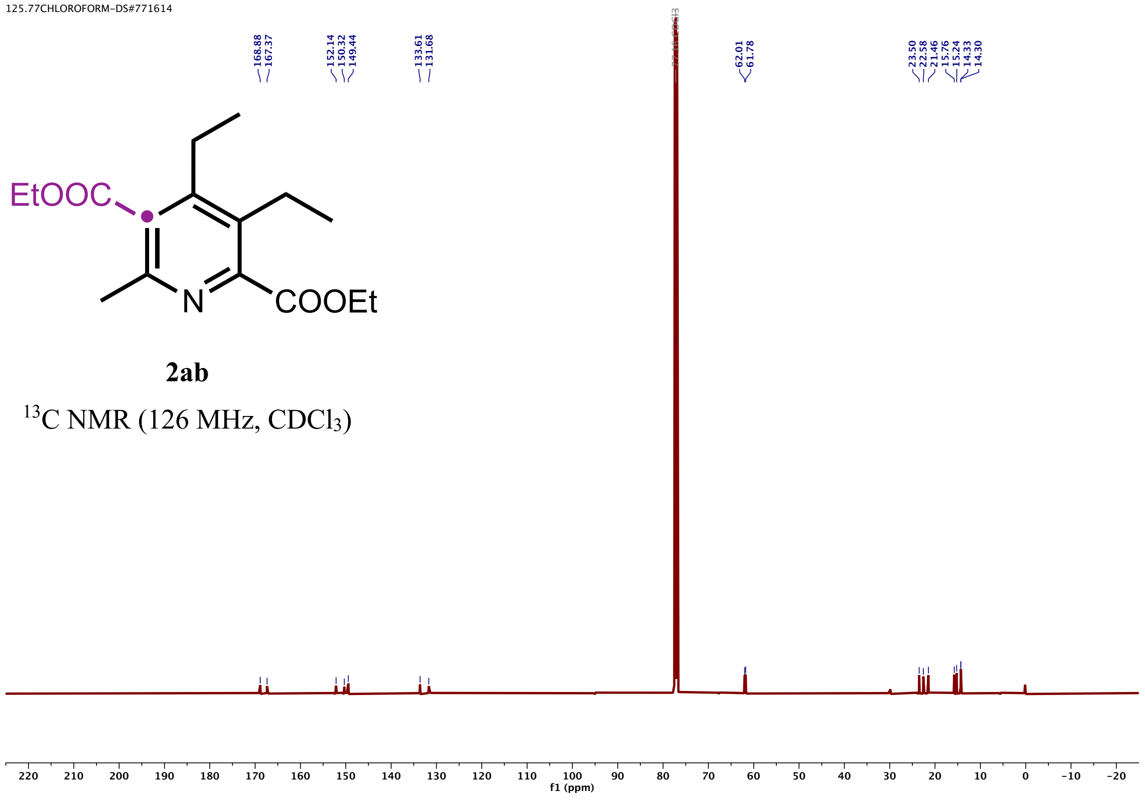
125.77CHLOROFORM-DS#771614
168.88 167.37
152.14 150.32 149.44
133.61 131.68
62.01 61.78
23.50 22.58 21.46 15.76 15.24 14.33 14.30
EtOOC
N
COOEt
2ab
¹³C NMR (126 MHz, CDCl3)
f1 (ppm)

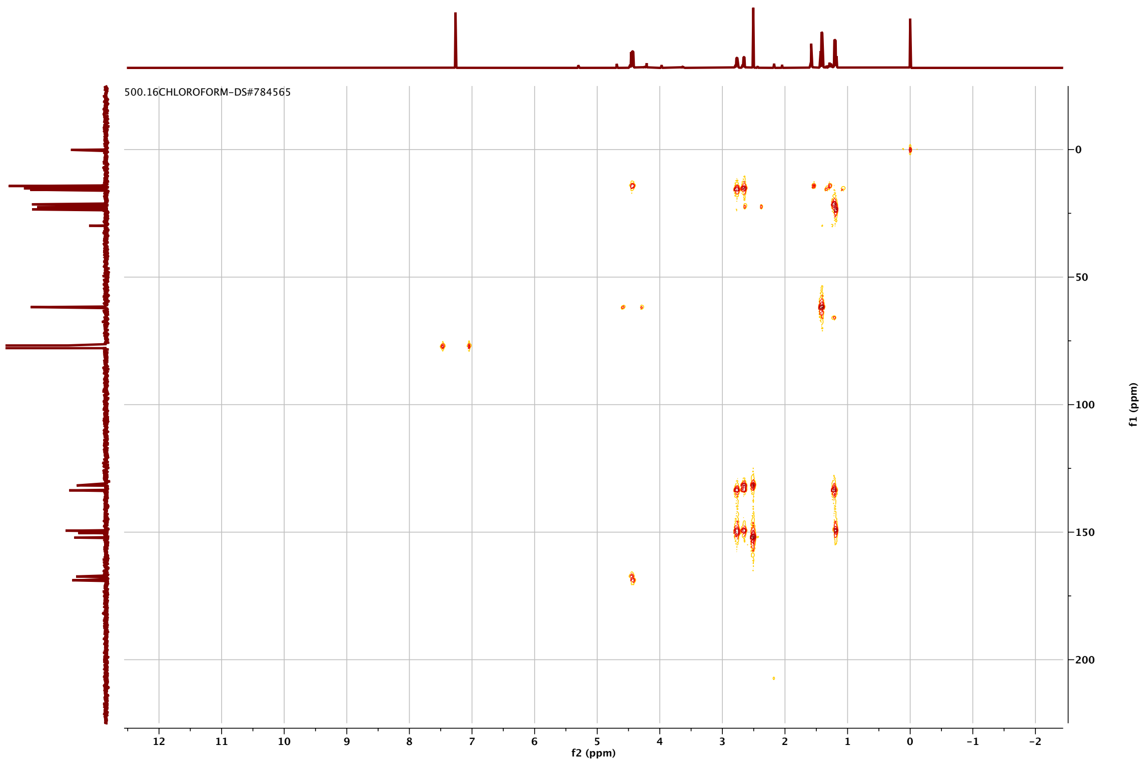

HMBC spectrum of diethyl 4,5-diethyl-6-methylpyridine-2,3-dicarboxylate **(2ab)**.

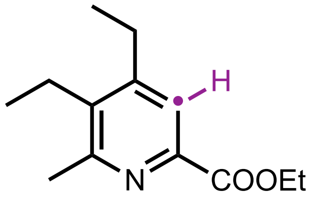

2ac

^{1}H NMR (500 MHz, $CDCl_3$)

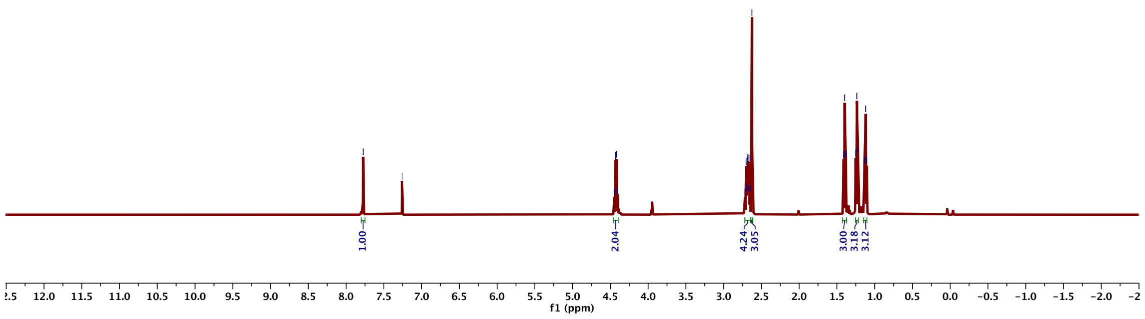

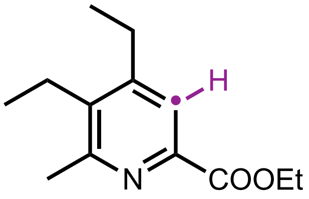

2ac

^{13}C NMR (126 MHz, $CDCl_3$)

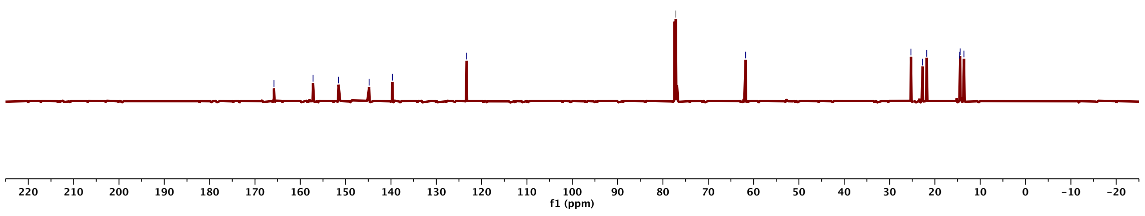

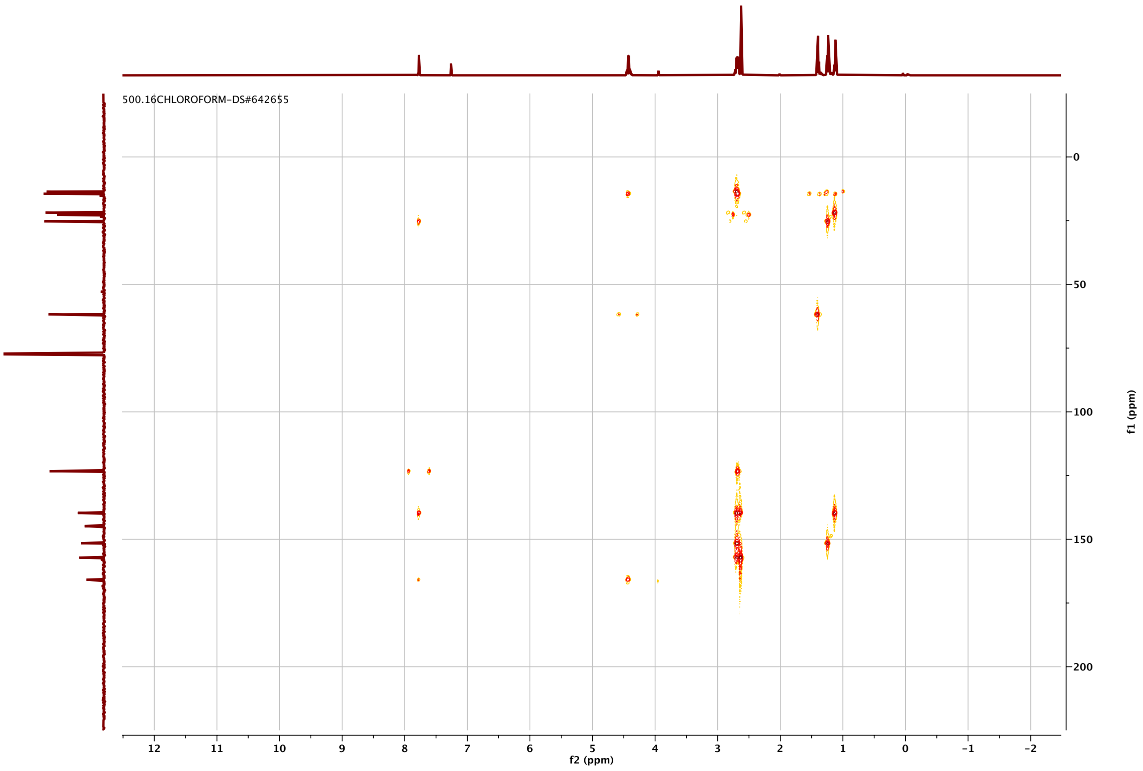

HMBC spectrum of 3,4-diethyl-2,6-dimet**hylpyridine (2ac)**.

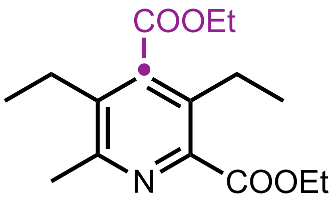

2ad

^1H NMR (500 MHz, $CDCl_3$)

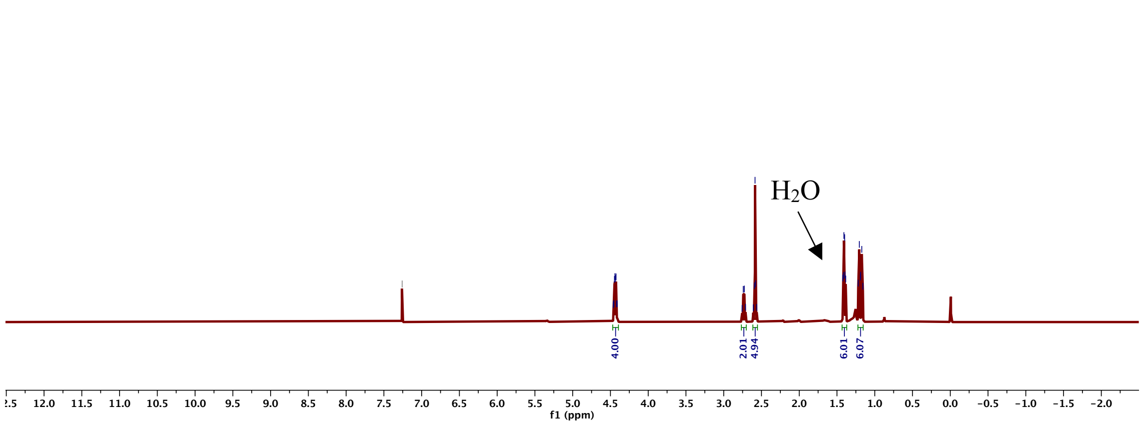

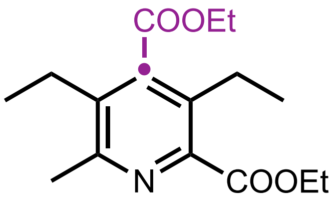

2ad

^{13}C NMR (126 MHz, $CDCl_3$)

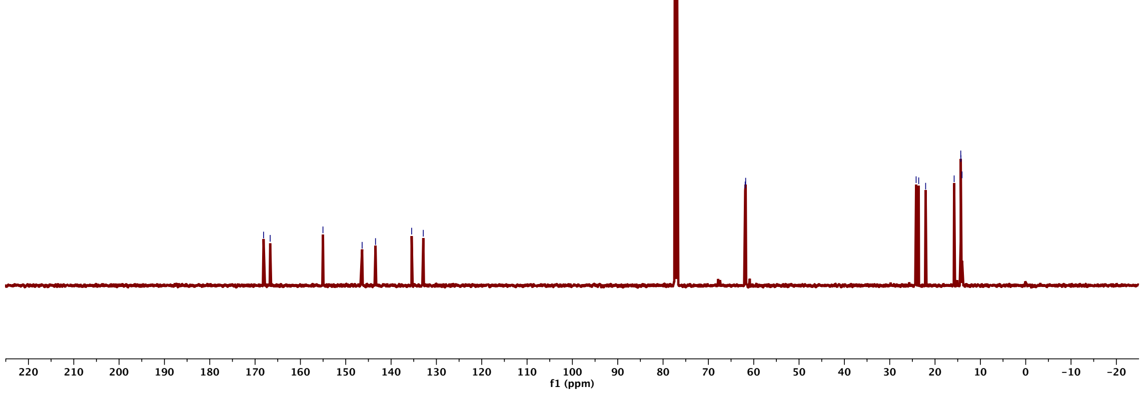

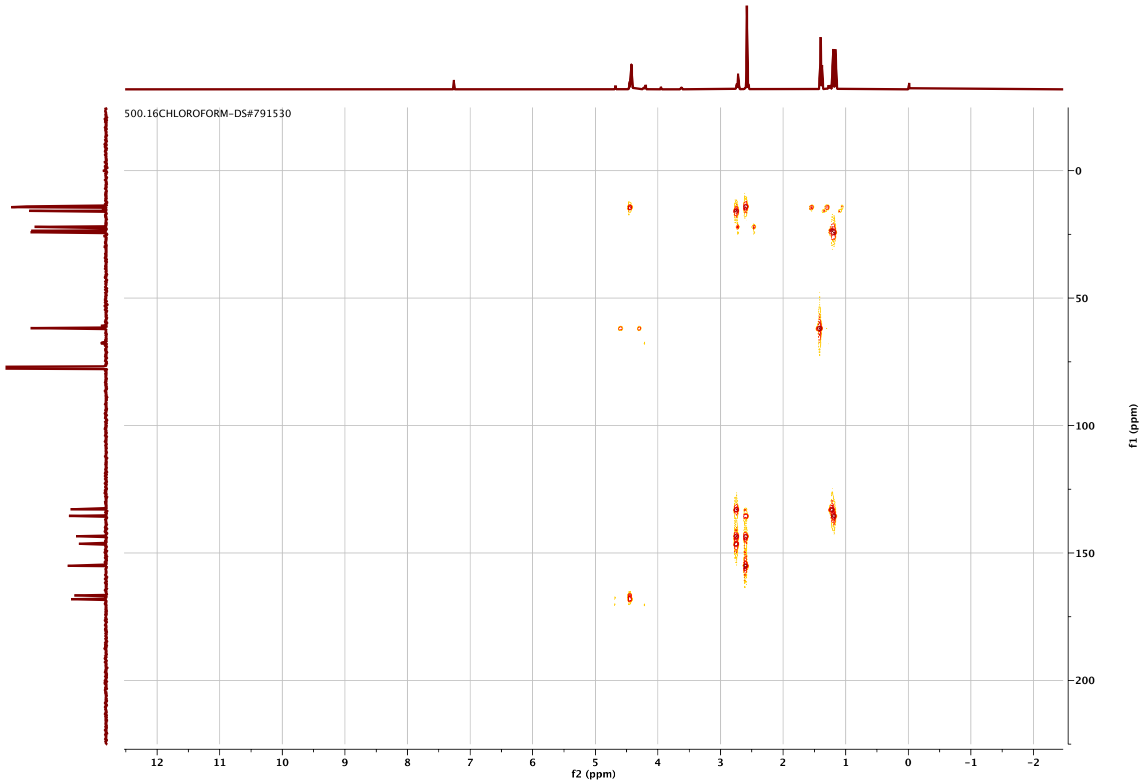

HMBC spectrum of diethyl 3,5-diethyl-6-methylpyridine-2,4-dicarboxylate **(2ad)**.

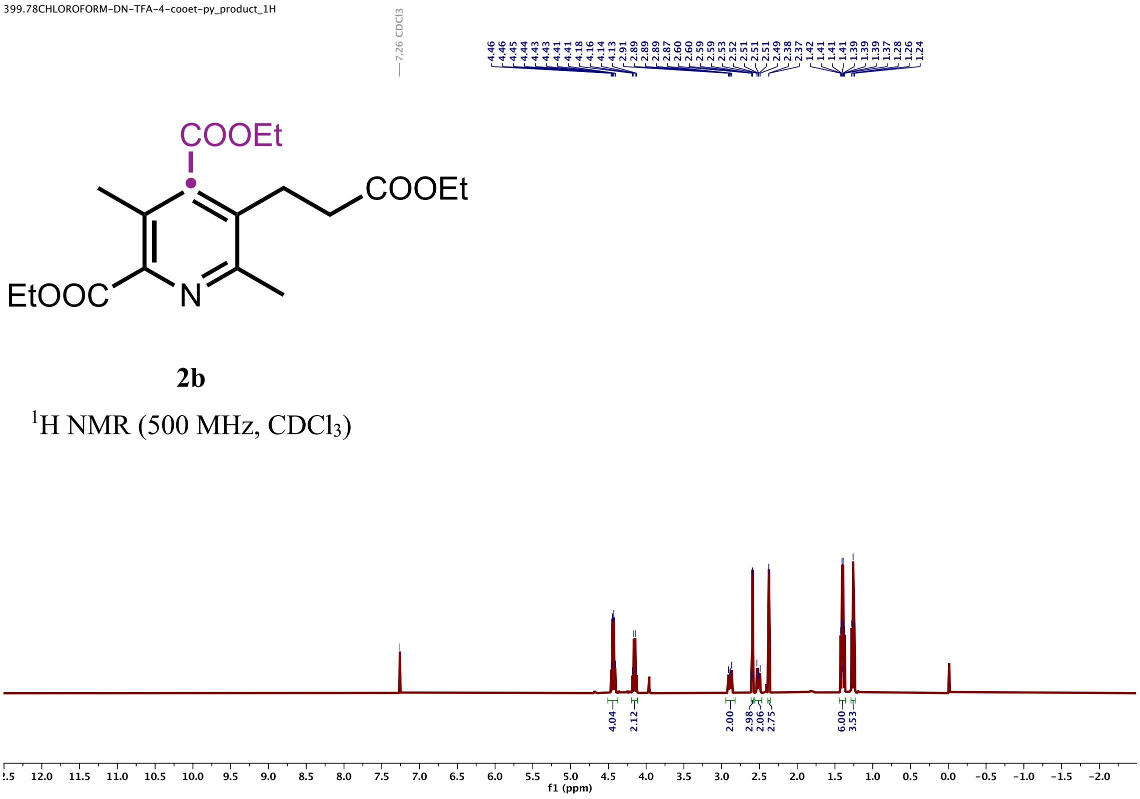

2b

^{1}H NMR (500 MHz, $CDCl_3$)

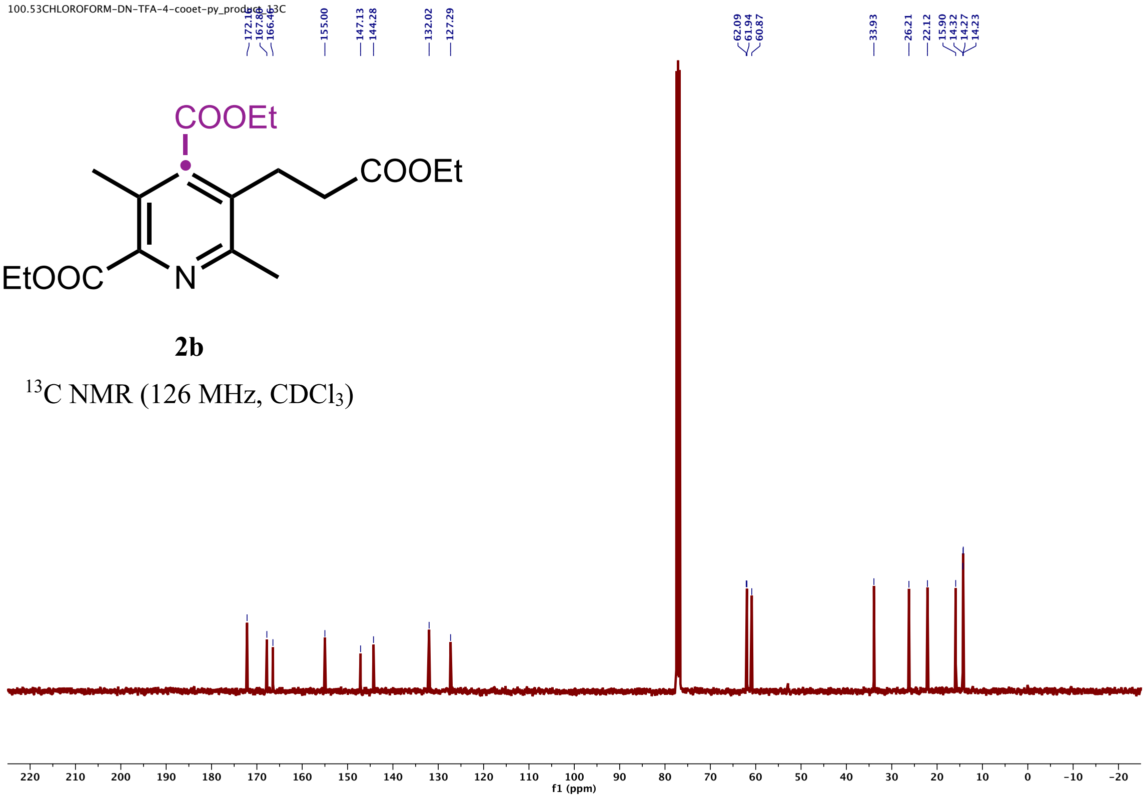

2b

^{13}C NMR (126 MHz, $CDCl_3$)

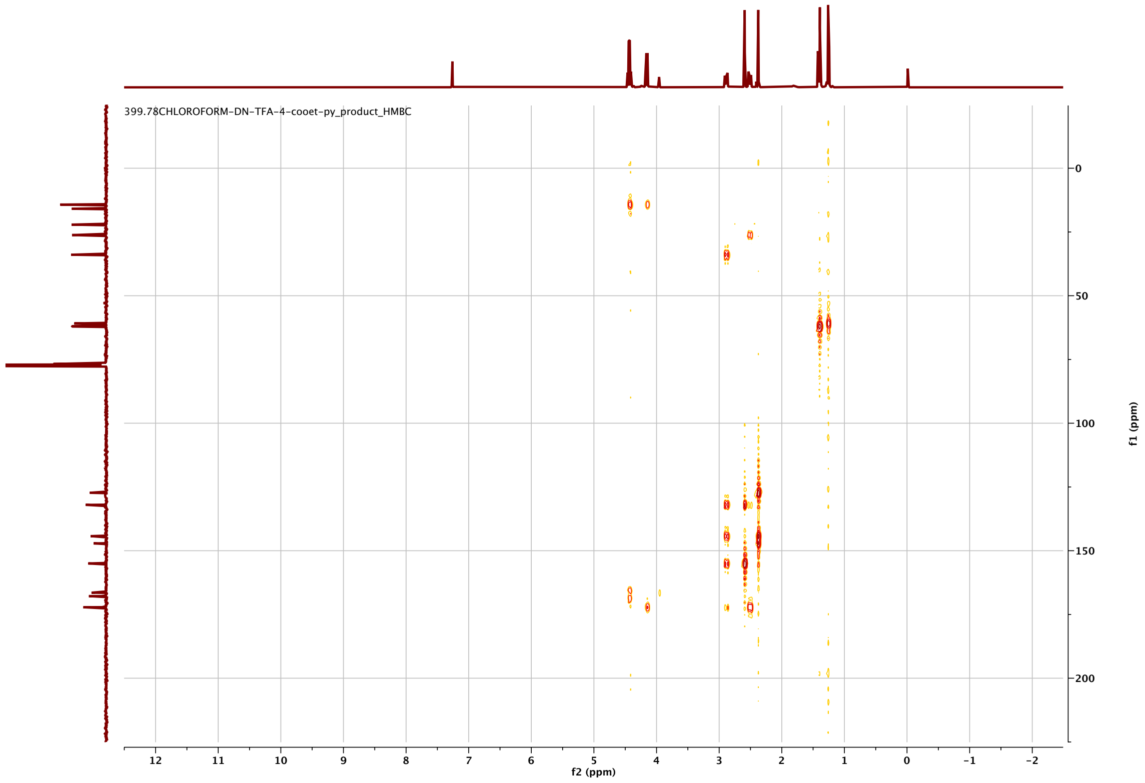

HMBC spectrum of diethyl 5-(3-ethoxy-3-oxopropyl)-3,6-dimethylpyridine-2,4-dicarboxylate **(2b)**.

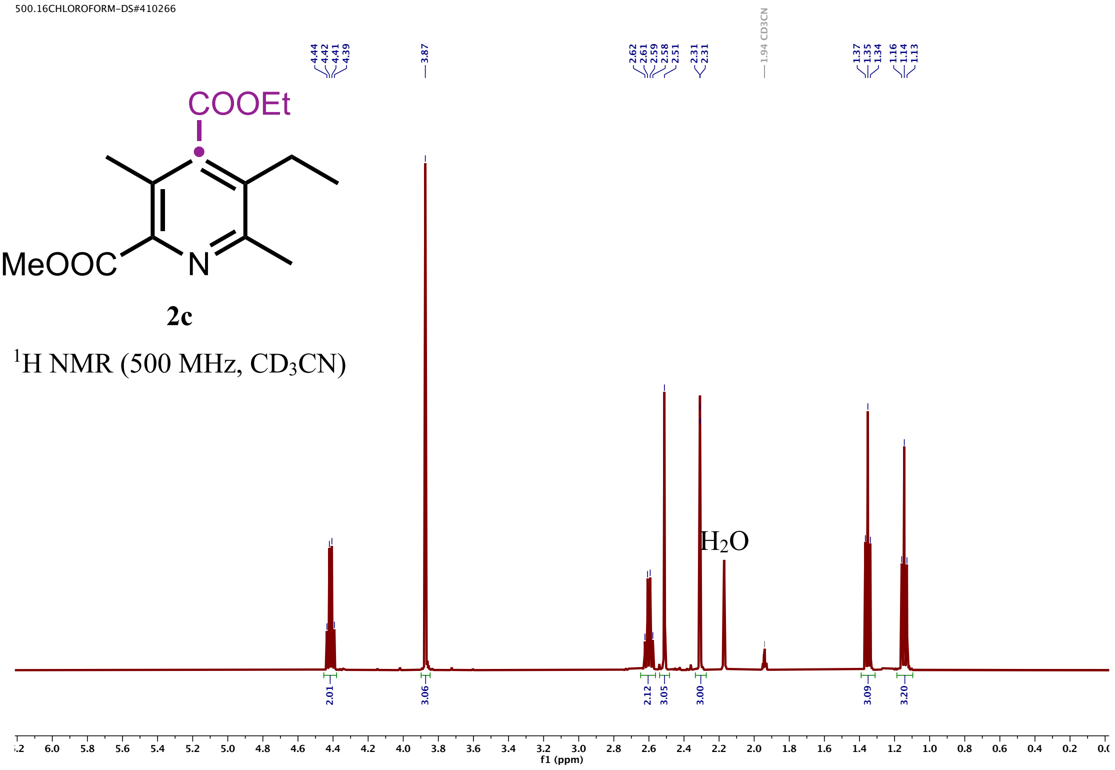
500.16CHLOROFORM-DS#410266
4.44
4.42
4.41
4.39
3.87
2.62
2.61
2.59
2.58
2.51
2.31
2.31
1.94 CD3CN
1.37
1.35
1.34
1.16
1.14
1.13
COOEt
MeOOC
N
2c
1H NMR (500 MHz, CD3CN)
H2O
2.01
3.06
2.12
3.05
3.00
3.09
3.20
f1 (ppm)

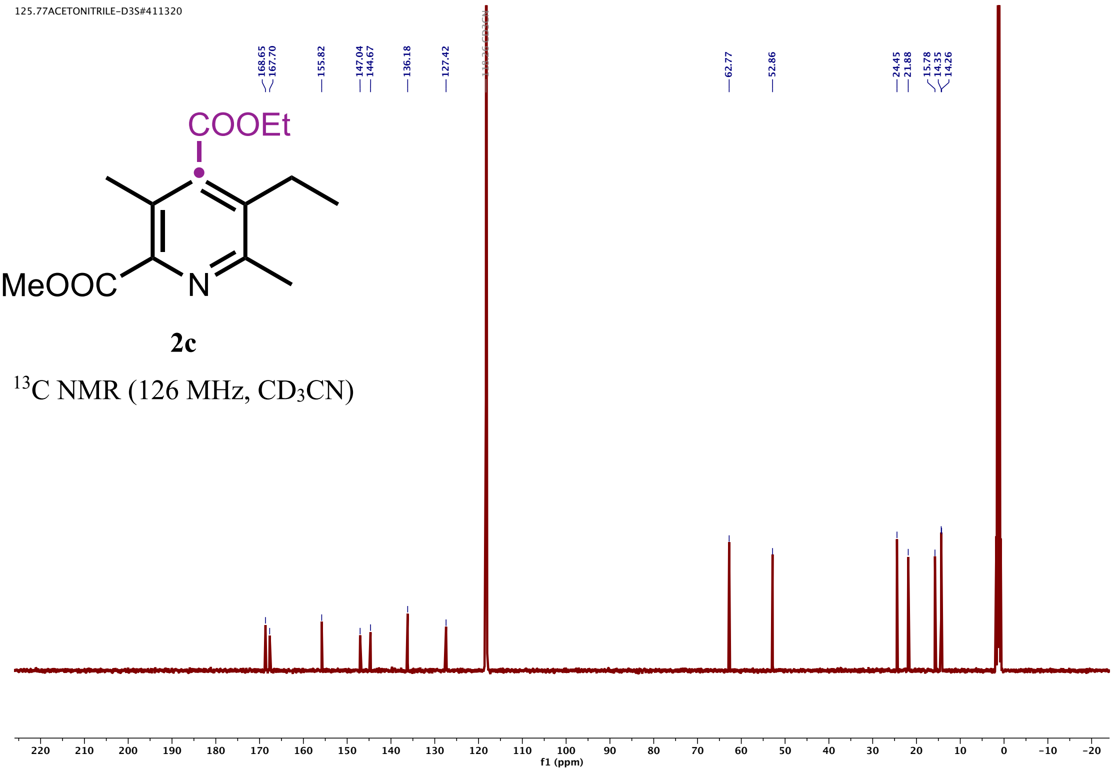
125.77ACETONITRILE-D3S#411320
168.65
167.70
155.82
147.04
144.67
136.18
127.42
62.77
52.86
24.45
21.88
15.78
14.35
14.26
COOEt
MeOOC
N
2c
13C NMR (126 MHz, CD3CN)
f1 (ppm)

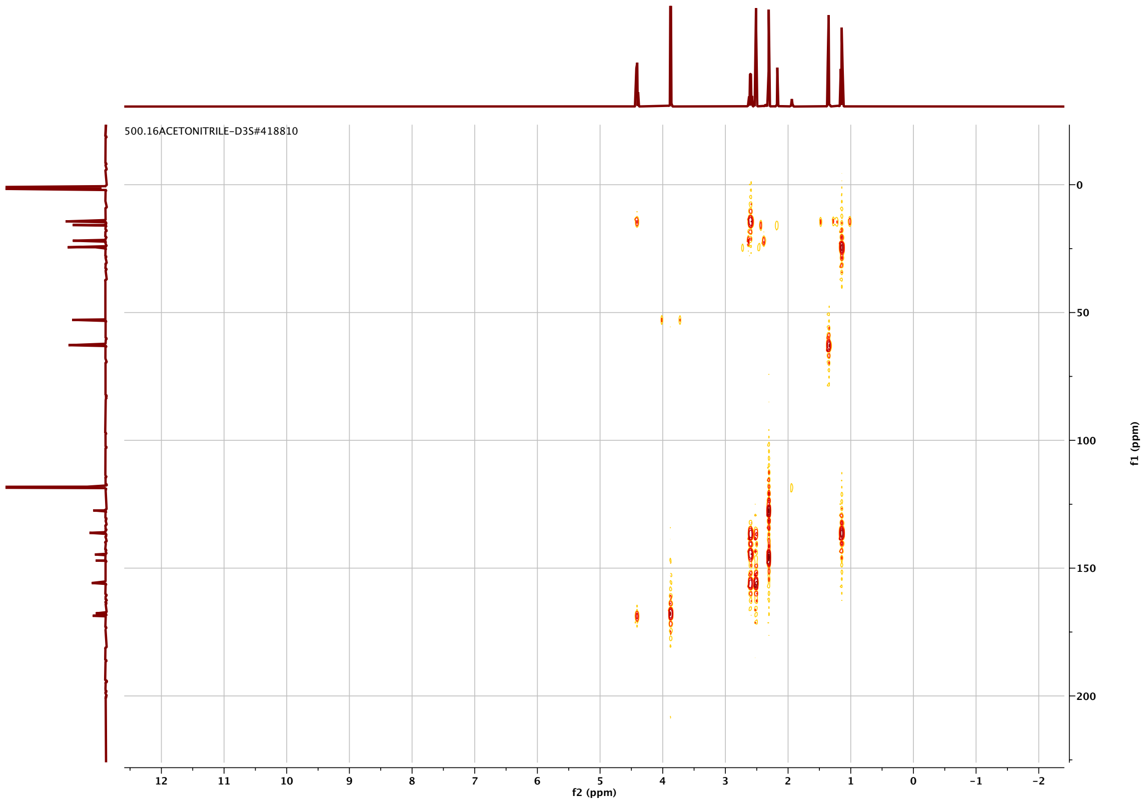

HMBC spectrum of 4-ethyl 2-methyl 5-ethyl-3,6-dimethylpyridine-2,4-dicarboxylate **(2c)**.

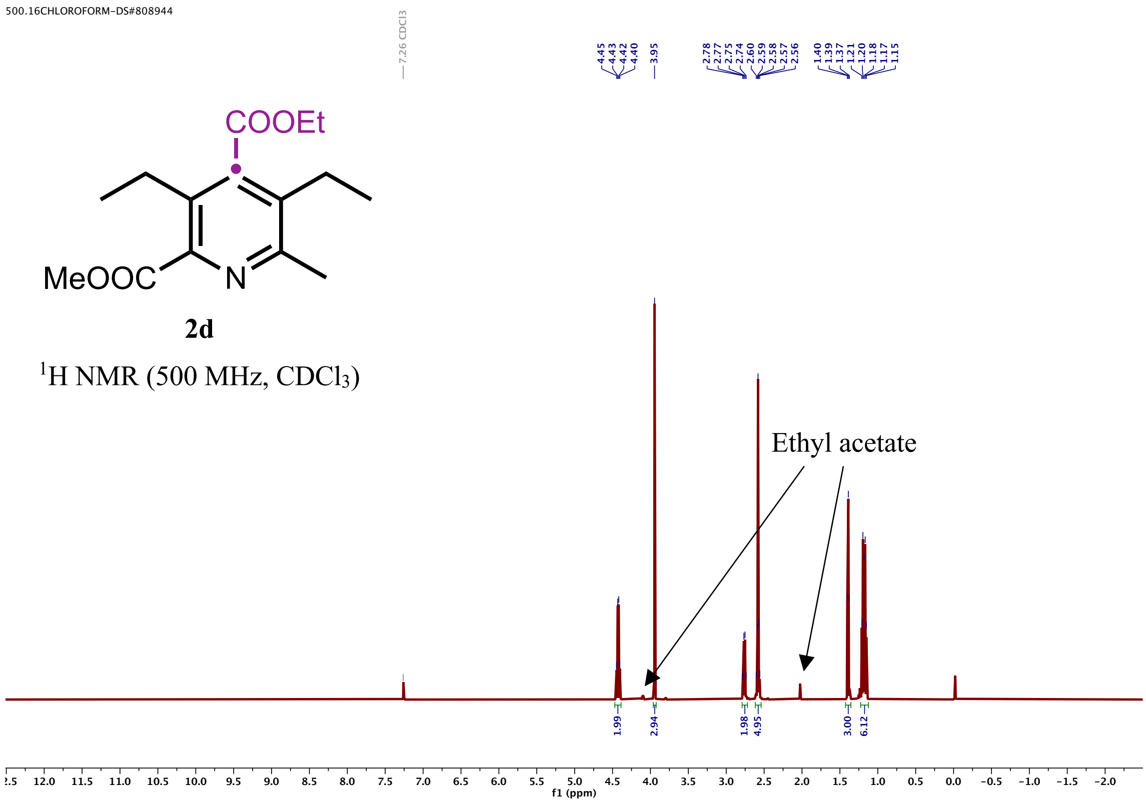

500.16CHLOROFORM-DS#808944
7.26 CDCl3
4.45
4.43
4.42
4.40
3.95
2.78
2.77
2.75
2.74
2.60
2.59
2.58
2.57
2.56
1.40
1.39
1.37
1.21
1.20
1.18
1.17
1.15
COOEt
MeOOC
N
2d
^{1}H NMR (500 MHz, $CDCl_3$)
Ethyl acetate
1.99
2.94
1.98
4.95
3.00
6.12
f1 (ppm)

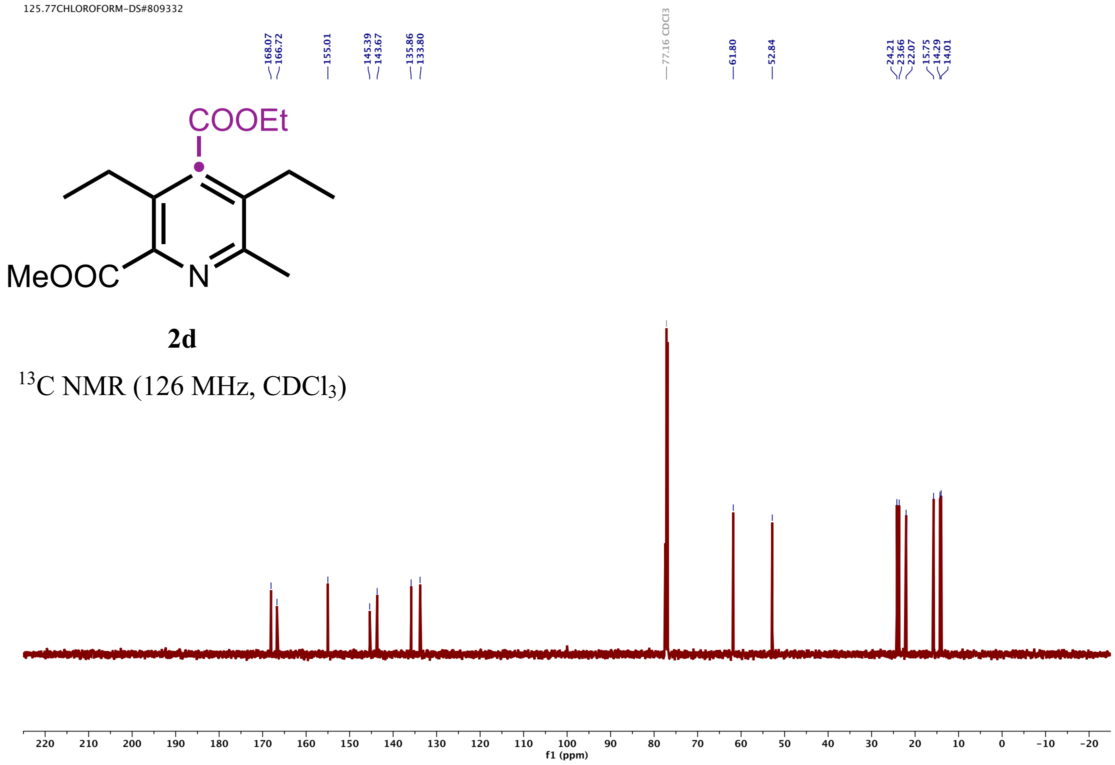

125.77CHLOROFORM-DS#809332
168.07
166.72
155.01
145.39
143.67
135.86
133.80
77.16 CDCl3
61.80
52.84
24.21
23.66
22.07
15.75
14.29
14.01
COOEt
MeOOC
N
2d
^{13}C NMR (126 MHz, $CDCl_3$)
f1 (ppm)

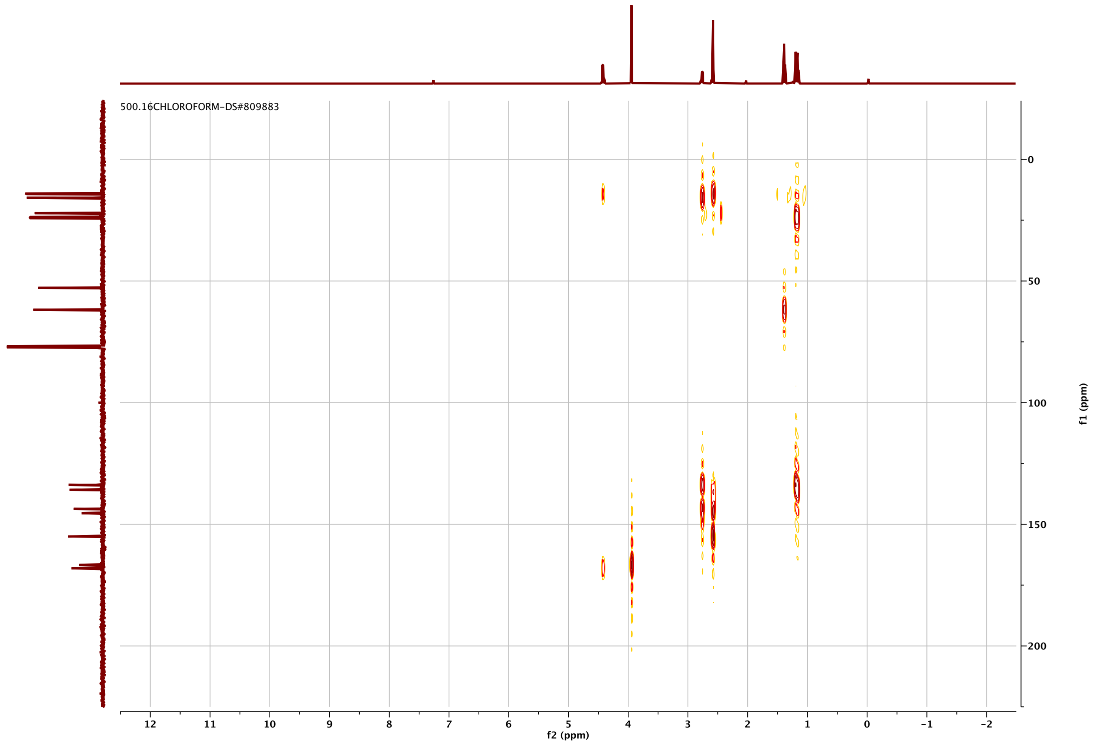

HMBC spectrum of 4-ethyl 2-methyl 3,5-diethyl-6-methylpyridine-2,4-dicarboxylate **(2d)**.

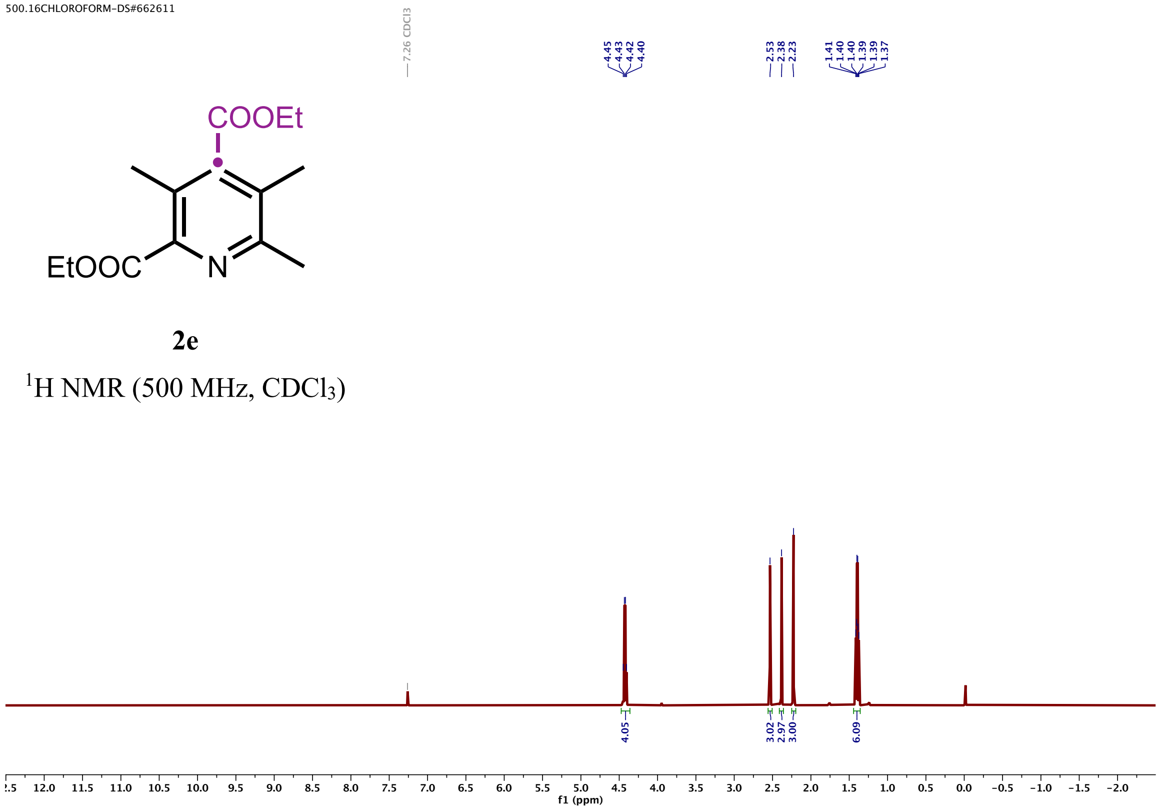

2e

^{1}H NMR (500 MHz, $CDCl_3$)

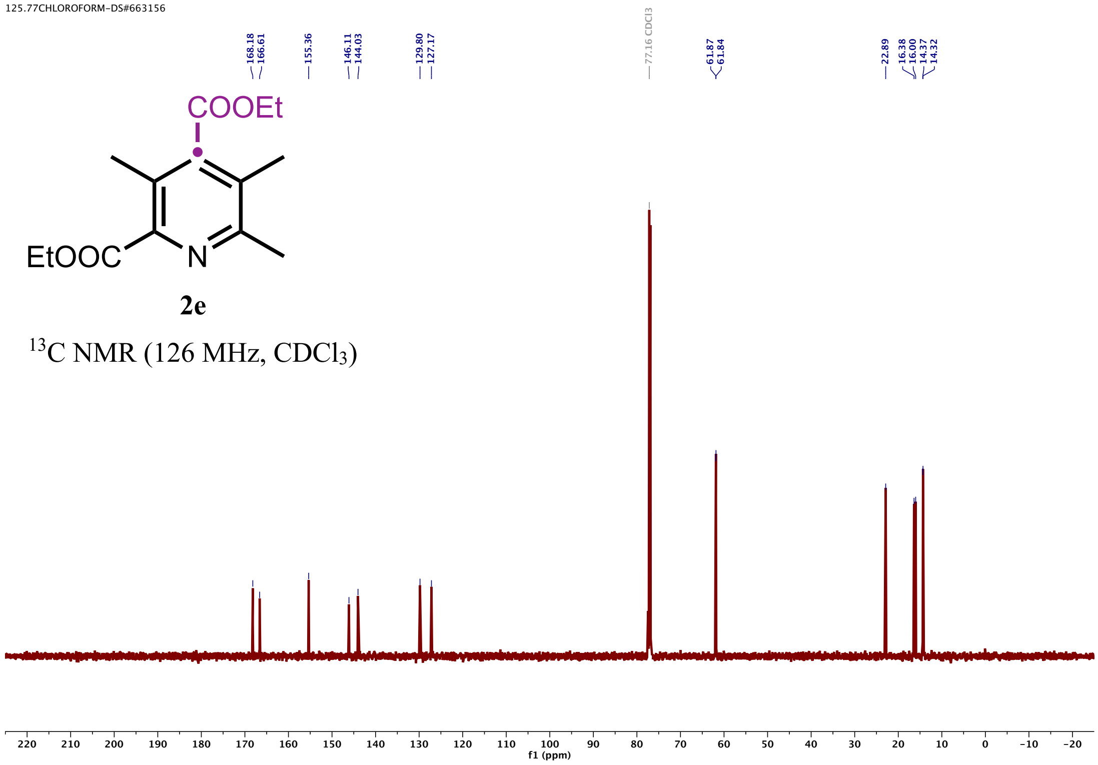

2e

^{13}C NMR (126 MHz, $CDCl_3$)

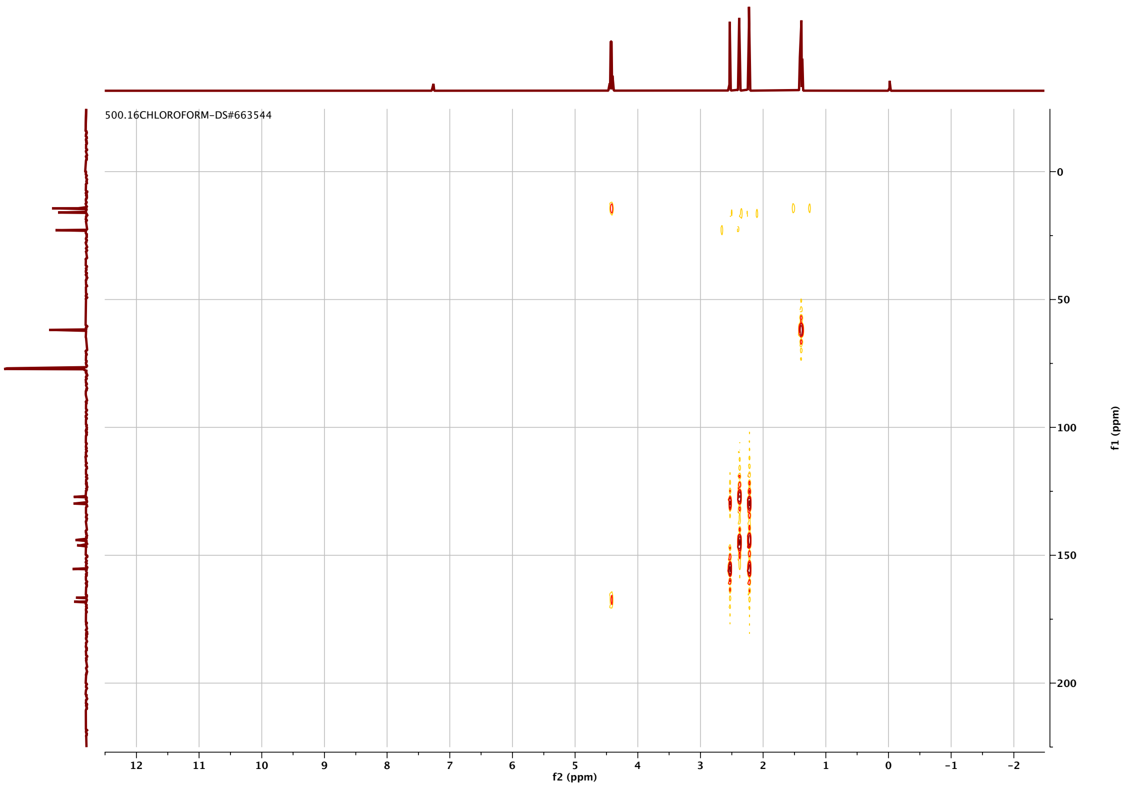

HMBC spectrum of diethyl 3,5,6-trimethylpyridine-2,4-dicarboxylate **(2e)**

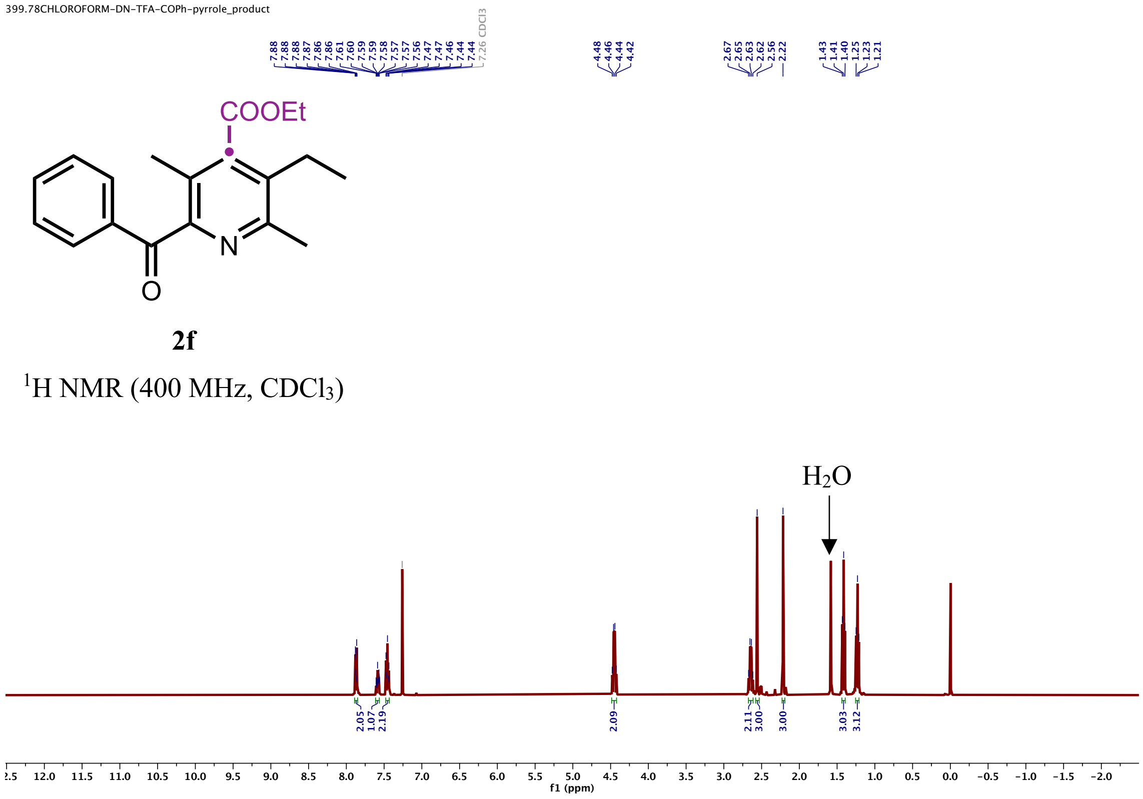

^{1}H NMR (400 MHz, $CDCl_3$)

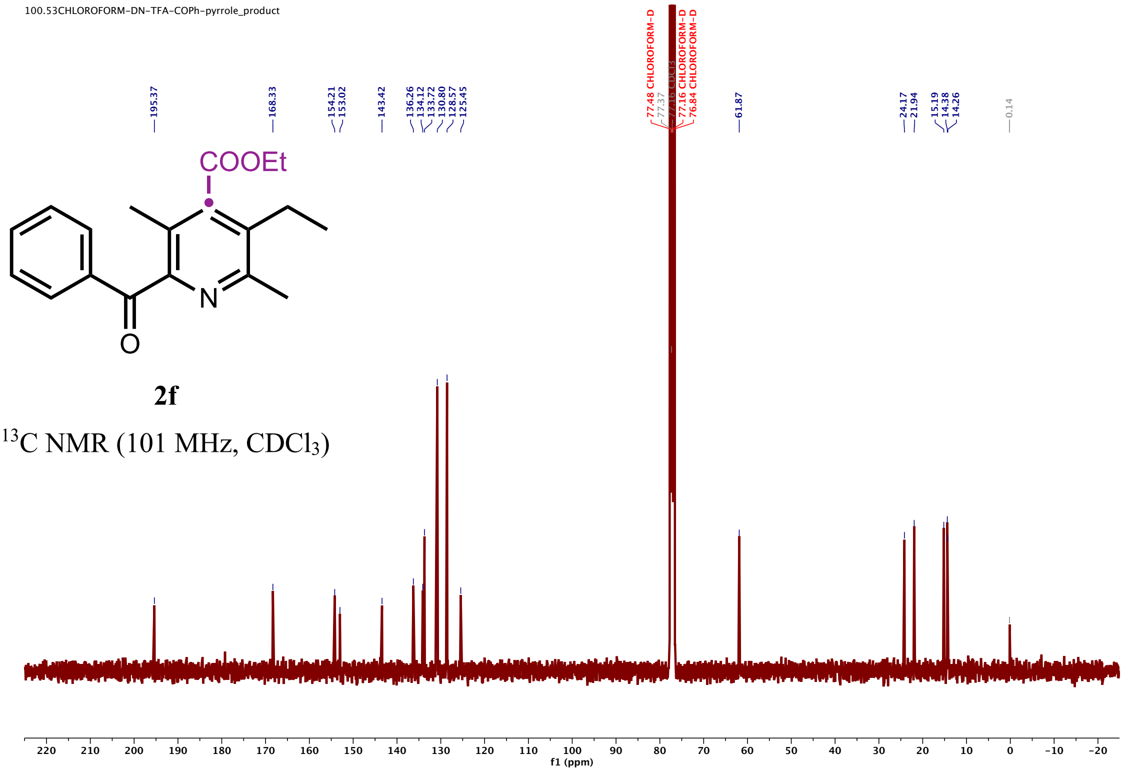

^{13}C NMR (101 MHz, $CDCl_3$)

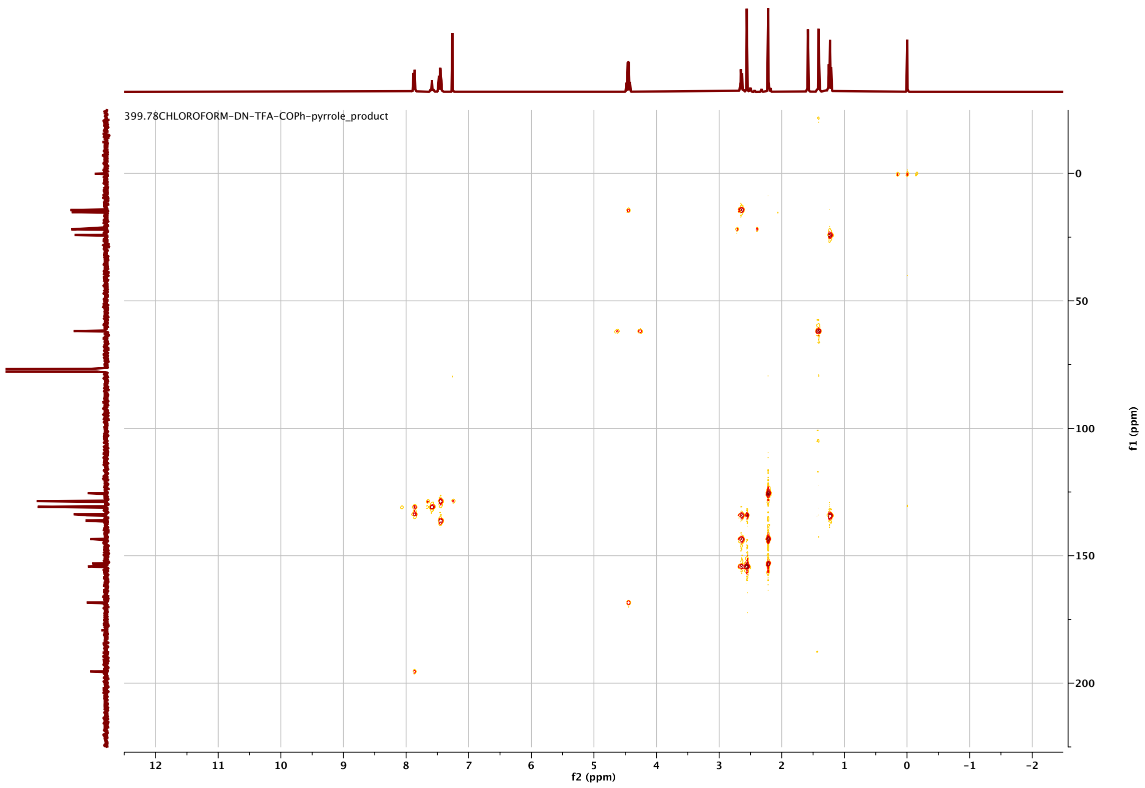

HMBC spectrum of ethyl 2-benzoyl-5-ethyl-3,6-dimethylisonicotinate (**2f**)

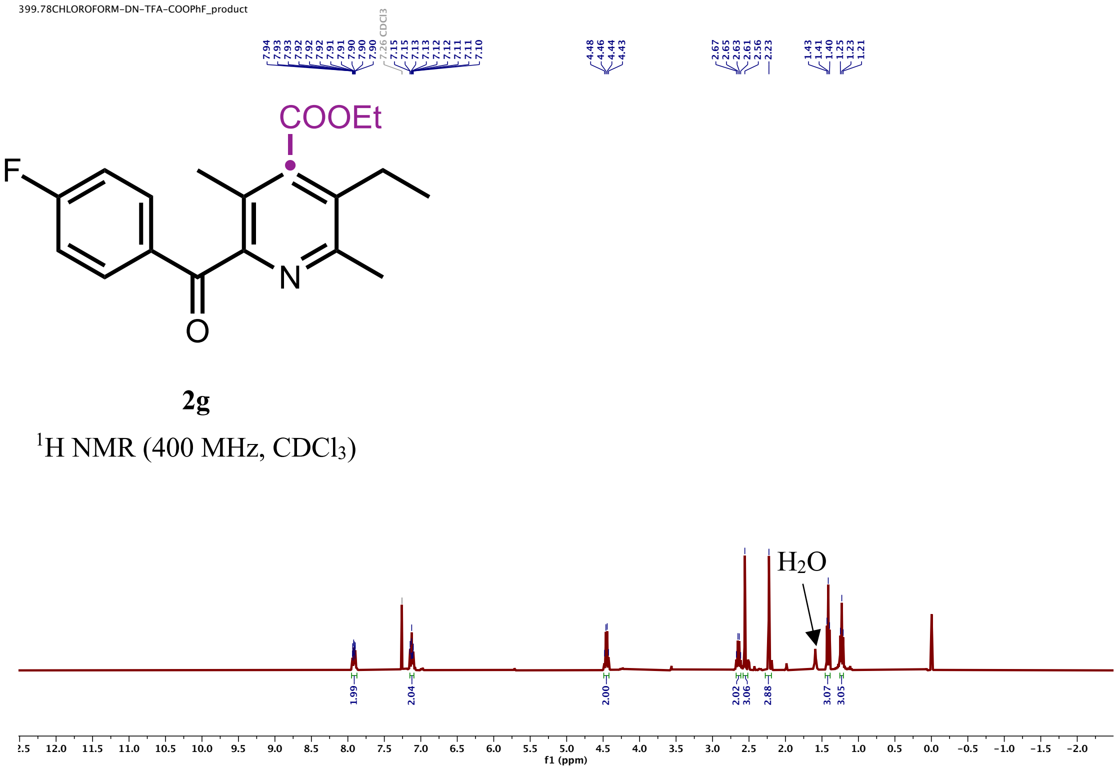

2g

^{1}H NMR (400 MHz, $CDCl_3$)

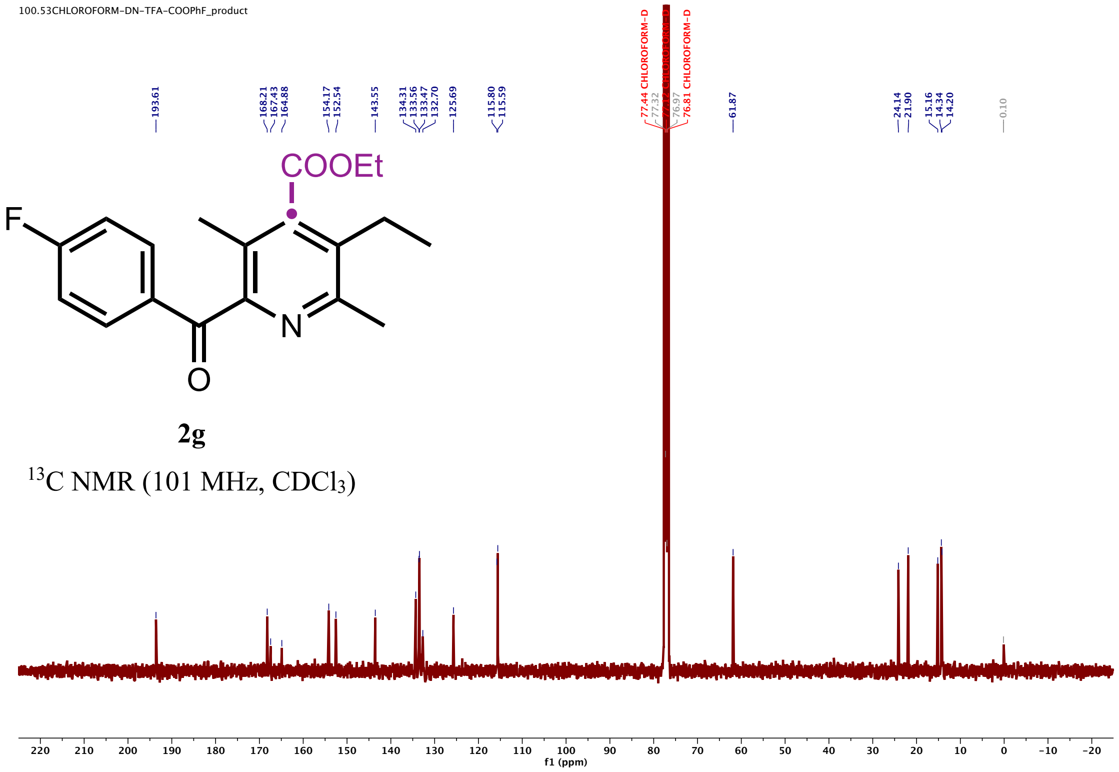

2g

^{13}C NMR (101 MHz, $CDCl_3$)

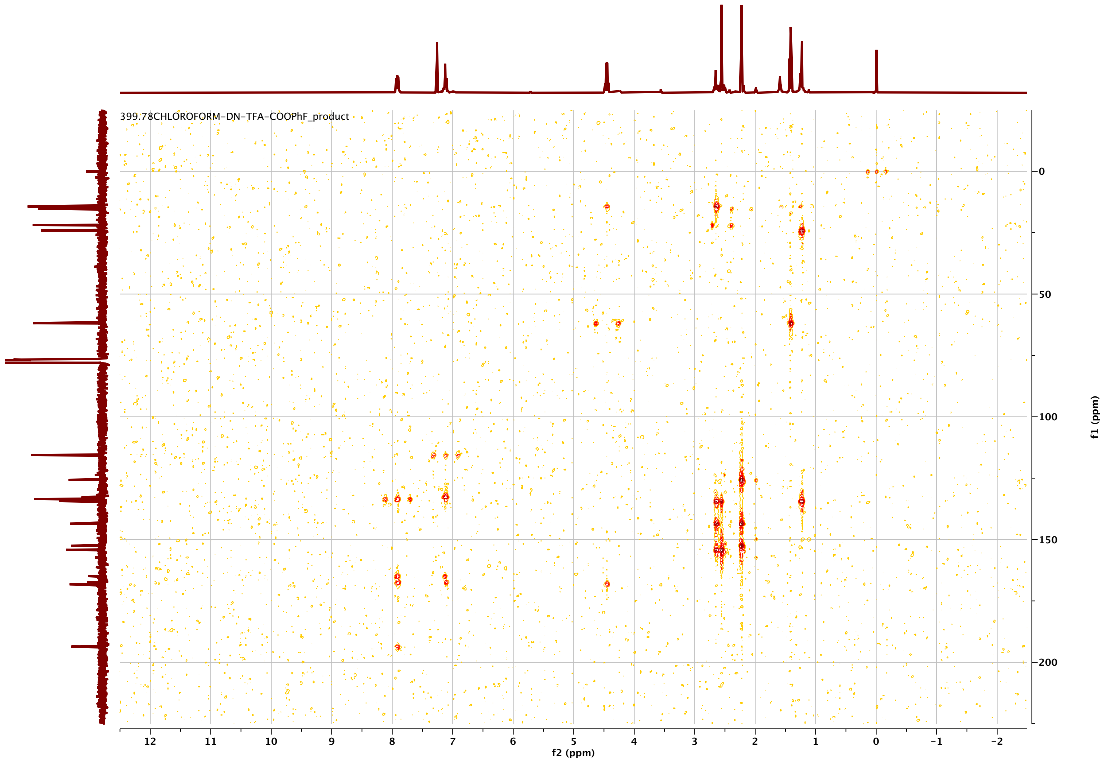

HMBC spectrum of ethyl 3-ethyl-6-(4-fluorobenzoyl)-2,5-dimethylisonicotinate (**2g**)

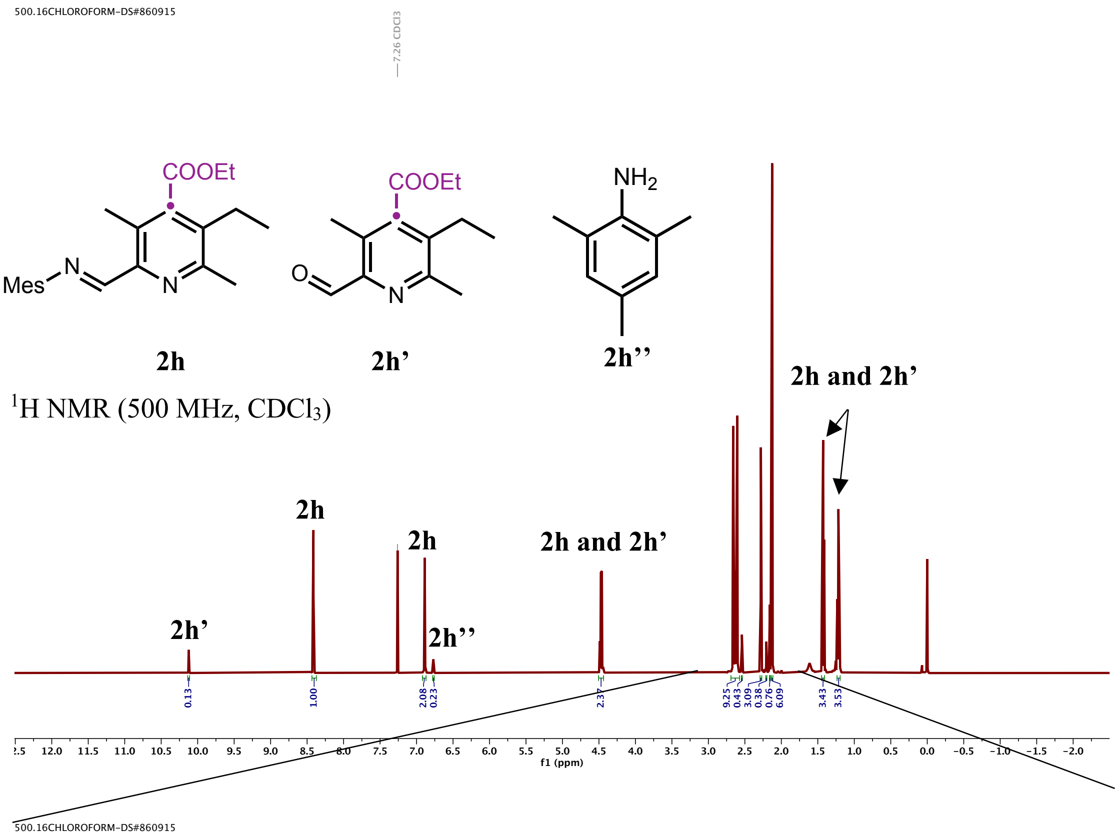

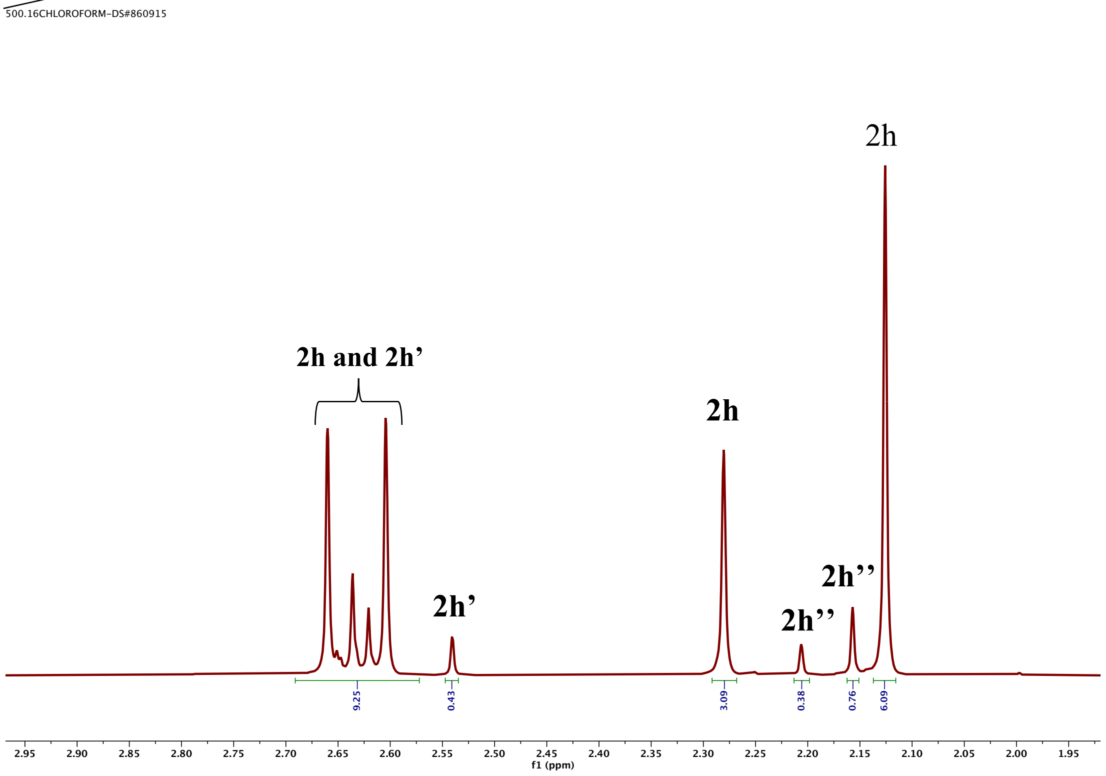

Enlarged ^{1}H NMR **(2h, 2h’, 2h’’)**

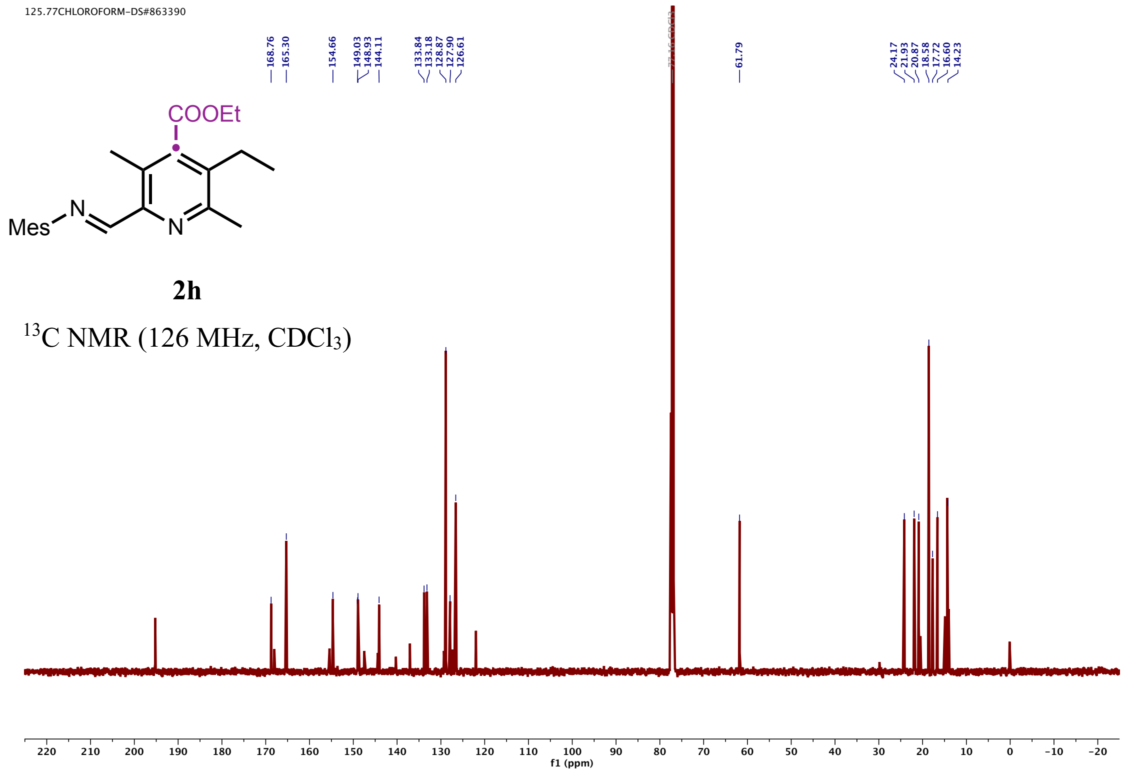

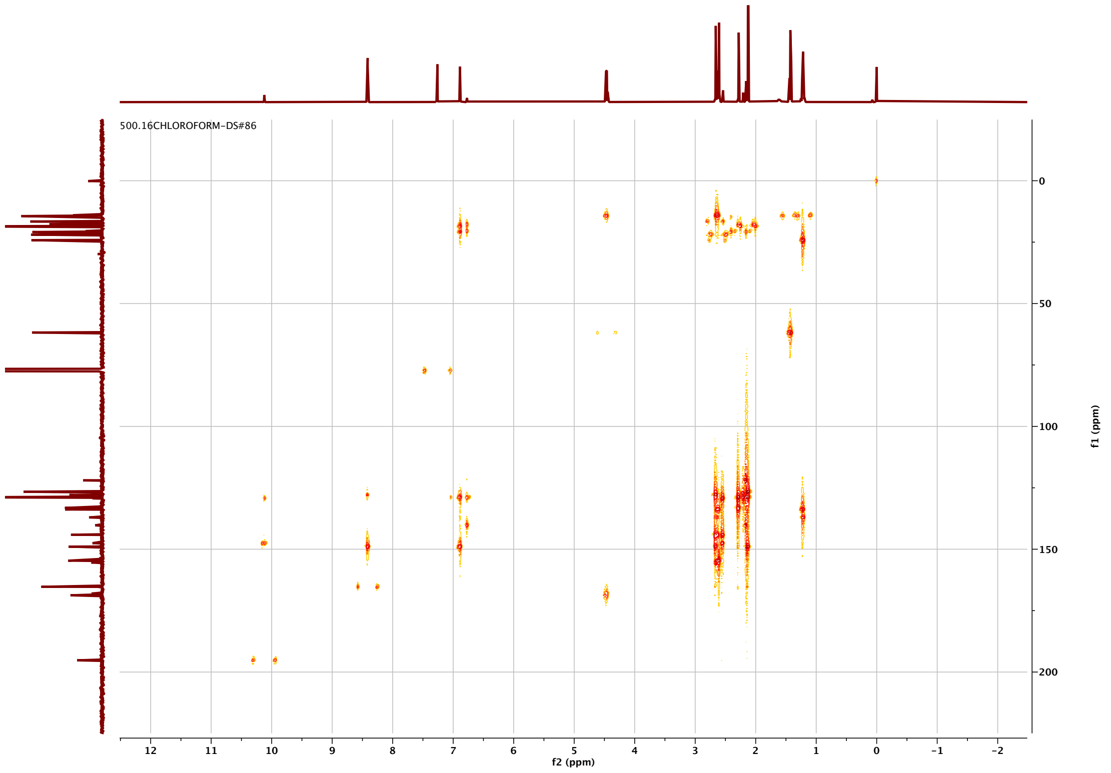

HMBC spectrum of ethyl 3-ethyl-6-formyl-2,5-dimethylisonicotinate (**2h**) (containing **2h'** and **2h''**).

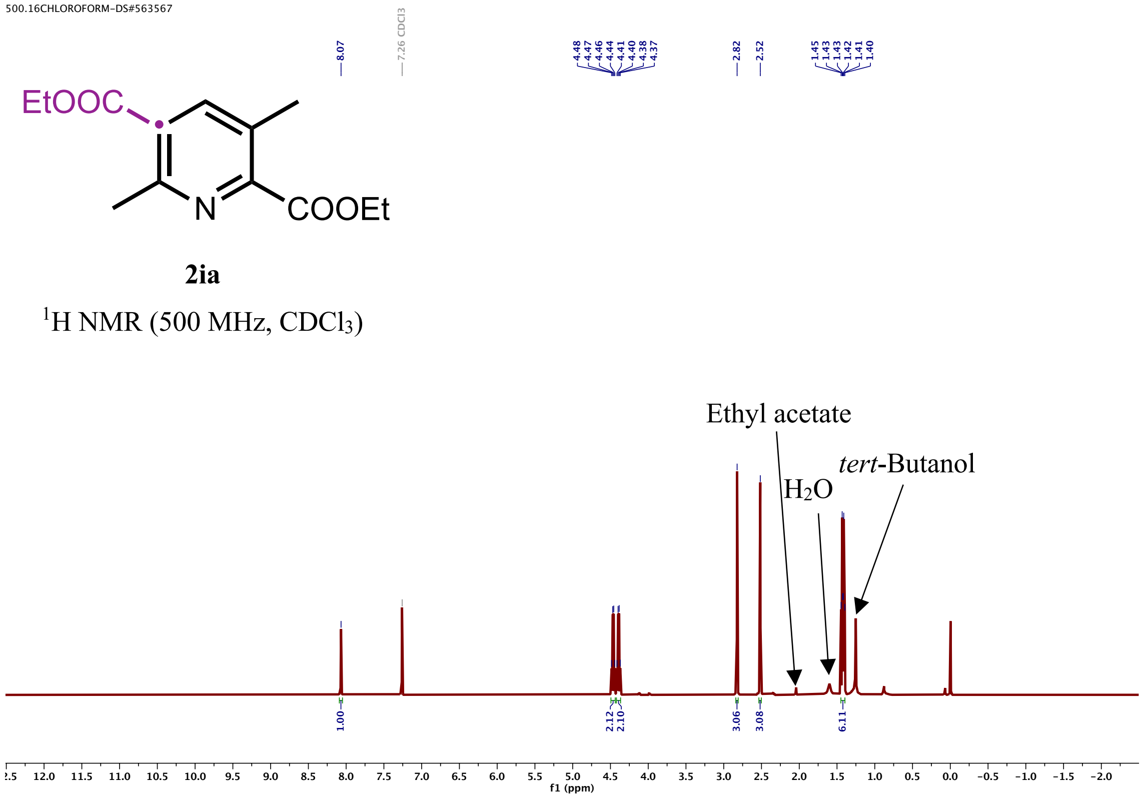

2ia

^{1}H NMR (500 MHz, $CDCl_3$)

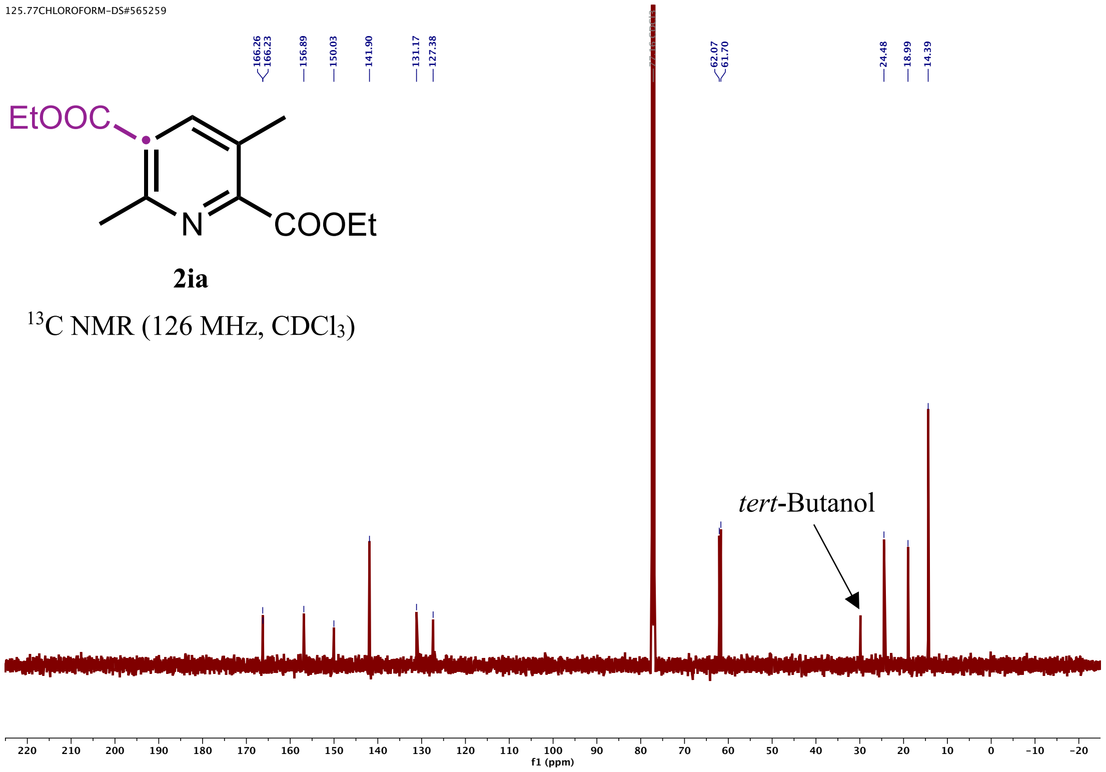

2ia

^{13}C NMR (126 MHz, $CDCl_3$)

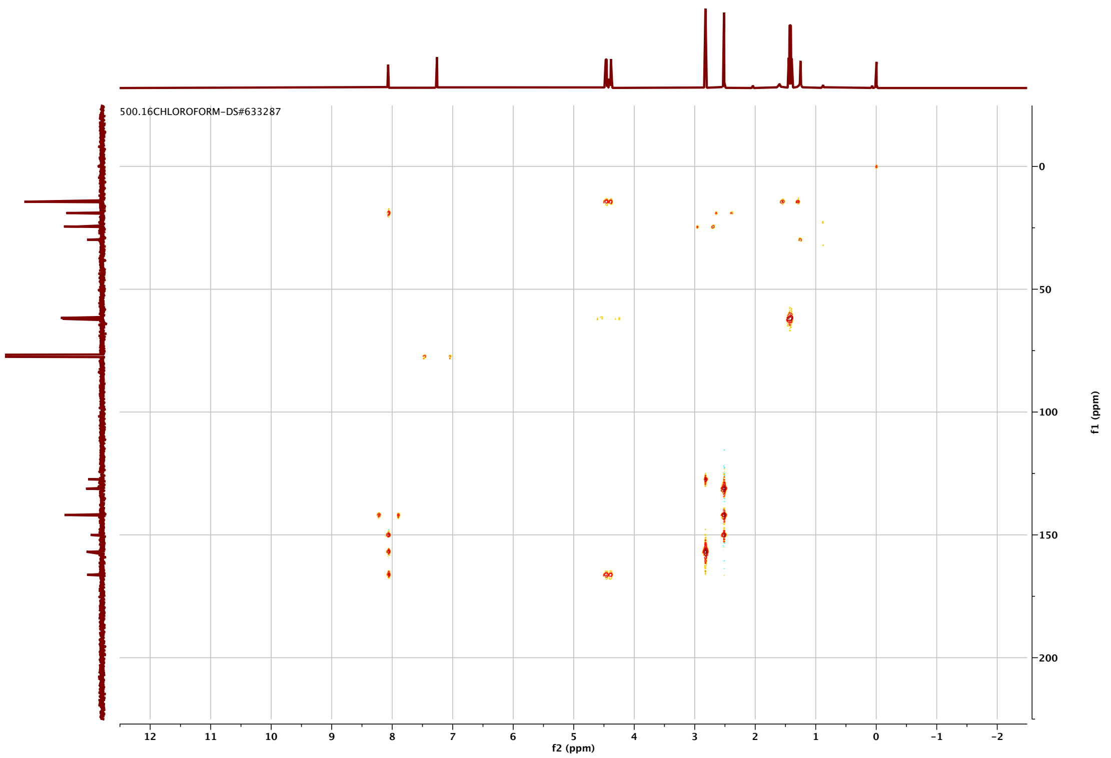

HMBC spectrum of diethyl 3,6-dimethylpyridine-2,5-dicarboxylate **(2ia)**.

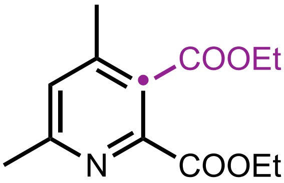

2ib

^{1}H NMR (500 MHz, $CDCl_3$)

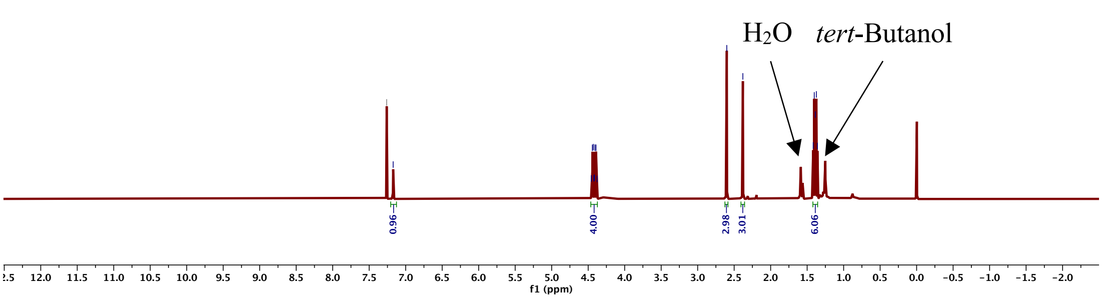

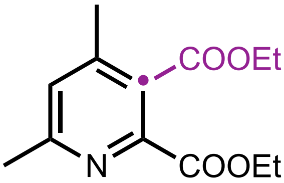

2ib

^{13}C NMR (126 MHz, $CDCl_3$)

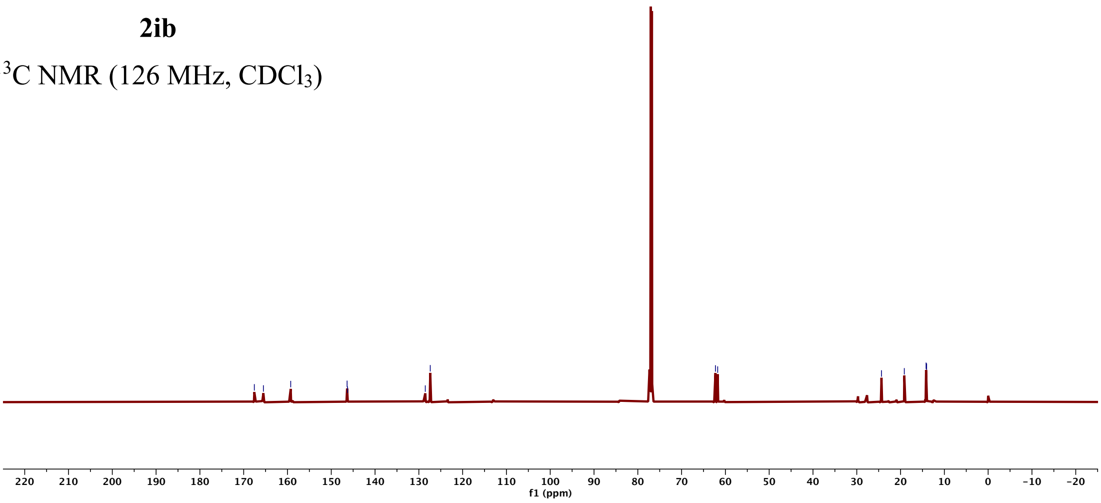

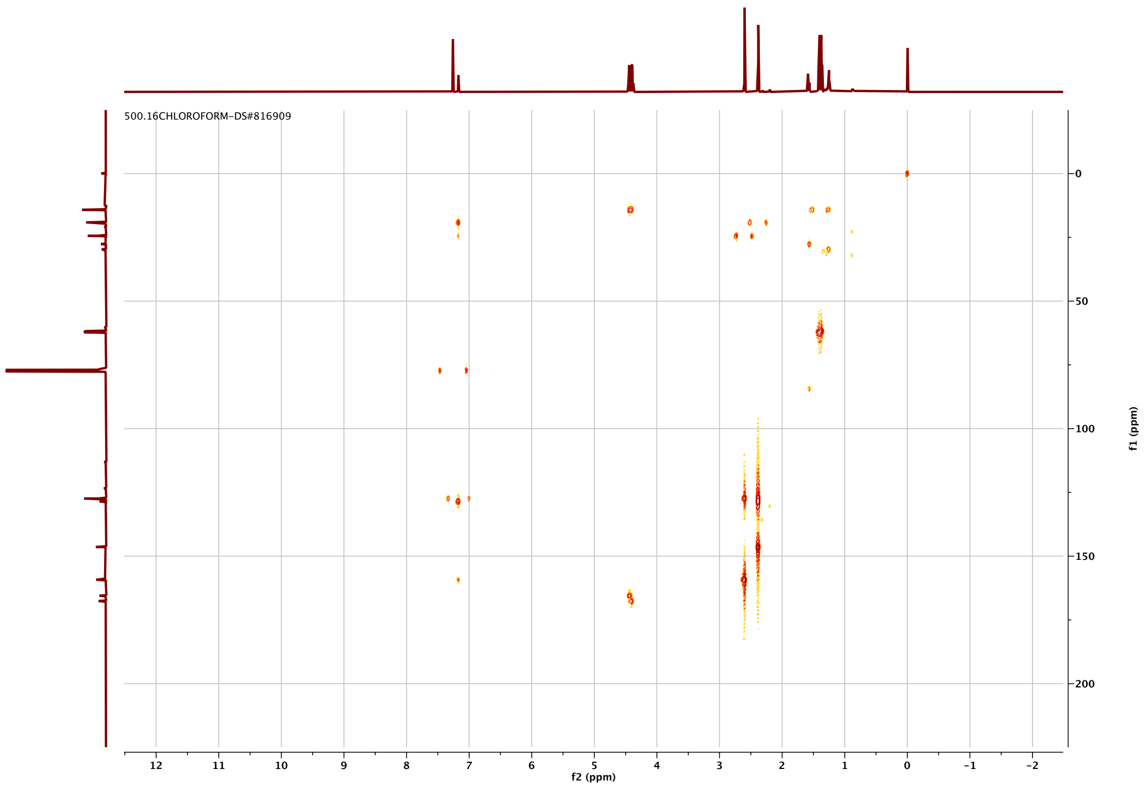

HMBC spectrum of diethyl 4,6-dimethylpyridine-2,3-dicarboxylate **(2ib)**.

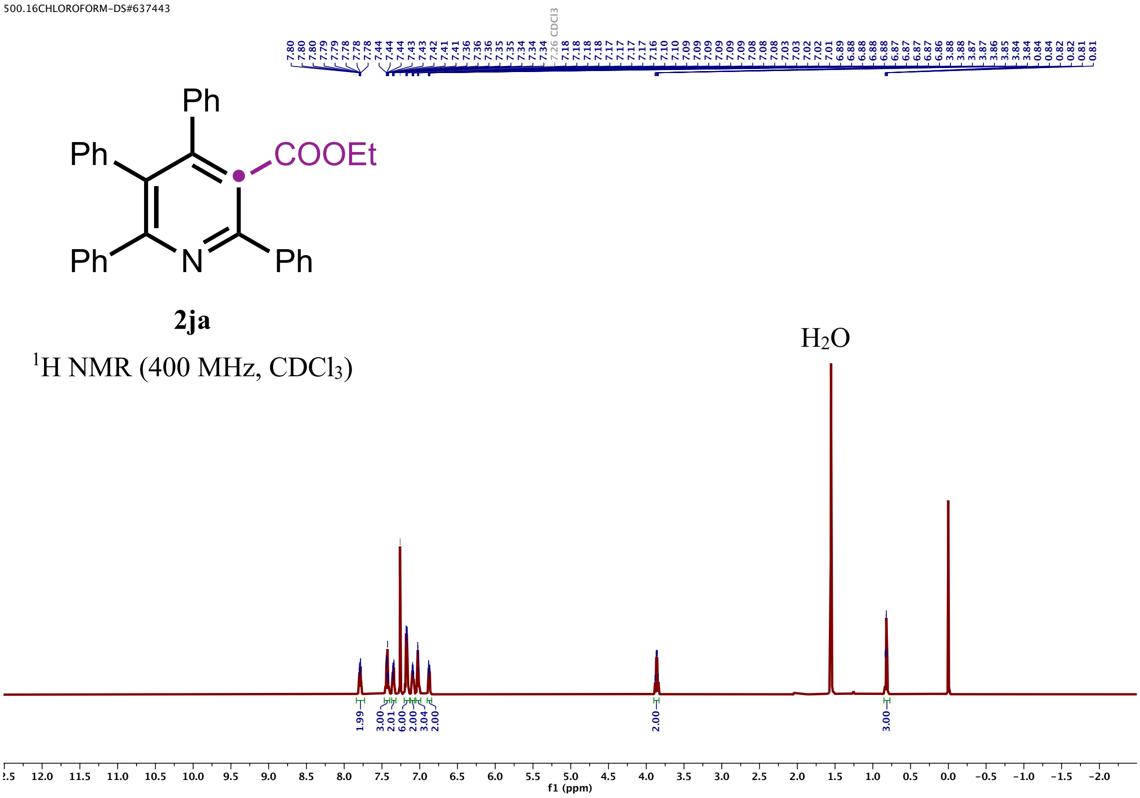

2ja

^{1}H NMR (400 MHz, $CDCl_3$)

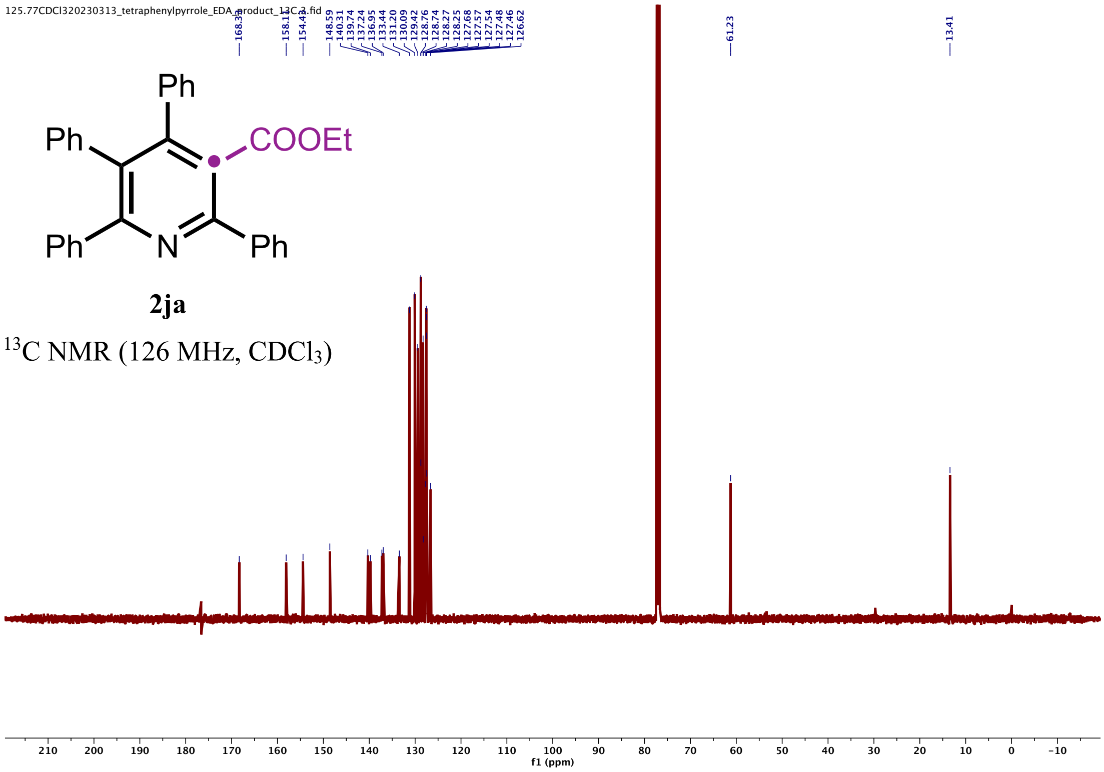

2ja

^{13}C NMR (126 MHz, $CDCl_3$)

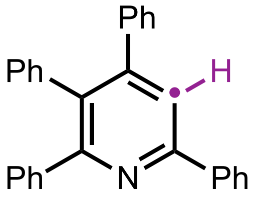

2jb

^{1}H NMR (500 MHz, $CDCl_3$)

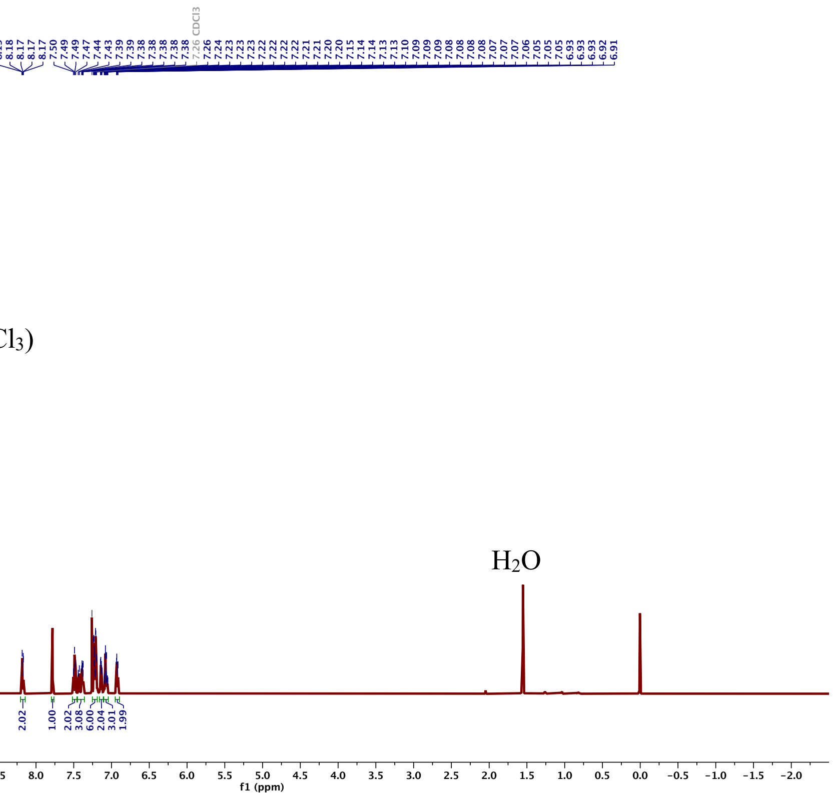

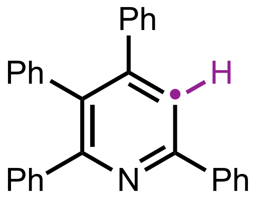

2jb

^{13}C NMR (126 MHz, $CDCl_3$)

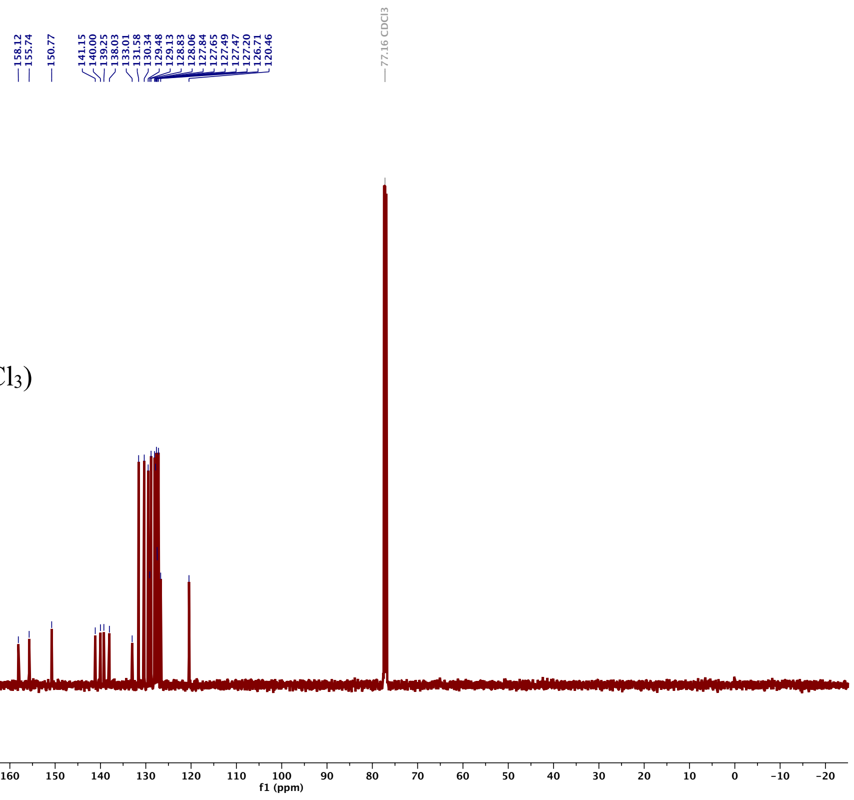

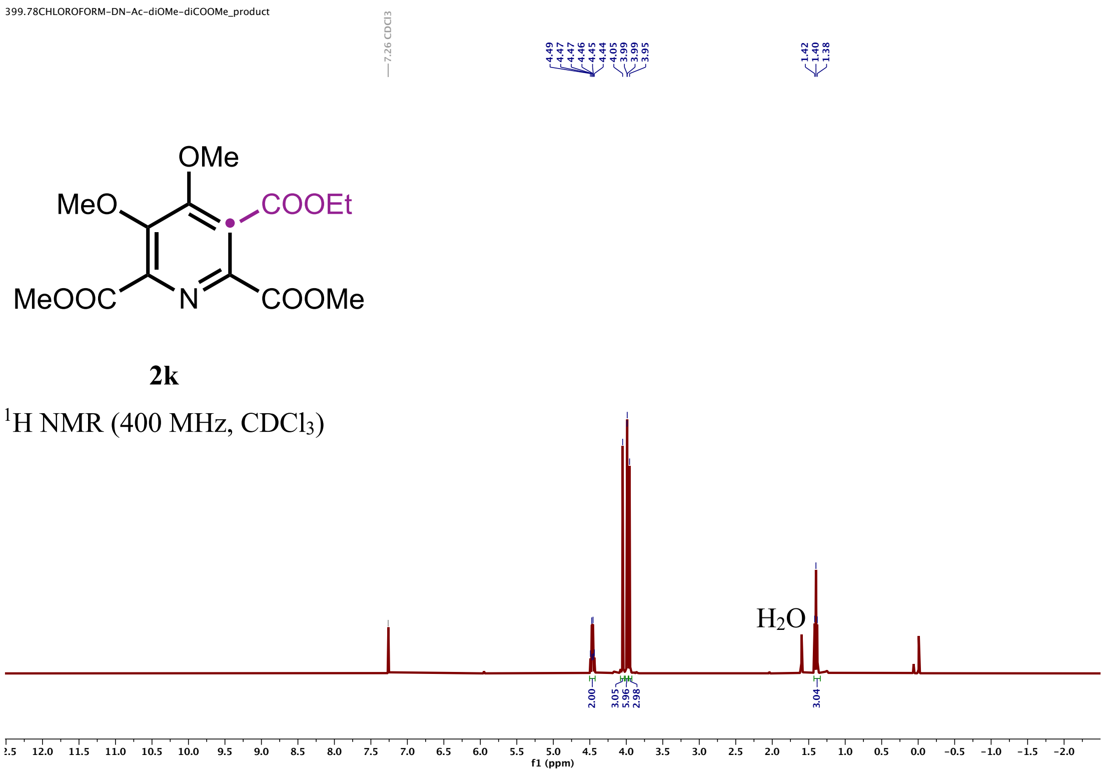

399.78CHLOROFORM-DN-Ac-diOMe-diCOOMe_product
7.26 CDCl3
4.49
4.47
4.47
4.46
4.45
4.44
4.05
3.99
3.99
3.95
1.42
1.40
1.38
OMe
MeO
COOEt
MeOOC
N
COOMe
2k
¹H NMR (400 MHz, CDCl₃)
H₂O
2.00
3.05
5.96
2.98
3.04
f1 (ppm)

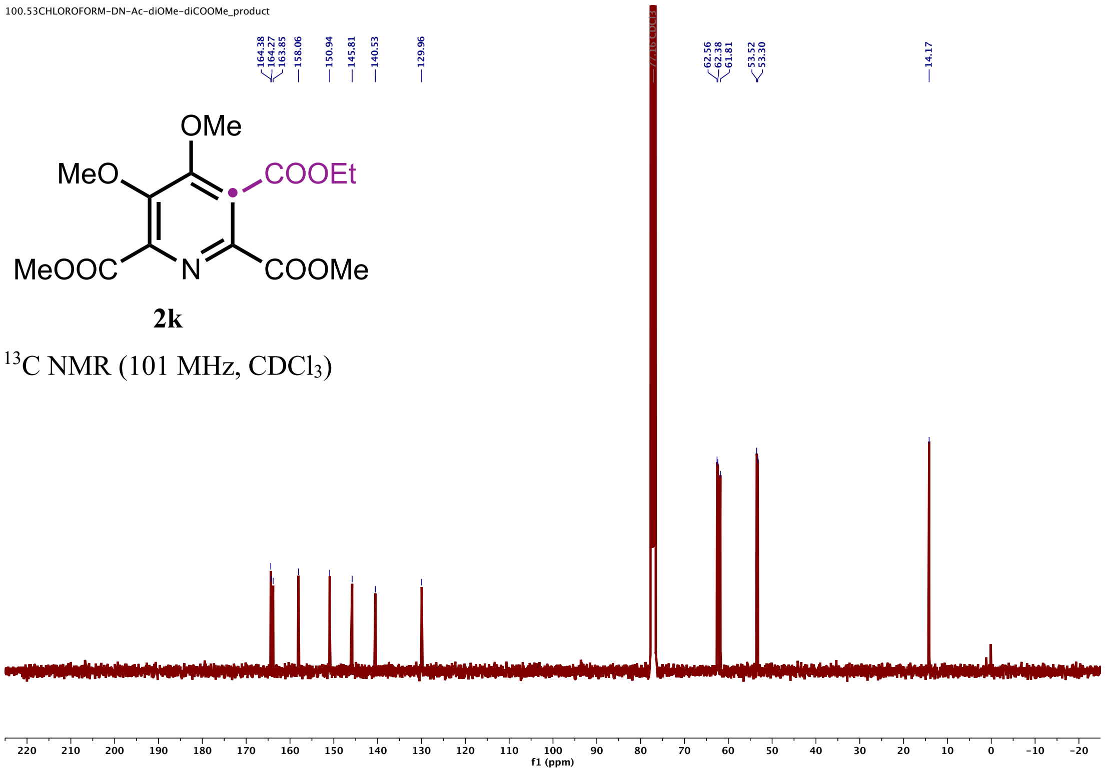

100.53CHLOROFORM-DN-Ac-diOMe-diCOOMe_product
164.38
164.27
163.85
158.06
150.94
145.81
140.53
129.96
62.56
62.38
61.81
53.52
53.30
14.17
OMe
MeO
COOEt
MeOOC
N
COOMe
2k
¹³C NMR (101 MHz, CDCl₃)
f1 (ppm)

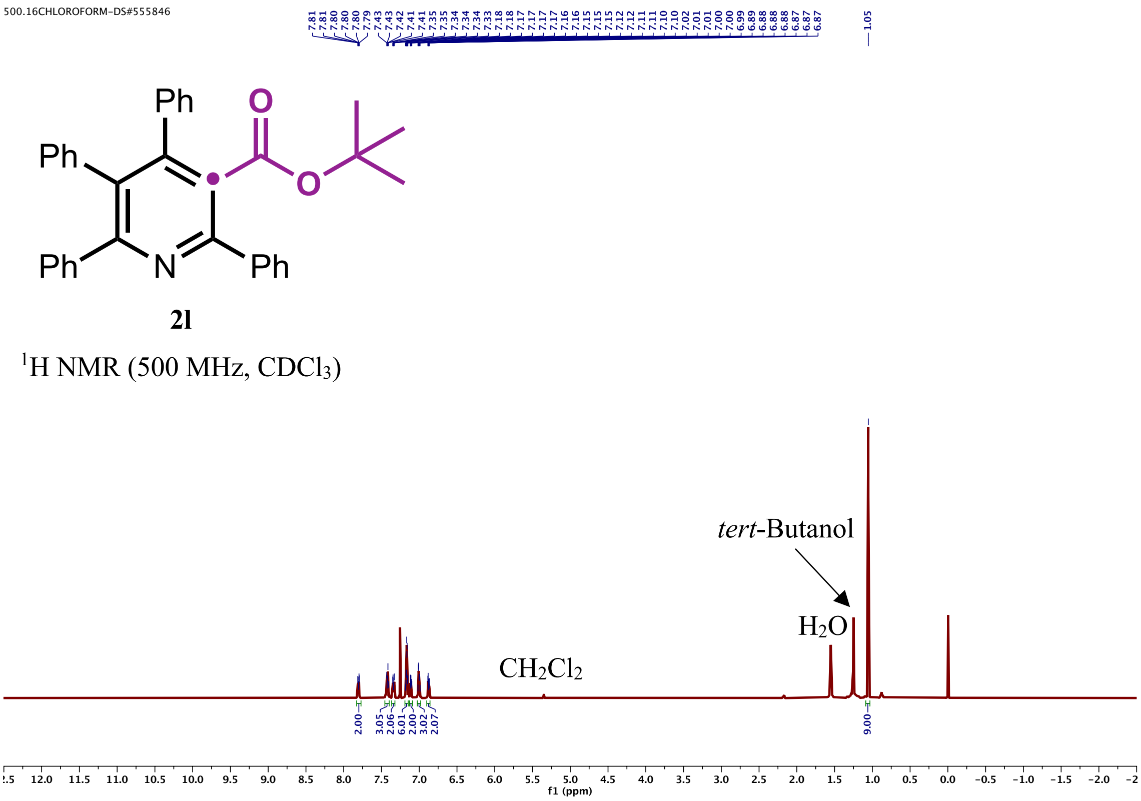

2l

^{1}H NMR (500 MHz, $CDCl_3$)

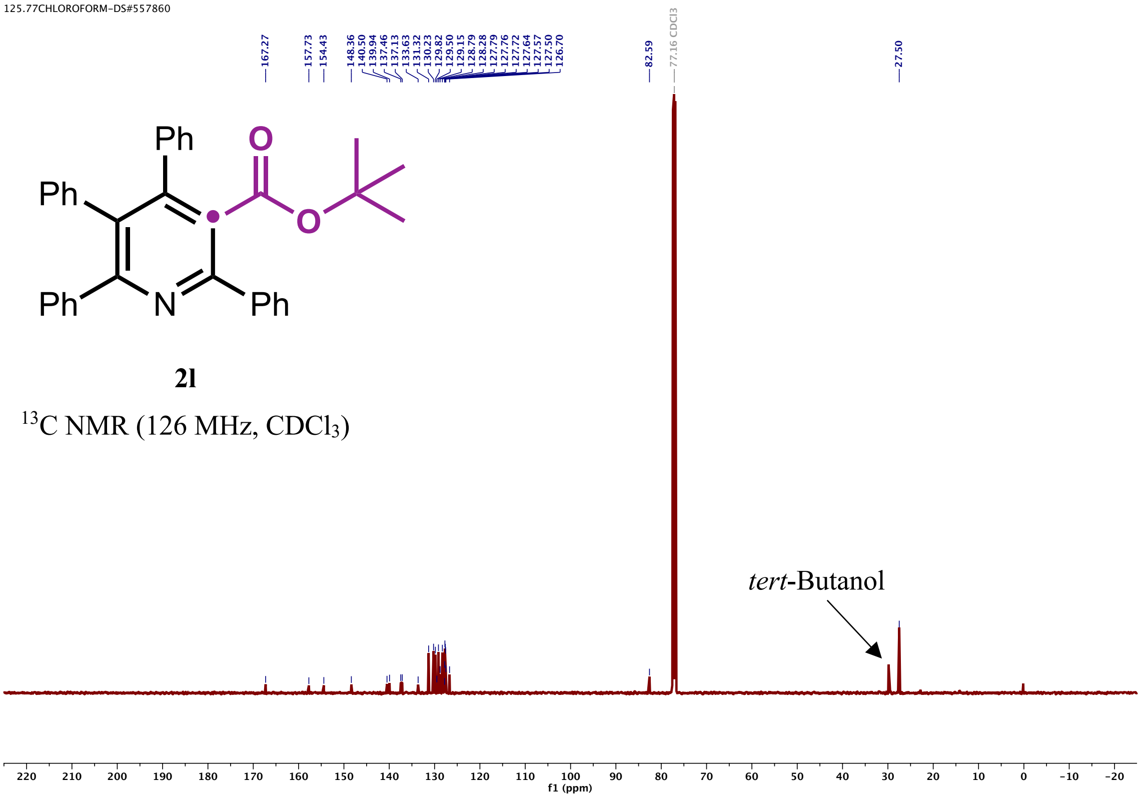

2l

^{13}C NMR (126 MHz, $CDCl_3$)

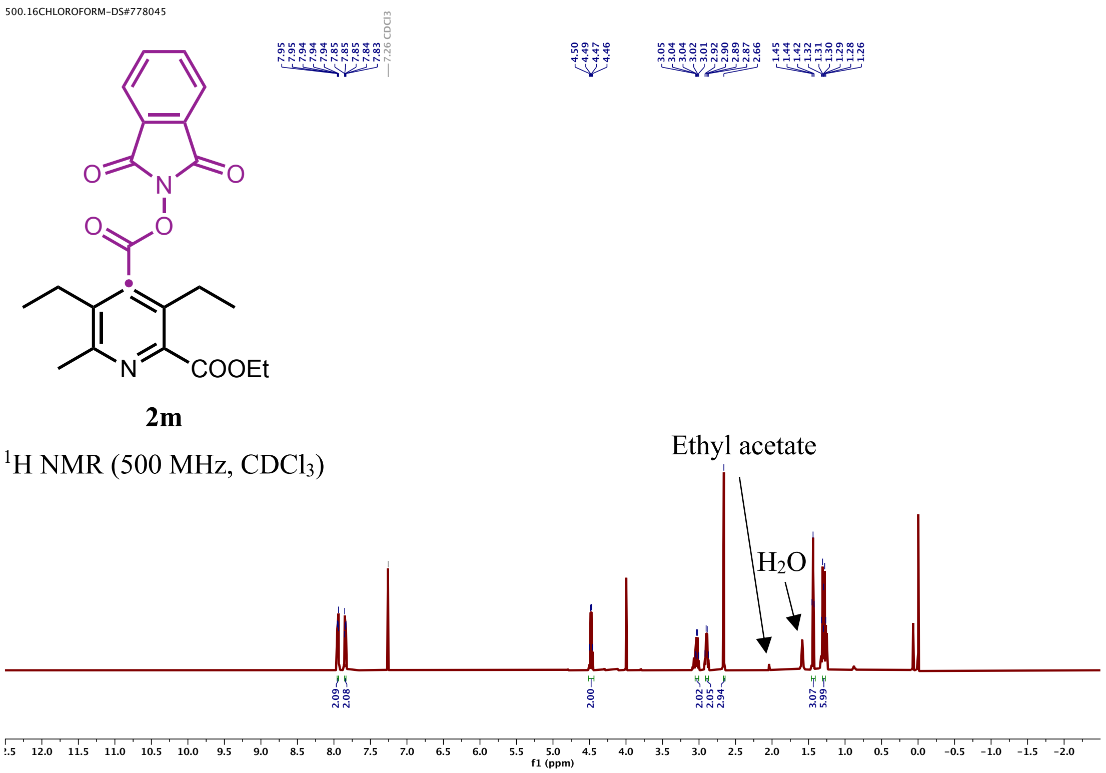

2m

^1H NMR (500 MHz, $CDCl_3$)

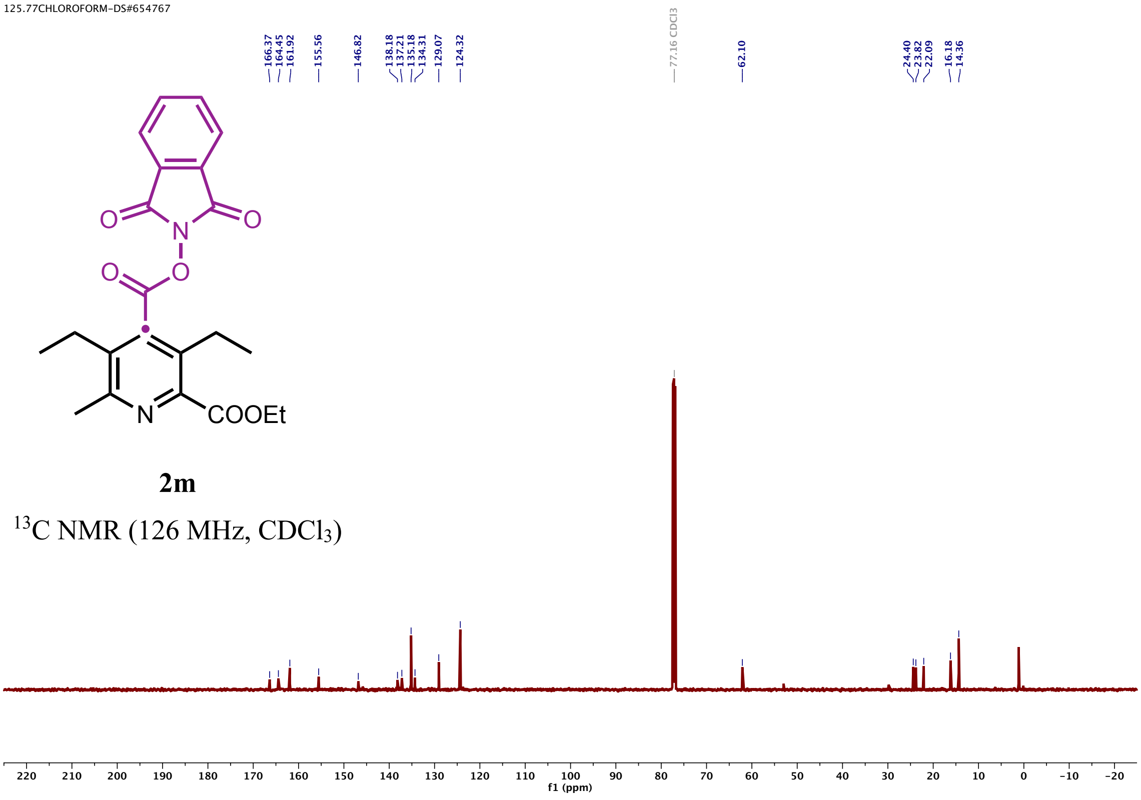

2m

^{13}C NMR (126 MHz, $CDCl_3$)

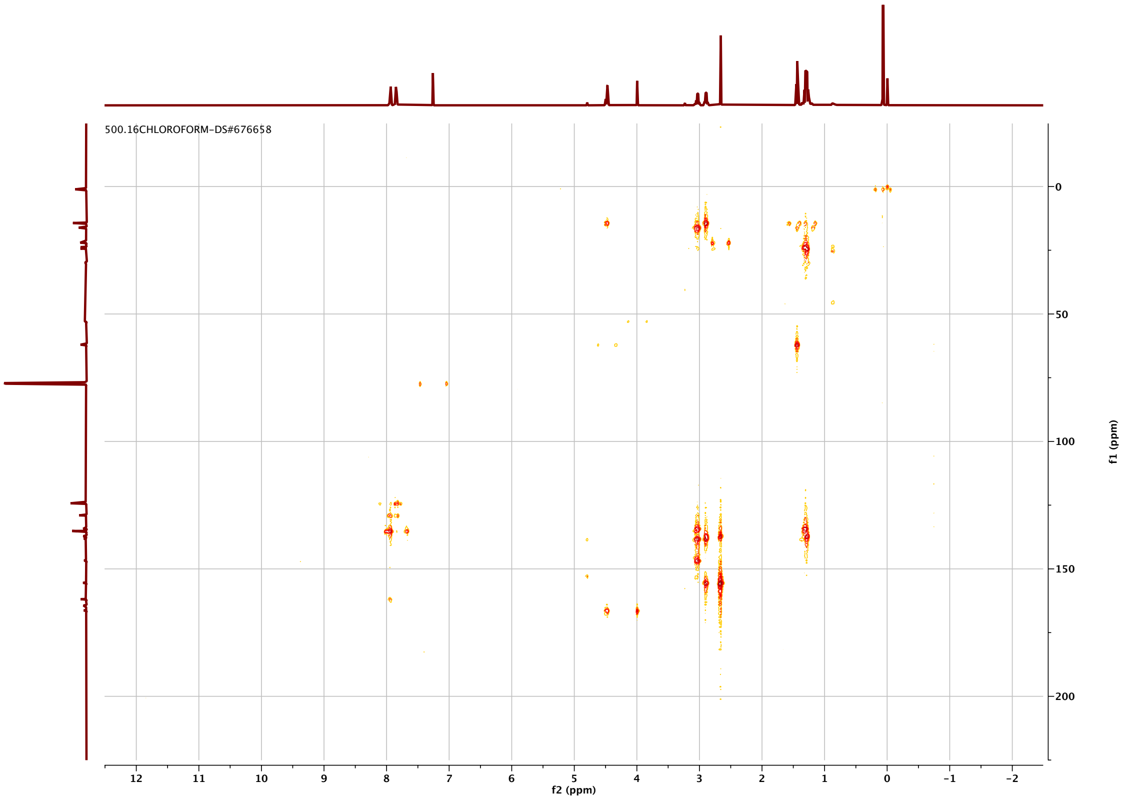

HMBC spectrum of 4-(1,3-dioxo-2,3-dihydro-1*H*-inden-2-yl) 2-ethyl 3,5-diethyl-6-methylpyridine-2,4-dicarboxylate **(2m)**

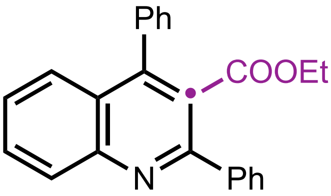

2n

^{1}H NMR (500 MHz, $CDCl_3$)

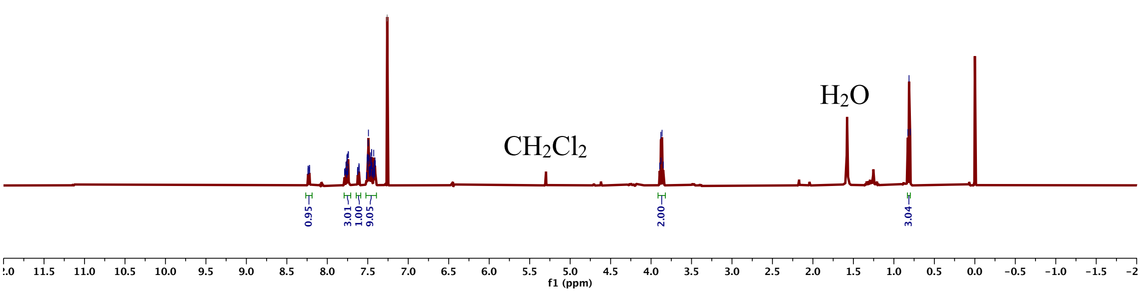

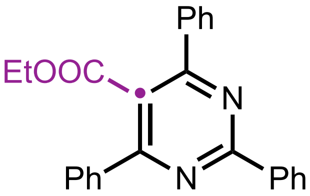

2o

^{1}H NMR (400 MHz, $CDCl_3$)

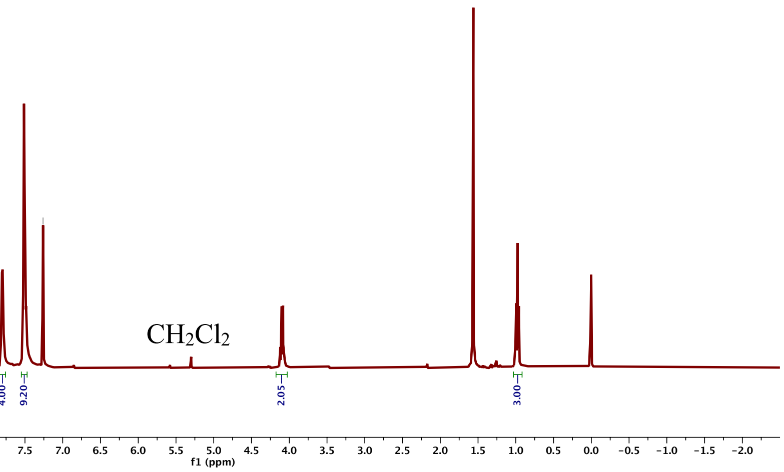

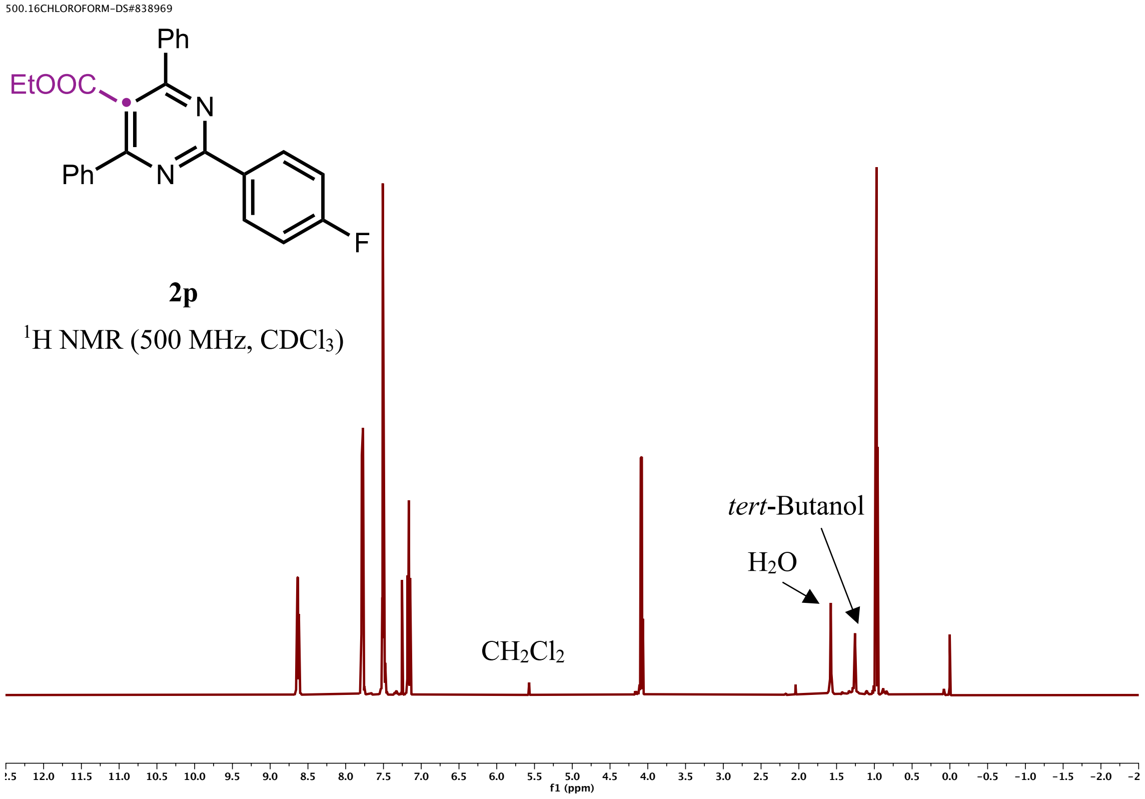
500.16CHLOROFORM-DS#838969
Ph
EtOOC
N
Ph
N
F
2p
1H NMR (500 MHz, CDCl3)
tert-Butanol
H2O
CH2Cl2
f1 (ppm)

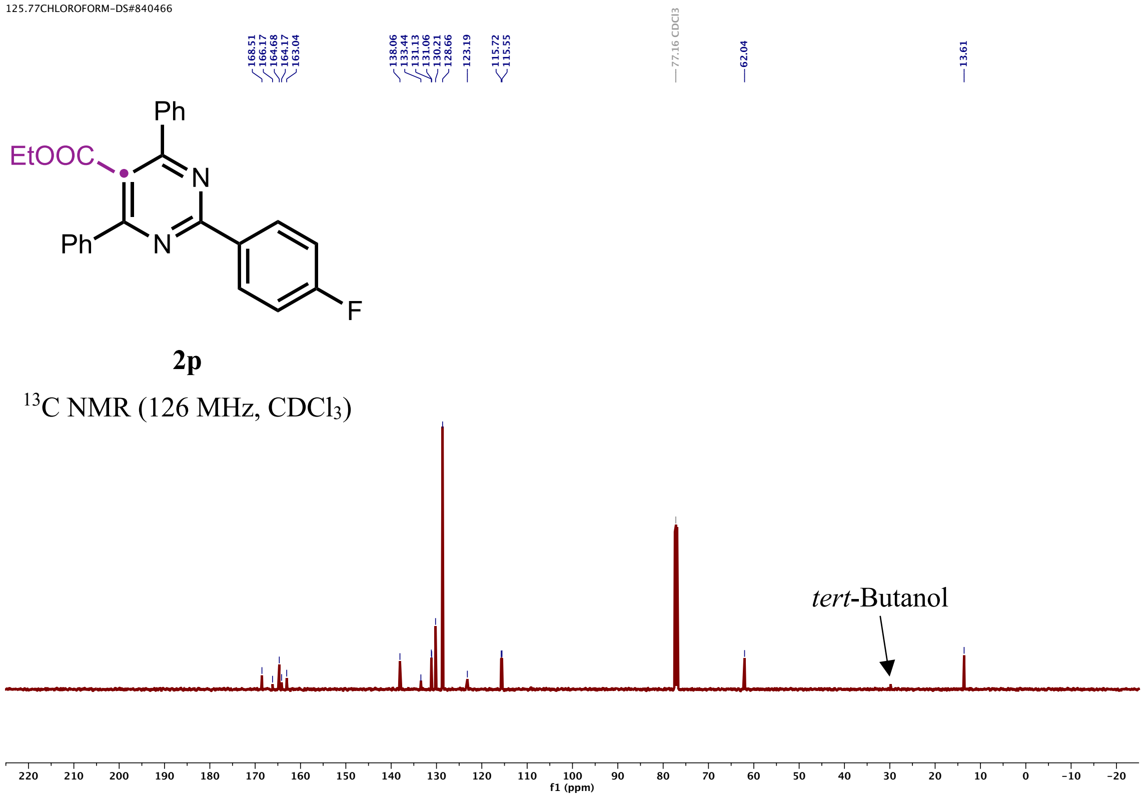
125.77CHLOROFORM-DS#840466
168.51
166.17
164.68
164.17
163.04
138.06
133.44
131.13
131.06
130.21
128.66
123.19
115.72
115.55
77.16 CDCl3
62.04
13.61
Ph
EtOOC
N
Ph
N
F
2p
13C NMR (126 MHz, CDCl3)
tert-Butanol
f1 (ppm)

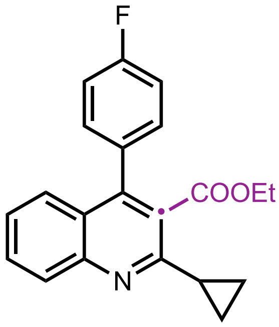

2q

^{1}H NMR (500 MHz, $CDCl_3$)

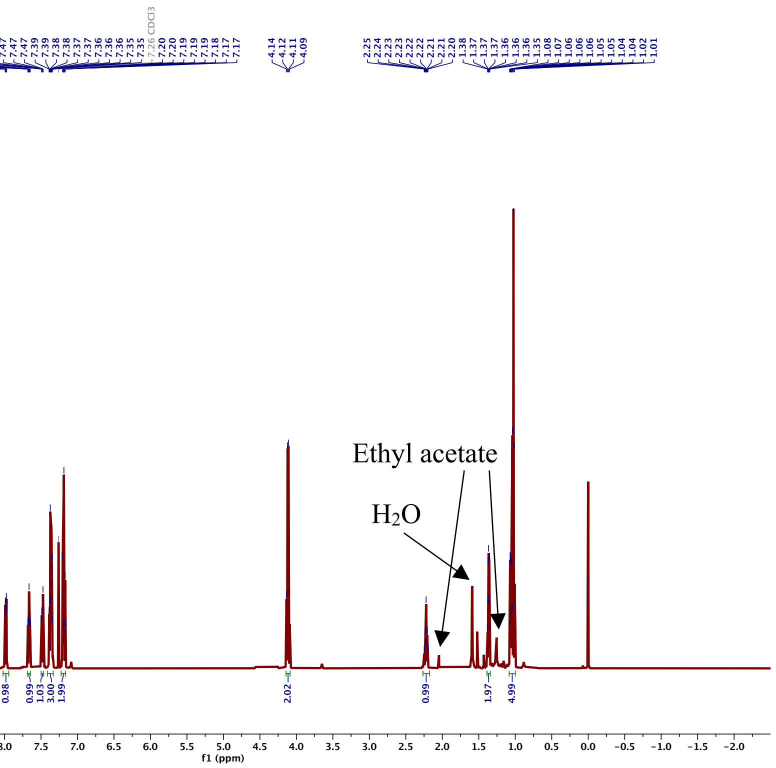

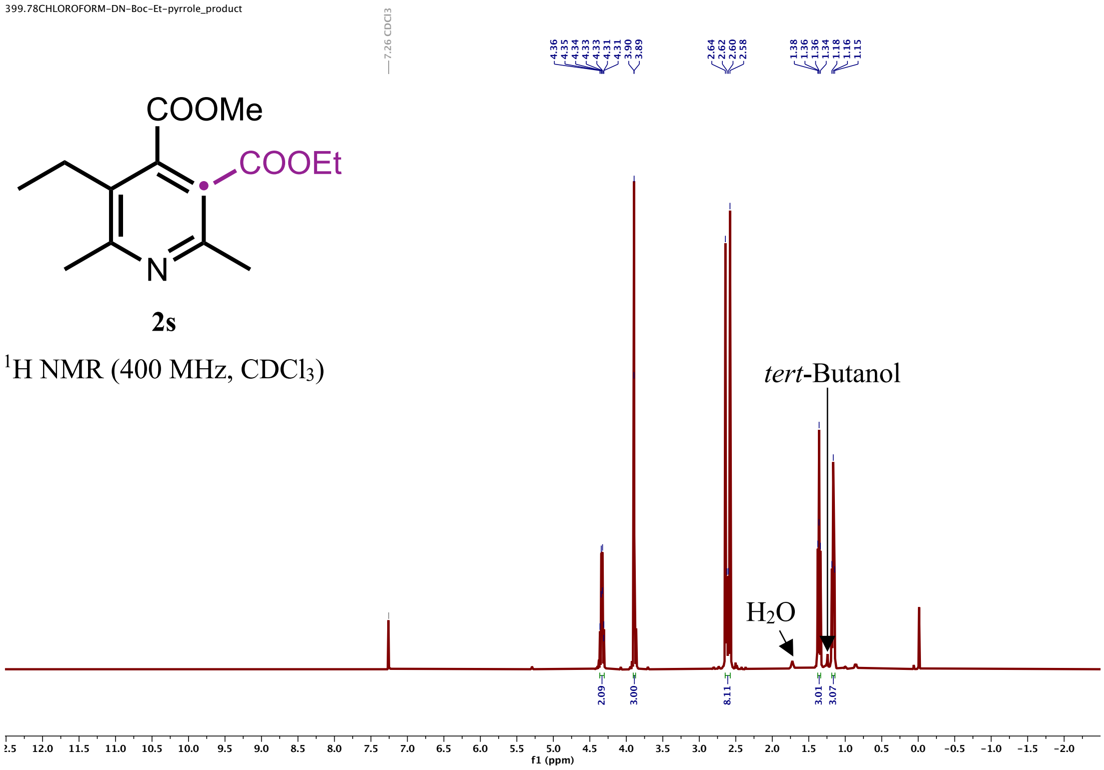
399.78CHLOROFORM-DN-Boc-Et-pyrrole_product
COOMe
COOEt
N
2s
1H NMR (400 MHz, CDCl3)
tert-Butanol
H2O
7.26 CDCl3
4.36 4.35 4.34 4.33 4.33 4.31 4.31 3.90 3.89
2.64 2.62 2.60 2.58
1.38 1.36 1.36 1.34 1.18 1.16 1.15
2.09 3.00 8.11 3.01 3.07
f1 (ppm)

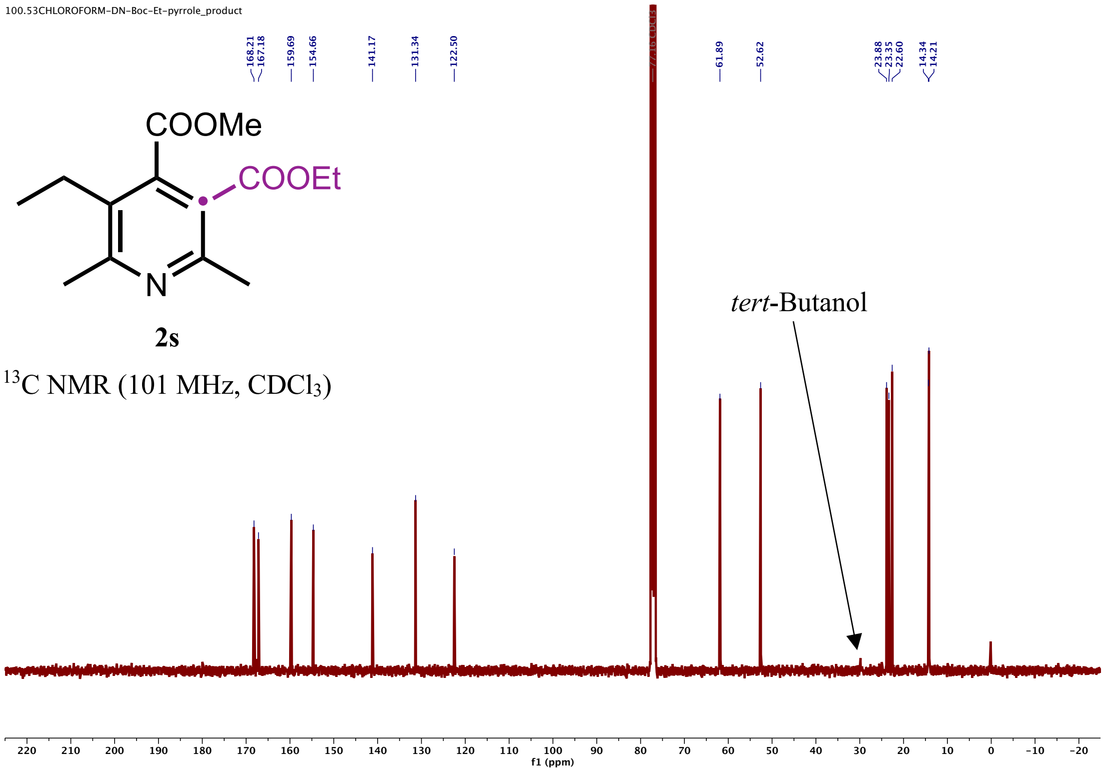
100.53CHLOROFORM-DN-Boc-Et-pyrrole_product
COOMe
COOEt
N
2s
13C NMR (101 MHz, CDCl3)
tert-Butanol
168.21 167.18 159.69 154.66 141.17 131.34 122.50
61.89 52.62
23.88 23.35 22.60 14.34 14.21
f1 (ppm)

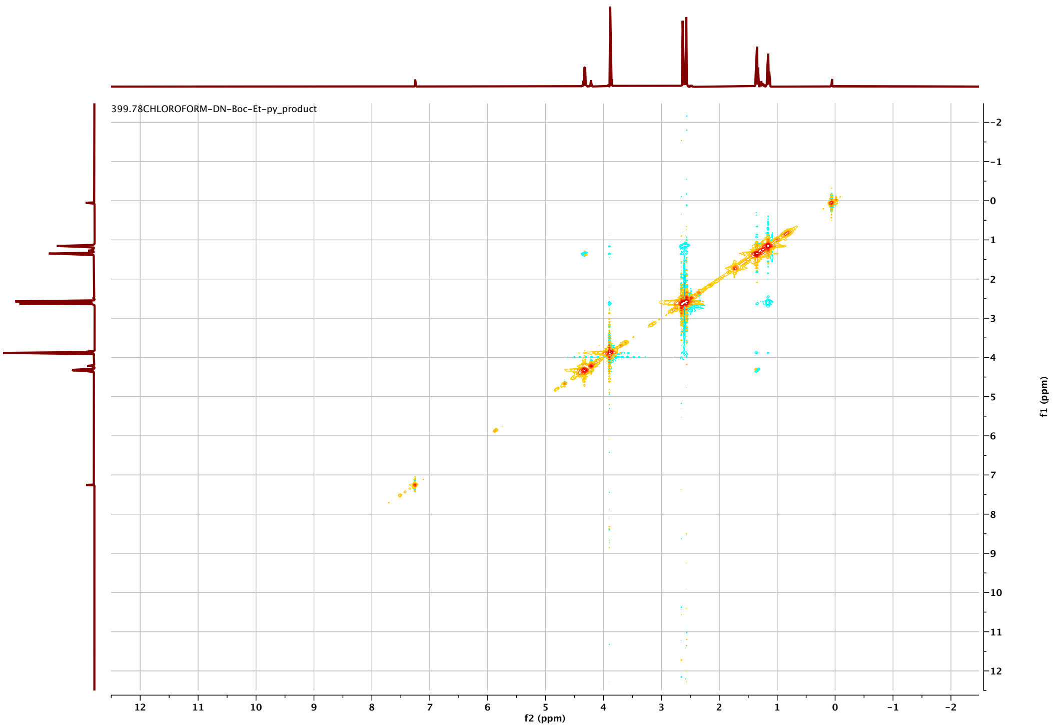

NOESY spectrum of 3-ethyl 4-methyl 5-ethyl-2,6-dimethylpyridine-3,4-dicarboxylate **(2s)**

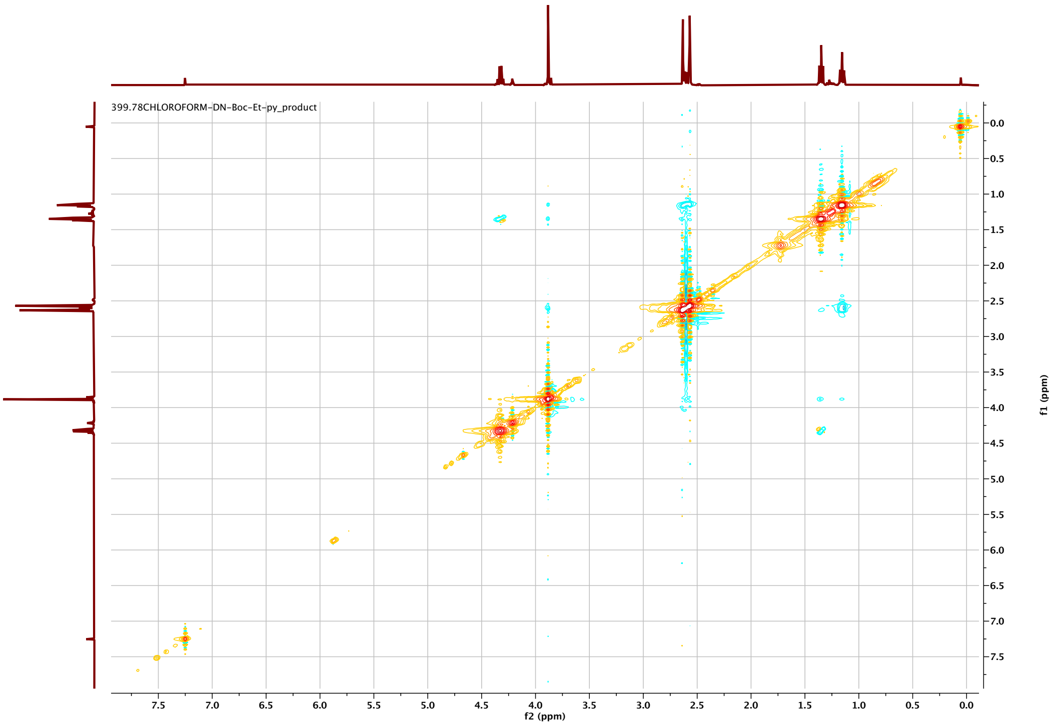

Enlarged NOESY spectrum of 3-ethyl 4-methyl 5-ethyl-2,6-dimethylpyridine-3,4-dicarboxylate **(2s)**

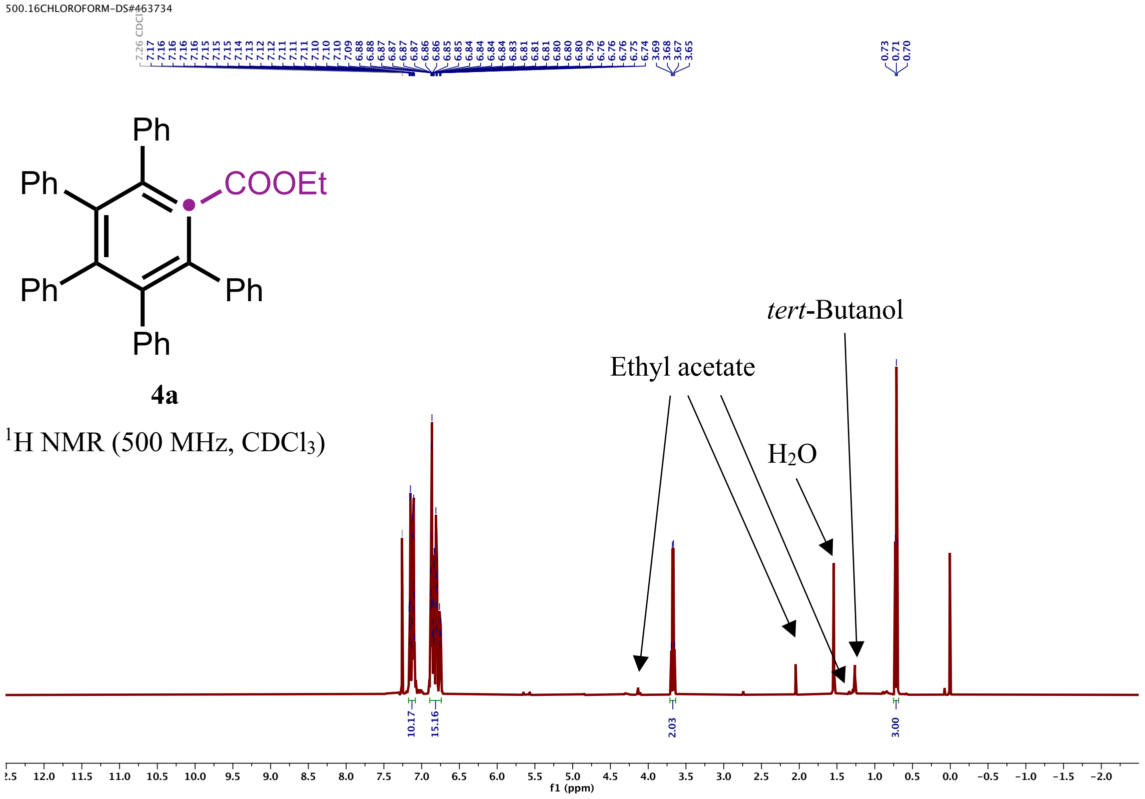

4a

^{1}H NMR (500 MHz, $CDCl_3$)

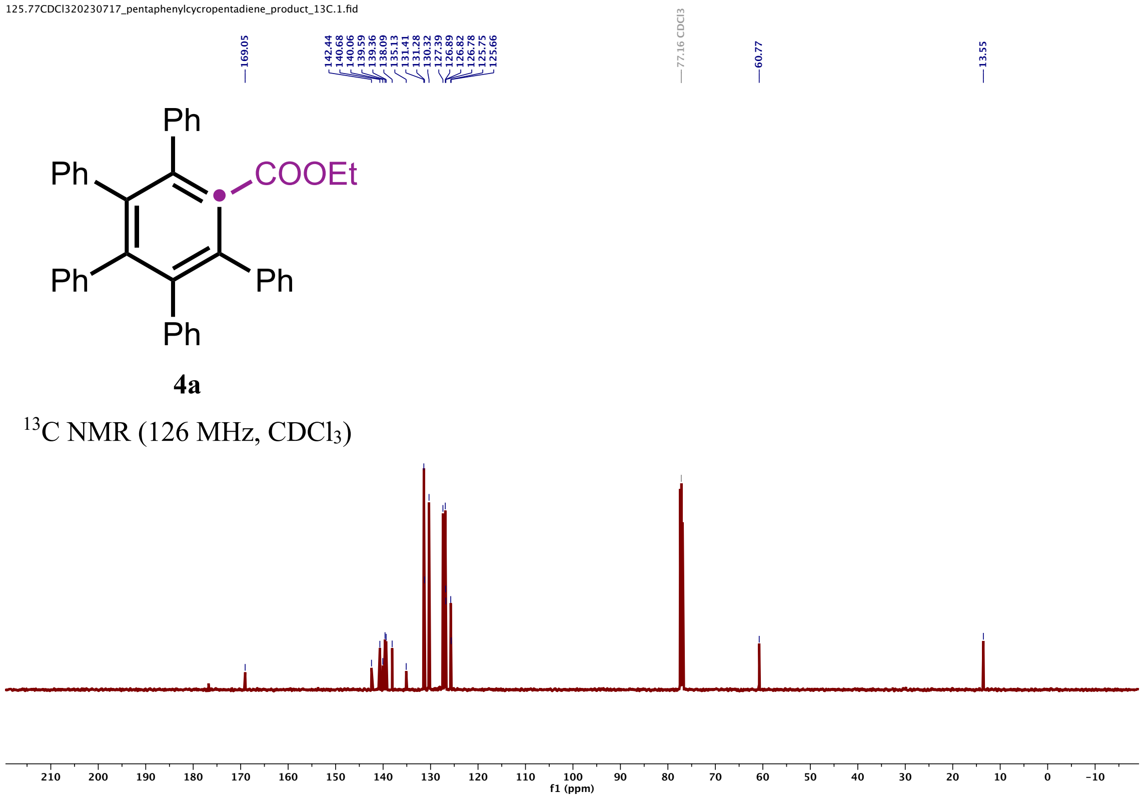

4a

^{13}C NMR (126 MHz, $CDCl_3$)

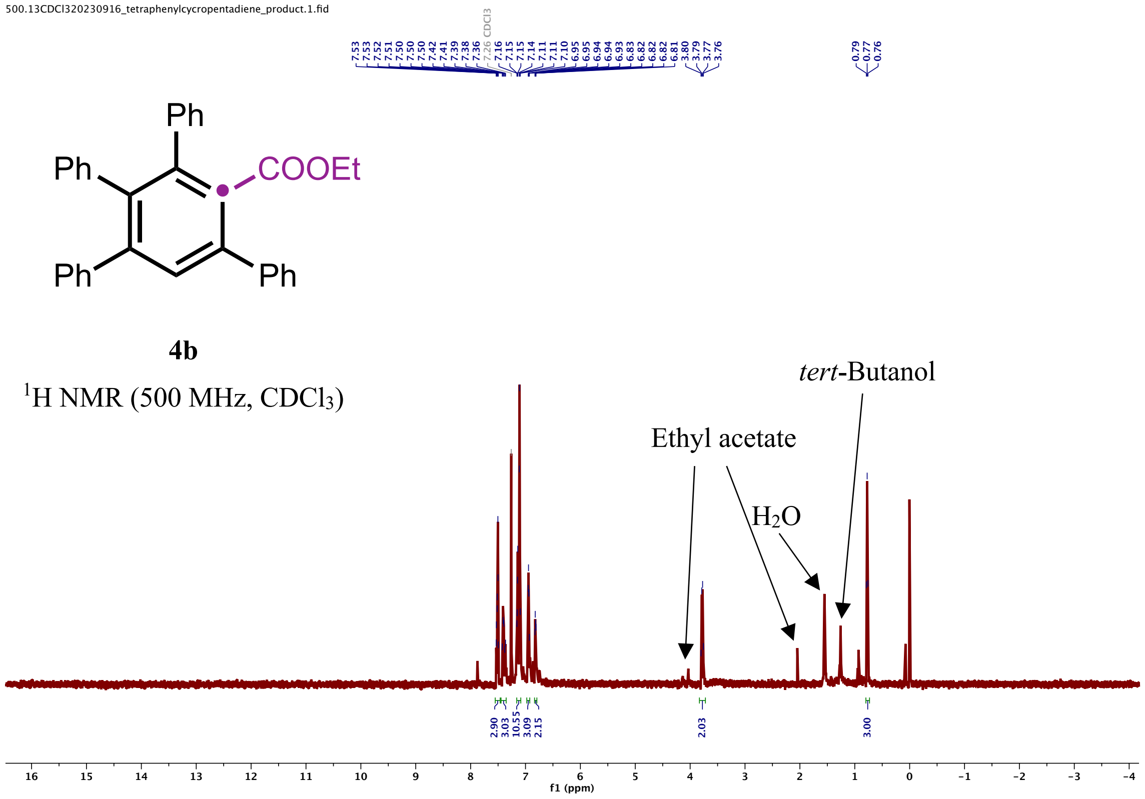

500.13CDCl320230916_tetraphenylcycropentadiene_product.1.fid
7.53 7.53 7.52 7.51 7.50 7.50 7.50 7.42 7.41 7.39 7.38 7.36 7.26 CDCl3 7.16 7.15 7.15 7.14 7.11 7.11 7.10 6.95 6.95 6.94 6.94 6.93 6.83 6.82 6.82 6.82 6.81 3.80 3.79 3.77 3.76
0.79 0.77 0.76
Ph
Ph
COOEt
Ph
Ph
4b
^{1}H NMR (500 MHz, CDCl$_3$)
tert-Butanol
Ethyl acetate
H_2O
2.90 3.03 10.55 3.09 2.15
2.03
3.00
f1 (ppm)

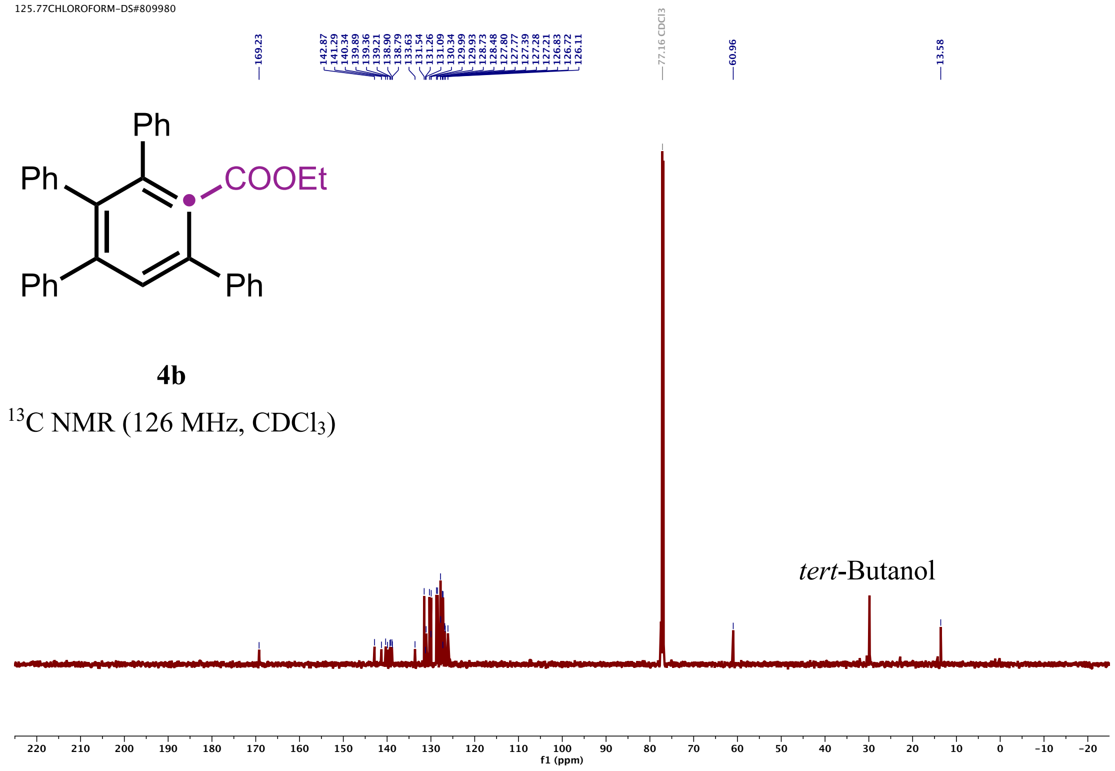

125.77CHLOROFORM-DS#809980
169.23
142.87 141.29 140.34 139.89 139.36 139.21 138.90 138.79 133.63 131.54 131.26 131.09 130.34 129.99 129.93 128.73 128.48 127.80 127.77 127.39 127.28 127.21 126.83 126.72 126.11
77.16 CDCl3
60.96
13.58
Ph
Ph
COOEt
Ph
Ph
4b
^{13}C NMR (126 MHz, CDCl$_3$)
tert-Butanol
f1 (ppm)

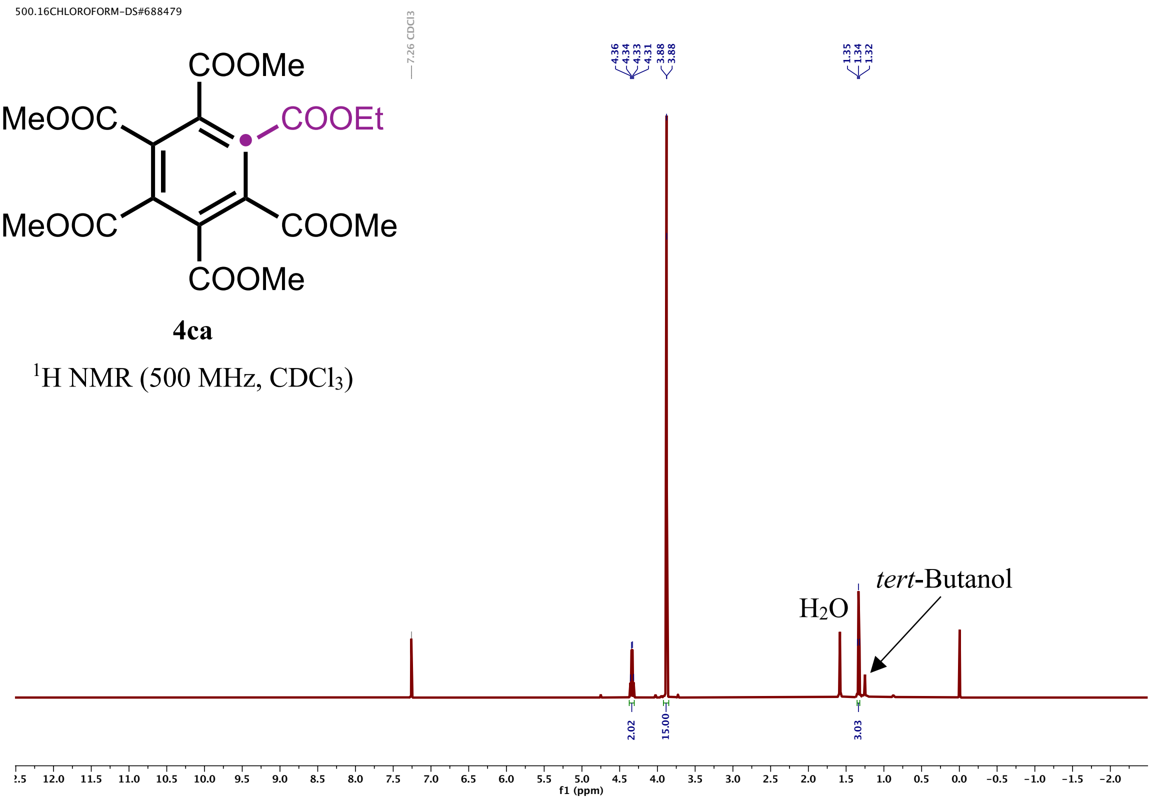
500.16CHLOROFORM-DS#688479
COOMe
MeOOC
COOEt
MeOOC
COOMe
COOMe
4ca
1H NMR (500 MHz, CDCl3)
7.26 CDCl3
4.36
4.34
4.33
4.31
3.88
3.88
1.35
1.34
1.32
H2O
tert-Butanol
2.02
15.00
3.03
f1 (ppm)

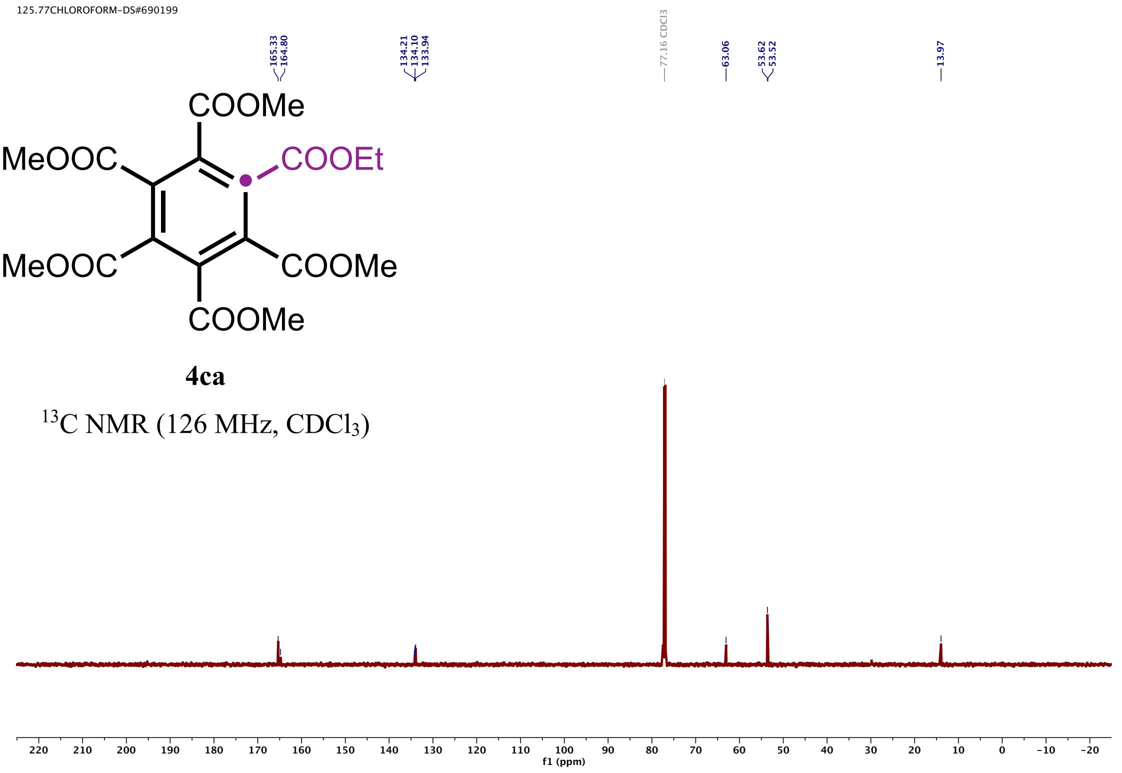
125.77CHLOROFORM-DS#690199
165.33
164.80
134.21
134.10
133.94
77.16 CDCl3
63.06
53.62
53.52
13.97
COOMe
MeOOC
COOEt
MeOOC
COOMe
COOMe
4ca
13C NMR (126 MHz, CDCl3)
f1 (ppm)

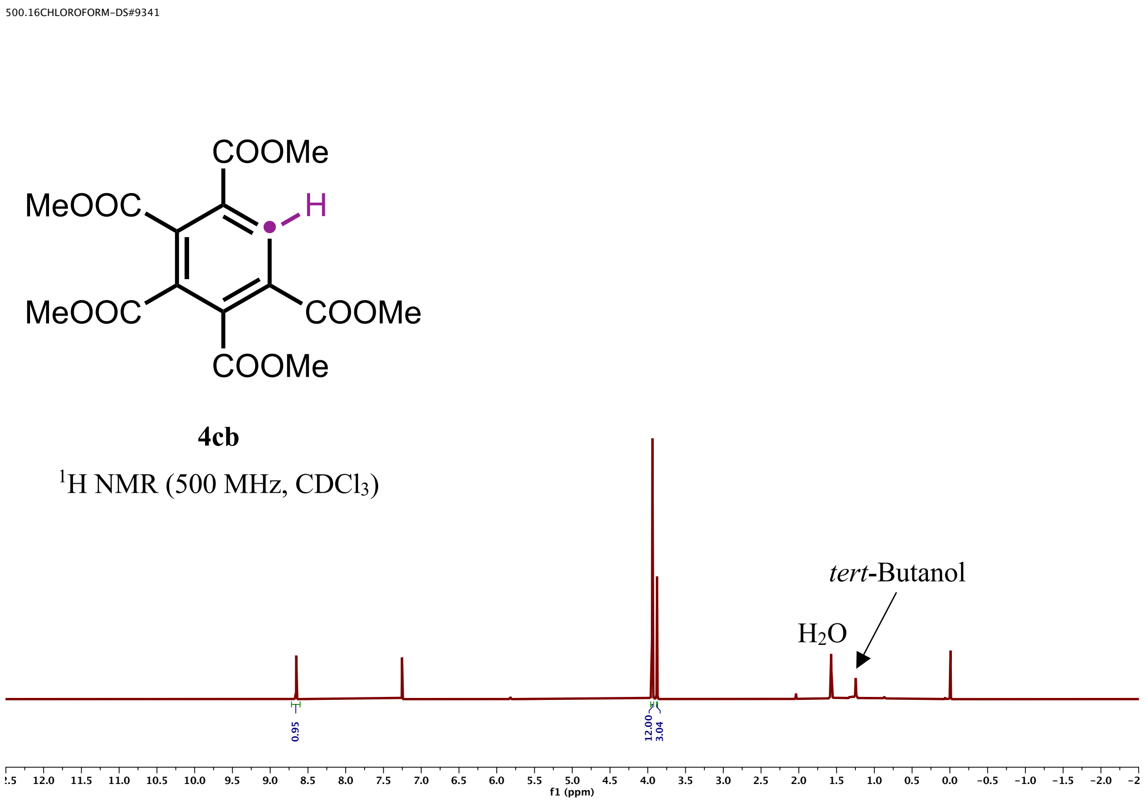

4cb

^{1}H NMR (500 MHz, $CDCl_3$)

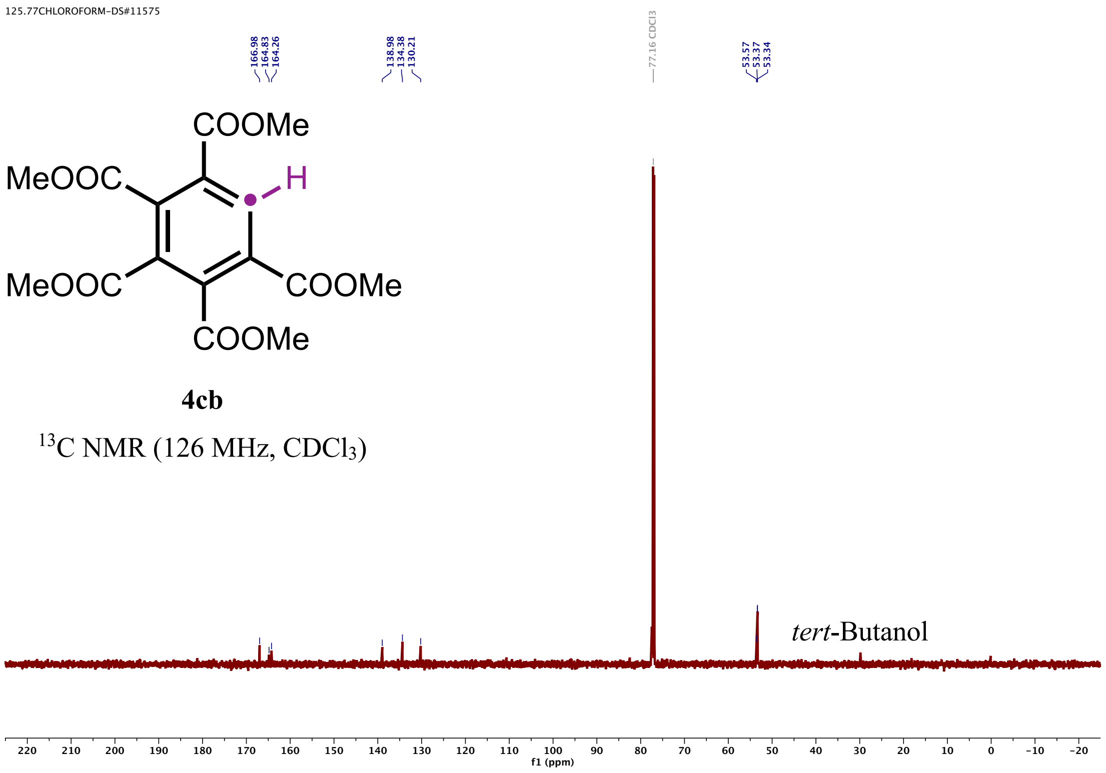

4cb

^{13}C NMR (126 MHz, $CDCl_3$)

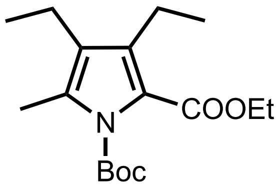

1a-Boc

^{1}H NMR (500 MHz, $CDCl_3$)

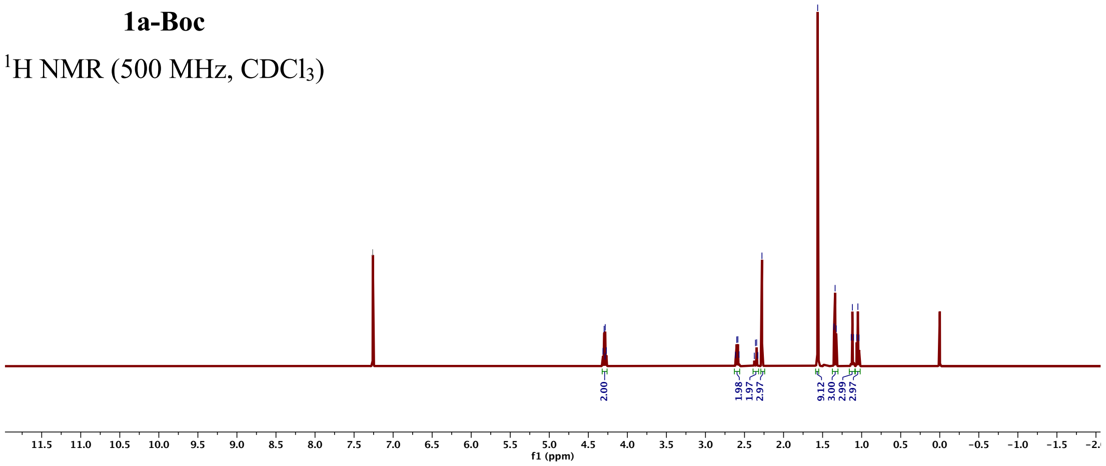

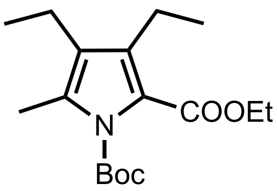

1a-Boc

^{13}C NMR (126 MHz, $CDCl_3$)

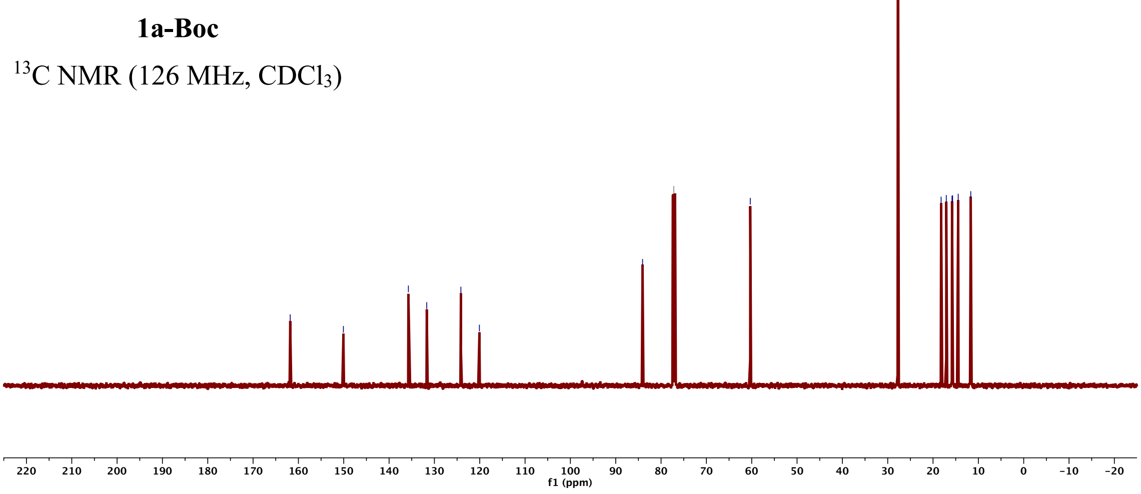

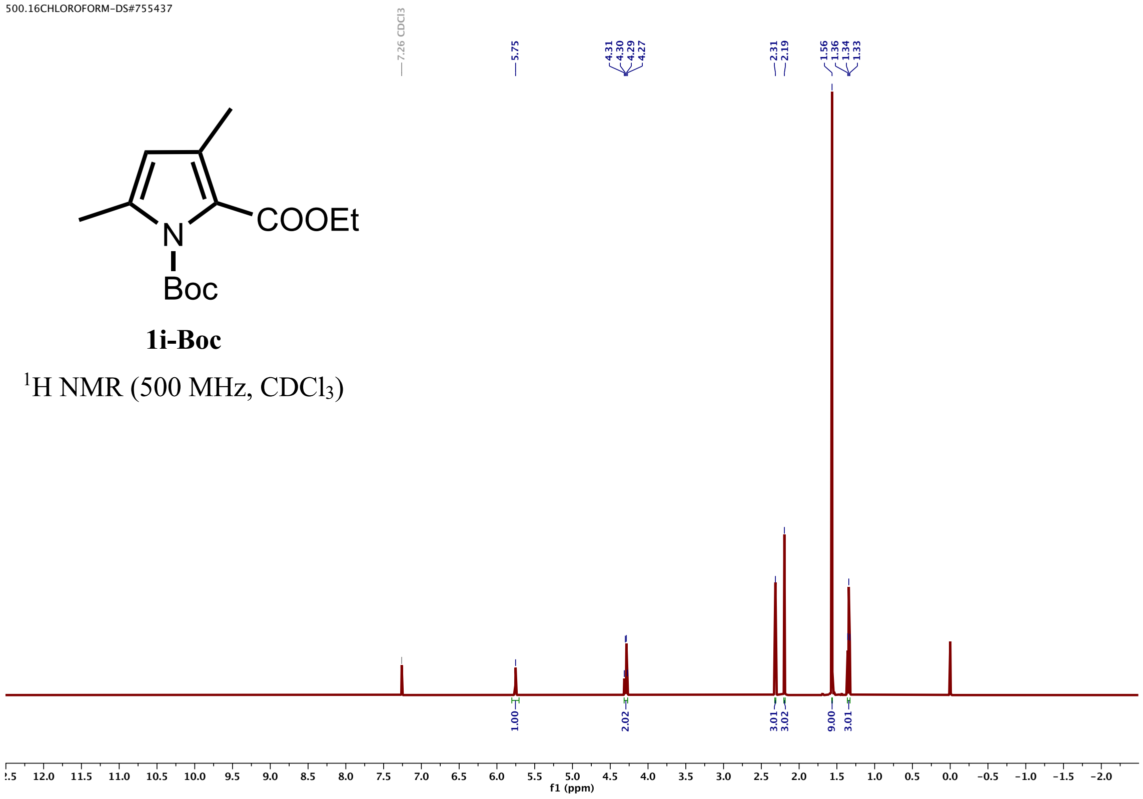

1i-Boc

^{1}H NMR (500 MHz, $CDCl_3$)

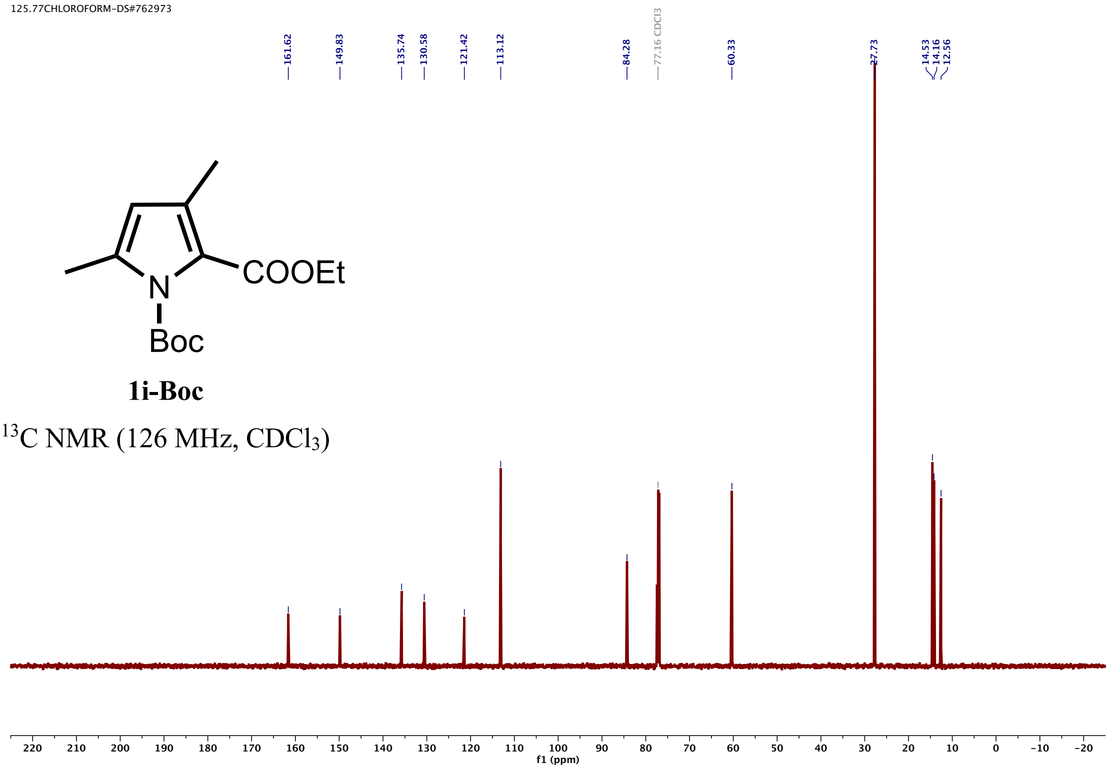

1i-Boc

^{13}C NMR (126 MHz, $CDCl_3$)

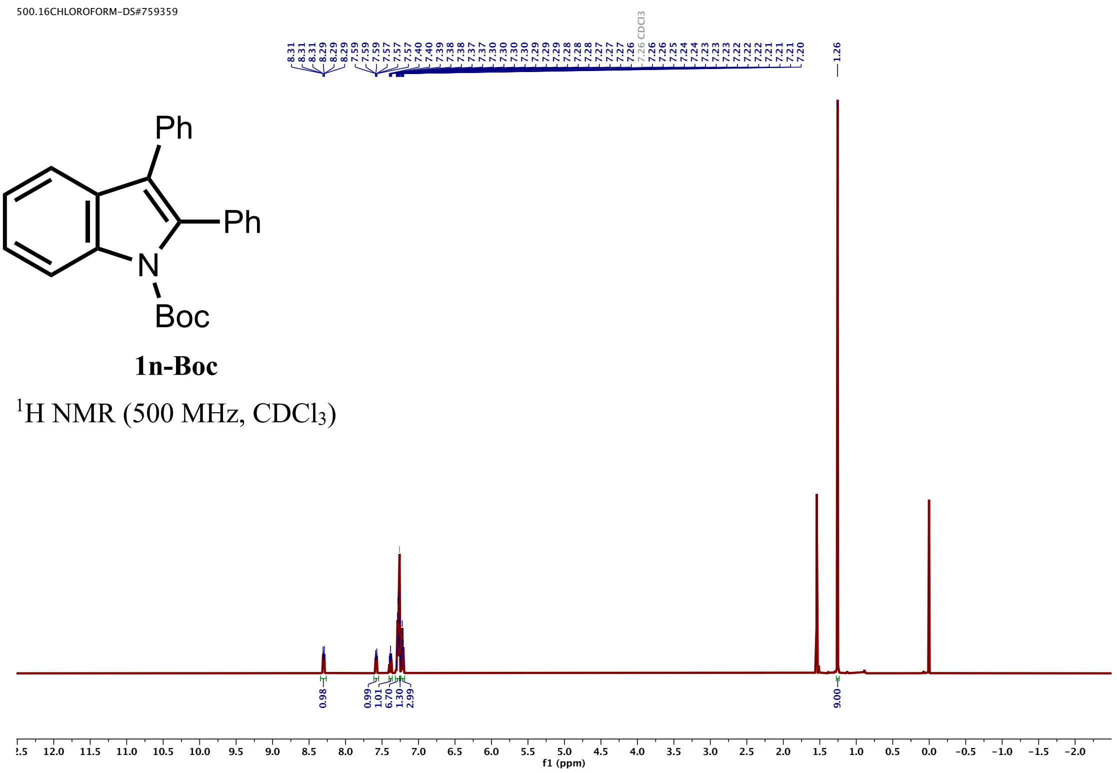

1n-Boc

^{1}H NMR (500 MHz, CDCl$_3$)

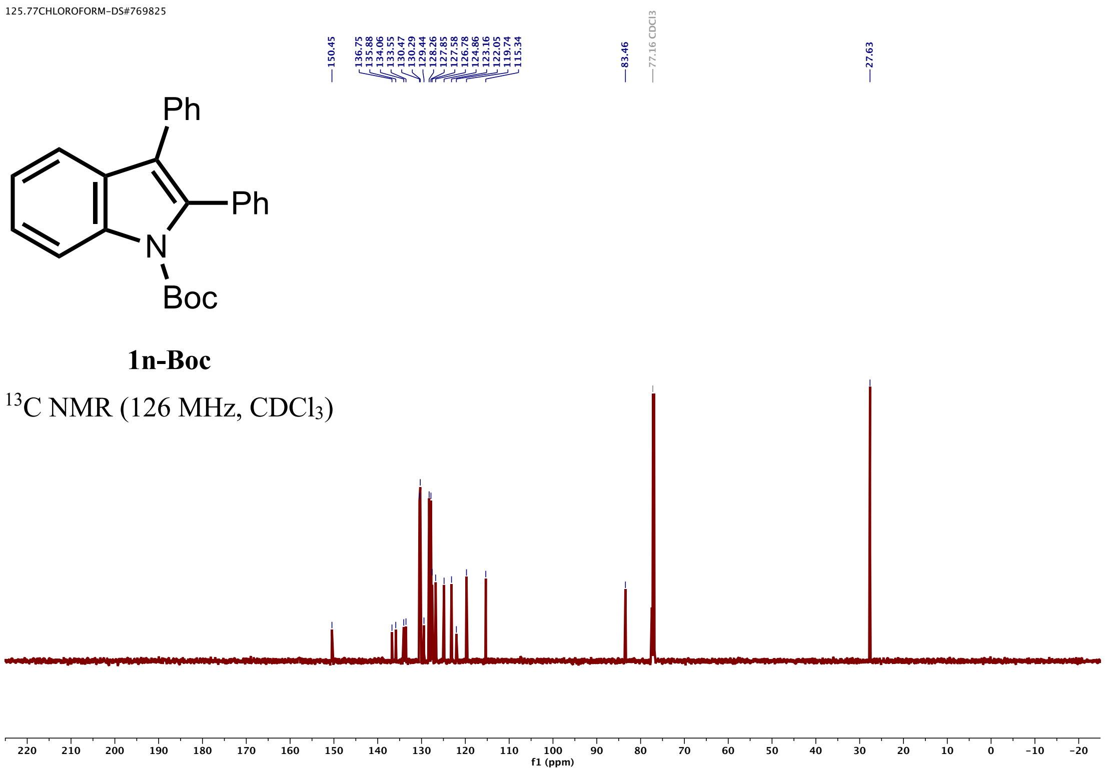

1n-Boc

^{13}C NMR (126 MHz, CDCl$_3$)

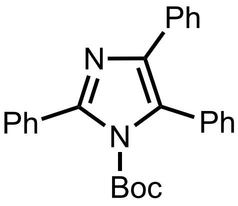

1o-Boc

^{1}H NMR (500 MHz, $CDCl_3$)

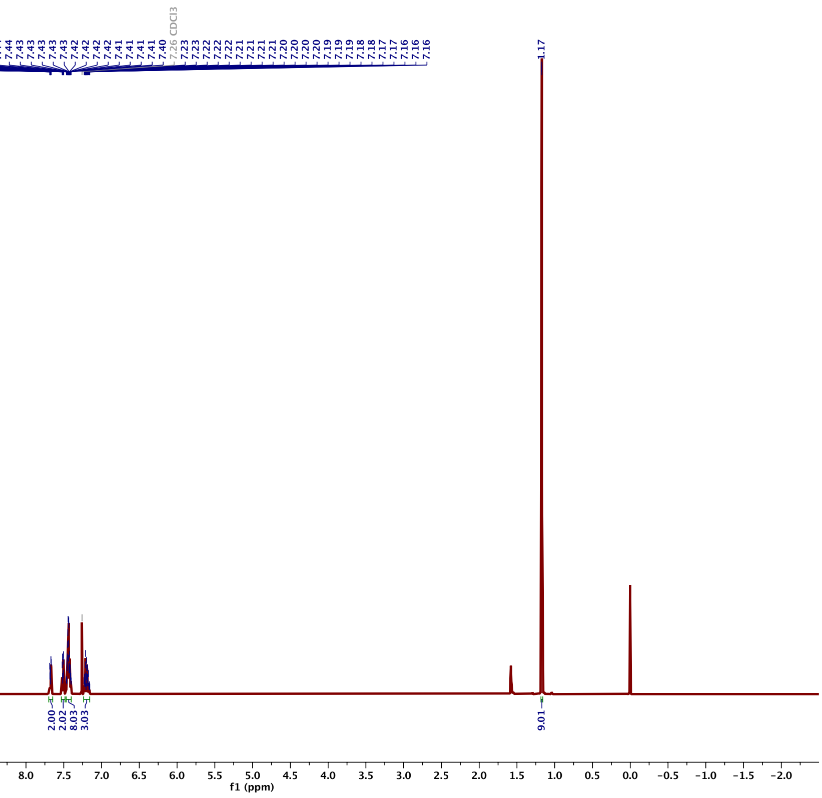

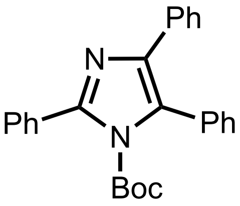

1o-Boc

^{13}C NMR (126 MHz, $CDCl_3$)

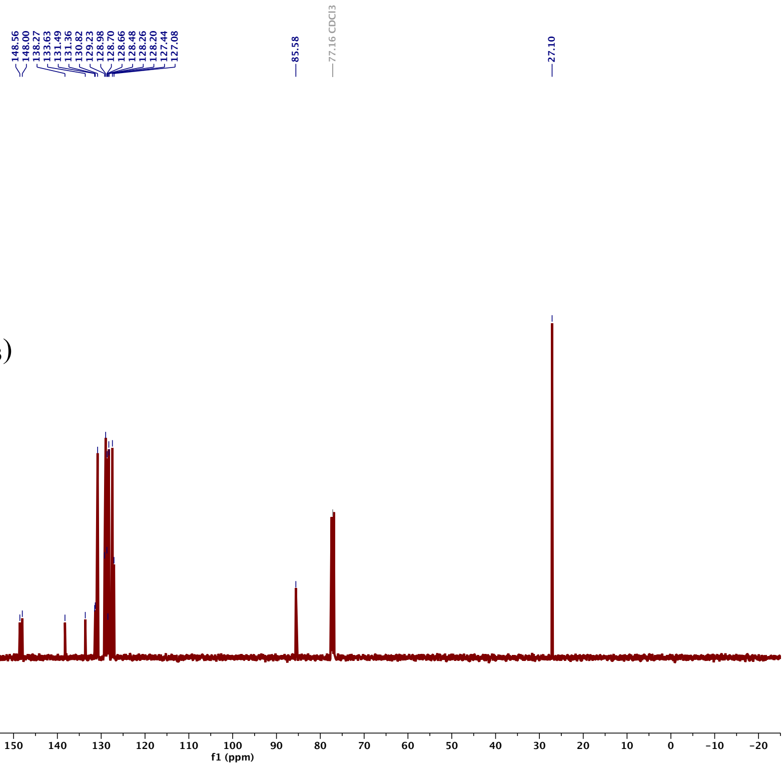

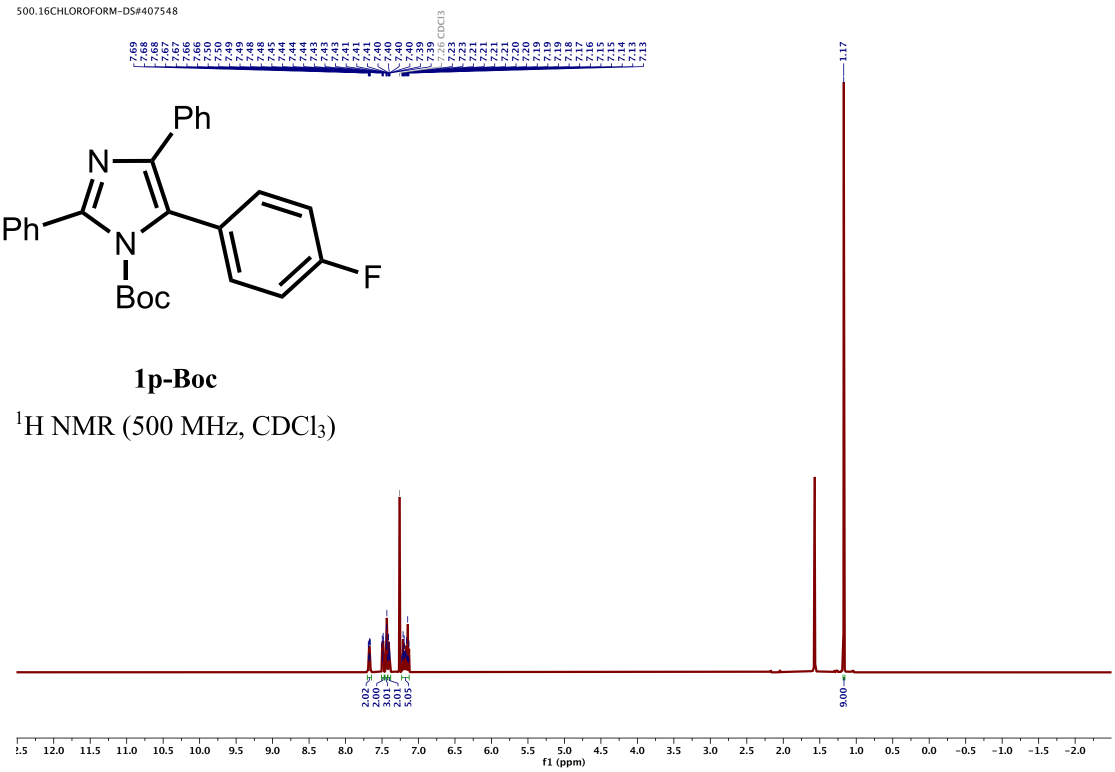

1p-Boc

^{1}H NMR (500 MHz, $CDCl_3$)

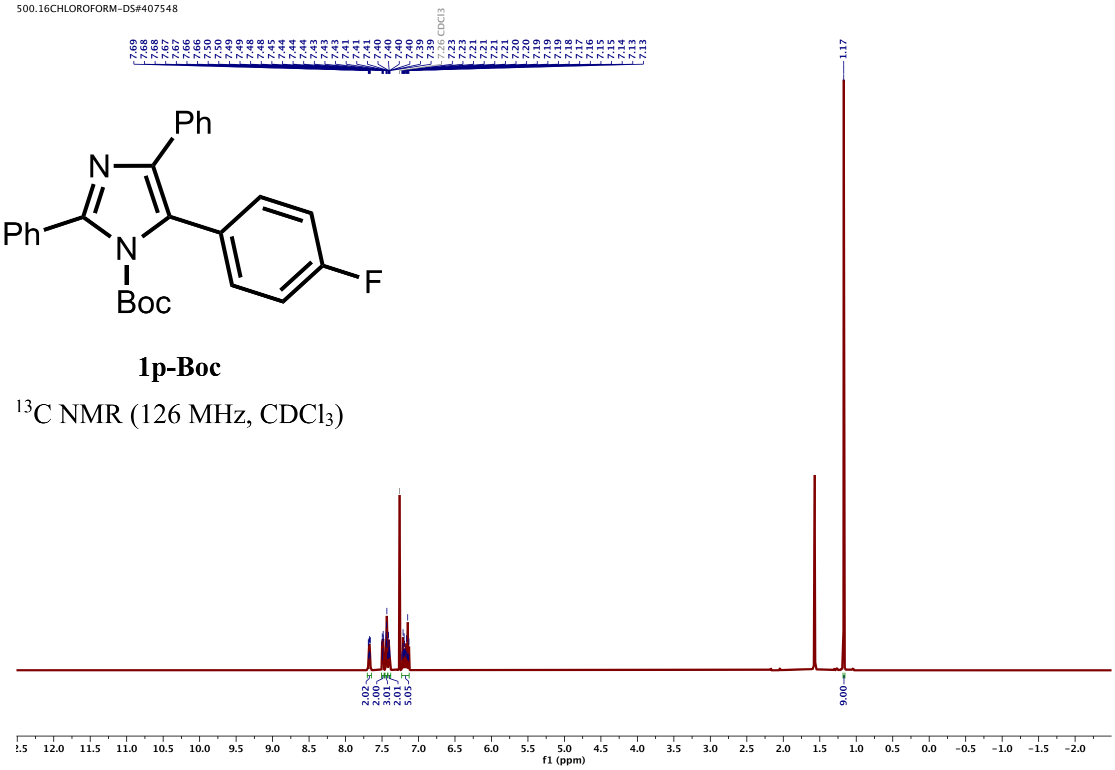

1p-Boc

^{13}C NMR (126 MHz, $CDCl_3$)

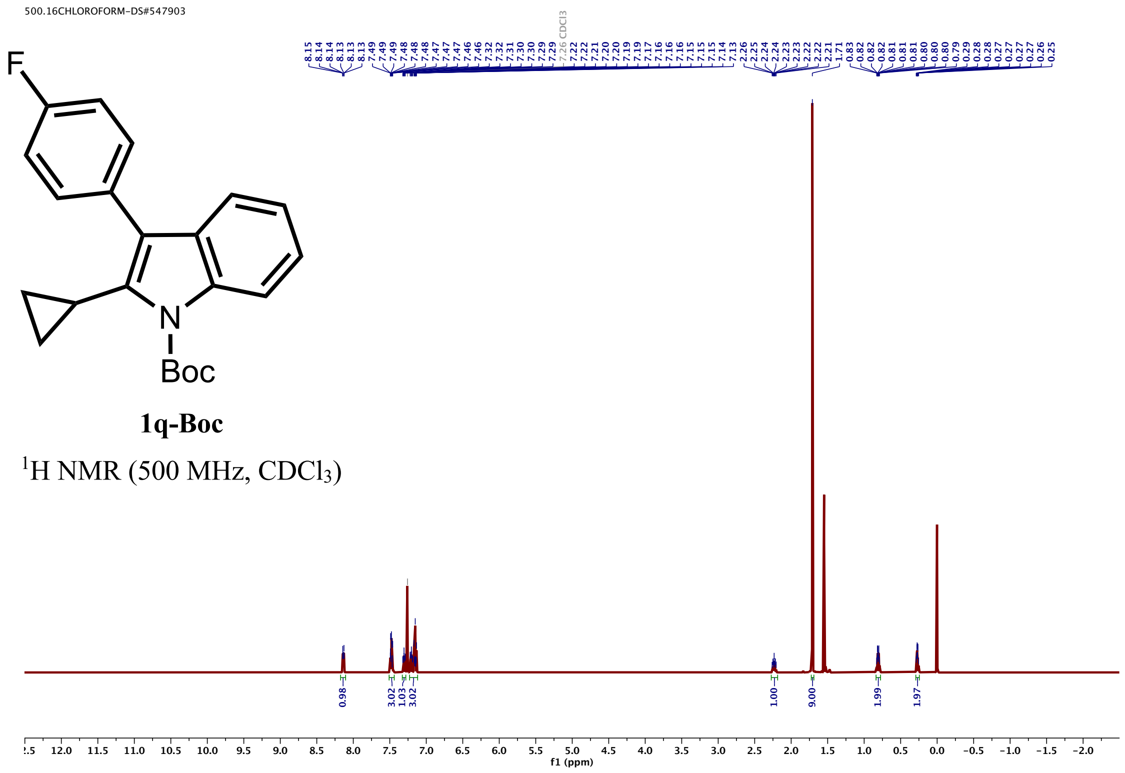

1q-Boc

^{1}H NMR (500 MHz, $CDCl_3$)

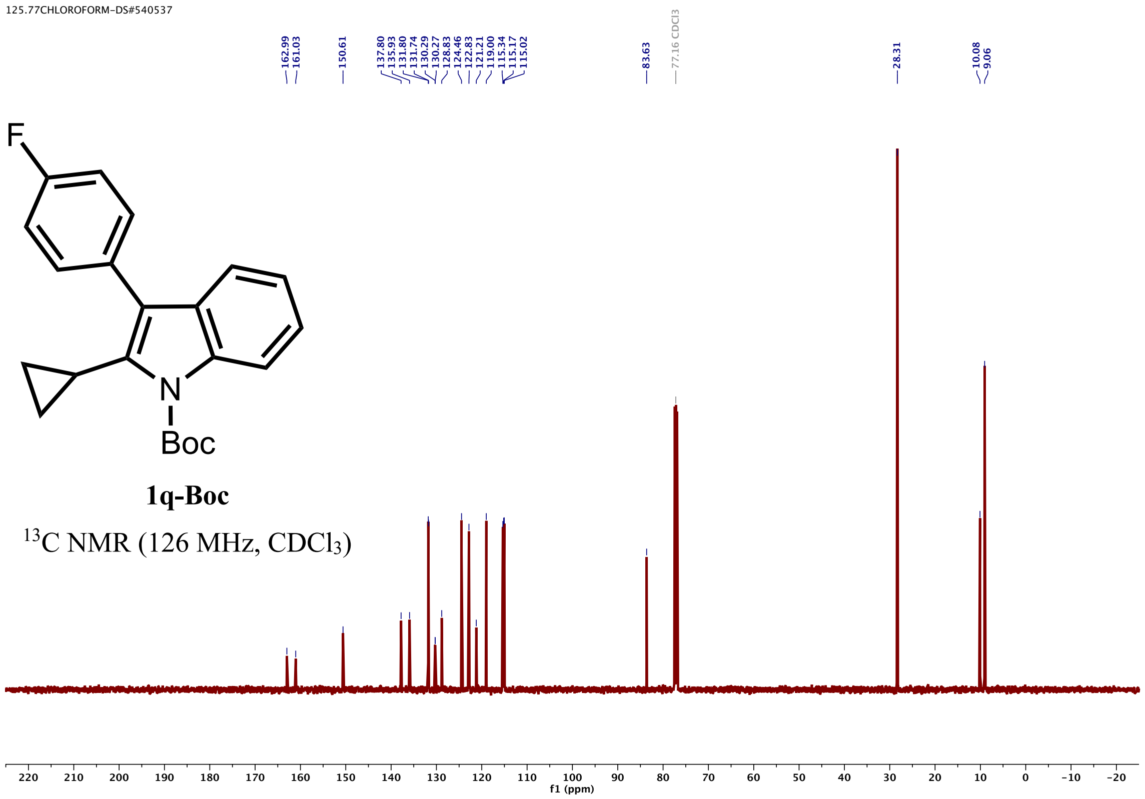

1q-Boc

^{13}C NMR (126 MHz, $CDCl_3$)

470.62CHLOROFORM-DS#551749

—-115.41

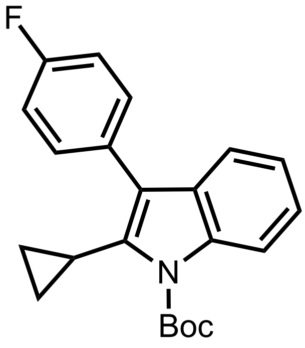

1q-Boc

^{19}F NMR (471 MHz, $CDCl_3$)

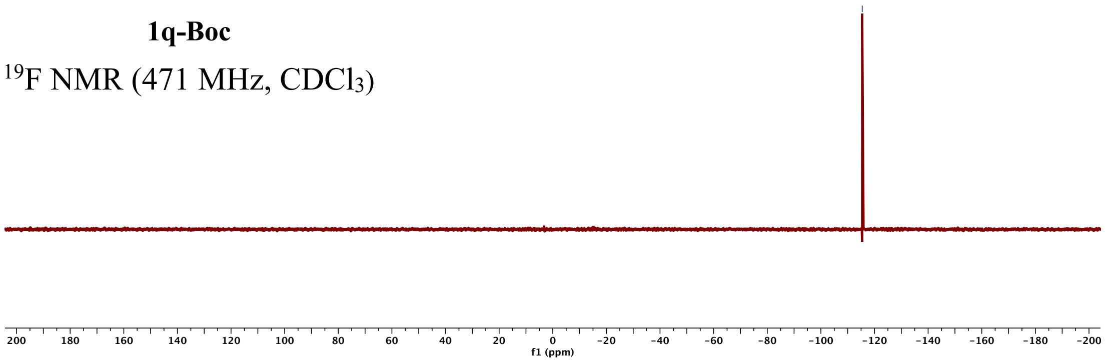

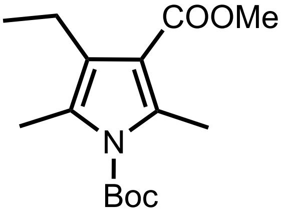

1s-Boc

^1H NMR (400 MHz, $CDCl_3$)

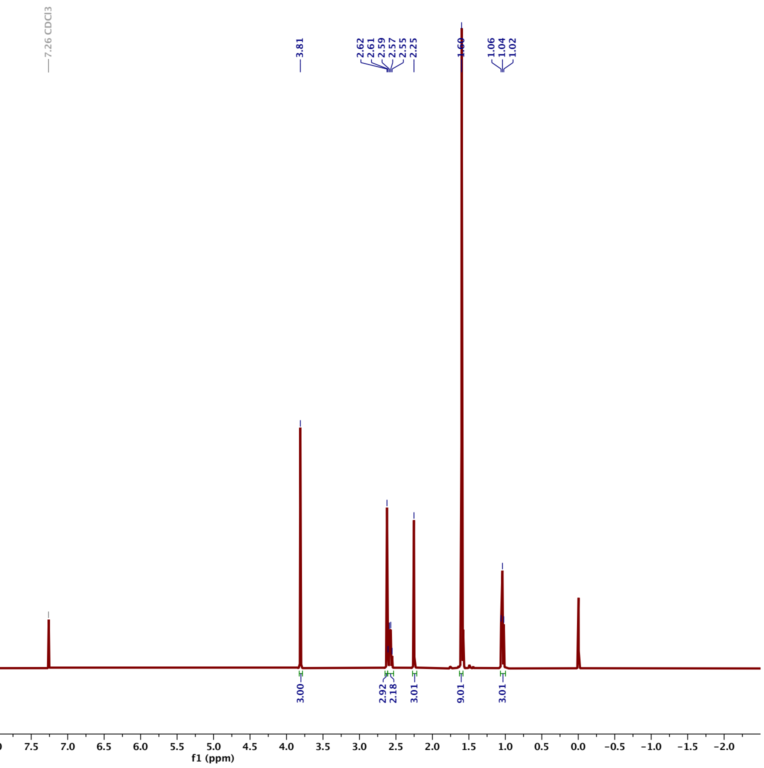

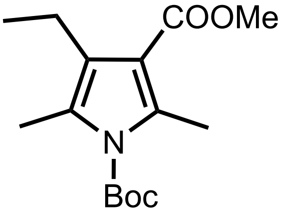

1s-Boc

^{13}C NMR (101 MHz, $CDCl_3$)

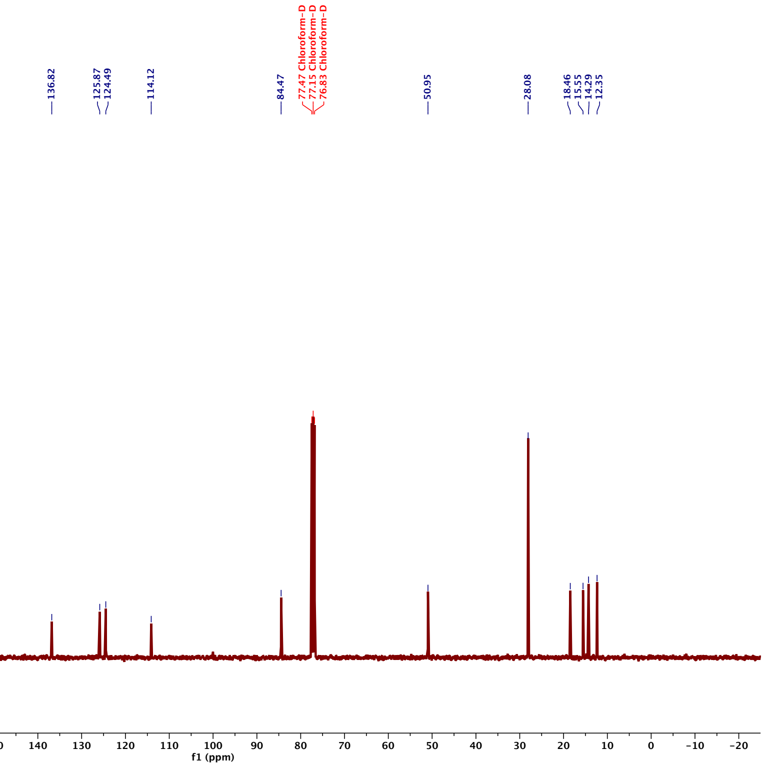

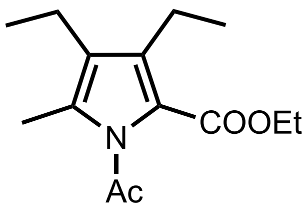

1a-Ac

^{1}H NMR (500 MHz, $CDCl_3$)

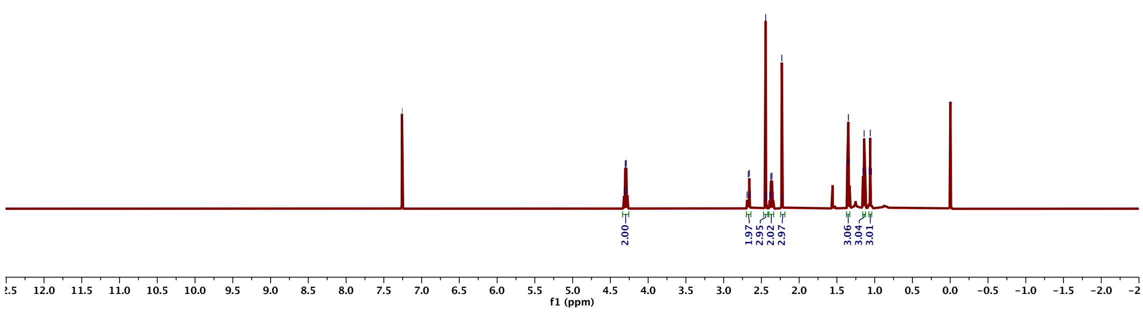

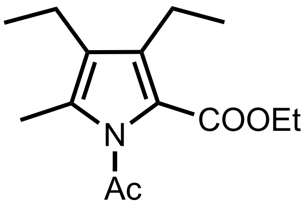

1a-Ac

^{13}C NMR (126 MHz, $CDCl_3$)

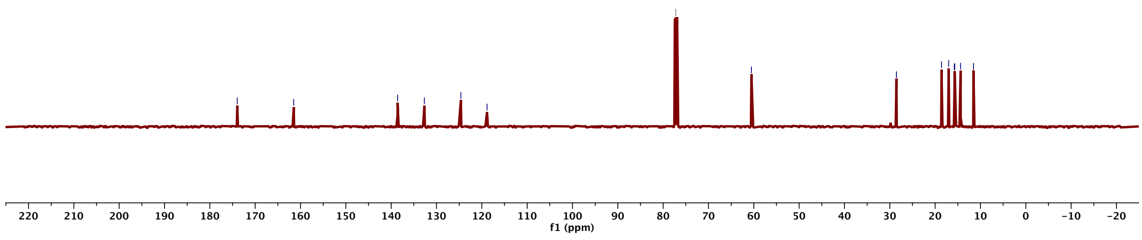

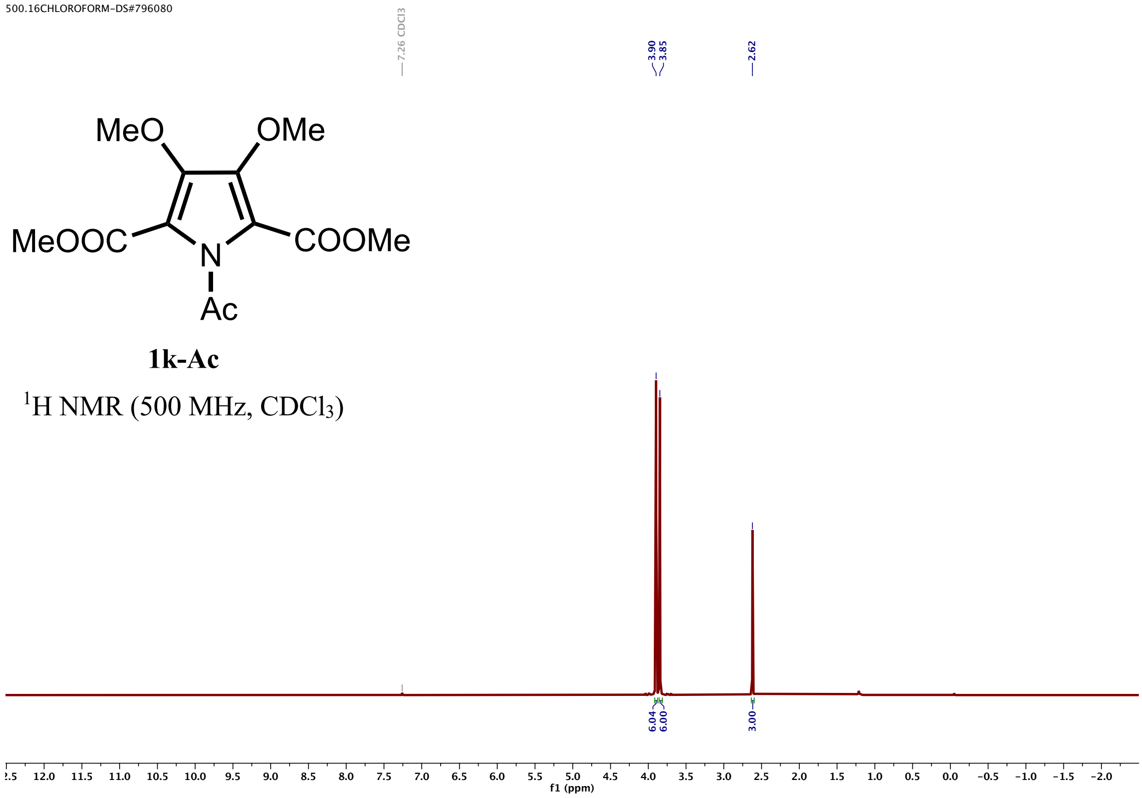
500.16CHLOROFORM-DS#796080
7.26 CDCl3
3.90
3.85
2.62
MeO
OMe
MeOOC
COOMe
N
Ac
1k-Ac
^{1}H NMR (500 MHz, $CDCl_3$)
6.04
6.00
3.00
f1 (ppm)

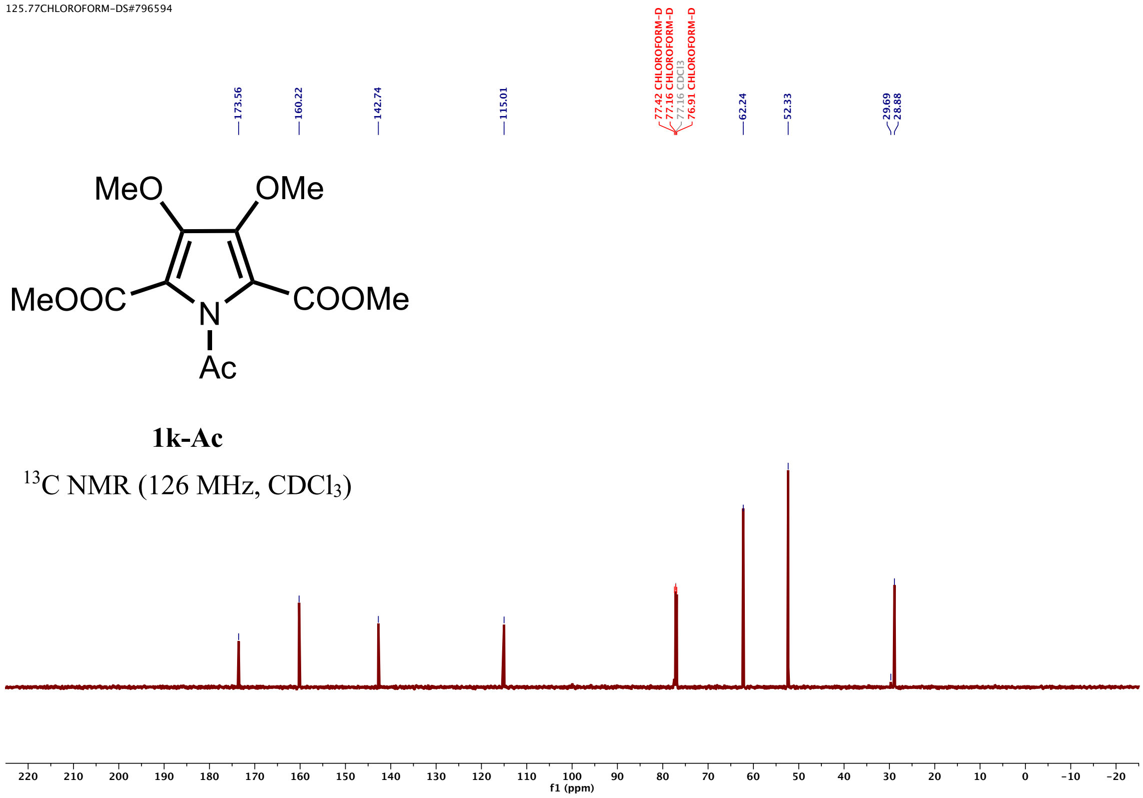
125.77CHLOROFORM-DS#796594
173.56
160.22
142.74
115.01
77.42 CHLOROFORM-D
77.16 CHLOROFORM-D
77.16 CDCl3
76.91 CHLOROFORM-D
62.24
52.33
29.69
28.88
MeO
OMe
MeOOC
COOMe
N
Ac
1k-Ac
^{13}C NMR (126 MHz, $CDCl_3$)
f1 (ppm)

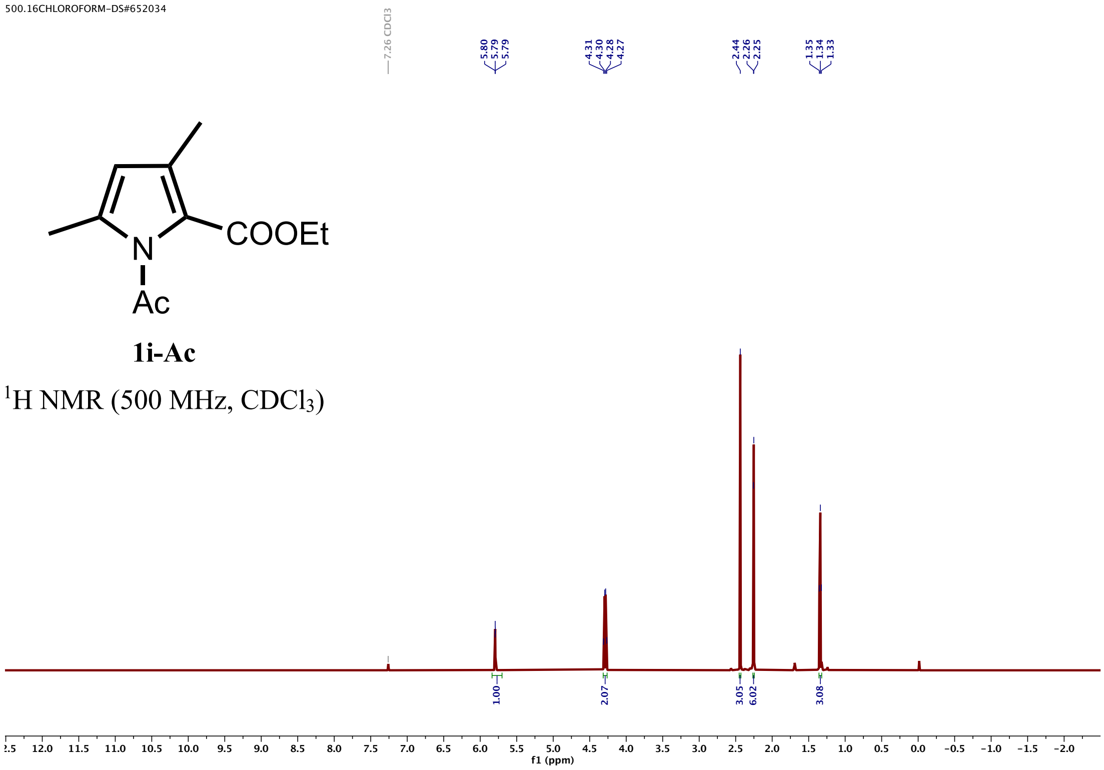

1i-Ac

^{1}H NMR (500 MHz, $CDCl_3$)

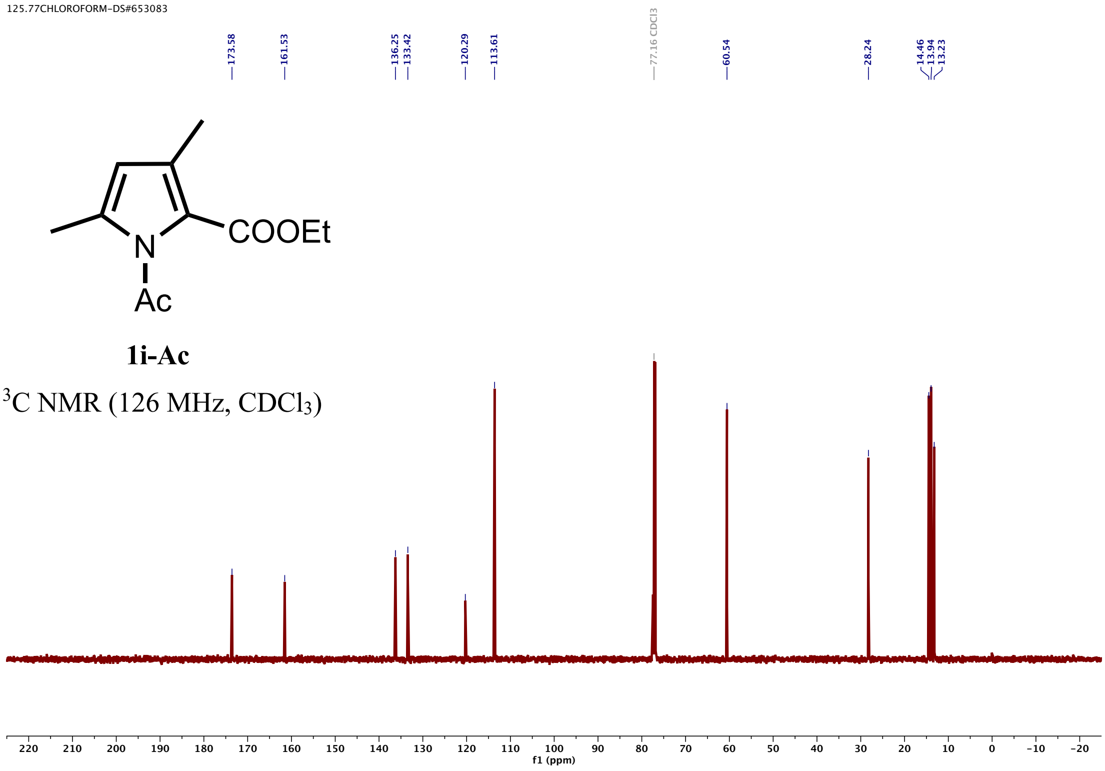

1i-Ac

^{13}C NMR (126 MHz, $CDCl_3$)

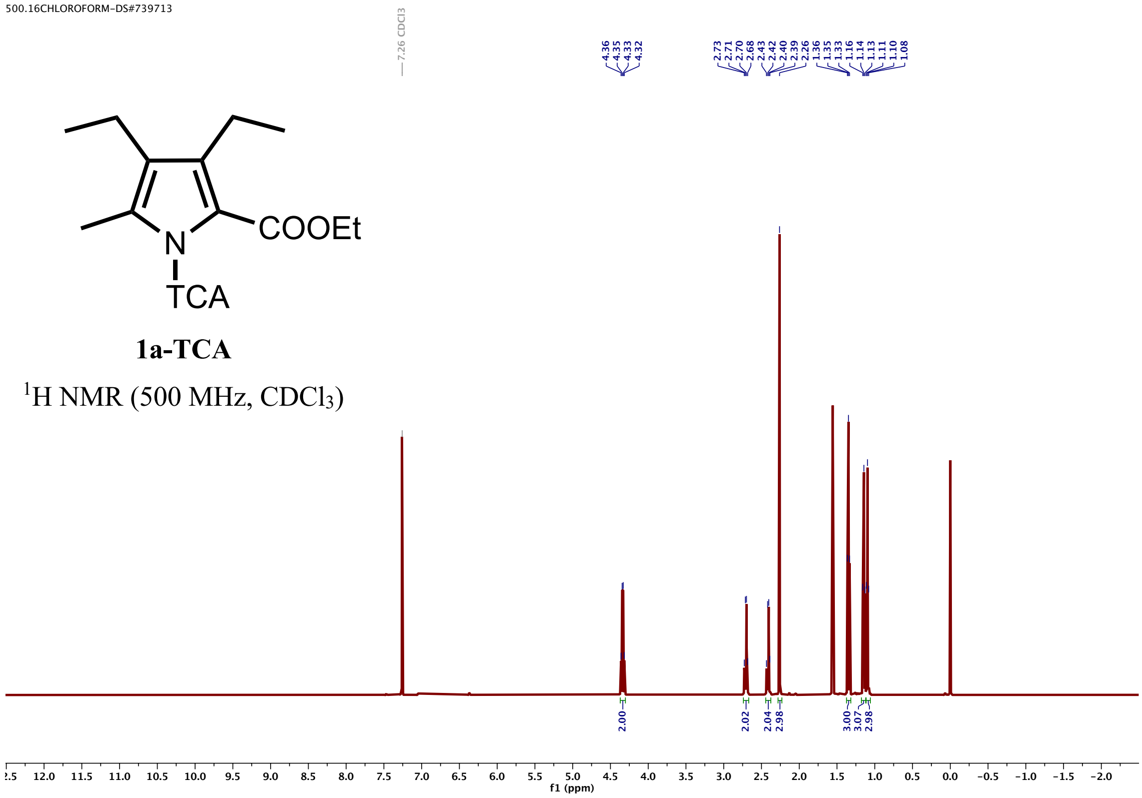
500.16CHLOROFORM-DS#739713
7.26 CDCl3
4.36
4.35
4.33
4.32
2.73
2.71
2.70
2.68
2.43
2.42
2.40
2.39
2.26
1.36
1.35
1.33
1.16
1.14
1.13
1.11
1.10
1.08
COOEt
N
TCA
1a-TCA
1H NMR (500 MHz, CDCl3)
2.00
2.02
2.04
2.98
3.00
3.07
2.98
f1 (ppm)

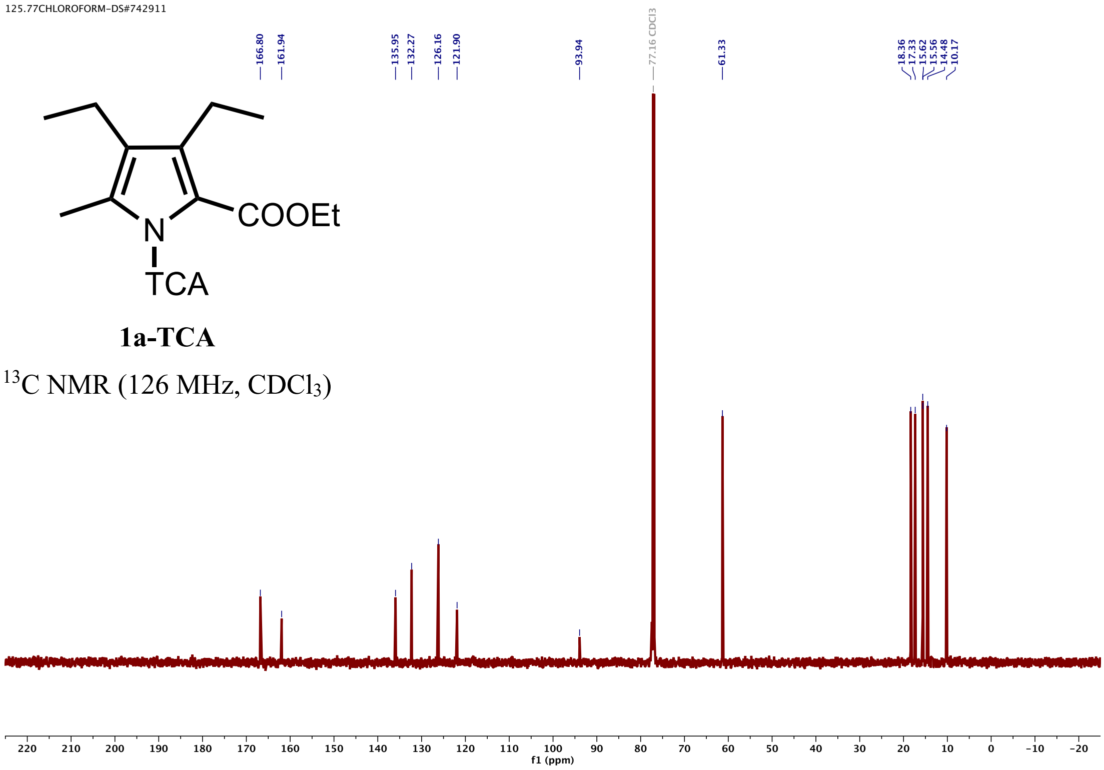
125.77CHLOROFORM-DS#742911
166.80
161.94
135.95
132.27
126.16
121.90
93.94
77.16 CDCl3
61.33
18.36
17.33
15.62
15.56
14.48
10.17
COOEt
N
TCA
1a-TCA
13C NMR (126 MHz, CDCl3)
f1 (ppm)

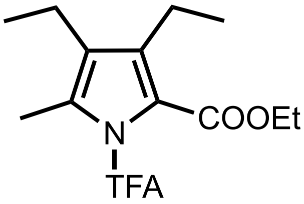

1a-TFA

^{1}H NMR (500 MHz, $CDCl_3$)

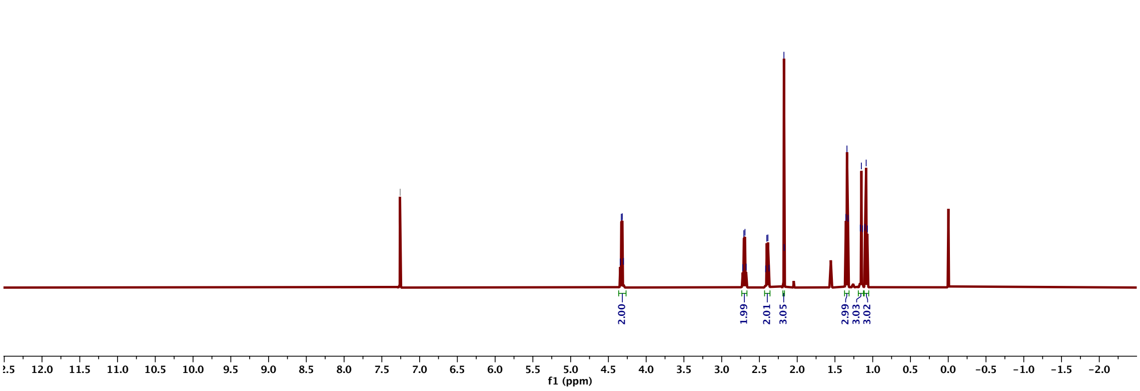

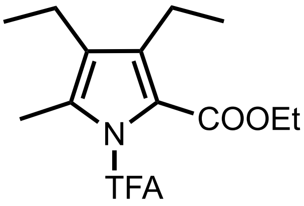

1a-TFA

^{13}C NMR (126 MHz, $CDCl_3$)

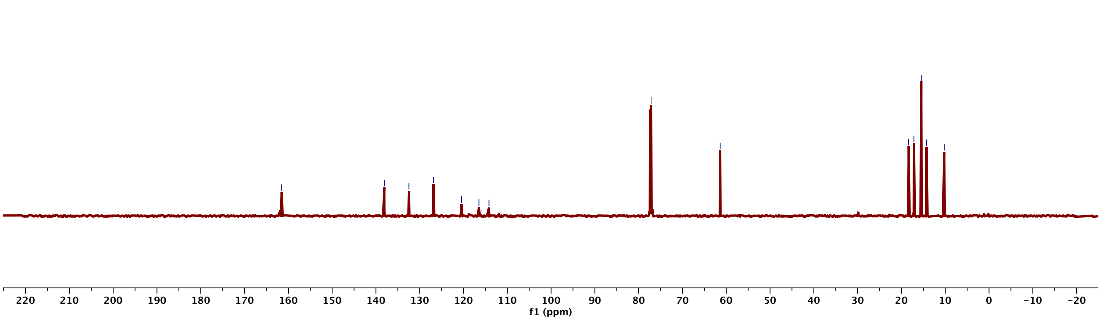

470.62CHLOROFORM-DS#588422

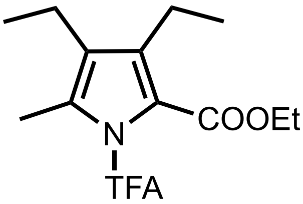

1a-TFA

^{19}F NMR (471 MHz, $CDCl_3$)

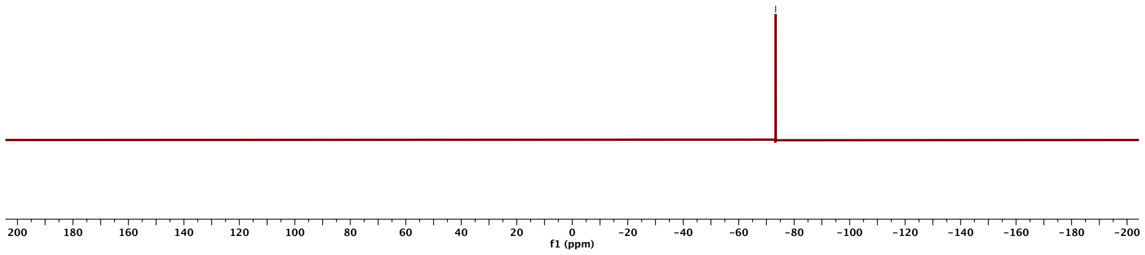

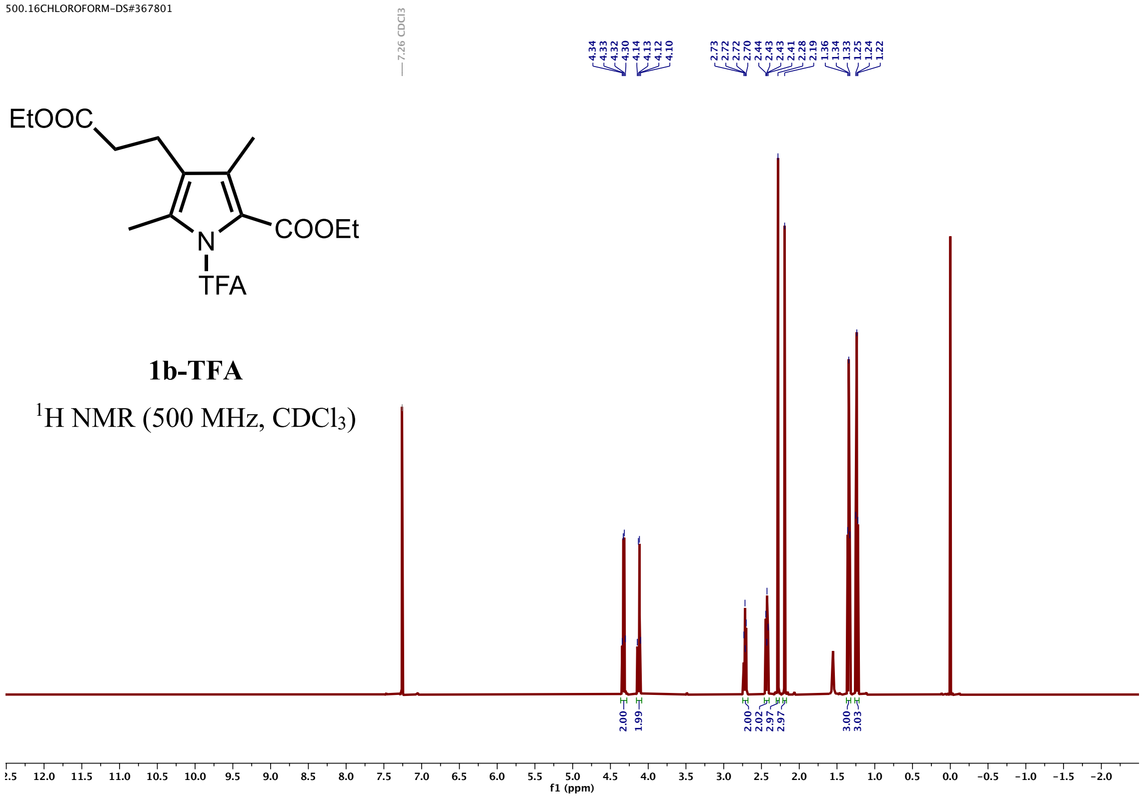

1b-TFA

^{1}H NMR (500 MHz, $CDCl_3$)

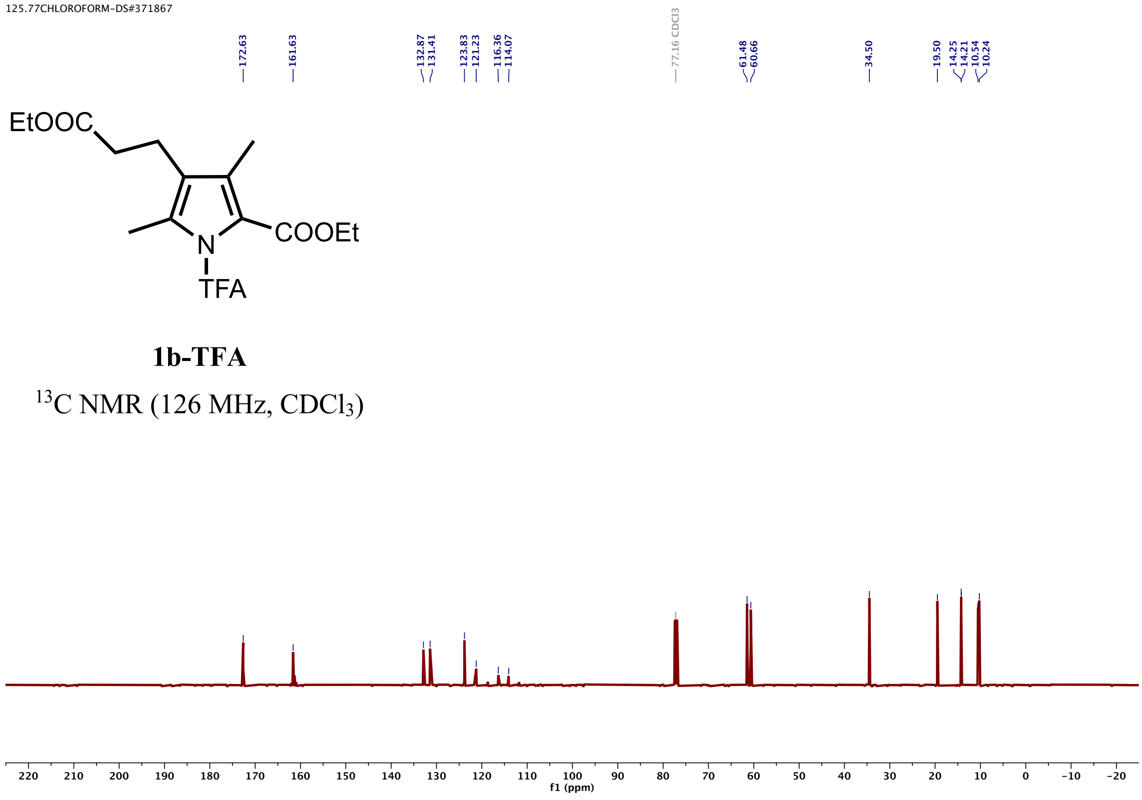

1b-TFA

^{13}C NMR (126 MHz, $CDCl_3$)

470.62CHLOROFORM-DS#368763

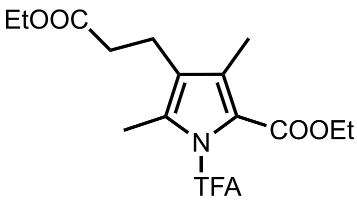

1b-TFA

^{19}F NMR (471 MHz, $CDCl_3$)

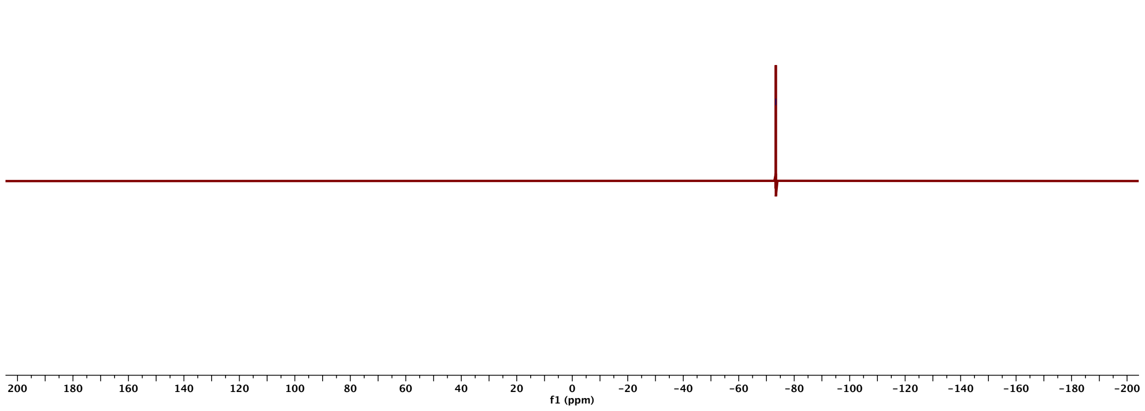

500.16CHLOROFORM-DS#746333

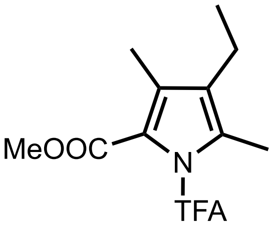

1c-TFA

^{1}H NMR (500 MHz, $CDCl_3$)

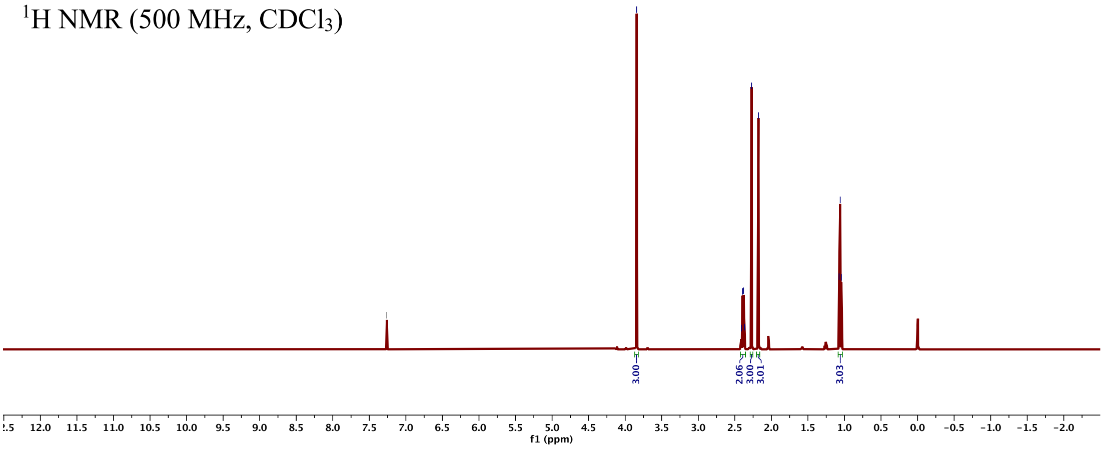

125.77CHLOROFORM-DS#747343

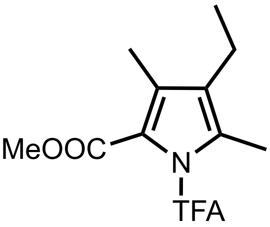

1c-TFA

^{13}C NMR (126 MHz, $CDCl_3$)

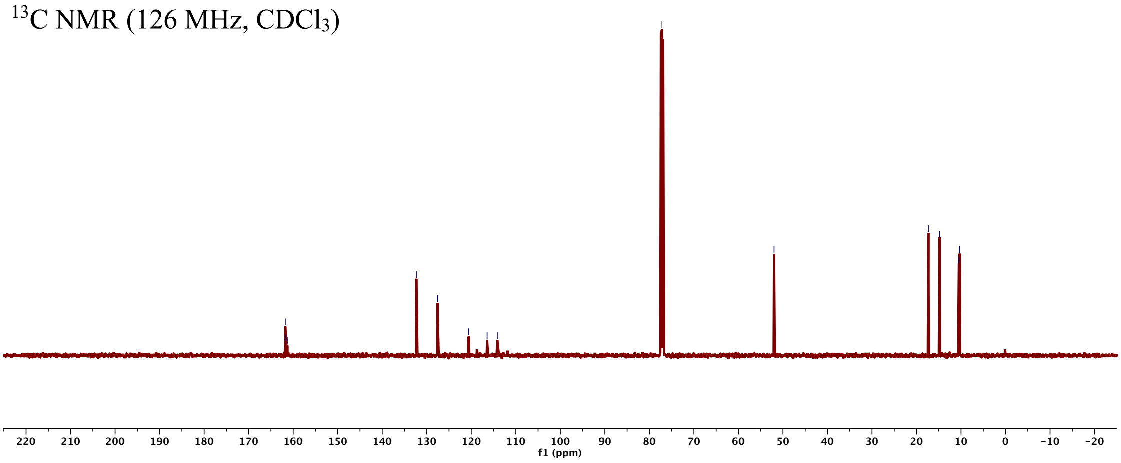

470.62CHLOROFORM-DS#746868

—-73.25

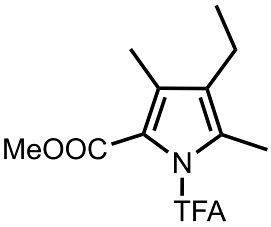

1c-TFA

^{19}F NMR (471 MHz, $CDCl_3$)

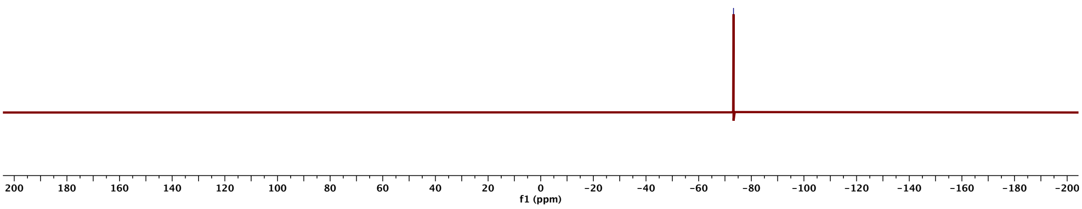

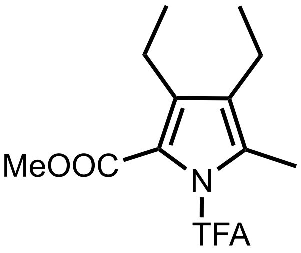

1d-TFA

^{1}H NMR (500 MHz, $CDCl_3$)

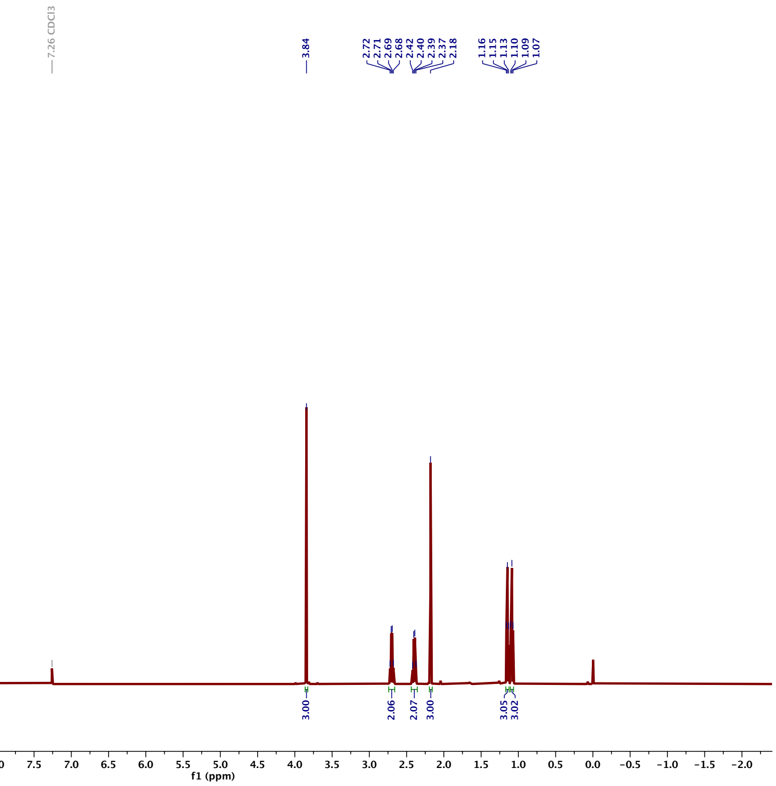

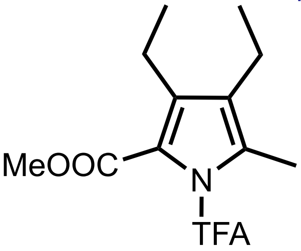

1d-TFA

^{13}C NMR (126 MHz, $CDCl_3$)

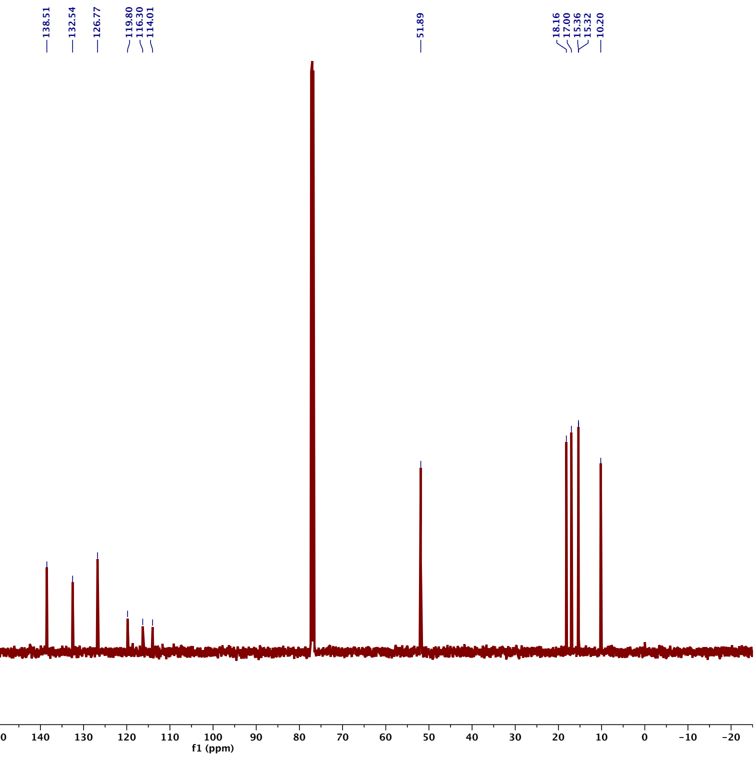

470.62CHLOROFORM-DS#653214

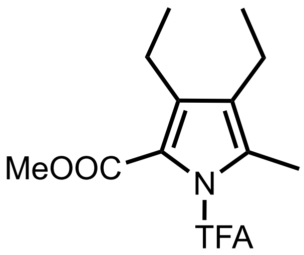

1d-TFA

^{19}F NMR (471 MHz, $CDCl_3$)

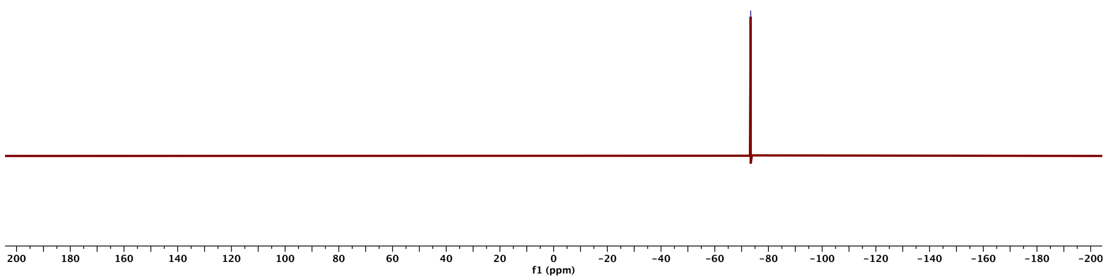

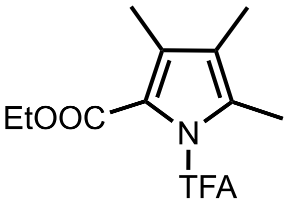

1e-TFA

^{1}H NMR (400 MHz, $CDCl_3$)

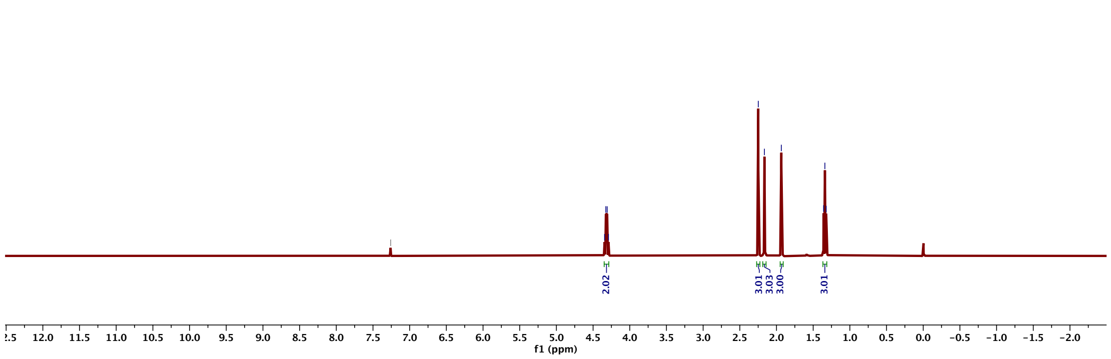

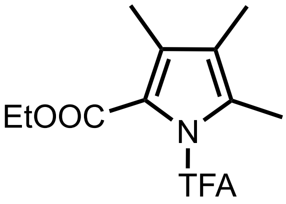

1e-TFA

^{13}C NMR (126 MHz, $CDCl_3$)

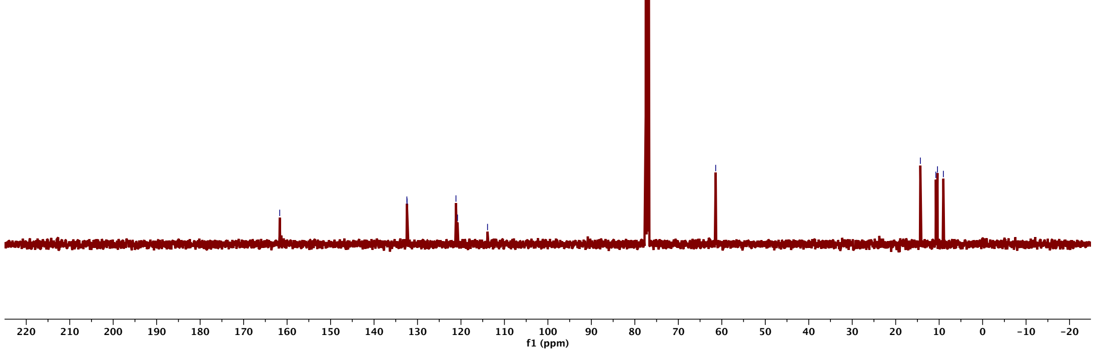

470.62CHLOROFORM-DS#747711

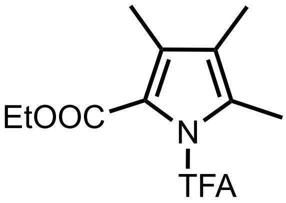

1e-TFA

^{19}F NMR (471 MHz, $CDCl_3$)

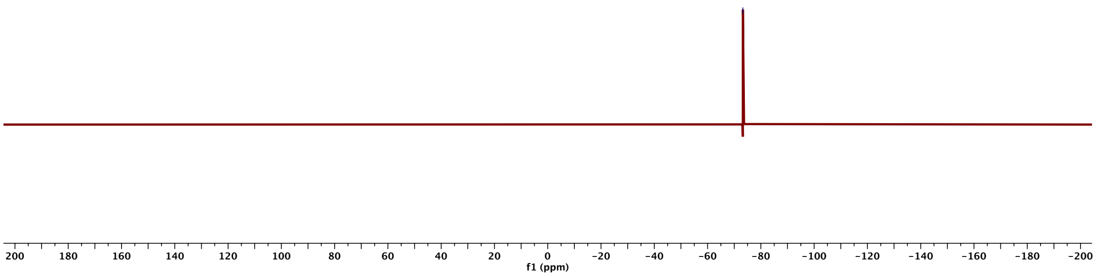

500.16CHLOROFORM-DS#761371

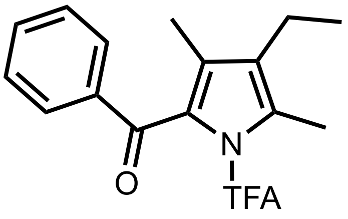

1f-TFA

^{1}H NMR (500 MHz, CDCl$_3$)

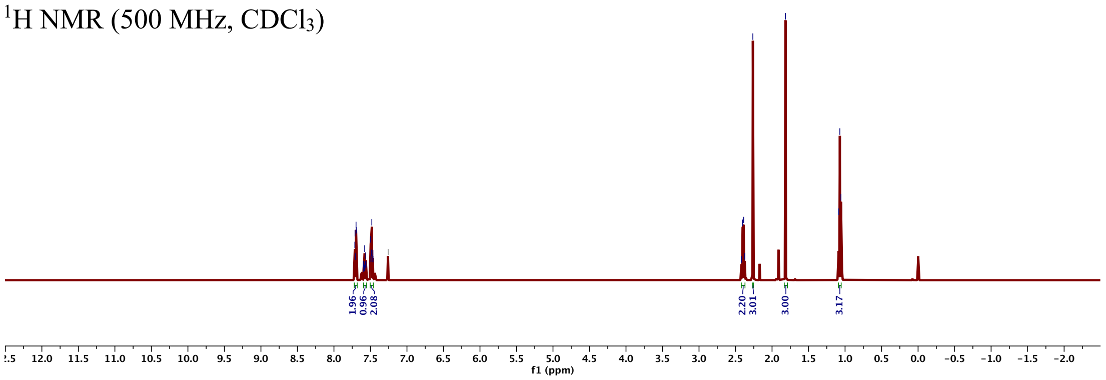

125.77CHLOROFORM-DS#762942

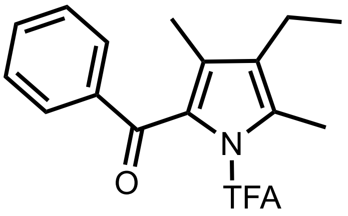

1f-TFA

^{13}C NMR (126 MHz, CDCl$_3$)

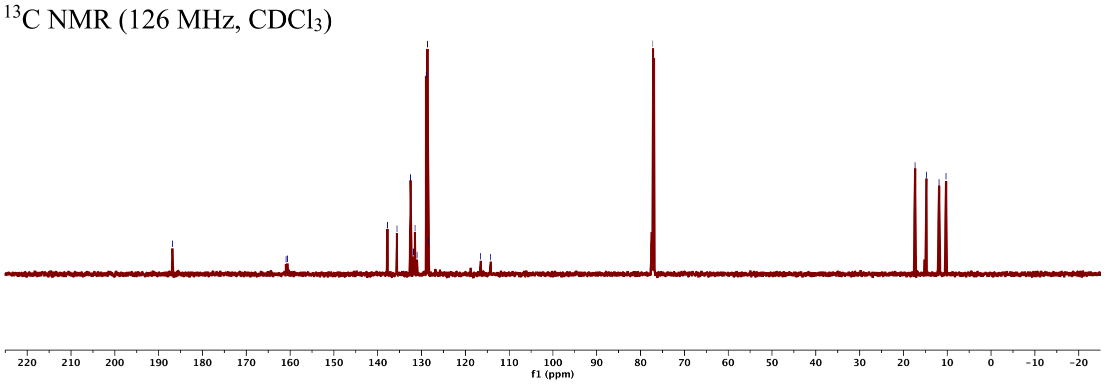

470.62CHLOROFORM-DS#593192

−73.20

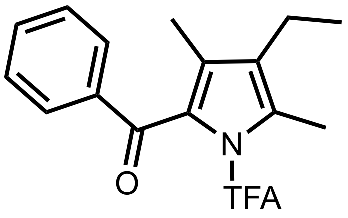

1f-TFA

^{19}F NMR (471 MHz, $CDCl_3$)

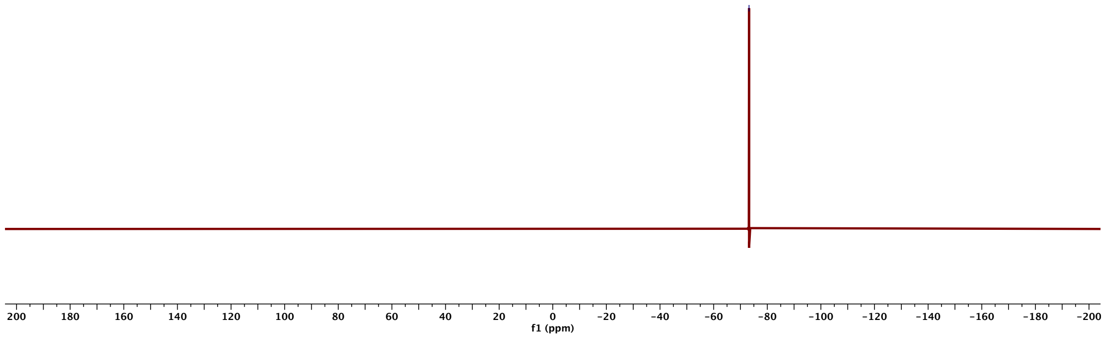

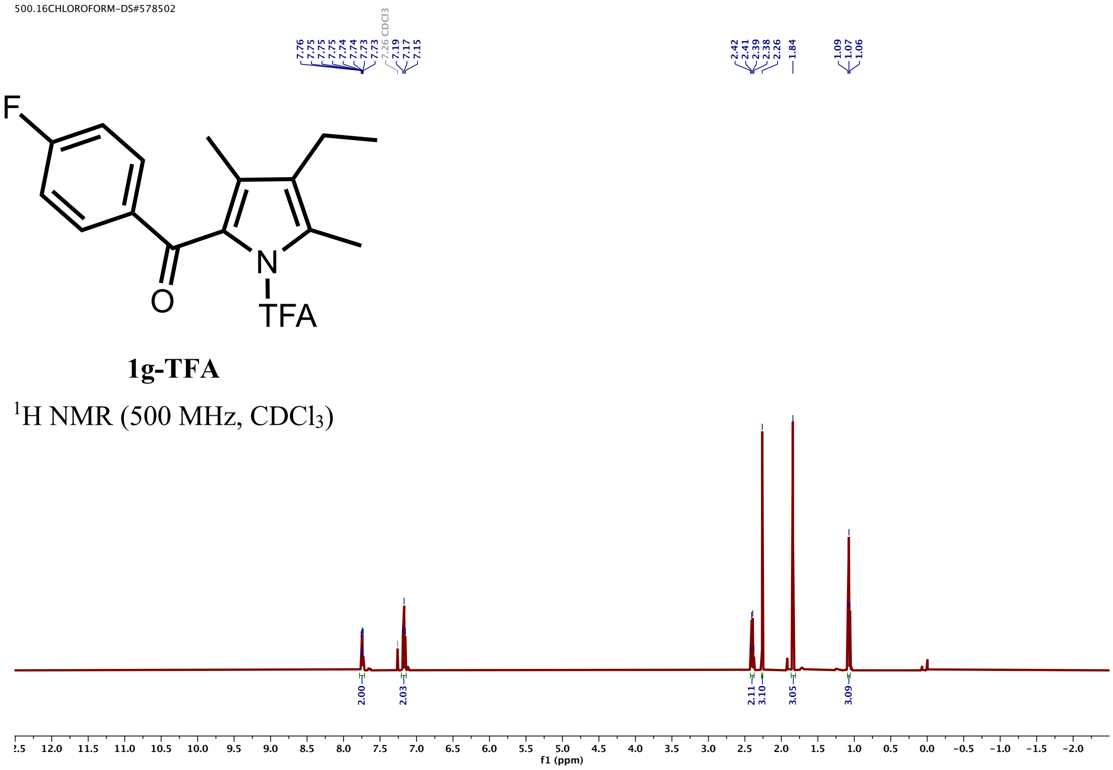

1g-TFA

^{1}H NMR (500 MHz, $CDCl_3$)

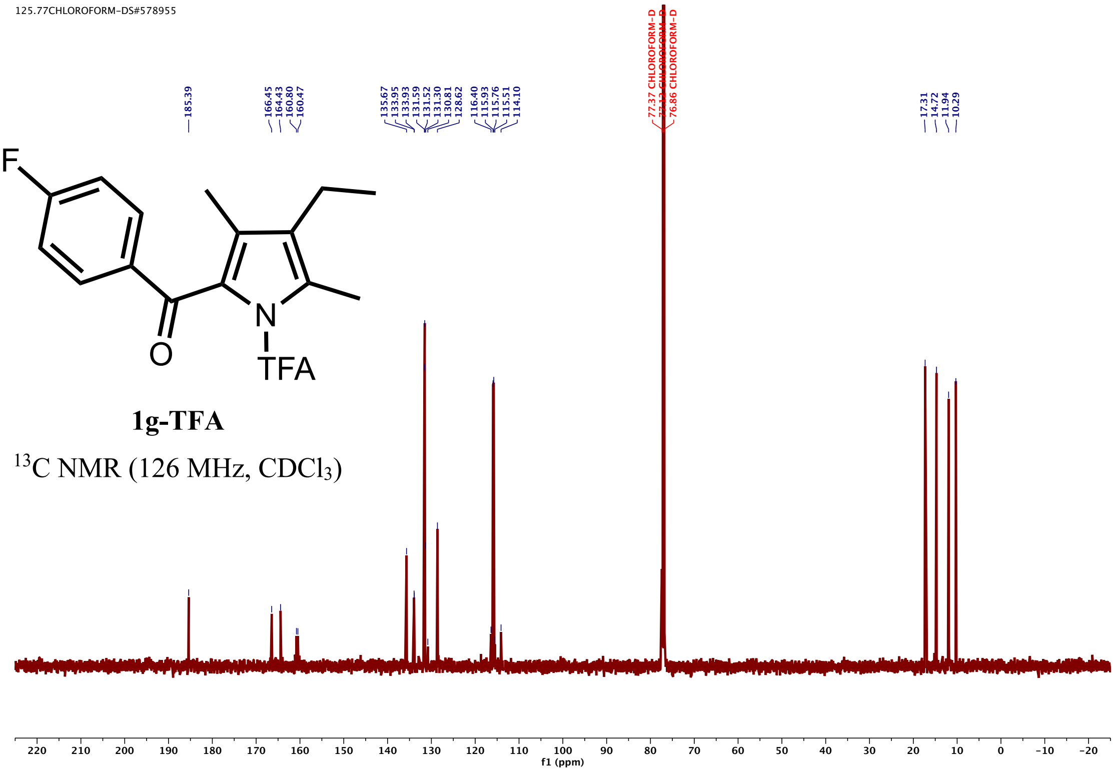

1g-TFA

^{13}C NMR (126 MHz, $CDCl_3$)

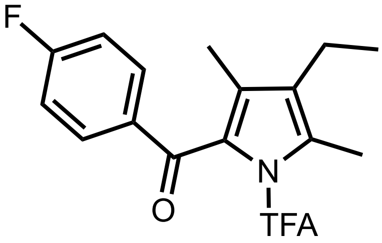

1g-TFA

^{19}F NMR (471 MHz, $CDCl_3$)

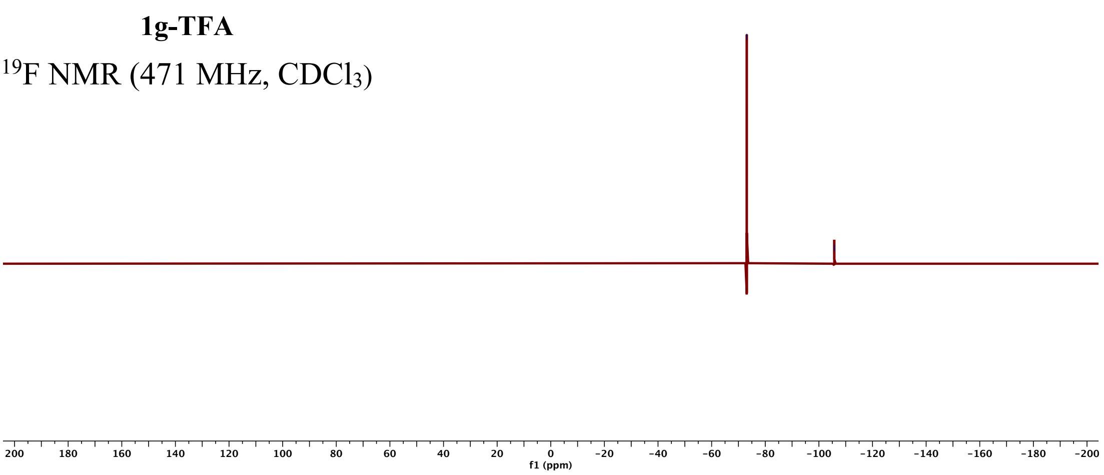

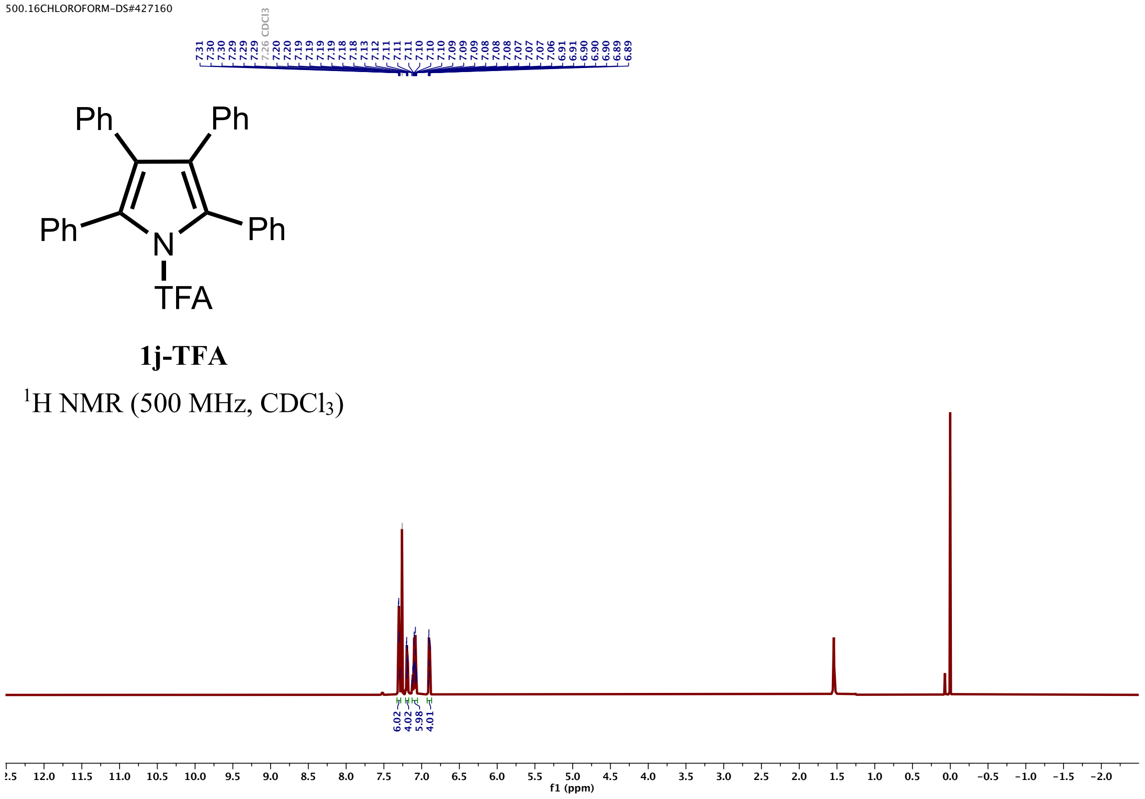

1j-TFA

^{1}H NMR (500 MHz, $CDCl_3$)

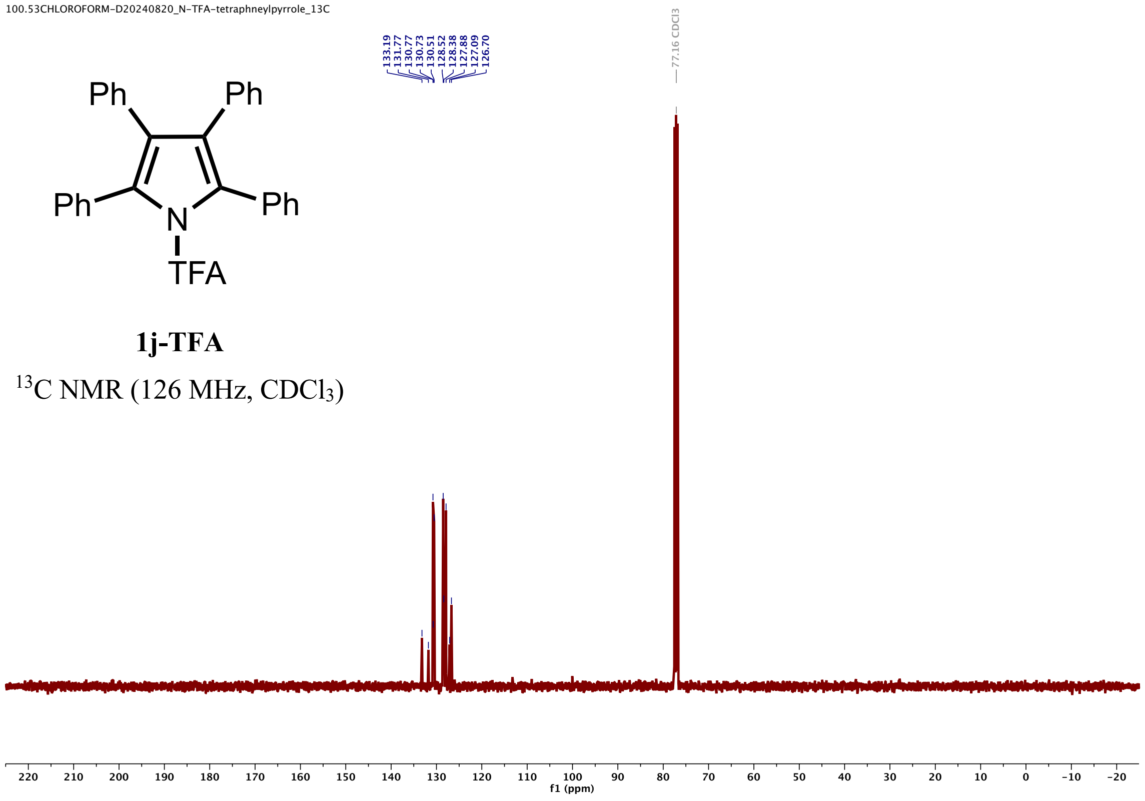

1j-TFA

^{13}C NMR (126 MHz, $CDCl_3$)

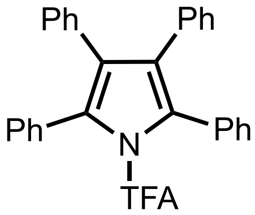

1j-TFA

^{19}F NMR (471 MHz, $CDCl_3$)

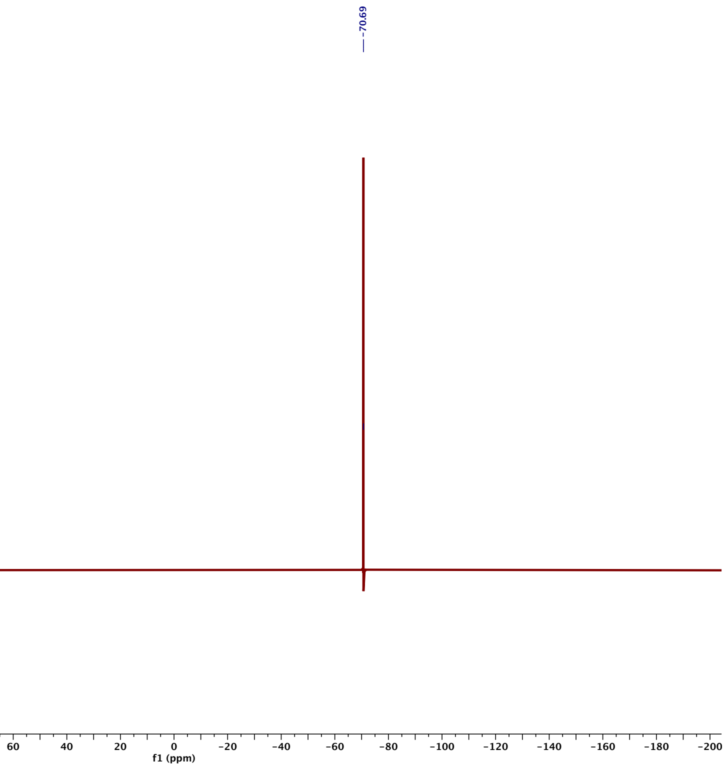

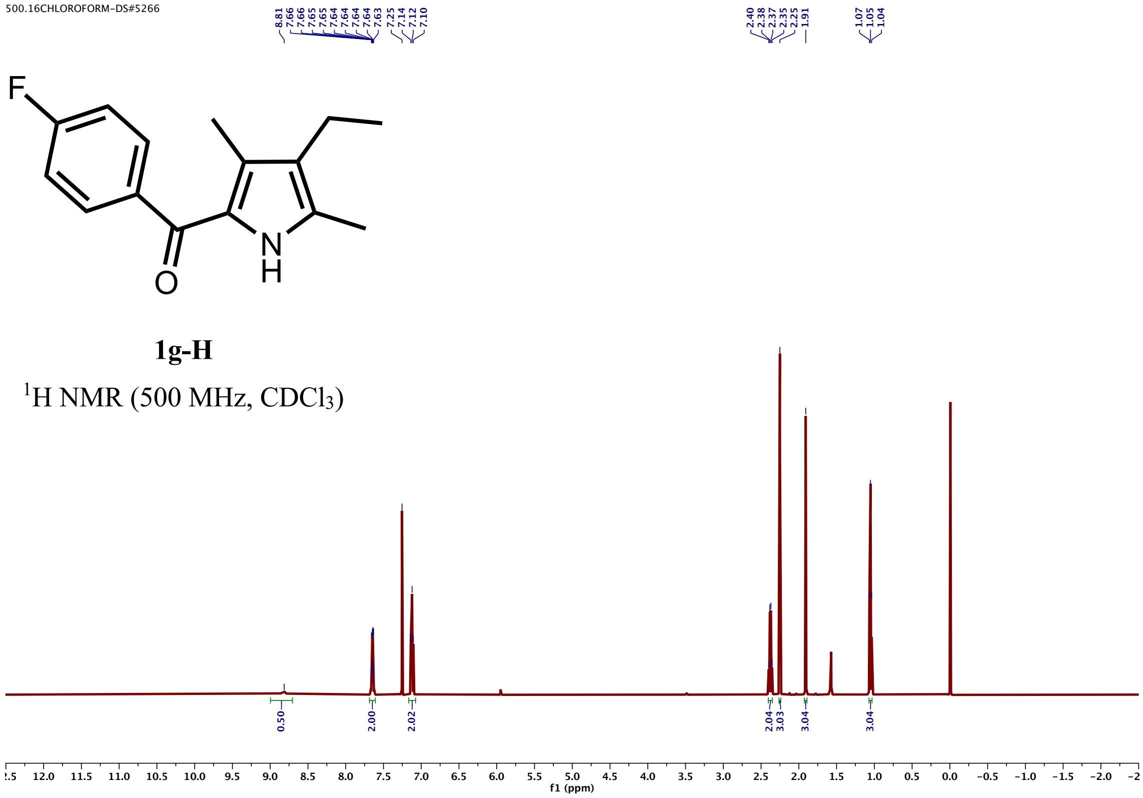

1g-H

^{1}H NMR (500 MHz, $CDCl_3$)

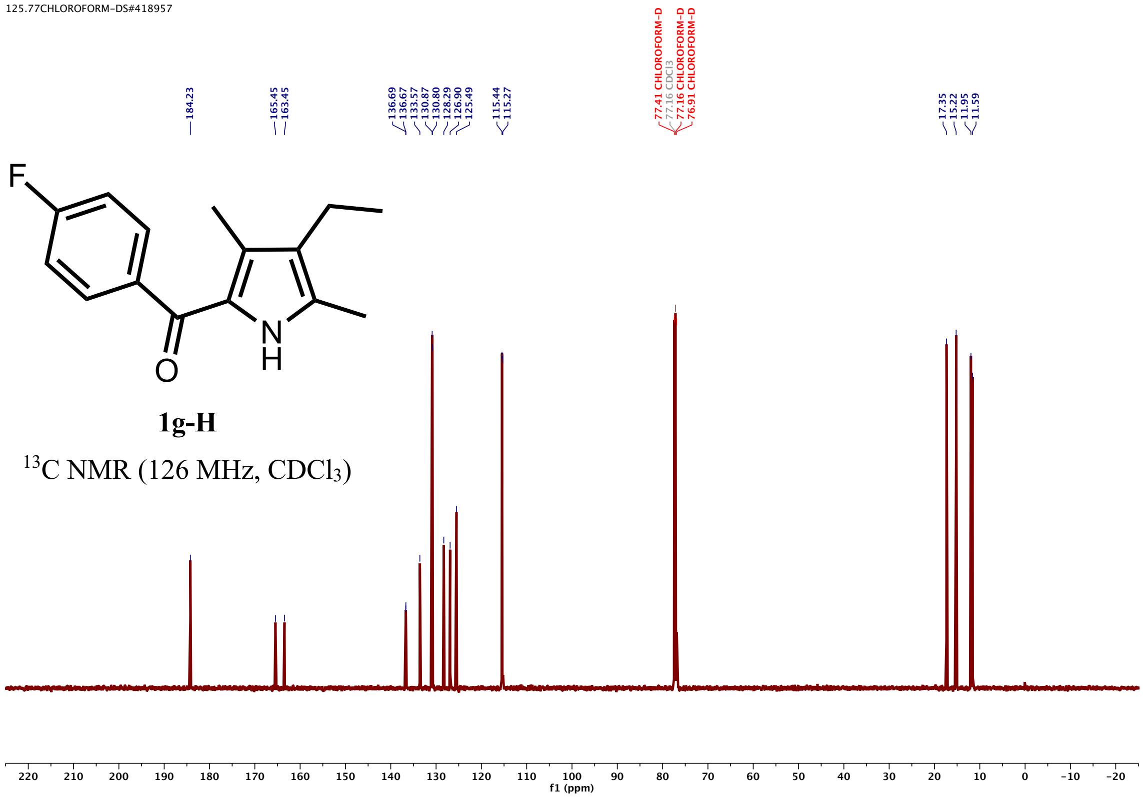

1g-H

^{13}C NMR (126 MHz, $CDCl_3$)

470.62CHLOROFORM-DS#6548

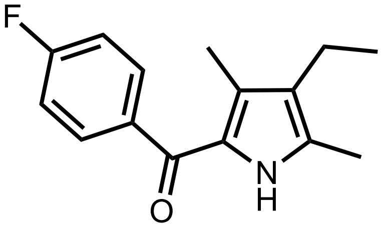

1g-H

^{19}F NMR (471 MHz, $CDCl_3$)

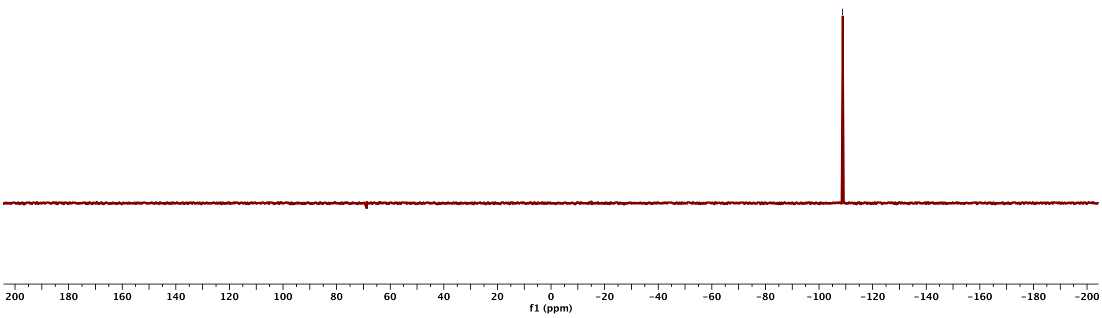

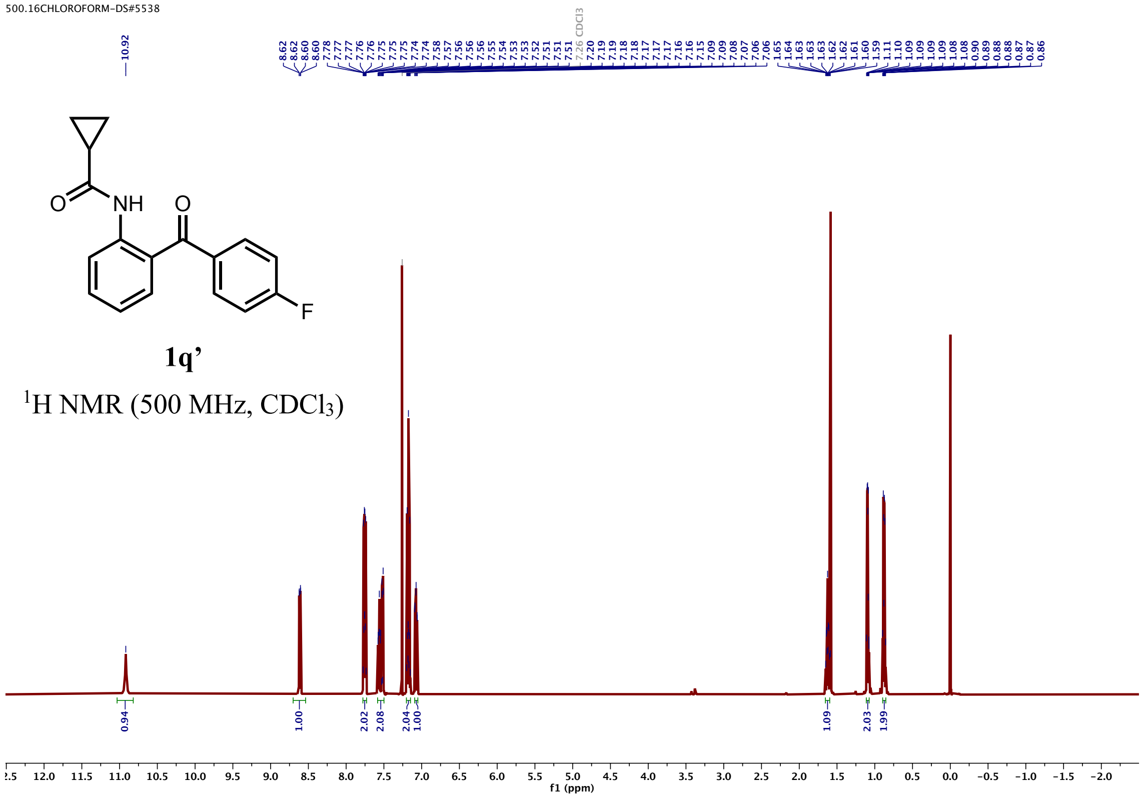
500.16CHLOROFORM-DS#5538
NH
O
O
F
1q’
¹H NMR (500 MHz, CDCl₃)
f1 (ppm)

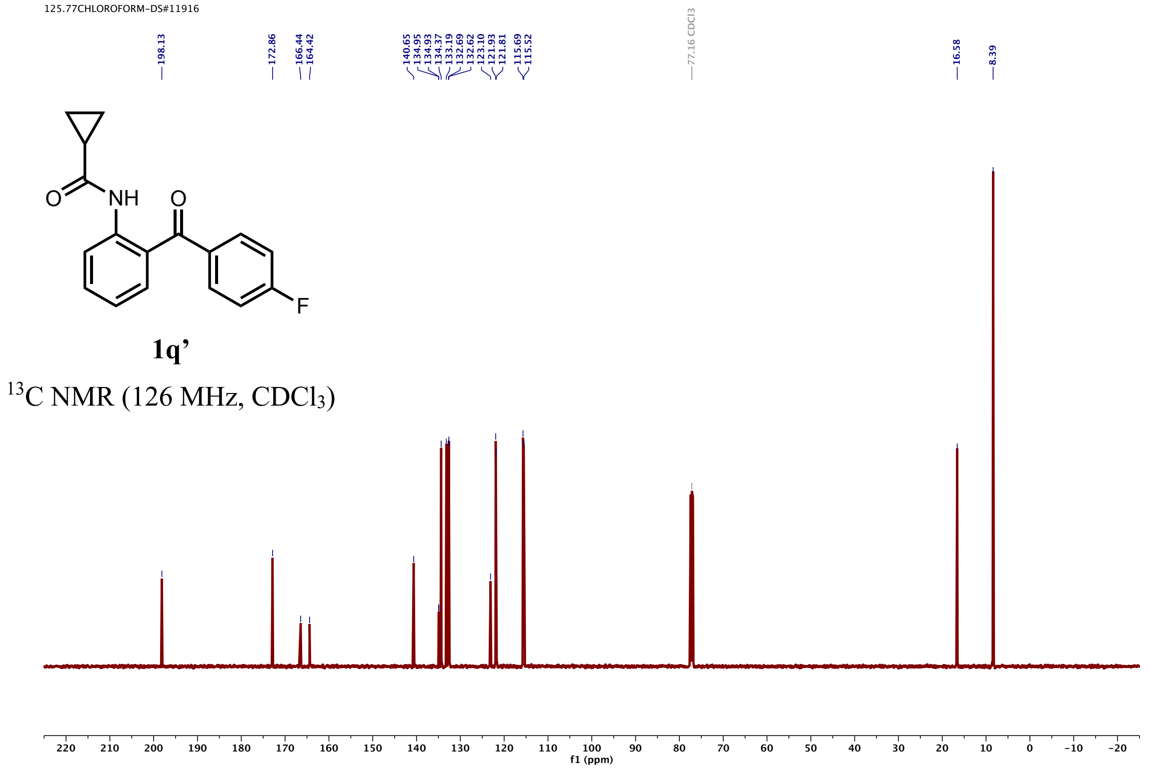
125.77CHLOROFORM-DS#11916
NH
O
O
F
1q’
¹³C NMR (126 MHz, CDCl₃)
f1 (ppm)

470.62CHLOROFORM-DS#7013

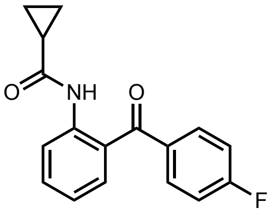

1q'

^{19}F NMR (471 MHz, $CDCl_3$)

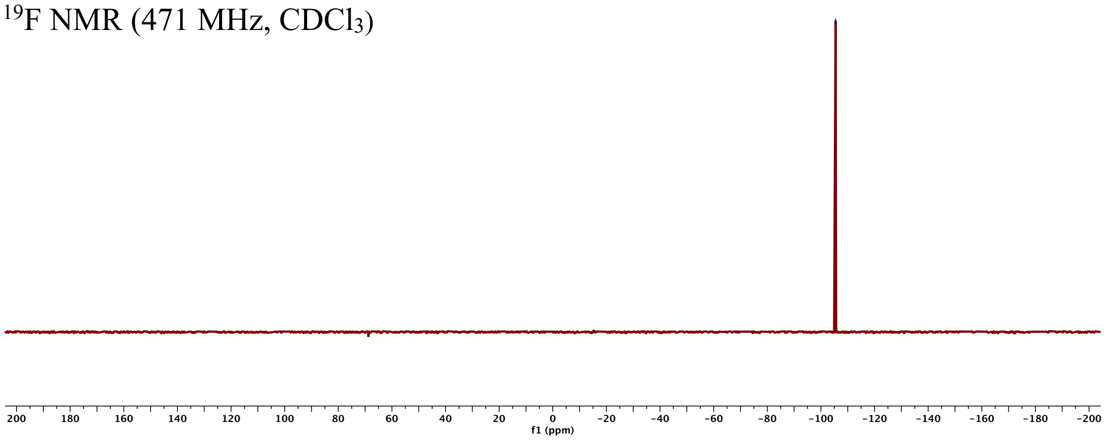

1q

^{1}H NMR (500 MHz, $CDCl_3$)

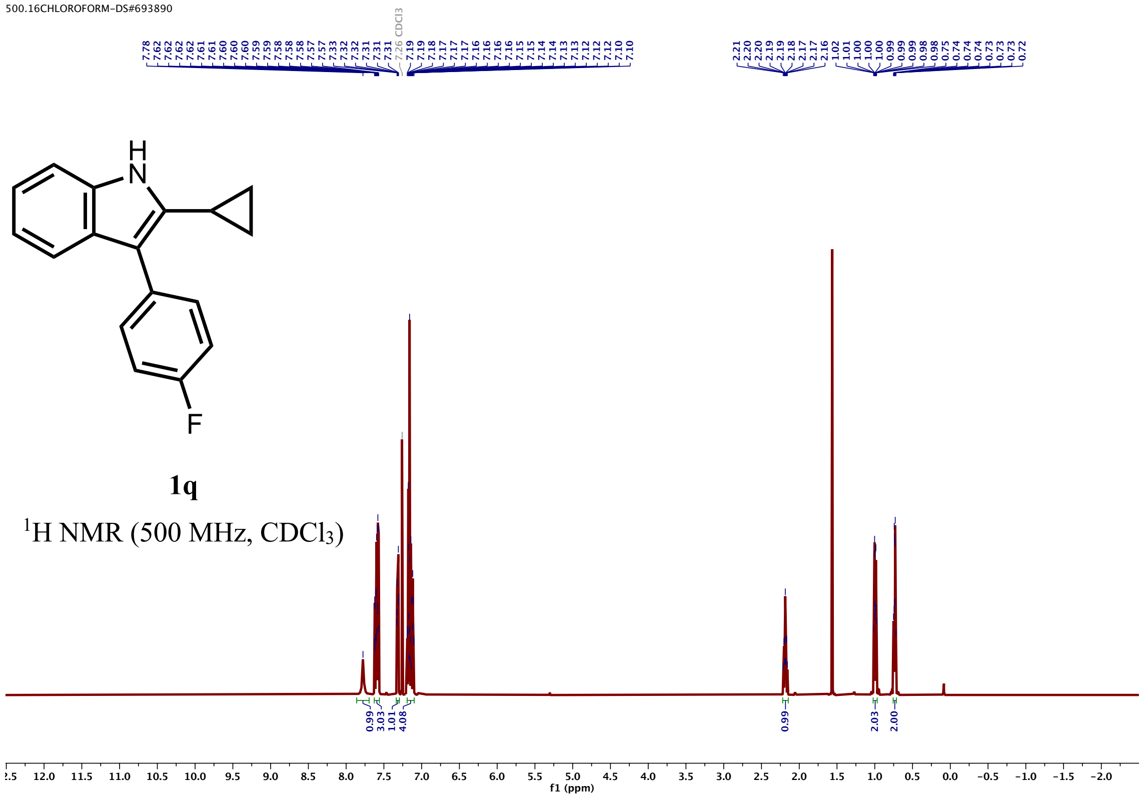

1q

^{13}C NMR (126 MHz, $CDCl_3$)

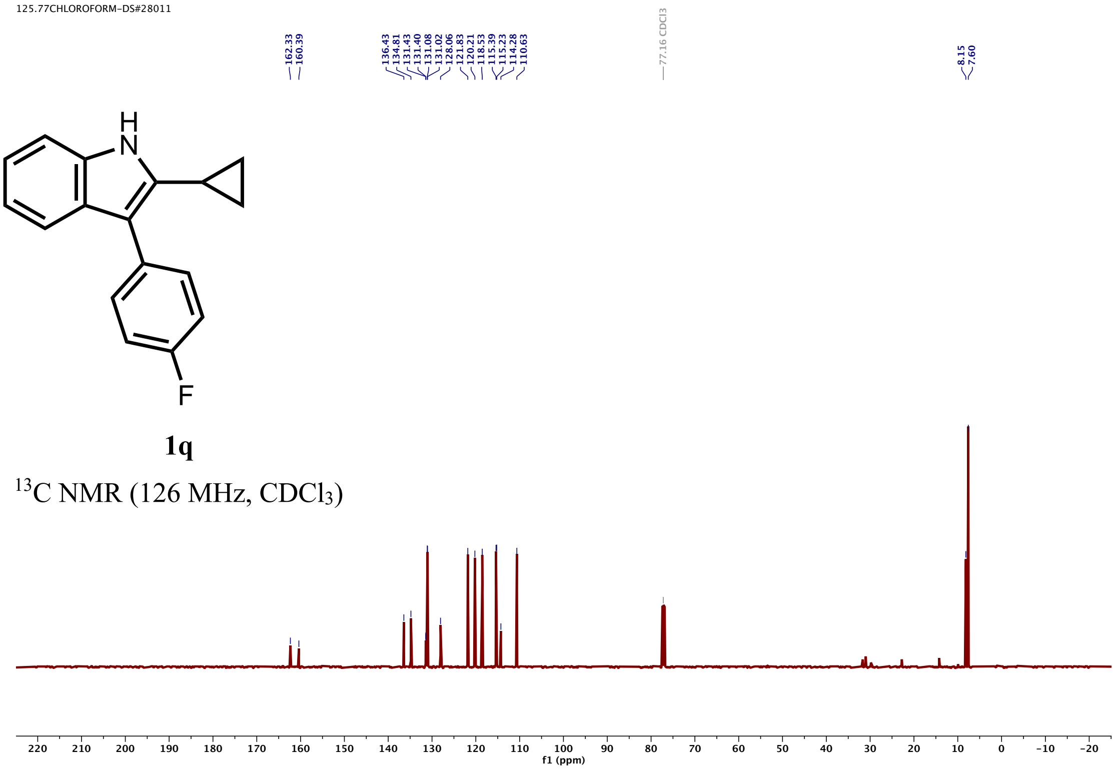

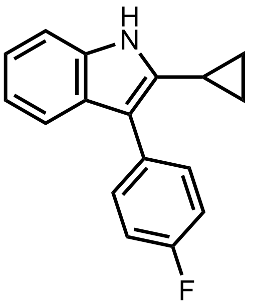

1q

^{19}F NMR (471 MHz, $CDCl_3$)

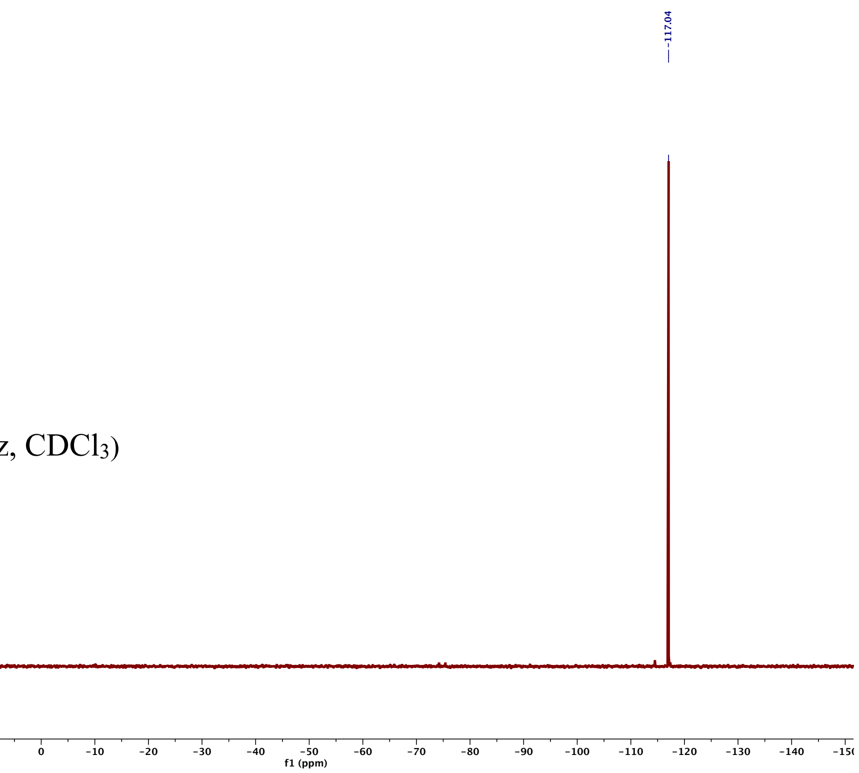

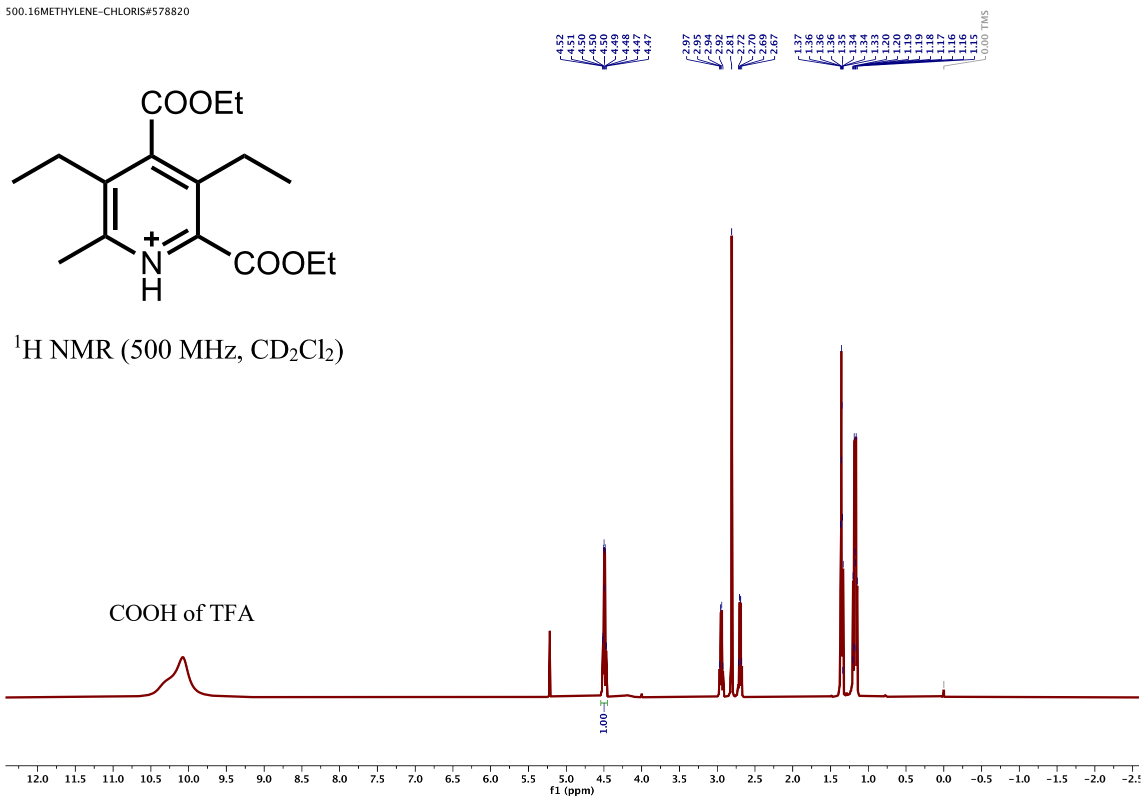
500.16METHYLENE-CHLORIS#578820
COOEt
COOEt
N
H
1H NMR (500 MHz, CD2Cl2)
COOH of TFA
4.52 4.51 4.50 4.50 4.49 4.48 4.47 4.47
2.97 2.95 2.94 2.92 2.81 2.72 2.70 2.69 2.67
1.37 1.36 1.36 1.35 1.34 1.34 1.33 1.20 1.19 1.19 1.18 1.17 1.16 1.16 1.15
0.00 TMS
1.00
f1 (ppm)

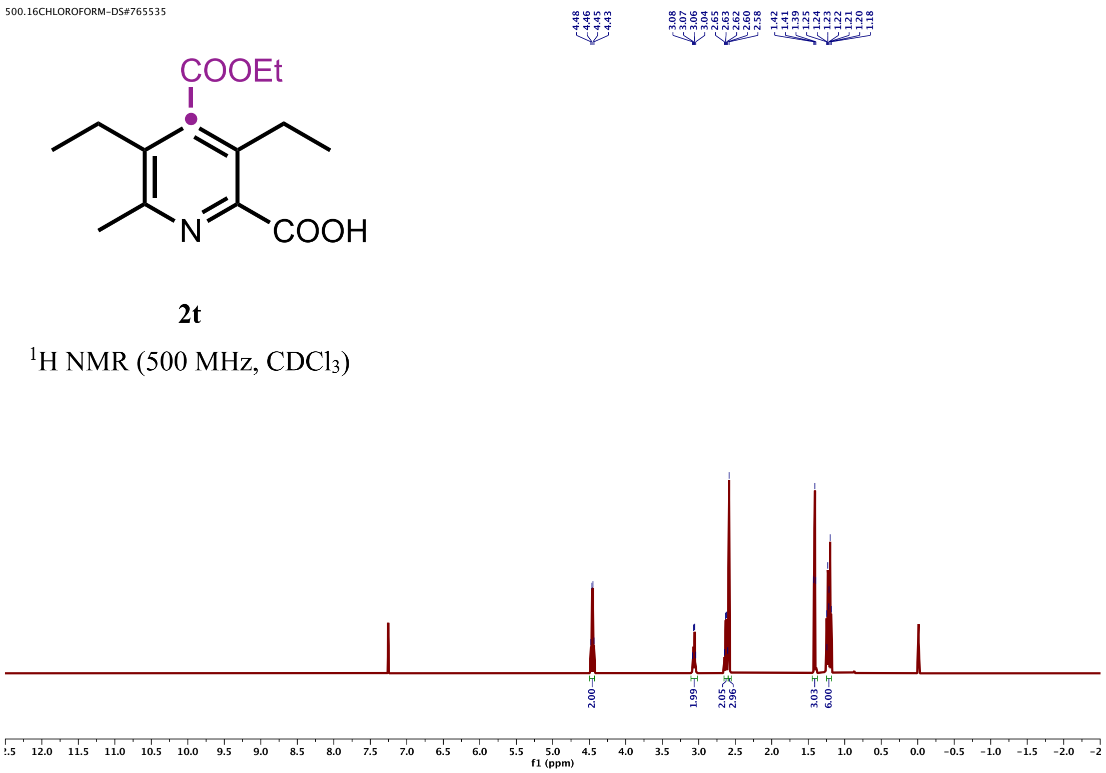

2t

^{1}H NMR (500 MHz, $CDCl_3$)

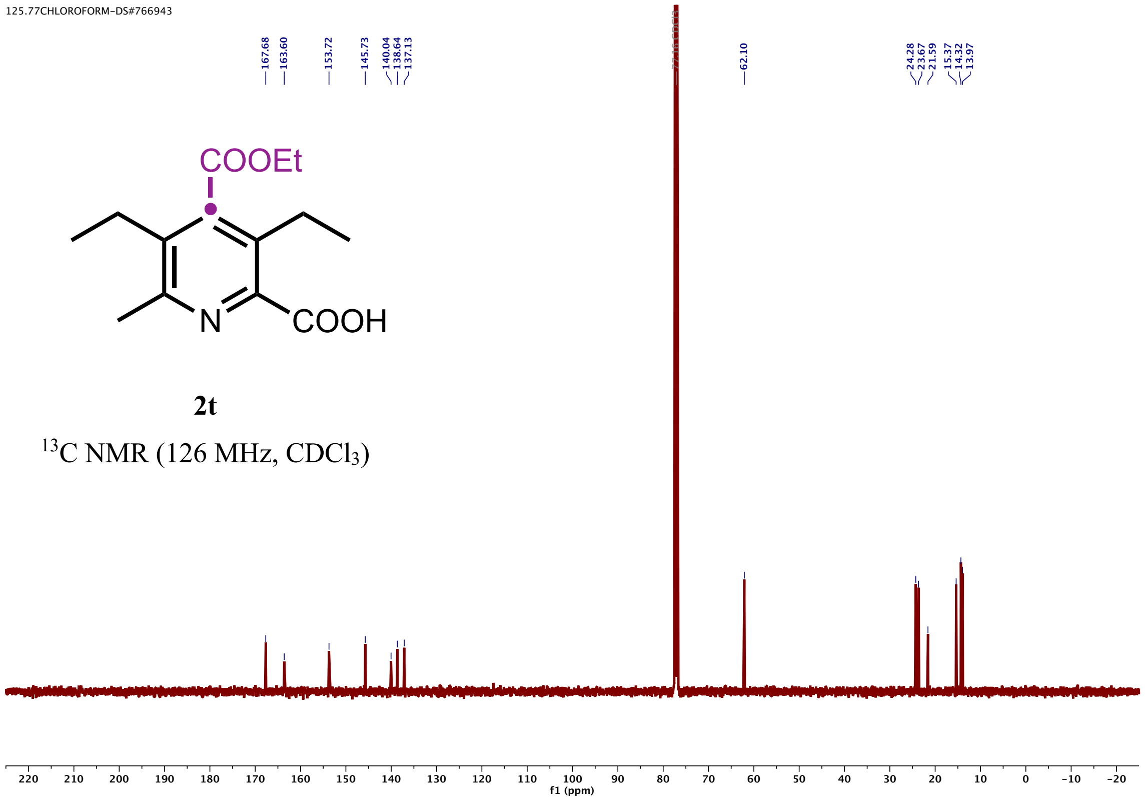

2t

^{13}C NMR (126 MHz, $CDCl_3$)

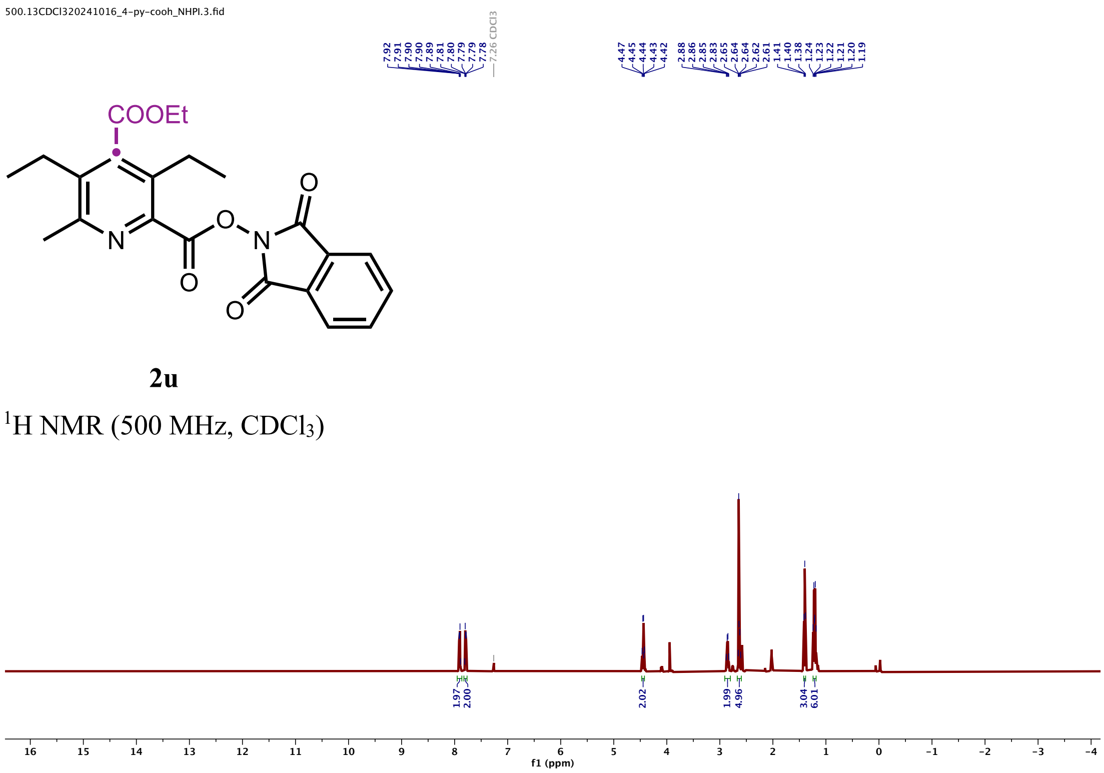

2u

^{1}H NMR (500 MHz, $CDCl_3$)

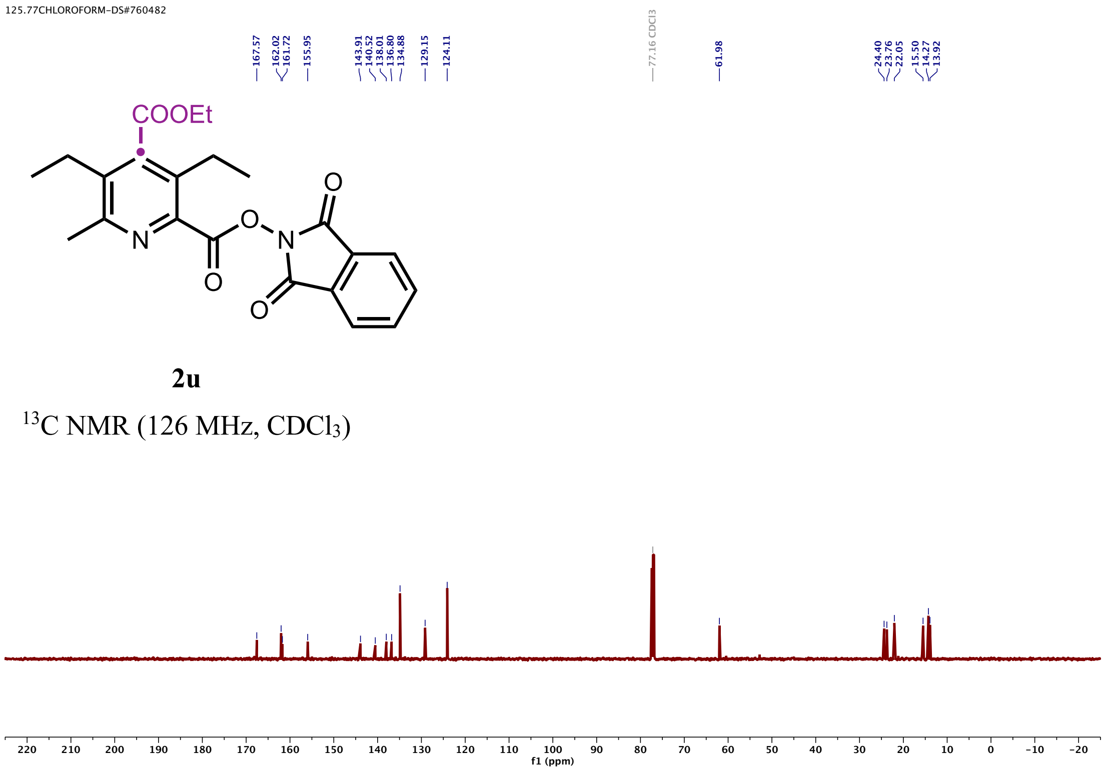

2u

^{13}C NMR (126 MHz, $CDCl_3$)

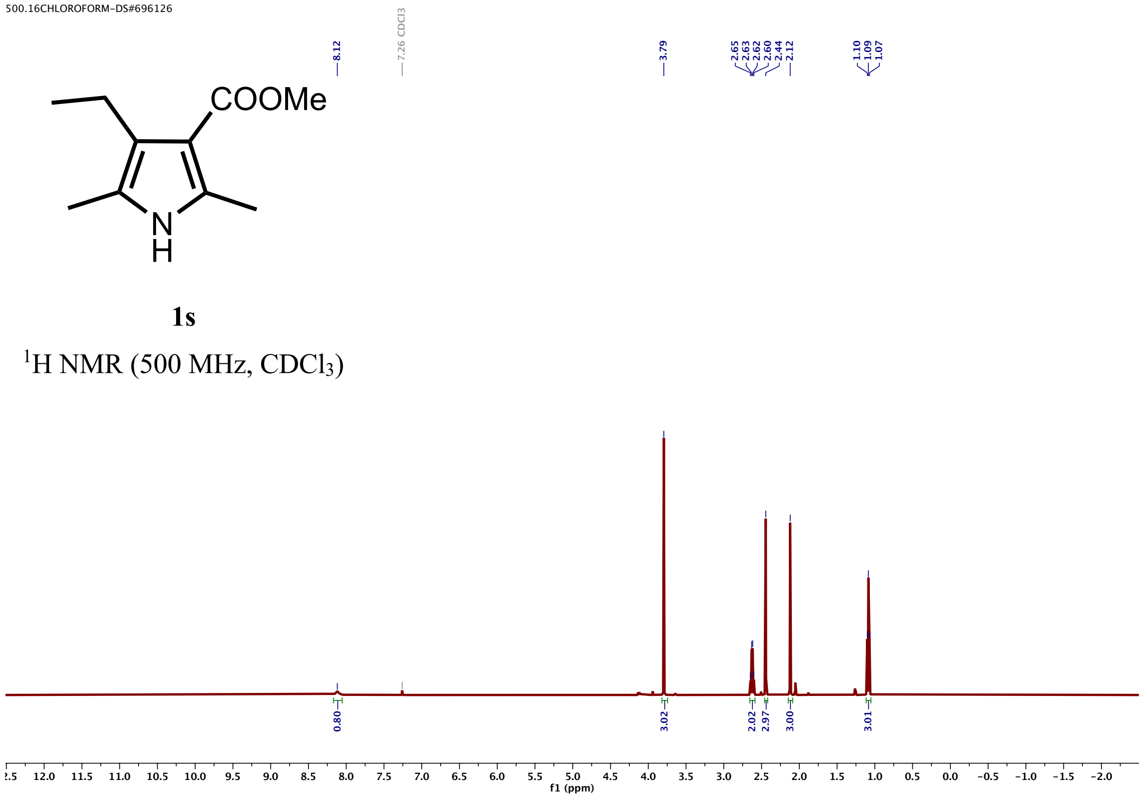

1s

^{1}H NMR (500 MHz, $CDCl_3$)

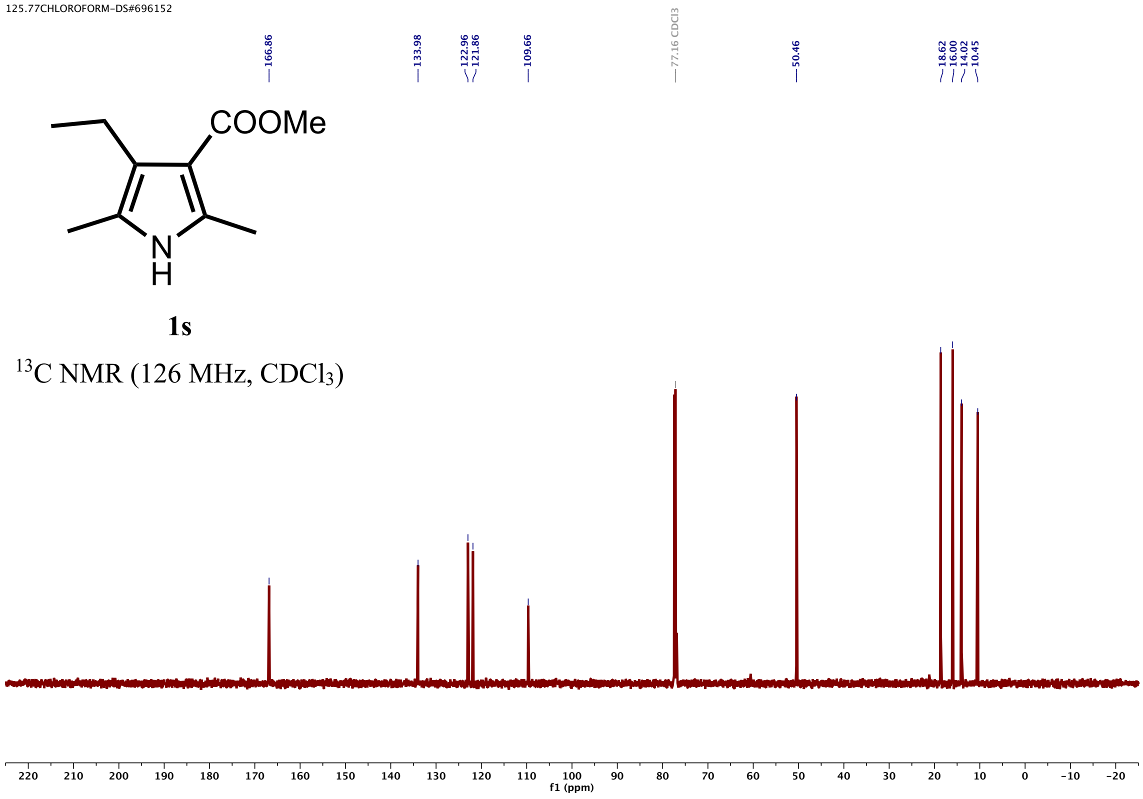

1s

^{13}C NMR (126 MHz, $CDCl_3$)

7. Coordinates for compounds and transition states

7.1 Coordinates for $\Delta G^{\ddagger}$ (298.15 K) of the addition reaction of the ethyl diazoacetate (4.4.1)

Addition carbon 2

C	0.99345000	1.82859300	-0.27606100
C	0.62836700	0.85816300	0.72597800
N	-0.76857500	1.03205900	0.93840300
C	-1.20393200	2.13741000	0.20735500
C	-0.12579700	2.60978400	-0.55024400
C	-2.54077700	2.76903500	0.32600700
H	-2.49740800	3.77080000	-0.09911500
H	-3.30714300	2.20608100	-0.21234000
H	-2.86111300	2.85113400	1.36575300
C	1.08360400	-0.57617200	0.53725100
O	0.36453300	-1.41039000	0.05190300
O	2.34786500	-0.74726800	0.89947900
C	2.90890000	-2.07907400	0.67337400
H	2.27831600	-2.79544600	1.20295600
H	2.84363200	-2.28672000	-0.39615000
C	2.34517100	1.96335300	-0.90423900
H	2.90305600	1.03112100	-0.79244400
H	2.20014400	2.11325000	-1.97827700
C	-0.20242900	3.74864000	-1.52208400
H	-0.68334100	4.60505900	-1.03847700
H	0.80866700	4.07716200	-1.76853500
C	3.17969600	3.13180000	-0.35147500
H	2.63337800	4.07535200	-0.39443400
H	3.47557800	2.97871500	0.68885700
H	4.09486600	3.23629000	-0.93696300
C	-0.95278600	3.37704000	-2.80624000
H	-1.98520800	3.08376600	-2.59919100
H	-0.97736600	4.22605300	-3.49190200
H	-0.46668700	2.54234700	-3.31757400
C	4.32845500	-2.06015500	1.17972500

H	4.92892400	-1.31755600	0.64946500
H	4.36607200	-1.84715100	2.25090100
H	4.78092200	-3.04078000	1.01705800
C	-1.41000900	0.14917100	1.80970200
O	-0.77868000	-0.48841300	2.61112900
C	-2.93838800	-0.06516300	1.71692500
F	-3.57925700	0.89774500	2.40695600
F	-3.36052400	-0.03606600	0.44779900
F	-3.24612500	-1.23398100	2.24591900
C	0.72285400	2.62713500	2.85451600
O	0.78278400	3.68637000	2.27942800
C	1.56070700	1.46472800	2.40890100
O	-0.08588100	2.30769400	3.85079800
H	2.53048300	1.71968700	1.99746400
C	-0.97980700	3.35155600	4.34231300
H	-1.50040600	3.77671400	3.48152100
H	-0.36011100	4.13210300	4.78790800
C	-1.91510800	2.70833700	5.33411500
H	-2.51505000	1.92643900	4.86278400
H	-2.59294800	3.46619900	5.73317200
H	-1.36281700	2.27144200	6.16843500
N	1.69846500	0.47248200	3.33342900
N	1.76550500	-0.43080700	3.98932400

Addition carbon 3

C	0.89584300	2.10023300	-0.48366700
C	0.28525600	1.18807200	0.48362700
N	-1.04049200	1.45224400	0.53572000
C	-1.38512100	2.44713400	-0.39992500
C	-0.26296600	2.82465900	-1.06381800
C	-2.79313000	2.90733600	-0.52306500
H	-2.88670200	3.60820700	-1.35090300
H	-3.47415400	2.07169000	-0.70802100
H	-3.11375200	3.41093500	0.39302300
C	0.82624300	0.28807200	1.49689900
O	0.08433300	-0.24066300	2.30806000

O	2.14195300	0.14389000	1.45925100
C	2.71977900	-0.74931600	2.46115100
H	2.52772900	-0.31005700	3.44238600
H	2.19052300	-1.70133400	2.39960400
C	2.03688600	1.54618000	-1.33896400
H	2.70008200	0.97447000	-0.68670800
H	1.56884700	0.80687000	-1.99720800
C	-0.21818100	3.84604100	-2.16242800
H	-0.95931400	4.62191800	-1.95300500
H	0.74790800	4.35900400	-2.15856400
C	2.85370800	2.51822100	-2.18852600
H	2.26088200	3.00244100	-2.96324000
H	3.34615300	3.30142500	-1.60425200
H	3.64750400	1.96118600	-2.68968900
C	-0.48165900	3.24961600	-3.55057300
H	-1.47601500	2.79903500	-3.59547700
H	-0.42664300	4.02421600	-4.31820900
H	0.24216700	2.47047000	-3.80323600
C	4.19049400	-0.88221800	2.15798500
H	4.35052500	-1.29740800	1.16049900
H	4.70387000	0.07941300	2.22824100
H	4.64537300	-1.56007600	2.88358400
C	-1.97768300	0.85247900	1.46266200
O	-2.50252800	1.49318800	2.31843900
C	-2.44847200	-0.57436100	1.09731000
F	-3.59213900	-0.43364000	0.40046500
F	-1.57489400	-1.21253100	0.31099500
F	-2.68677500	-1.29143200	2.17913100
C	0.72755500	3.78712000	1.70731200
O	-0.16631500	4.57616600	1.55178600
C	1.67425400	3.45291300	0.56844300
O	0.99277900	3.06649700	2.78173000
H	1.78780200	4.27243800	-0.13757700
C	0.08848100	3.22898400	3.92821500
H	-0.92980700	3.09932600	3.55910600
H	0.21055700	4.25105500	4.29078400

C	0.47414600	2.18974200	4.94842500
H	0.33752500	1.18181500	4.55043200
H	-0.16868700	2.29805600	5.82486000
H	1.51027200	2.31664600	5.27100200
N	2.91889400	3.07422600	1.01527300
N	3.90042800	2.61572000	1.29470000

Addition carbon 4

C	0.62654700	2.06352700	-0.12024000
C	0.24003500	0.90007800	0.45327000
N	-1.17176300	0.81153800	0.42518000
C	-1.70163900	1.87903000	-0.17477400
C	-0.60966800	2.78708900	-0.49362600
C	-3.15573200	2.07425400	-0.37906900
H	-3.65881300	1.13454300	-0.61652500
H	-3.60756300	2.47326800	0.53724000
H	-3.33722900	2.78200700	-1.18835600
C	0.92628900	-0.18661500	1.16226700
O	0.27291600	-1.03455400	1.74838500
O	2.23812400	-0.12943000	1.10538900
C	2.97212400	-1.19223300	1.78918000
H	2.67978000	-1.17292500	2.84083400
H	2.65285800	-2.14300900	1.35862100
C	2.02611300	2.56532000	-0.31942000
H	2.62896300	1.74103700	-0.70726100
H	2.02087700	3.33824700	-1.09255300
C	-0.65177500	3.60599700	-1.77130000
H	-1.61672900	4.10970900	-1.86066000
H	0.10695100	4.39293900	-1.72087000
C	2.70144300	3.10478200	0.95007400
H	2.20287200	3.98952100	1.35375300
H	2.72262300	2.34647000	1.73331600
H	3.73030100	3.38996100	0.72271300
C	-0.41811600	2.73394900	-3.00667700
H	-1.18291100	1.95731000	-3.09491900
H	-0.45658500	3.34345100	-3.91120900

H	0.55714700	2.24269200	-2.97213300
C	4.44069900	-0.92172600	1.58568700
H	4.69915400	-0.93164500	0.52470700
H	4.72742400	0.04272700	2.01077700
H	5.02196800	-1.70004000	2.08493100
C	-1.94802200	-0.27203700	1.01945300
O	-2.65341300	-0.08243800	1.95686000
C	-2.00449500	-1.56384300	0.17431000
F	-3.11290200	-1.46888500	-0.58733600
F	-0.95898300	-1.67032300	-0.65340700
F	-2.09085900	-2.63541800	0.93510900
C	-1.28611300	3.45556300	2.17452000
O	-2.45354300	3.48381400	2.47884800
C	-0.76928900	4.13204500	0.91709900
O	-0.31139500	2.82250500	2.79405500
H	0.25571400	4.48815900	0.95194000
C	-0.67802000	2.04931700	3.98714900
H	0.11237800	1.30228600	4.05352000
H	-1.63073700	1.55912000	3.78457700
C	-0.73084600	2.94116100	5.20460900
H	-1.52726500	3.68241100	5.11714000
H	0.22276100	3.45034000	5.36067400
H	-0.93765500	2.32765400	6.08492800
N	-1.62136700	5.14956000	0.56554100
N	-2.41742300	5.69994300	-0.03319900

Addition carbon 5

C	0.48502000	1.75369800	-0.25608900
C	-0.01022000	0.53780900	0.24609600
N	-1.38835900	0.54088100	0.16749100
C	-1.83772300	1.82923100	-0.22025300
C	-0.61115200	2.54535800	-0.57374700
C	-3.05702300	1.89712100	-1.10887000
H	-3.29191300	2.93183000	-1.36104900
H	-2.84168500	1.37327300	-2.04358300
H	-3.93067100	1.43183400	-0.65015000

C	0.60983800	-0.53969500	1.04230800
O	-0.08653500	-1.34605800	1.62688500
O	1.92658700	-0.49446500	1.05910600
C	2.59854700	-1.51280700	1.85945100
H	2.23285100	-1.42194500	2.88412900
H	2.30050500	-2.48861000	1.47121500
C	1.92782800	2.14877800	-0.37690400
H	2.52659100	1.26205800	-0.58559700
H	2.03375600	2.81451700	-1.23760500
C	-0.58353300	3.91246300	-1.17903800
H	-1.46447600	4.48286300	-0.86956400
H	0.28166000	4.45702700	-0.79136600
C	2.45875800	2.84369700	0.88079100
H	1.91521500	3.77249400	1.07753000
H	2.36450900	2.19425800	1.75428300
H	3.51378400	3.09577300	0.75758600
C	-0.51728500	3.87249900	-2.71450300
H	-1.39061100	3.37445800	-3.14182300
H	-0.47738200	4.88658900	-3.11644800
H	0.37185100	3.33661900	-3.05392300
C	4.08091100	-1.26590500	1.74277400
H	4.41305700	-1.34728400	0.70554700
H	4.34681700	-0.27682100	2.12263000
H	4.61789900	-2.01253900	2.33192200
C	-2.27559300	-0.50802300	0.50004700
O	-3.28498800	-0.29833600	1.11709200
C	-2.09688300	-1.86379900	-0.22823100
F	-3.07680300	-1.90812300	-1.15051100
F	-0.93423900	-1.95736700	-0.88591200
F	-2.23503600	-2.88551600	0.59376000
C	-1.28479100	2.75982000	2.36084900
O	-0.71103500	1.81670600	2.84091600
C	-2.46259300	2.55534900	1.43738100
O	-0.99031200	4.03931800	2.50995500
H	-3.11783300	1.73400700	1.73462000
C	0.09275400	4.36901500	3.43679800

H	0.93592700	3.71726700	3.20903800
H	-0.26592000	4.13940300	4.44210400
C	0.41205900	5.82980000	3.25128300
H	0.75896500	6.03198500	2.23506200
H	1.20609800	6.11448500	3.94512600
H	-0.45940700	6.45484200	3.45668700
N	-3.18909200	3.69510900	1.23020600
N	-3.74239600	4.61476000	0.90866800

7.2 Coordinates for transition states of dissociation of N_2 after the addition reaction (Figure 5D)

C	0.86477400	1.94560500	-0.09498000
C	0.31122800	0.93463300	0.89280000
N	-1.12925800	1.00641200	0.60858200
C	-1.38578200	1.92872800	-0.30535500
C	-0.16112200	2.51470000	-0.77807600
C	-2.74057500	2.35492900	-0.71139400
H	-2.78247300	3.44502100	-0.63115200
H	-2.91535800	2.09717100	-1.75978500
H	-3.53495400	1.93313500	-0.10057300
C	0.76562200	-0.50927500	0.55520100
O	-0.01817300	-1.33907900	0.16618600
O	2.05797300	-0.66061900	0.73364200
C	2.58830600	-2.01575000	0.55044800
H	2.01888600	-2.67207500	1.21085600
H	2.39780900	-2.30404600	-0.48458200
C	2.31363400	2.29235600	-0.20778600
H	2.92469200	1.46759300	0.16147900
H	2.54862000	2.40996600	-1.26965900
C	-0.09082500	3.54704700	-1.86294500
H	-0.88755100	4.28304400	-1.71726600
H	0.84686200	4.09820000	-1.77024700
C	2.67424600	3.59422700	0.53277300
H	2.07631400	4.43637800	0.17895700
H	2.52591600	3.50980400	1.61167600
H	3.72626900	3.82866200	0.36239200
C	-0.19186900	2.93219100	-3.26465700
H	-1.13166600	2.39214100	-3.40677900
H	-0.14030700	3.71323200	-4.02515900
H	0.62516300	2.22918000	-3.44458600
C	4.05515200	-1.97012400	0.88982300
H	4.59493500	-1.28728900	0.22976700
H	4.21008100	-1.66151700	1.92593600
H	4.48121900	-2.96816500	0.76595600

C	-2.05046200	0.10407800	1.23823300
O	-2.02770800	-0.08261200	2.41323300
C	-3.03915900	-0.67337200	0.32798800
F	-4.27568000	-0.18798700	0.51729700
F	-2.73330000	-0.54566000	-0.96884900
F	-3.02247900	-1.95006300	0.66075100
C	0.03685800	2.59250700	2.85821600
O	-0.93003900	3.11870400	2.34818700
C	0.71936500	1.35978300	2.31277100
O	0.65624700	2.99813300	3.94785400
H	1.80097300	1.47204400	2.35065500
C	0.06693000	4.10777300	4.69012200
H	0.90769700	4.51401400	5.25042600
H	-0.28806700	4.84558600	3.96954700
C	-1.03759400	3.61182500	5.59525600
H	-0.66515600	2.84614900	6.27893300
H	-1.86867700	3.20202800	5.01764500
H	-1.41755200	4.44634200	6.18980200
N	0.55336200	0.18957300	3.50088200
N	0.65318300	-0.92873100	3.27260700

7.3 Coordinates for transition states on the energy diagram of 1a-TFA

I

C	1.04483800	1.85212700	0.35966300
C	0.37877200	0.61424400	0.90526900
N	-1.02518200	0.82441500	0.50509400
C	-1.13034500	1.91697200	-0.26059300
C	0.13049600	2.59325000	-0.32602700
C	-2.34321600	2.36113100	-0.98032300
H	-2.05458900	3.01065000	-1.80509400
H	-2.92419400	1.52631100	-1.36791000
H	-2.98436700	2.93673800	-0.30582200
C	0.90149500	-0.59811400	0.06585500
O	0.44949500	-0.84649400	-1.02101700
O	1.89357500	-1.20522800	0.67391500
C	2.48409200	-2.35262900	-0.02005900
H	1.68369300	-3.07528000	-0.18687700
H	2.84312100	-2.00023100	-0.98929600
C	2.50078800	2.11614500	0.52797800
H	2.87279000	1.58644500	1.40781500
H	2.64146700	3.18513700	0.71108200
C	0.35536500	3.90850700	-1.01074200
H	-0.07390100	3.87879400	-2.01721800
H	1.42847500	4.05132600	-1.15181200
C	3.31416300	1.69877600	-0.71235800
H	4.36597000	1.93999700	-0.55171400
H	3.23933200	0.62593000	-0.89850200
H	2.97536100	2.22253200	-1.60841800
C	-0.22438200	5.08705700	-0.21995900
H	-1.30607800	4.99606800	-0.08900900
H	0.22925700	5.15207400	0.77229200
H	-0.03217900	6.02582300	-0.74218300
C	3.58531500	-2.88697500	0.85781200
H	4.36007000	-2.13511900	1.02352300
H	3.19508000	-3.21248200	1.82427400
H	4.04578900	-3.74837100	0.36892000

C	-1.94345600	-0.14728700	0.96801700
O	-1.52958800	-1.17429300	1.42239100
C	-3.46378000	0.14543800	0.99217500
F	-3.70340500	1.41458800	1.35985000
F	-3.99613500	-0.05627400	-0.22128400
F	-4.04260700	-0.66426200	1.85506800
C	-0.07527300	1.25386700	3.40654600
O	0.28810000	1.23304700	4.56418100
C	0.58604400	0.46031100	2.38050700
O	-1.10382000	1.98920900	2.94202400
H	1.35809700	-0.21761500	2.71540300
C	-1.78806600	2.83680900	3.90640400
H	-2.78801700	2.95504700	3.48870500
H	-1.85008300	2.29680900	4.85155400
C	-1.07272200	4.16044900	4.05889200
H	-0.99415800	4.67610400	3.09794800
H	-0.07336300	4.01974000	4.47519600
H	-1.63473400	4.80150000	4.74233800

I-TS–II

C	1.51282700	0.60205100	-0.54601100
C	0.78956800	-0.56860800	0.02954000
N	-0.58119900	-0.38184900	-0.40785500
C	-0.71214500	0.82798600	-1.01972600
C	0.55040900	1.44625800	-1.11944900
C	-1.99043700	1.43137400	-1.45991600
H	-1.82556000	2.46531700	-1.75442600
H	-2.40632600	0.88833600	-2.31184800
H	-2.73007000	1.40955300	-0.65875900
C	1.32352600	-1.97123300	-0.26611500
O	0.83417900	-2.66126000	-1.12246900
O	2.37431900	-2.24719800	0.47737400
C	2.99937900	-3.55432300	0.26631500
H	2.23353300	-4.31127600	0.44291200
H	3.30655600	-3.60678500	-0.78002800
C	2.99593200	0.69091900	-0.73776300

H	3.51117900	0.10248900	0.02207400
H	3.31342300	1.72909700	-0.61242100
C	0.82600900	2.78919300	-1.71815400
H	0.12618000	2.98588300	-2.53388200
H	1.82032900	2.77692800	-2.17075200
C	3.38677500	0.17663400	-2.13298400
H	4.46854200	0.24792500	-2.25825800
H	3.09881500	-0.86870400	-2.26944600
H	2.91517200	0.76055500	-2.92647400
C	0.75136900	3.91016000	-0.67103500
H	-0.24553400	3.97845000	-0.22972400
H	1.46509600	3.74076800	0.13959700
H	0.98441100	4.87166100	-1.13161200
C	4.15711700	-3.65700600	1.22493900
H	4.90009500	-2.87904900	1.03487000
H	3.81877400	-3.57874200	2.26019600
H	4.64210300	-4.62731400	1.09756700
C	-1.50729700	-1.30299800	0.13286200
O	-1.17776000	-2.00766900	1.04299100
C	-2.90742700	-1.46242700	-0.50167100
F	-3.74128000	-0.52358100	-0.01940900
F	-2.84784400	-1.33737300	-1.83428200
F	-3.38837500	-2.65093900	-0.20000200
C	0.29000300	0.94629300	2.18983200
O	0.76114900	1.40499200	3.20652200
C	1.12686100	0.10140400	1.31451800
O	-0.96832700	1.12909800	1.77862800
H	2.09458200	-0.13419500	1.73615200
C	-1.84300000	1.88732500	2.66921900
H	-2.83677000	1.49739000	2.44818800
H	-1.57498700	1.63811200	3.69567500
C	-1.73693500	3.37065600	2.40125600
H	-2.01122700	3.60653800	1.36965900
H	-0.72759700	3.73589900	2.60029900
H	-2.42467100	3.90543400	3.06070700

II

C	1.53251700	0.77261100	-0.41637600
C	0.73351200	-0.41359200	0.06848500
N	-0.61449200	-0.18361900	-0.40789800
C	-0.73114000	0.98887800	-1.05184600
C	0.53842200	1.60895300	-1.11614500
C	-2.01854300	1.55537600	-1.52217400
H	-1.93601300	2.63680000	-1.61938800
H	-2.28215500	1.14169700	-2.50034400
H	-2.83268200	1.33150300	-0.83210200
C	1.14442200	-1.85388900	-0.04100500
O	0.41700200	-2.66748300	-0.56413700
O	2.33709300	-2.07507200	0.46790500
C	2.83475800	-3.44991400	0.40267400
H	2.11687700	-4.08148000	0.92862200
H	2.85239800	-3.74374700	-0.64852900
C	2.95127700	0.68012000	-0.94699300
H	3.54966100	0.10075500	-0.24228400
H	3.37685200	1.68819400	-0.95860600
C	0.85444500	2.91859800	-1.73002600
H	0.01754500	3.27509300	-2.33310400
H	1.70096800	2.78322300	-2.41265700
C	3.01993400	0.04622700	-2.33494600
H	4.05338700	0.00838900	-2.68372000
H	2.63651500	-0.97793300	-2.32569500
H	2.44394700	0.60866700	-3.07530800
C	1.23680700	3.96935000	-0.66787500
H	0.40593000	4.16312400	0.01196200
H	2.09493700	3.64439100	-0.07508400
H	1.50178200	4.90517400	-1.16183100
C	4.20080700	-3.46133400	1.03827900
H	4.89382200	-2.81111700	0.49953400
H	4.15256400	-3.14132000	2.08125400
H	4.59944900	-4.47787700	1.01216800
C	-1.64794800	-1.05378900	0.08737200
O	-1.64347200	-1.40986900	1.22306500

C	-2.71317000	-1.54572700	-0.92145500
F	-3.84614900	-0.84726600	-0.74286700
F	-2.30350600	-1.36636600	-2.18363800
F	-2.95745500	-2.82420600	-0.71372200
C	0.48215500	1.42518400	2.01501600
O	0.95629900	1.79988500	3.05743400
C	1.27766500	0.55025000	1.09036400
O	-0.74772600	1.72511900	1.58466900
H	2.16161400	0.17698500	1.59540300
C	-1.59237200	2.48884500	2.49952100
H	-2.60475500	2.18860900	2.22865100
H	-1.37446100	2.15443800	3.51350300
C	-1.37441800	3.97504400	2.33635800
H	-1.59935200	4.29978600	1.31685500
H	-0.34917400	4.25211600	2.58921300
H	-2.04366500	4.51285100	3.01229600

II-TS-III

C	0.22549800	1.68664300	-0.26779500
C	-0.37441200	0.33073800	-0.20833500
N	-1.59665000	0.39213000	-0.72601500
C	-1.89171600	1.67727100	-1.23961600
C	-0.82425000	2.47639500	-0.99931200
C	-3.22552900	1.95404900	-1.83312600
H	-3.25787700	2.97295000	-2.21541600
H	-3.45309700	1.27553800	-2.65938400
H	-4.01027200	1.84811000	-1.07830600
C	0.10926300	-0.98547500	0.28914200
O	-0.63894100	-1.94026300	0.24149700
O	1.34207900	-0.96622600	0.73059700
C	1.88076600	-2.23479300	1.23914700
H	1.22196100	-2.56567900	2.04399600
H	1.82929500	-2.95749000	0.42304000
C	1.67050300	1.83228000	-0.76376900
H	2.32458100	1.26061400	-0.10286900
H	1.94622700	2.88481000	-0.65355200

C	-0.65963800	3.92174400	-1.32579400
H	-1.51519400	4.26225400	-1.91263800
H	0.21986200	4.03493300	-1.97056900
C	1.84087900	1.36991000	-2.20694000
H	1.20980500	1.93651700	-2.89641800
H	2.87676200	1.50076900	-2.52398000
H	1.59508700	0.30958200	-2.31907200
C	-0.49379900	4.80790000	-0.08363500
H	0.38018000	4.51991400	0.50758300
H	-0.35661800	5.84756500	-0.38484100
H	-1.37402100	4.75523400	0.55978900
C	3.28863300	-1.96724000	1.70202200
H	3.91564500	-1.61701200	0.87931000
H	3.30765900	-1.22816800	2.50585500
H	3.71876400	-2.89520700	2.08510600
C	-2.58538700	-0.70268900	-0.67244500
O	-3.45847000	-0.69256200	0.12793100
C	-2.51997900	-1.68418800	-1.86723300
F	-3.42792900	-1.27100500	-2.76795800
F	-1.31946600	-1.65613000	-2.45707900
F	-2.81098900	-2.91026100	-1.48575400
C	-1.02103500	2.13471900	2.11788100
O	-0.79311400	2.40805300	3.27769900
C	0.09438500	1.72981200	1.24470600
O	-2.22258800	2.14163400	1.54633600
H	0.97707600	1.41846700	1.79281200
C	-3.36103100	2.45798600	2.40188800
H	-4.19110000	1.93509100	1.92703600
H	-3.17548900	2.02697600	3.38593400
C	-3.58427300	3.95126800	2.47169400
H	-3.74932800	4.37246400	1.47661600
H	-2.73649000	4.45409300	2.94142700
H	-4.47215000	4.15525300	3.07506800

III

C	0.50256300	1.95750500	-0.40919900
C	-0.36289600	0.80335000	-0.03298500
N	-1.55183100	0.96623400	-0.52168700
C	-1.69501900	2.24663000	-1.17370700
C	-0.50167100	2.86180500	-1.11416600
C	-3.00739200	2.63977800	-1.75178100
H	-2.90582300	3.59736900	-2.26165200
H	-3.36582500	1.90663400	-2.48025800
H	-3.76859200	2.74855700	-0.97444500
C	-0.07783800	-0.42027200	0.77332400
O	-0.97806900	-1.19884700	1.00815100
O	1.16823000	-0.49343200	1.15583400
C	1.53609600	-1.62938900	2.01097600
H	0.87036800	-1.60760100	2.87578800
H	1.34117100	-2.53881600	1.43967900
C	1.62759600	1.47227600	-1.38974900
H	2.29456700	0.82595700	-0.81505200
H	2.19861800	2.36728300	-1.65110100
C	-0.16168500	4.22268700	-1.62884000
H	-0.75842900	4.42876000	-2.52148400
H	0.88460200	4.23661700	-1.94939500
C	1.14172200	0.76840200	-2.65083800
H	0.49619500	1.40883600	-3.25601900
H	2.00062100	0.49505200	-3.26609700
H	0.59772200	-0.15457300	-2.43174200
C	-0.39839100	5.32583600	-0.58601900
H	0.22394100	5.18560900	0.30104200
H	-0.15528200	6.30074100	-1.01227600
H	-1.44303700	5.34486800	-0.26645300
C	2.98652000	-1.45937400	2.37798900
H	3.62221200	-1.46505000	1.49008400
H	3.14813600	-0.52918800	2.92671300
H	3.29060800	-2.29023000	3.01838600
C	-2.69515100	0.06309400	-0.27542900
O	-3.55705800	0.39162900	0.46710000

C	-2.78374100	-1.14865700	-1.23110400
F	-3.54161300	-0.76656600	-2.27205700
F	-1.58019700	-1.48950700	-1.70772300
F	-3.34220100	-2.17824200	-0.63174800
C	0.48031100	2.74438600	2.07372800
O	0.90409300	3.43114200	2.97893400
C	1.13207400	2.62425500	0.78172300
O	-0.65058500	2.00613000	2.13862700
H	2.07777200	3.13723600	0.65689400
C	-1.47101000	2.12409100	3.33493400
H	-1.99447800	1.16938200	3.39311300
H	-0.80430600	2.23252000	4.19085500
C	-2.42520800	3.28901200	3.20375800
H	-3.06643400	3.16897300	2.32678300
H	-1.88238300	4.23372600	3.12984000
H	-3.06539300	3.33636600	4.08792400

III-TS-IV

C	0.39290400	1.89981400	-0.48954800
C	-0.52305600	0.72886800	-0.49501900
N	-1.61042000	1.02819900	-1.18336900
C	-1.60969200	2.34155000	-1.64641000
C	-0.39531600	2.91393800	-1.30193000
C	-2.75071300	2.88907500	-2.42525900
H	-2.44941400	3.80514200	-2.93150600
H	-3.09890700	2.18209200	-3.18256100
H	-3.59600100	3.12049200	-1.76921700
C	-0.45162400	-0.60190700	0.14454600
O	-1.36644100	-1.38696500	-0.01051000
O	0.63986800	-0.78266200	0.84535300
C	0.74848700	-2.05190100	1.56810200
H	-0.11666200	-2.12028400	2.23032700
H	0.68888300	-2.85412400	0.83032900
C	1.86681600	1.63013800	-0.79974200
H	2.40180900	2.58191300	-0.74506200
H	2.26412600	0.99928600	-0.00178600

C	0.12012000	4.23410500	-1.80051500
H	0.03864300	4.22413400	-2.89335200
H	1.18980700	4.30041100	-1.58573700
C	2.06545000	0.97960200	-2.16460500
H	1.66286200	1.60099600	-2.96962100
H	3.12772900	0.82966000	-2.36366600
H	1.58177600	-0.00058500	-2.22033900
C	-0.60021900	5.46465100	-1.24096900
H	-1.67021600	5.44325400	-1.45841700
H	-0.48550800	5.53560700	-0.15679700
H	-0.18806200	6.37284300	-1.68421000
C	2.05877800	-2.03297400	2.31093100
H	2.90198100	-1.94444600	1.62244900
H	2.09495900	-1.20794600	3.02552000
H	2.16915600	-2.96812700	2.86427600
C	-2.78019500	0.12761900	-1.32581100
O	-3.79087600	0.36204700	-0.75778300
C	-2.64586700	-0.90786300	-2.46664600
F	-3.23118400	-0.36320200	-3.54955600
F	-1.36591700	-1.14309900	-2.77757200
F	-3.25507500	-2.03309500	-2.16112300
C	-0.92086700	2.50331200	1.67382300
O	-1.57733400	1.47052000	1.67322900
C	0.08557000	2.80847600	0.65794800
O	-1.01779600	3.46799200	2.57453400
H	0.69484000	3.69458500	0.78130400
C	-1.94742700	3.26402600	3.67861300
H	-2.16756000	4.27570900	4.01630700
H	-2.85178500	2.80333500	3.27884200
C	-1.31546200	2.42524200	4.76667900
H	-0.39244300	2.88500700	5.12657800
H	-1.10156500	1.41501800	4.41217500
H	-2.00856400	2.34789400	5.60795200

IV

C	1.44872900	0.93803400	-0.19163600
C	0.60182600	-0.25774600	-0.15878100
N	-0.61626300	0.02250300	-0.75062800
C	-0.71026000	1.28248400	-1.16641000
C	0.57419600	1.97786900	-0.92952800
C	-1.90542600	1.83005000	-1.83792100
H	-1.85738900	2.91731600	-1.85928700
H	-1.96733700	1.46163100	-2.86867000
H	-2.81119300	1.52507800	-1.31005200
C	0.77356900	-1.62755800	0.31815400
O	-0.14061300	-2.42758900	0.17023600
O	1.93191100	-1.87238500	0.88293300
C	2.14951700	-3.23494700	1.37159800
H	1.35623400	-3.45693400	2.08784800
H	2.04671100	-3.90855200	0.51868600
C	2.96009900	0.82888900	-0.32368100
H	3.30565600	1.67470800	-0.92518900
H	3.19461600	-0.06996800	-0.90006900
C	1.07833700	2.96406800	-1.96252500
H	0.35452000	3.78122200	-2.04865800
H	1.99292600	3.42252200	-1.57795500
C	3.70150800	0.81321000	1.01130300
H	4.77595500	0.72322000	0.83981800
H	3.53213900	1.73502100	1.57363800
H	3.38563400	-0.02612800	1.63131100
C	1.33916400	2.32283900	-3.32326000
H	0.42932600	1.89518500	-3.75469300
H	1.71533900	3.06361400	-4.03074700
H	2.08191800	1.52392500	-3.24925200
C	3.52418100	-3.27417000	1.98711100
H	4.29283900	-3.02482800	1.25255200
H	3.60040500	-2.58249800	2.82887700
H	3.72115400	-4.28275200	2.35702800
C	-1.74511200	-0.95019000	-0.78704600
O	-2.65200200	-0.86500300	-0.03338100

C	-1.73702100	-1.83729300	-2.05153800
F	-2.47664600	-1.19578400	-2.97773600
F	-0.50303700	-1.97533700	-2.54961400
F	-2.26608000	-3.01683000	-1.81015000
C	-0.22386500	2.06513400	1.55090100
O	-1.39033000	1.90702500	1.24677300
C	0.87320100	2.15894300	0.53650500
O	0.24377500	2.16700600	2.77905600
H	1.63505600	2.89669200	0.76708300
C	-0.71553500	2.04246200	3.87232200
H	-0.21953700	2.53882200	4.70488700
H	-1.61545400	2.59355700	3.59686800
C	-1.00544400	0.58696100	4.16271000
H	-0.08450800	0.04602700	4.39355200
H	-1.50925600	0.10844800	3.32002900
H	-1.66569800	0.51708800	5.03054400

IV-TS-pro3,4

C	0.60749000	1.96355600	0.05905000
C	-0.23192200	0.87563600	-0.14434800
N	-1.46816800	1.15654700	-0.75998100
C	-1.52307900	2.33358900	-1.40149100
C	-0.39195200	3.18601700	-1.11942700
C	-2.64948300	2.71970500	-2.28283500
H	-2.49194500	3.71861900	-2.68318000
H	-2.76642900	2.01527700	-3.11093600
H	-3.57974200	2.73084700	-1.70594400
C	0.02957100	-0.57720100	-0.01567800
O	-0.78126000	-1.37543200	-0.44938100
O	1.14679500	-0.87258800	0.60736900
C	1.44843400	-2.29451500	0.77262500
H	0.63073200	-2.73789100	1.34382600
H	1.46202400	-2.74620200	-0.22121500
C	2.11342900	1.87930600	0.11732200
H	2.50209600	2.88369900	0.30121500
H	2.38039100	1.28390600	0.99550000

C	0.22310200	4.10066600	-2.13708200
H	0.01936700	3.73135300	-3.14514300
H	1.31019300	4.06789800	-2.01040200
C	2.76265100	1.29130000	-1.13617500
H	2.44508400	0.26220800	-1.31177800
H	2.51862800	1.87883800	-2.02516800
H	3.84883000	1.28760300	-1.02703000
C	-0.25803800	5.55082300	-1.97580700
H	-1.33855500	5.64000700	-2.10774400
H	-0.00877000	5.94530600	-0.98794700
H	0.22616400	6.18309900	-2.72224000
C	2.77644700	-2.38937300	1.47819300
H	3.57085300	-1.92584500	0.88906000
H	2.73648000	-1.91086600	2.45902300
H	3.03079600	-3.44164000	1.62285700
C	-2.60596000	0.25953000	-0.52227300
O	-3.06480400	0.14929400	0.56688600
C	-3.20275200	-0.45875600	-1.75519400
F	-4.33759300	0.16936400	-2.10612100
F	-2.36479500	-0.41313500	-2.79872800
F	-3.48216500	-1.71263700	-1.46048000
C	-1.24560200	3.33199900	1.28330900
O	-2.39550200	3.42673700	0.91113900
C	-0.07516700	3.28858900	0.31506700
O	-0.83019100	3.25761300	2.53028000
H	0.65695500	4.05798100	0.55143600
C	-1.84700900	3.23239000	3.58017700
H	-1.32145500	3.61415500	4.45411600
H	-2.64125200	3.92332700	3.29600200
C	-2.35806900	1.82453000	3.78417400
H	-1.54014200	1.14774300	4.04268200
H	-2.86441700	1.45244100	2.89140100
H	-3.07561400	1.81854900	4.60836700

II-TS-pro2,3

C	1.05623400	2.16363600	0.06174200
C	0.04184900	0.57653400	0.66338000
N	-1.22745900	0.90471000	0.11011600
C	-1.27955200	2.10081000	-0.50685500
C	0.01163600	2.67964900	-0.70494600
C	-2.54708200	2.72782500	-0.95517000
H	-2.43147300	3.81069200	-0.98268300
H	-2.81154200	2.39001900	-1.96254100
H	-3.37567900	2.48172500	-0.29166500
C	0.51882800	-0.79683100	0.35012400
O	-0.14181600	-1.53966800	-0.34238100
O	1.68989100	-1.05405800	0.89839200
C	2.24770400	-2.38333100	0.65269300
H	1.53139200	-3.11290200	1.03479100
H	2.32893600	-2.51158300	-0.42890400
C	2.50255700	2.25983200	-0.35030600
H	3.14161700	2.05262300	0.50920800
H	2.69565000	3.30144800	-0.62871900
C	0.21137600	3.76230400	-1.73098000
H	-0.58674700	3.72857600	-2.47525600
H	1.13856100	3.57067500	-2.27504500
C	2.86698900	1.33378200	-1.51664500
H	3.90865400	1.48918100	-1.80304900
H	2.75068300	0.28494100	-1.23576500
H	2.24316400	1.52033100	-2.39400400
C	0.27256300	5.15401300	-1.08187900
H	-0.64760100	5.38651200	-0.53963600
H	1.10070500	5.22439000	-0.37219300
H	0.41480800	5.91855800	-1.84767000
C	3.57979400	-2.44700900	1.35371700
H	4.27048500	-1.69760600	0.96051500
H	3.46618000	-2.29426800	2.42893000
H	4.02156100	-3.43307900	1.19433300
C	-2.28675200	-0.00216900	0.44612800
O	-2.30803600	-0.53578600	1.51187000

C	-3.33550700	-0.34229800	-0.64138800
F	-4.45009700	0.37725200	-0.43423600
F	-2.87493500	-0.06283500	-1.86789700
F	-3.63317000	-1.62471600	-0.56851800
C	0.07534700	2.50956700	2.44419000
O	0.72679700	3.06029200	3.29355000
C	0.73976300	1.57375800	1.44752900
O	-1.23142300	2.66094300	2.24481700
H	1.64698300	1.17744100	1.89315200
C	-1.93102400	3.58213700	3.13692700
H	-2.95444300	3.20832000	3.14428500
H	-1.49799700	3.48118500	4.13188400
C	-1.83720800	4.99841900	2.61758800
H	-2.26254900	5.07931900	1.61342400
H	-0.80136800	5.34352400	2.60092600
H	-2.40224900	5.66367900	3.27473600

pro3,4

C	1.22307900	0.28200100	0.37062000
C	0.60065900	-0.73736400	-0.26453700
N	-0.46192700	-0.50750200	-1.16246500
C	-0.94247900	0.72758300	-1.46634600
C	-0.34182800	1.83624500	-0.88344100
C	-2.10288600	0.84772500	-2.40925000
H	-2.24873200	1.88255800	-2.70181500
H	-1.96246200	0.26220600	-3.31749200
H	-3.01418500	0.49103800	-1.92231300
C	0.86414600	-2.19222900	-0.10612700
O	0.14212100	-2.99057900	-0.68624900
O	1.86252100	-2.52512100	0.66344400
C	2.11712400	-3.96033400	0.83594200
H	1.20771500	-4.40471900	1.24386800
H	2.29876800	-4.37984400	-0.15483300
C	2.36792600	0.14773400	1.33881700
H	2.87801500	1.11392000	1.38171900
H	3.08494000	-0.58117500	0.96223400

C	-0.70742200	3.24224400	-1.24764500
H	-1.79002900	3.34846900	-1.34377600
H	-0.40124500	3.91033000	-0.43866100
C	1.93387100	-0.25399800	2.75853400
H	2.81487800	-0.30092900	3.40154300
H	1.23901300	0.46573500	3.19152600
H	1.45543900	-1.23323400	2.76495500
C	-0.01080700	3.68747200	-2.54724200
H	-0.31660200	3.07809200	-3.40066200
H	-0.26538200	4.72635200	-2.76335400
H	1.07603300	3.61949800	-2.45562600
C	3.30143000	-4.09129400	1.75764200
H	4.18928000	-3.62094100	1.32966900
H	3.09561300	-3.64371800	2.73228400
H	3.51852500	-5.15085300	1.90964100
C	-1.11352300	-1.67886800	-1.84861700
O	-2.25146100	-1.93808700	-1.63658200
C	-0.35179400	-2.16624000	-3.10874500
F	-0.84408400	-1.50578800	-4.17268200
F	0.95825500	-1.88032000	-3.03652000
F	-0.51892900	-3.45915200	-3.29649700
C	0.18552200	2.25798900	1.47013100
O	-0.82255000	1.83832500	1.97966000
C	0.73324200	1.67644400	0.13403000
O	0.94366100	3.24615100	1.91001400
H	1.58715200	2.31089400	-0.14079400
C	0.51297600	3.93513600	3.12739100
H	0.99602600	4.90778200	3.04632200
H	-0.57013400	4.05465300	3.07688000
C	0.94394700	3.18827700	4.36812200
H	2.02146800	3.00841400	4.36320300
H	0.41549500	2.23847700	4.46555900
H	0.70680900	3.79404300	5.24621800

pro2,3

C	1.51742800	1.60470500	-0.86566700
C	0.49633900	-0.52180400	-0.11585400
N	-0.66601300	-0.13141000	-0.70483300
C	-0.80906700	1.06897000	-1.30474100
C	0.33618500	1.91407500	-1.48091200
C	-2.14028700	1.46165700	-1.85614700
H	-2.28575700	2.53364600	-1.73028700
H	-2.17876100	1.24864600	-2.93031600
H	-2.96918500	0.94789600	-1.37733200
C	0.50273100	-1.87369000	0.43502000
O	-0.46843400	-2.60430500	0.28742600
O	1.60976700	-2.17465600	1.07075900
C	1.69352600	-3.51077000	1.66594600
H	0.85444700	-3.61172200	2.35684800
H	1.57054300	-4.23491200	0.85839300
C	2.79267400	2.36150600	-1.05555000
H	3.34247900	2.36536600	-0.11040300
H	2.59445200	3.40004800	-1.32036500
C	0.20057800	3.15029800	-2.34559400
H	-0.54791500	2.98689200	-3.12261700
H	1.13888600	3.30686800	-2.88045000
C	3.65435200	1.69959100	-2.14758400
H	4.59831100	2.23807200	-2.24461400
H	3.88900600	0.66043700	-1.90220100
H	3.15028300	1.71375900	-3.11676000
C	-0.14693000	4.40380400	-1.53426900
H	-1.09275400	4.29297500	-0.99657900
H	0.62525300	4.62059400	-0.79177300
H	-0.23659300	5.27077400	-2.19137200
C	3.03268500	-3.61214700	2.34808100
H	3.84961400	-3.49007000	1.63386200
H	3.13341700	-2.85998700	3.13335200
H	3.12556700	-4.59912900	2.80658000
C	-1.84464700	-1.08180900	-0.64763700
O	-2.71952700	-0.89719500	0.12778400

C	-1.94582300	-2.01172000	-1.87971300
F	-2.71392400	-1.38597700	-2.79140400
F	-0.74668100	-2.21673600	-2.43738000
F	-2.50374400	-3.16006800	-1.56200000
C	1.57432900	1.01969000	1.53809800
O	2.53524000	1.54121800	2.03647700
C	1.59076700	0.44563100	0.08717100
O	0.37392200	0.87797200	2.07993900
H	2.55769000	-0.05934600	0.00303000
C	0.17076600	1.43189800	3.41769900
H	-0.65926700	0.84713700	3.81185000
H	1.07116200	1.23763400	4.00125000
C	-0.15222100	2.90576800	3.33655400
H	-1.03585400	3.07833300	2.71746000
H	0.68974000	3.47309500	2.93469200
H	-0.36317600	3.28348300	4.33988700

7.4 Coordinates for transition states on the energy diagram of 1j-H

I

C	0.78257600	0.07761200	-0.28024300
C	0.63214400	-1.30595600	0.35292300
N	-0.82456600	-1.37180300	0.49412300
C	-1.43143000	-0.28551900	0.06396600
C	-0.44977400	0.62716400	-0.47904600
H	-1.28108600	-2.09602700	1.03274700
C	1.14072900	-2.36210800	-0.63792500
C	0.28506300	-2.90753200	-1.59334200
C	2.48620600	-2.72488800	-0.63838600
C	0.76533600	-3.82122100	-2.52347100
C	2.96316200	-3.63781300	-1.57016200
C	2.10448000	-4.19093900	-2.51242900
H	-0.76133900	-2.62488500	-1.62062100
H	3.17752600	-2.28892000	0.07329000
H	0.08812700	-4.24527100	-3.25671900
H	4.01146200	-3.91482400	-1.55771300
H	2.47840100	-4.90614300	-3.23667600
C	2.07854000	0.61266300	-0.69272600
C	3.19329400	0.54544900	0.15246000
C	2.21638300	1.19438000	-1.95972500
C	4.41504500	1.05921800	-0.25686100
C	3.44408700	1.68817300	-2.37101200
C	4.54354200	1.62647700	-1.52009900
H	3.10619000	0.11401000	1.14369100
H	1.36236900	1.24217000	-2.62465200
H	5.26752600	1.01568000	0.41179000
H	3.54387500	2.12333100	-3.35915600
H	5.50137300	2.02063900	-1.84199700
C	-0.76154300	1.93338700	-1.09470900
C	-1.61617900	2.01529600	-2.19589900
C	-0.19685300	3.09938900	-0.57270600
C	-1.90453900	3.24908800	-2.76468000
C	-0.49057700	4.33160300	-1.14163200

C	-1.34468300	4.40799400	-2.23703500
H	-2.05384700	1.11188400	-2.60907800
H	0.47760200	3.03431700	0.27469500
H	-2.56606500	3.30551900	-3.62223800
H	-0.05037300	5.23349400	-0.73045600
H	-1.57215800	5.37089900	-2.68132200
C	-2.87315300	-0.12670000	0.17921700
C	-3.70352000	-1.25542400	0.11671700
C	-3.43350900	1.13870200	0.39897500
C	-5.07287900	-1.11921100	0.27211600
C	-4.80319500	1.26227400	0.57299500
C	-5.62187500	0.13917400	0.50561500
H	-3.28415400	-2.23482300	-0.09385800
H	-2.79825500	2.01276200	0.45342100
H	-5.71404300	-1.99078700	0.20620300
H	-5.23426800	2.23968800	0.75745000
H	-6.69416100	0.24425800	0.63066400
C	1.02794000	-0.57396800	2.85133200
O	1.62896300	-0.70660600	3.90036600
C	1.25462800	-1.45853000	1.71582300
O	0.12028600	0.38746200	2.62127700
H	1.92274600	-2.28957900	1.89952300
C	-0.11581000	1.31727500	3.70636100
H	0.81869000	1.84431900	3.91221300
H	-0.38312800	0.74552900	4.59729500
C	-1.21858600	2.25059100	3.26927100
H	-0.93702200	2.79872200	2.36653600
H	-1.41726900	2.97718700	4.06019100
H	-2.14230800	1.70097800	3.07248500

I-TS-II

C	0.92004700	0.24568000	-0.48702400
C	0.68115600	-1.11941100	0.11380700
N	-0.76082100	-1.13394900	0.24910500
C	-1.31267200	0.03613700	-0.04498900
C	-0.30095700	0.92682200	-0.53432700

H	-1.22779400	-1.82987300	0.81537800
C	1.25182700	-2.35503000	-0.52740700
C	0.78627800	-2.72426200	-1.79066900
C	2.21299600	-3.12965400	0.11138700
C	1.28899400	-3.85654000	-2.41280600
C	2.71715400	-4.26485000	-0.51581200
C	2.25932400	-4.62650000	-1.77566300
H	0.02920200	-2.12238500	-2.28580600
H	2.56904300	-2.85809400	1.09936800
H	0.92666500	-4.14014500	-3.39481400
H	3.46726300	-4.86647900	-0.01469000
H	2.65440900	-5.51111700	-2.26300900
C	2.17977300	0.65885700	-1.14805900
C	3.17600800	1.36908100	-0.47702300
C	2.34321600	0.34801700	-2.49922300
C	4.32796200	1.75509400	-1.14993000
C	3.49551500	0.73939400	-3.16768900
C	4.48937500	1.44037700	-2.49434900
H	3.05159000	1.62347500	0.57102200
H	1.56935500	-0.19767100	-3.02707400
H	5.09895200	2.30471900	-0.62132400
H	3.61641900	0.49429300	-4.21711300
H	5.38956900	1.74293400	-3.01810600
C	-0.51920400	2.26716500	-1.08885600
C	-1.52741200	2.48528000	-2.03573300
C	0.29482200	3.33348700	-0.69462300
C	-1.71428400	3.74786200	-2.57696200
C	0.09746700	4.59734000	-1.23303700
C	-0.90433700	4.80614700	-2.17468800
H	-2.15582300	1.66105900	-2.35654900
H	1.07328600	3.17354800	0.04217600
H	-2.49074800	3.90696700	-3.31708500
H	0.72778700	5.42078600	-0.91606200
H	-1.05391800	5.79378500	-2.59723800
C	-2.72810800	0.28260500	0.18959300
C	-3.64732200	-0.76832200	0.07339800

C	-3.16442000	1.54922300	0.59922700
C	-4.98502400	-0.55449900	0.36512400
C	-4.50216600	1.75031800	0.90243400
C	-5.41158600	0.70301400	0.78401000
H	-3.32193000	-1.74275100	-0.27848400
H	-2.45553200	2.36142100	0.69963800
H	-5.69712900	-1.36520300	0.26086900
H	-4.83707200	2.72748400	1.23157100
H	-6.45846100	0.86808300	1.01468600
C	0.84581100	0.09399300	2.50665800
O	1.56655700	0.72108700	3.25623000
C	1.38365100	-0.53688300	1.29648200
O	-0.46632100	-0.08860000	2.70708100
H	2.46397700	-0.55268100	1.25856300
C	-1.01831100	0.50755300	3.91077500
H	-0.94822800	1.59342700	3.81099900
H	-0.39717900	0.20538000	4.75554300
C	-2.44364600	0.02881000	4.04146800
H	-3.04892000	0.33961400	3.18731400
H	-2.88627600	0.45368200	4.94529000
H	-2.48370600	-1.05982300	4.12618400

II

C	0.98145200	0.04436900	-0.29571100
C	0.70084800	-1.30476600	0.38235900
N	-0.72971600	-1.29855700	0.57522700
C	-1.31513000	-0.16144300	0.20443300
C	-0.35788800	0.68592100	-0.41982100
H	-1.16517900	-1.93487500	1.23179300
C	1.31905400	-2.60230400	0.00491300
C	0.85802600	-3.26996600	-1.13172000
C	2.34458900	-3.15907200	0.76394100
C	1.42293900	-4.48017100	-1.50564200
C	2.91297500	-4.36994100	0.38501800
C	2.45417900	-5.02936700	-0.74849000
H	0.06151700	-2.83459500	-1.72757200

H	2.69560600	-2.65317200	1.65732400
H	1.06442700	-4.99389400	-2.39088900
H	3.71158700	-4.79956500	0.97963600
H	2.89833000	-5.97395100	-1.04312400
C	1.99456300	0.19237900	-1.38218400
C	3.27837200	0.66660700	-1.12704800
C	1.63824600	-0.15099100	-2.68718800
C	4.19871400	0.78844000	-2.16217000
C	2.55628000	-0.02899400	-3.71985700
C	3.84038600	0.43904500	-3.45805200
H	3.56081600	0.96133500	-0.12133800
H	0.63544200	-0.51178400	-2.89532900
H	5.19560700	1.16127900	-1.95381900
H	2.27126800	-0.30104500	-4.73035600
H	4.55890000	0.53362800	-4.26492900
C	-0.61139300	1.93070100	-1.07926400
C	-1.85499400	2.20662700	-1.69245200
C	0.40896400	2.90548200	-1.14034200
C	-2.06415000	3.41176000	-2.33303600
C	0.18031300	4.11691000	-1.76357300
C	-1.05214100	4.37247300	-2.36411800
H	-2.63604500	1.45660300	-1.68940900
H	1.36320600	2.70907100	-0.66863300
H	-3.01414100	3.60762000	-2.81730100
H	0.96239900	4.86714400	-1.78592900
H	-1.22377200	5.32049800	-2.86258300
C	-2.70310200	0.10226800	0.58573500
C	-3.65699900	-0.91753400	0.49970600
C	-3.05304400	1.34352100	1.13109000
C	-4.95083700	-0.69379400	0.94817700
C	-4.34478200	1.55323900	1.58966000
C	-5.29348400	0.53860800	1.49645400
H	-3.39345600	-1.87312000	0.05607100
H	-2.31037300	2.12925800	1.21047700
H	-5.69322300	-1.47973500	0.86690000
H	-4.61233900	2.51069600	2.02245300

H	-6.30416000	0.70949700	1.85080800
C	0.84444000	0.57094200	2.26781200
O	1.15269600	1.72336200	2.46052800
C	1.42247600	-0.23529300	1.14187600
O	-0.03476500	-0.11263700	3.00280300
H	2.49200200	-0.38406600	1.23918000
C	-0.67153400	0.60232900	4.09379200
H	-0.97830100	1.58314300	3.72550400
H	0.07465300	0.75171800	4.87703900
C	-1.84055400	-0.23188400	4.55761600
H	-2.58088800	-0.34570600	3.76114800
H	-2.32661200	0.25946500	5.40358500
H	-1.51339700	-1.22197400	4.88292900

II-TS-III

C	0.86782200	-0.10829300	-0.44062400
C	0.51305100	-1.50091300	-0.00961200
N	-0.78803000	-1.46046800	0.33085900
C	-1.39841200	-0.23374100	0.09201200
C	-0.45283500	0.62224900	-0.39820500
H	-1.27942300	-2.24411300	0.74389500
C	1.27818100	-2.75166800	0.00038800
C	2.22890700	-3.02954100	-0.98717100
C	1.03948900	-3.68503100	1.01824300
C	2.91748600	-4.23449400	-0.95688400
C	1.72962600	-4.88597300	1.03776400
C	2.66954900	-5.16218700	0.04848300
H	2.41486100	-2.31958200	-1.78202000
H	0.34425400	-3.45643600	1.82145100
H	3.64858200	-4.45066800	-1.72782600
H	1.54715800	-5.60018400	1.83267400
H	3.21341900	-6.10025100	0.06651000
C	1.70844500	0.11914900	-1.67493600
C	2.98630100	0.66224600	-1.61015700
C	1.15511800	-0.20609200	-2.91405000
C	3.71432300	0.86764800	-2.77799500

C	1.88361700	-0.00392500	-4.07649400
C	3.16746100	0.53180300	-4.00931100
H	3.42120100	0.93490700	-0.65440400
H	0.15067800	-0.61650500	-2.96677100
H	4.71031500	1.29276000	-2.72110800
H	1.44955300	-0.26045000	-5.03663500
H	3.73680500	0.69151600	-4.91836700
C	-0.60597200	2.00878500	-0.81642800
C	-1.76782900	2.43638200	-1.47313700
C	0.42746800	2.92686600	-0.59251200
C	-1.89187000	3.75349300	-1.88561100
C	0.29410900	4.24602000	-0.99783800
C	-0.86451800	4.66183400	-1.64572600
H	-2.56395100	1.72899500	-1.67520100
H	1.33044600	2.60992800	-0.08292000
H	-2.79048600	4.07234600	-2.40218000
H	1.09594400	4.95115500	-0.80897900
H	-0.96589400	5.69262200	-1.96748300
C	-2.80137800	-0.05274400	0.47818000
C	-3.73649100	-1.05869600	0.21734100
C	-3.20077400	1.10523800	1.15256400
C	-5.05450900	-0.90953600	0.62903900
C	-4.51843900	1.24835200	1.56085400
C	-5.44557400	0.24261600	1.30184400
H	-3.44499600	-1.94726800	-0.33675000
H	-2.47444400	1.88391000	1.35294200
H	-5.77744800	-1.68923600	0.41655200
H	-4.82397800	2.14656800	2.08612700
H	-6.47503800	0.35901600	1.62249300
C	1.21332100	0.26890300	2.23182800
O	1.92497900	0.00448700	3.18005900
C	1.60435700	-0.15486300	0.87885800
O	0.05890000	0.93427000	2.31029300
H	2.60374900	-0.57063900	0.84648700
C	-0.38490800	1.32102300	3.63784500
H	-1.00355000	2.20090200	3.46128100

H	0.48942700	1.60188000	4.22535200
C	-1.16600300	0.19850700	4.28491800
H	-2.02850800	-0.07708500	3.67271900
H	-1.53226800	0.52358200	5.26183700
H	-0.53250800	-0.67763700	4.43976000

III

C	0.78679100	0.25400700	-0.18142500
C	0.39254000	-1.21691700	-0.17919900
N	-0.88331000	-1.27995200	-0.44362200
C	-1.51938000	-0.00412200	-0.52109900
C	-0.57741400	0.94141800	-0.36705800
H	-1.41003600	-2.14348300	-0.49393500
C	1.22782900	-2.36603300	0.08265800
C	0.64931500	-3.61762300	0.35526800
C	2.62329900	-2.23578500	0.10049400
C	1.44995400	-4.71176900	0.62405200
C	3.42001100	-3.33713600	0.37050600
C	2.83741700	-4.57264400	0.62972700
H	-0.42880300	-3.73721300	0.39558000
H	3.08259400	-1.28271600	-0.12383300
H	0.99758600	-5.67259100	0.84103800
H	4.49866400	-3.23106300	0.37657800
H	3.46386700	-5.43179900	0.84374400
C	1.66658300	0.53221600	-1.42132000
C	2.97381800	0.99985400	-1.32251400
C	1.12486000	0.29853800	-2.68646200
C	3.72327900	1.23604600	-2.47142200
C	1.87537100	0.52708500	-3.82993800
C	3.17915800	1.00053100	-3.72581800
H	3.43497100	1.18338800	-0.35998300
H	0.10148600	-0.04912000	-2.78558700
H	4.73776800	1.60748000	-2.37703300
H	1.43819700	0.34152700	-4.80490100
H	3.76558200	1.18601800	-4.61881600
C	-0.72904400	2.39549400	-0.51586900

C	-1.33586600	2.90325200	-1.66964800
C	-0.26952800	3.28430700	0.45995000
C	-1.47886100	4.27197300	-1.84233900
C	-0.41715900	4.65399100	0.28460900
C	-1.01929800	5.14998000	-0.86613000
H	-1.68739500	2.21866200	-2.43391700
H	0.18277000	2.91042200	1.37209300
H	-1.94586000	4.65463900	-2.74328400
H	-0.06408600	5.33418200	1.05192900
H	-1.13074800	6.22023100	-1.00214100
C	-2.96974500	0.04424300	-0.71907600
C	-3.60222900	-0.88248000	-1.55408900
C	-3.73386400	1.00985300	-0.05692200
C	-4.98001900	-0.84400800	-1.72387100
C	-5.10873400	1.04555200	-0.23385600
C	-5.73401900	0.11979300	-1.06424400
H	-3.01944800	-1.61697800	-2.10483100
H	-3.24588300	1.72481400	0.59538600
H	-5.46306800	-1.56129500	-2.37810000
H	-5.69673500	1.79549000	0.28363600
H	-6.80985500	0.15048600	-1.19743500
C	0.92008700	0.25658300	2.42313300
O	1.38361600	0.67299900	3.46720300
C	1.41134000	0.66455200	1.11847500
O	-0.10242800	-0.61394800	2.34863800
H	2.16409200	1.43888200	1.13374500
C	-0.68200800	-1.05022500	3.59859900
H	-0.99870500	-0.16634000	4.15629000
H	0.09162600	-1.55773900	4.17900500
C	-1.83858900	-1.95874000	3.25671200
H	-2.58293200	-1.43309200	2.65246900
H	-2.32481400	-2.30072000	4.17285100
H	-1.49406500	-2.84115600	2.71037400

III-TS-IV

C	0.89908700	-0.16221700	-0.18179400
C	0.39866600	-1.57880600	-0.10860800
N	-0.91423900	-1.54064800	-0.21652300
C	-1.44607800	-0.24886100	-0.34083300
C	-0.38885700	0.63822200	-0.37695100
H	-1.50154300	-2.34464000	-0.03181900
C	1.17220000	-2.78191600	0.03400400
C	0.59289200	-4.04504100	-0.17246000
C	2.52106300	-2.69425600	0.40928600
C	1.34522900	-5.19109700	0.00237700
C	3.26624700	-3.84706700	0.59028000
C	2.68182100	-5.09324200	0.38743100
H	-0.43989700	-4.13915800	-0.49356400
H	2.97859400	-1.72537200	0.56296000
H	0.89648300	-6.16347000	-0.16490800
H	4.30600300	-3.77495700	0.88786600
H	3.26965500	-5.99445700	0.52408000
C	2.04120900	0.08613100	-1.13711800
C	3.20516300	0.73682800	-0.74435600
C	1.90373000	-0.34423300	-2.45709000
C	4.22433800	0.95737500	-1.66553100
C	2.92070600	-0.12600000	-3.37429500
C	4.08408600	0.52832300	-2.97875100
H	3.32916900	1.06775900	0.28109300
H	0.99464600	-0.85183000	-2.77019600
H	5.12955900	1.46580700	-1.35219100
H	2.80716100	-0.46521400	-4.39813600
H	4.87979400	0.70118700	-3.69502300
C	-0.42641700	2.05809000	-0.79884800
C	-0.37871800	2.34500700	-2.16453000
C	-0.52152800	3.10719400	0.11804600
C	-0.42454600	3.66149700	-2.60446900
C	-0.56504400	4.42276700	-0.32514400
C	-0.51582300	4.70139200	-1.68653800
H	-0.30340900	1.53554900	-2.88191900

H	-0.56963200	2.89214800	1.18103300
H	-0.38700500	3.87436400	-3.66720500
H	-0.63917800	5.23062200	0.39465500
H	-0.54972200	5.72899700	-2.03182500
C	-2.88276300	-0.06507800	-0.46645400
C	-3.68024800	-1.08524900	-1.00616400
C	-3.48864900	1.12869900	-0.05405500
C	-5.05108400	-0.91707800	-1.12212400
C	-4.85967200	1.29040800	-0.17557700
C	-5.64426400	0.27064500	-0.70551100
H	-3.23009700	-2.00149800	-1.37806400
H	-2.88525300	1.92168300	0.36811500
H	-5.65685700	-1.70942400	-1.54741700
H	-5.32002300	2.21629400	0.15090800
H	-6.71682000	0.40203400	-0.79700900
C	0.45917100	-0.12238600	2.39472700
O	-0.02176000	-1.24306300	2.47770100
C	0.94198200	0.48551500	1.16451400
O	0.60426800	0.70733100	3.42985300
H	1.33207000	1.49199100	1.21153400
C	0.17465000	0.21022400	4.72088600
H	0.72683200	-0.70825100	4.93303900
H	-0.88631900	-0.04149600	4.65223300
C	0.44598300	1.29337000	5.73548100
H	1.51036900	1.53450000	5.77600900
H	0.13374600	0.95217100	6.72514900
H	-0.10895100	2.20313300	5.49603200

IV

C	0.84503700	-0.11301400	-0.08877900
C	0.34406800	-1.50063800	-0.14582300
N	-1.00507600	-1.46285800	-0.20310700
C	-1.54794700	-0.22590900	-0.19084600
C	-0.45217600	0.76094000	-0.11956100
H	-1.56687700	-2.29132800	-0.05123100
C	1.08741800	-2.72546100	-0.15075000

C	0.48739700	-3.94205000	-0.53117500
C	2.43494700	-2.72784900	0.25394000
C	1.20908600	-5.12007300	-0.49240000
C	3.14871800	-3.91275500	0.29077500
C	2.54083300	-5.10965700	-0.07892300
H	-0.53671700	-3.96704900	-0.89138600
H	2.91189700	-1.79973900	0.54209500
H	0.74033300	-6.05003000	-0.79363100
H	4.18470300	-3.90523600	0.61013800
H	3.10506100	-6.03539000	-0.05170700
C	2.07507300	0.26212300	-0.84632000
C	3.14576100	0.91124500	-0.23631500
C	2.14742900	-0.05048800	-2.20459300
C	4.27397200	1.24692400	-0.97610800
C	3.27368000	0.28334600	-2.94220800
C	4.33897300	0.93518000	-2.32852000
H	3.11057000	1.14573400	0.82272000
H	1.31662300	-0.55778900	-2.68605900
H	5.10404400	1.75001700	-0.49230400
H	3.32012100	0.03826100	-3.99765500
H	5.21927800	1.19804600	-2.90488800
C	-0.54110000	2.02498200	-0.90818100
C	-0.81357800	1.94507900	-2.27464400
C	-0.36106900	3.27410400	-0.31898700
C	-0.90249900	3.09795000	-3.04077600
C	-0.44890600	4.42941900	-1.08731900
C	-0.71799700	4.34315600	-2.44767900
H	-0.95626500	0.97431000	-2.74010000
H	-0.16521600	3.35263600	0.74555600
H	-1.11254300	3.02596700	-4.10233000
H	-0.30981700	5.39799000	-0.61965200
H	-0.78570500	5.24498400	-3.04630800
C	-2.96130400	0.00219700	-0.24738300
C	-3.85071200	-1.01948900	-0.63463200
C	-3.48239500	1.25862500	0.11239000
C	-5.21402900	-0.79296000	-0.64581600

C	-4.84905300	1.47522000	0.09931300
C	-5.71761800	0.45388300	-0.27649200
H	-3.47784600	-1.98563800	-0.96129100
H	-2.81108200	2.05578900	0.40523200
H	-5.88973000	-1.58355400	-0.95188600
H	-5.24125600	2.44488500	0.38433700
H	-6.78776400	0.62915300	-0.28844400
C	-0.01881400	0.09263900	2.40741300
O	-0.69643800	-0.90856000	2.51238500
C	0.42183500	0.70154800	1.11944300
O	0.43631100	0.80293500	3.43428800
H	1.01960500	1.59347400	1.24997200
C	0.08478700	0.32729200	4.76025900
H	0.46150000	-0.69263300	4.86349700
H	-1.00472600	0.29564400	4.82932600
C	0.70086800	1.27583100	5.75835300
H	1.78828600	1.29806600	5.65922900
H	0.45602200	0.94741800	6.77103500
H	0.31705000	2.28959400	5.62506500

IV-TS-pro3,4

C	0.45421500	-0.20329700	-0.40643800
C	-0.15418100	-1.47470100	-0.46869000
N	-1.53146900	-1.43792600	-0.43971000
C	-2.13315500	-0.24806400	-0.71110600
C	-1.23028700	0.86431200	-0.67112800
H	-2.07216200	-2.24637800	-0.16168700
C	0.50650200	-2.75867600	-0.59994800
C	-0.15564400	-3.84951500	-1.18832500
C	1.81008100	-2.93547200	-0.10651500
C	0.46922800	-5.08251200	-1.27730300
C	2.42759300	-4.17030000	-0.19961400
C	1.76049900	-5.24556300	-0.78272000
H	-1.14475300	-3.72214700	-1.61681600
H	2.32350400	-2.10249200	0.35834000
H	-0.04465700	-5.91593600	-1.74304200

H	3.43149600	-4.29999000	0.18883000
H	2.24880400	-6.21135300	-0.85426000
C	1.72952300	0.11584100	-1.06203300
C	2.68923200	0.89999200	-0.41298200
C	1.99329100	-0.35471600	-2.35361300
C	3.89287200	1.19418700	-1.03830900
C	3.19512500	-0.05699900	-2.97663400
C	4.14711800	0.71778500	-2.32009500
H	2.50520200	1.25545300	0.59606700
H	1.24653900	-0.94972300	-2.86901400
H	4.63564500	1.79321900	-0.52294800
H	3.38846400	-0.42230700	-3.97929100
H	5.08654700	0.95191000	-2.80906300
C	-1.29978200	1.97663700	-1.61569200
C	-1.69858800	1.74875400	-2.94113900
C	-0.95553700	3.27757300	-1.22556700
C	-1.76289500	2.79523500	-3.84565900
C	-1.02241300	4.32337500	-2.13369900
C	-1.42442400	4.08487700	-3.44405800
H	-1.94993000	0.74254500	-3.25921000
H	-0.66416900	3.48008200	-0.20062200
H	-2.06962600	2.60678300	-4.86854800
H	-0.76543200	5.32826300	-1.81751100
H	-1.47301900	4.90390700	-4.15341500
C	-3.54324800	-0.20009800	-1.01183500
C	-4.21618900	-1.33840100	-1.49142600
C	-4.26781300	0.98488500	-0.79583200
C	-5.57540400	-1.28993400	-1.74538300
C	-5.62694900	1.02411900	-1.05107900
C	-6.28303300	-0.10986500	-1.52450200
H	-3.66733000	-2.24949000	-1.70795100
H	-3.76007000	1.85808500	-0.40557300
H	-6.08579300	-2.16720100	-2.12656400
H	-6.18163300	1.93827000	-0.87267200
H	-7.34856600	-0.07455200	-1.72388800
C	-0.71143700	0.42564200	1.81171800

O	-1.86858700	0.25295400	2.11927100
C	-0.24293800	0.82996100	0.43083100
O	0.32613500	0.28462000	2.62929400
H	0.35890100	1.73251500	0.48575100
C	0.03185900	-0.10552800	3.99651700
H	-0.51339700	-1.05151400	3.96760100
H	-0.62441800	0.65192600	4.43024400
C	1.34689500	-0.21863900	4.72665100
H	1.98808500	-0.97324800	4.26546200
H	1.16417900	-0.51161900	5.76300100
H	1.87710300	0.73619200	4.72983400

pro3,4

C	1.57295200	-0.07139400	-0.28318000
C	0.83229100	-1.23154200	-0.19952300
N	-0.52896800	-1.12031900	-0.15621900
C	-1.27367400	0.00427000	-0.37657400
C	-0.61145400	1.21021500	-0.46623500
H	-1.04516300	-1.97217200	0.03779000
C	1.33840000	-2.61507300	-0.08846700
C	0.82515600	-3.62042300	-0.91260100
C	2.29158200	-2.93469700	0.88244600
C	1.26673100	-4.93041400	-0.77145500
C	2.72557700	-4.24412200	1.02140100
C	2.21574800	-5.24227400	0.19511300
H	0.10675400	-3.37351900	-1.68963100
H	2.68751000	-2.15440600	1.52303500
H	0.87497900	-5.70488900	-1.42150400
H	3.46213100	-4.48922800	1.77858900
H	2.56054900	-6.26462600	0.30509100
C	3.00951800	-0.02878000	-0.54375800
C	3.79029800	1.03400000	-0.05550500
C	3.63419600	-1.01011100	-1.33564400
C	5.15049800	1.08704200	-0.31593500
C	4.98906700	-0.93988300	-1.60714600
C	5.75502500	0.10195300	-1.09019100

H	3.34476000	1.81589600	0.54833900
H	3.04881200	-1.81375900	-1.76304200
H	5.73984800	1.90470200	0.08377900
H	5.45009900	-1.69688600	-2.23181700
H	6.81798800	0.15120000	-1.30017000
C	-1.23201200	2.46023400	-0.89753800
C	-2.28590400	2.46493600	-1.82998500
C	-0.74475800	3.69442700	-0.43200500
C	-2.84203100	3.65676400	-2.25969900
C	-1.31967000	4.88326500	-0.85268800
C	-2.36940000	4.86989500	-1.76547200
H	-2.64958300	1.53182100	-2.24018300
H	0.07339900	3.73415300	0.27760400
H	-3.64334200	3.64131800	-2.99009700
H	-0.94226700	5.82468600	-0.46932400
H	-2.81115800	5.80227000	-2.10003300
C	-2.73186300	-0.22696900	-0.43049000
C	-3.25594300	-1.22526800	-1.25636600
C	-3.58773100	0.51370900	0.38942300
C	-4.62296900	-1.47453300	-1.26702100
C	-4.95036400	0.25805400	0.37750600
C	-5.46956500	-0.73392400	-0.45038200
H	-2.60003200	-1.78385800	-1.91863700
H	-3.17993300	1.28647900	1.03167700
H	-5.02654700	-2.24154500	-1.91868300
H	-5.61096900	0.83185700	1.01803500
H	-6.53649700	-0.92827600	-0.45890200
C	0.91781700	1.57701200	1.46812500
O	1.46588600	2.56224700	1.89687300
C	0.84073400	1.23009300	-0.04518400
O	0.31032100	0.65827600	2.20520200
H	1.35833900	2.03917500	-0.55586100
C	0.30974400	0.86332600	3.64403300
H	-0.16864900	1.82357200	3.84739100
H	1.34816300	0.92165500	3.97658100
C	-0.43284500	-0.29769500	4.25669500

H	-1.46277500	-0.33939900	3.89436700
H	-0.45760000	-0.18357300	5.34269500
H	0.05983800	-1.24479500	4.02424100

II-TS-pro2,3

C	0.83622800	0.53005300	-0.70934600
C	0.41922400	-1.20121200	-0.00669600
N	-0.99535500	-1.04864200	0.05971600
C	-1.46878800	0.17241100	-0.24309300
C	-0.48926700	0.97846600	-0.90240200
H	-1.49492200	-1.63394100	0.71841700
C	0.96138600	-2.40534600	-0.59352700
C	0.15833800	-3.18306400	-1.44640300
C	2.28910000	-2.79987700	-0.35851600
C	0.66793600	-4.32621400	-2.03720200
C	2.79318300	-3.94067500	-0.95837500
C	1.98632700	-4.70629000	-1.79709300
H	-0.85975900	-2.87148800	-1.65124800
H	2.92295800	-2.22744500	0.30815200
H	0.04153300	-4.92023400	-2.69324100
H	3.81715100	-4.24100000	-0.76685800
H	2.38555900	-5.60068500	-2.26284700
C	1.92842700	0.75004900	-1.67461400
C	3.18232700	1.19345500	-1.24609800
C	1.71523400	0.50240600	-3.03435500
C	4.20281200	1.39725100	-2.16541200
C	2.73952000	0.69533500	-3.94801300
C	3.98399400	1.14498200	-3.51523300
H	3.35082000	1.41166000	-0.19565800
H	0.74365100	0.15528400	-3.36960800
H	5.16896200	1.75555100	-1.82743700
H	2.56956400	0.49320700	-4.99986400
H	4.78365300	1.29872200	-4.23178300
C	-0.84926000	2.07669300	-1.80160200
C	-1.98567000	2.00064600	-2.61976900
C	-0.05045600	3.22825900	-1.84930900

C	-2.31300600	3.05029500	-3.46357300
C	-0.38795500	4.27897400	-2.68671200
C	-1.51720800	4.19170400	-3.49668700
H	-2.60396000	1.10976200	-2.60415300
H	0.82717000	3.29731700	-1.21651200
H	-3.18816500	2.97788300	-4.09980600
H	0.22966300	5.16999200	-2.70780100
H	-1.77648600	5.01379300	-4.15508500
C	-2.82621800	0.53952800	0.12629200
C	-3.82641200	-0.43838400	0.23447700
C	-3.12143000	1.86961900	0.45988200
C	-5.09473500	-0.09133100	0.66983400
C	-4.38944300	2.20624000	0.90638800
C	-5.37607500	1.22989500	1.01103600
H	-3.62203400	-1.46446000	-0.05627800
H	-2.35051300	2.62720100	0.38586700
H	-5.86840400	-0.84782000	0.73778500
H	-4.60934100	3.23340100	1.17469500
H	-6.36894000	1.49870100	1.35499200
C	0.74088900	0.61076100	1.86817500
O	1.26185500	1.64540500	2.20690900
C	1.18278200	-0.12469600	0.62129800
O	-0.26081000	0.01305100	2.51116300
H	2.25006100	-0.30878500	0.67164500
C	-0.78348300	0.69359300	3.68374500
H	-0.90296800	1.74992100	3.43650100
H	-0.03906700	0.61071600	4.47830500
C	-2.09171400	0.03163900	4.04152600
H	-2.82040500	0.14101800	3.23396100
H	-2.50439300	0.50139600	4.93724200
H	-1.95096400	-1.03122700	4.25168000

pro2,3

C	0.83622800	0.53005300	-0.70934600
C	0.41922400	-1.20121200	-0.00669600
N	-0.99535500	-1.04864200	0.05971600
C	-1.46878800	0.17241100	-0.24309300
C	-0.48926700	0.97846600	-0.90240200
H	-1.49492200	-1.63394100	0.71841700
C	0.96138600	-2.40534600	-0.59352700
C	0.15833800	-3.18306400	-1.44640300
C	2.28910000	-2.79987700	-0.35851600
C	0.66793600	-4.32621400	-2.03720200
C	2.79318300	-3.94067500	-0.95837500
C	1.98632700	-4.70629000	-1.79709300
H	-0.85975900	-2.87148800	-1.65124800
H	2.92295800	-2.22744500	0.30815200
H	0.04153300	-4.92023400	-2.69324100
H	3.81715100	-4.24100000	-0.76685800
H	2.38555900	-5.60068500	-2.26284700
C	1.92842700	0.75004900	-1.67461400
C	3.18232700	1.19345500	-1.24609800
C	1.71523400	0.50240600	-3.03435500
C	4.20281200	1.39725100	-2.16541200
C	2.73952000	0.69533500	-3.94801300
C	3.98399400	1.14498200	-3.51523300
H	3.35082000	1.41166000	-0.19565800
H	0.74365100	0.15528400	-3.36960800
H	5.16896200	1.75555100	-1.82743700
H	2.56956400	0.49320700	-4.99986400
H	4.78365300	1.29872200	-4.23178300
C	-0.84926000	2.07669300	-1.80160200
C	-1.98567000	2.00064600	-2.61976900
C	-0.05045600	3.22825900	-1.84930900
C	-2.31300600	3.05029500	-3.46357300
C	-0.38795500	4.27897400	-2.68671200
C	-1.51720800	4.19170400	-3.49668700
H	-2.60396000	1.10976200	-2.60415300

H	0.82717000	3.29731700	-1.21651200
H	-3.18816500	2.97788300	-4.09980600
H	0.22966300	5.16999200	-2.70780100
H	-1.77648600	5.01379300	-4.15508500
C	-2.82621800	0.53952800	0.12629200
C	-3.82641200	-0.43838400	0.23447700
C	-3.12143000	1.86961900	0.45988200
C	-5.09473500	-0.09133100	0.66983400
C	-4.38944300	2.20624000	0.90638800
C	-5.37607500	1.22989500	1.01103600
H	-3.62203400	-1.46446000	-0.05627800
H	-2.35051300	2.62720100	0.38586700
H	-5.86840400	-0.84782000	0.73778500
H	-4.60934100	3.23340100	1.17469500
H	-6.36894000	1.49870100	1.35499200
C	0.74088900	0.61076100	1.86817500
O	1.26185500	1.64540500	2.20690900
C	1.18278200	-0.12469600	0.62129800
O	-0.26081000	0.01305100	2.51116300
H	2.25006100	-0.30878500	0.67164500
C	-0.78348300	0.69359300	3.68374500
H	-0.90296800	1.74992100	3.43650100
H	-0.03906700	0.61071600	4.47830500
C	-2.09171400	0.03163900	4.04152600
H	-2.82040500	0.14101800	3.23396100
H	-2.50439300	0.50139600	4.93724200
H	-1.95096400	-1.03122700	4.25168000

7.5 Coordinates for transition states on the energy diagram of 1a-H

I

C	0.86722100	1.73171800	0.35395200
C	0.15947700	0.45658500	0.79853500
N	-1.17742600	0.67467900	0.26294600
C	-1.25859500	1.78945900	-0.41288500
C	0.00459500	2.49160400	-0.36356600
C	-2.48362900	2.21471500	-1.12322200
H	-2.26714000	2.30813000	-2.19173000
H	-3.30629400	1.51261800	-0.98657700
H	-2.78992400	3.20273000	-0.76936700
C	0.77156300	-0.75769900	0.05380100
O	0.26217100	-1.24610900	-0.92246800
O	1.89726400	-1.13140700	0.62723300
C	2.61203800	-2.24943000	0.01080700
H	1.94751500	-3.11497800	0.03155600
H	2.80136800	-1.98397000	-1.03135700
C	2.29139500	2.02414300	0.69082900
H	2.58985200	1.43575200	1.56158400
H	2.36878200	3.07825200	0.97335800
C	0.22867100	3.85634700	-0.94254700
H	-0.29062500	3.93742700	-1.90257700
H	1.29079800	3.98190300	-1.16304000
C	3.24799500	1.73793600	-0.47865900
H	4.26948700	1.98744200	-0.18661000
H	3.22671700	0.68414000	-0.76198300
H	2.99251600	2.33058300	-1.35952600
C	-0.23767500	4.97073700	0.00256600
H	-1.30310200	4.88698500	0.23494700
H	0.31032900	4.93532500	0.94710900
H	-0.07184700	5.94976000	-0.45054000
C	3.87663800	-2.46161800	0.80221800
H	4.51304000	-1.57433400	0.77508500
H	3.65435700	-2.70449300	1.84338900
H	4.43492500	-3.29481300	0.36970100
C	-0.41144000	1.16893200	3.25746500

O	-0.37684200	0.94168700	4.45010900
C	0.16150100	0.24462700	2.28658100
O	-0.95911900	2.26365100	2.70448400
H	0.66640100	-0.62142000	2.69331200
C	-1.50358600	3.25063100	3.62231200
H	-2.21155100	3.81198300	3.01201600
H	-2.04212200	2.72359400	4.41097000
C	-0.41409300	4.13716800	4.18384800
H	0.13591600	4.63777200	3.38297900
H	0.28401700	3.56244800	4.79468600
H	-0.86329600	4.90569000	4.81761900
H	-1.90009100	-0.03446200	0.29120400

I-TS-II

C	1.51454300	0.70114800	-0.56772700
C	0.71401800	-0.36473900	0.11973600
N	-0.62639500	-0.10293800	-0.31453100
C	-0.70546900	1.02154500	-1.00202800
C	0.59763600	1.56975400	-1.17439300
C	-1.99310100	1.57988100	-1.47519200
H	-2.19161600	2.51980300	-0.95161700
H	-1.94231100	1.80240900	-2.54351800
H	-2.82290100	0.89617200	-1.29383100
C	1.03181600	-1.85194900	0.10191900
O	0.16232600	-2.67069600	-0.06935100
O	2.30785400	-2.08559900	0.32789600
C	2.72239000	-3.48829900	0.37894000
H	2.16236000	-3.96611400	1.18476800
H	2.43417800	-3.95091200	-0.56667000
C	2.99918500	0.70403500	-0.78391200
H	3.50079800	0.15579000	0.01281400
H	3.36142500	1.73455800	-0.74595700
C	0.91968500	2.85335200	-1.86976600
H	0.12847300	3.09250500	-2.58542400
H	1.83444500	2.72717600	-2.45519800
C	3.35024300	0.06640700	-2.13523800

H	4.43107200	0.09331700	-2.28581600
H	3.03115100	-0.97772400	-2.17272200
H	2.88100600	0.59575900	-2.96781700
C	1.09821900	4.01661400	-0.88272500
H	0.18394200	4.19926800	-0.31411500
H	1.89998900	3.81022700	-0.16889500
H	1.35146500	4.93232800	-1.41992800
C	4.21138900	-3.50544000	0.61005000
H	4.74279400	-3.00628100	-0.20336400
H	4.47030200	-3.02007000	1.55352700
H	4.55549000	-4.54103200	0.65524500
C	0.40507200	1.36676300	2.16914200
O	0.94305900	1.87216400	3.13092900
C	1.14149600	0.39622200	1.34554300
O	-0.85430900	1.61255500	1.78662300
H	2.10565000	0.12361900	1.75353800
C	-1.62047000	2.52073600	2.63167600
H	-2.65422300	2.21565100	2.46912900
H	-1.34469000	2.33222300	3.66915700
C	-1.38509400	3.96268400	2.24364400
H	-1.67033200	4.14505900	1.20388500
H	-0.33985000	4.24318200	2.38608500
H	-1.99722300	4.61083100	2.87549900
H	-1.40377000	-0.69244300	-0.04161700

II

C	1.50946300	0.73606200	-0.55224500
C	0.70659700	-0.49892800	-0.35575800
N	-0.53399200	-0.23921100	-0.73812500
C	-0.68085200	1.04051200	-1.28112000
C	0.52102200	1.66760400	-1.20282100
C	-2.01661600	1.49304200	-1.74840000
H	-2.70910200	1.57802900	-0.90515800
H	-1.94487600	2.46994100	-2.22434900
H	-2.44380000	0.79396800	-2.47282400
C	1.02287200	-1.88289300	0.10443100

O	0.14980600	-2.72000800	0.13158400
O	2.28854400	-2.03986000	0.42928100
C	2.68689200	-3.37181800	0.89001700
H	2.07062900	-3.61485200	1.75758200
H	2.45575200	-4.07694400	0.08958100
C	2.89488200	0.62989800	-1.20197300
H	3.52020700	-0.01710200	-0.58414100
H	3.34374800	1.62682500	-1.17702600
C	0.87718300	3.05073000	-1.62949400
H	0.01392900	3.51645900	-2.10994200
H	1.65865600	2.98744800	-2.39635400
C	2.83210100	0.09578900	-2.62885000
H	3.83397700	0.05282700	-3.05927200
H	2.41937300	-0.91706400	-2.65794500
H	2.22007400	0.72821400	-3.27714200
C	1.37325100	3.93629700	-0.47872100
H	0.60043900	4.06744400	0.28045000
H	2.25919900	3.51547300	0.00514600
H	1.64521100	4.92239400	-0.85848100
C	4.15726800	-3.31169800	1.21314400
H	4.74516200	-3.04800300	0.33127000
H	4.35885300	-2.58731200	2.00530200
H	4.48718200	-4.29346500	1.55978000
C	0.65528100	1.47395600	1.93375100
O	1.07002700	1.71982000	3.04736700
C	1.55247500	0.82808200	0.96209500
O	-0.58338300	1.72727400	1.50788800
H	2.41899200	0.36913700	1.42519200
C	-1.49132200	2.33664000	2.47335800
H	-2.47904800	2.03510900	2.12435000
H	-1.29870300	1.89086400	3.44952600
C	-1.33352000	3.83988400	2.50005200
H	-1.52170000	4.27414500	1.51499000
H	-0.33511000	4.12465900	2.83742700
H	-2.05786100	4.26493900	3.19922700
H	-1.27744900	-0.92480400	-0.61425900

II-TS-III

C	1.50946300	0.73606200	-0.55224500
C	0.70659700	-0.49892800	-0.35575800
N	-0.53399200	-0.23921100	-0.73812500
C	-0.68085200	1.04051200	-1.28112000
C	0.52102200	1.66760400	-1.20282100
C	-2.01661600	1.49304200	-1.74840000
H	-2.70910200	1.57802900	-0.90515800
H	-1.94487600	2.46994100	-2.22434900
H	-2.44380000	0.79396800	-2.47282400
C	1.02287200	-1.88289300	0.10443100
O	0.14980600	-2.72000800	0.13158400
O	2.28854400	-2.03986000	0.42928100
C	2.68689200	-3.37181800	0.89001700
H	2.07062900	-3.61485200	1.75758200
H	2.45575200	-4.07694400	0.08958100
C	2.89488200	0.62989800	-1.20197300
H	3.52020700	-0.01710200	-0.58414100
H	3.34374800	1.62682500	-1.17702600
C	0.87718300	3.05073000	-1.62949400
H	0.01392900	3.51645900	-2.10994200
H	1.65865600	2.98744800	-2.39635400
C	2.83210100	0.09578900	-2.62885000
H	3.83397700	0.05282700	-3.05927200
H	2.41937300	-0.91706400	-2.65794500
H	2.22007400	0.72821400	-3.27714200
C	1.37325100	3.93629700	-0.47872100
H	0.60043900	4.06744400	0.28045000
H	2.25919900	3.51547300	0.00514600
H	1.64521100	4.92239400	-0.85848100
C	4.15726800	-3.31169800	1.21314400
H	4.74516200	-3.04800300	0.33127000
H	4.35885300	-2.58731200	2.00530200
H	4.48718200	-4.29346500	1.55978000
C	0.65528100	1.47395600	1.93375100

O	1.07002700	1.71982000	3.04736700
C	1.55247500	0.82808200	0.96209500
O	-0.58338300	1.72727400	1.50788800
H	2.41899200	0.36913700	1.42519200
C	-1.49132200	2.33664000	2.47335800
H	-2.47904800	2.03510900	2.12435000
H	-1.29870300	1.89086400	3.44952600
C	-1.33352000	3.83988400	2.50005200
H	-1.52170000	4.27414500	1.51499000
H	-0.33511000	4.12465900	2.83742700
H	-2.05786100	4.26493900	3.19922700
H	-1.27744900	-0.92480400	-0.61425900

III

C	0.45701500	1.95609700	-0.41568700
C	-0.40499500	0.79310600	-0.05043000
N	-1.57220500	0.94552800	-0.57784500
C	-1.71996200	2.19759700	-1.24479700
C	-0.54406700	2.84502400	-1.15168400
C	-3.02448200	2.54153300	-1.86987900
H	-2.95819900	3.51013200	-2.36468700
H	-3.31740100	1.79950500	-2.61879900
H	-3.82016500	2.60263700	-1.12103700
C	-0.12517300	-0.44931100	0.73420000
O	-0.99699500	-1.26981400	0.90472200
O	1.11174600	-0.48150100	1.17039400
C	1.49007500	-1.62344800	2.00538800
H	0.78415100	-1.66423200	2.83712800
H	1.36842700	-2.52387600	1.40017500
C	1.59361900	1.46693400	-1.38422800
H	2.26829500	0.84478100	-0.79208400
H	2.15068500	2.36511500	-1.66423800
C	-0.21346100	4.20826300	-1.66426100
H	-0.81567600	4.41362600	-2.55346000
H	0.83126600	4.22897900	-1.99051100
C	1.13071400	0.72552000	-2.63215600

H	0.46267600	1.33184100	-3.24770600
H	1.99818300	0.47056000	-3.24347100
H	0.62154400	-0.21342600	-2.39553600
C	-0.44936500	5.30926300	-0.61936000
H	0.17676800	5.16894700	0.26507100
H	-0.21097800	6.28600300	-1.04425900
H	-1.49277200	5.32402300	-0.29560500
C	2.91148800	-1.39898500	2.45104900
H	3.58953400	-1.34588100	1.59659700
H	3.00106100	-0.47940000	3.03330600
H	3.22443600	-2.23415300	3.08147000
C	0.47680500	2.72587800	2.07860500
O	0.96270500	3.35142600	2.99833800
C	1.08697700	2.63211100	0.76644700
O	-0.68563600	2.03960300	2.15637000
H	2.03081900	3.14645500	0.63285800
C	-1.41258600	2.10928300	3.41354900
H	-2.01631600	1.20101200	3.42074300
H	-0.68658800	2.07154900	4.22652600
C	-2.26143400	3.35923700	3.47406300
H	-2.96063500	3.39888500	2.63482800
H	-1.63852000	4.25552800	3.46539800
H	-2.84165700	3.36012600	4.39999000
H	-2.30018900	0.23932900	-0.46393500

III-TS-IV

C	-1.24435700	2.42544100	-0.04653600
C	-2.69913100	2.13780500	0.00811900
N	-3.35567000	3.27460400	-0.04679700
C	-2.54104500	4.38451400	-0.12641600
C	-1.22916300	3.94089200	-0.18650700
C	-3.10946800	5.75669000	-0.18429800
H	-3.24348500	6.16965900	0.82095000
H	-2.44085600	6.42326700	-0.73059600
H	-4.07959000	5.76343700	-0.68695700
C	-3.47720900	0.89027700	0.18249400

O	-4.68880700	0.92214300	0.21721700
O	-2.70333900	-0.16747000	0.30259600
C	-3.37314800	-1.44950200	0.51282800
H	-3.96963100	-1.36145500	1.42311000
H	-4.04848700	-1.60924900	-0.32989000
C	-0.39416900	1.55679200	-0.97729900
H	-0.42241800	0.53452300	-0.59468900
H	0.64258000	1.89437000	-0.89849900
C	-0.02880200	4.79174800	-0.48018700
H	-0.23275000	5.33016700	-1.41335200
H	0.82546900	4.14447200	-0.69375900
C	-0.86553900	1.61271600	-2.42619700
H	-0.85432400	2.63506800	-2.81475000
H	-0.21283300	1.01154300	-3.06126700
H	-1.88035400	1.21850900	-2.53706200
C	0.33498000	5.79135700	0.62209400
H	0.59056100	5.28149100	1.55391600
H	1.19560100	6.38882700	0.31643400
H	-0.48815500	6.47760800	0.83327100
C	-2.30169000	-2.50405000	0.61500500
H	-1.71388500	-2.55918600	-0.30382000
H	-1.63110100	-2.30503100	1.45367200
H	-2.77069400	-3.47702300	0.77690300
C	-1.30474600	2.66299200	2.63517400
O	-0.65324100	2.78313300	3.65130900
C	-0.66558400	2.67547300	1.31956900
O	-2.63534700	2.49483400	2.60565100
H	0.40470100	2.83719400	1.35361900
C	-3.30470800	2.41913100	3.89516100
H	-2.93997400	1.52669600	4.40812200
H	-3.01002100	3.29137900	4.48114200
C	-4.78920200	2.36912300	3.63017800
H	-5.05621200	1.51411400	3.00480900
H	-5.13342500	3.28376700	3.13975800
H	-5.32428800	2.27847800	4.57797400
H	-4.37236800	3.28723600	0.04007700

IV

C	0.44595100	2.11991700	-1.04837800
C	0.94049700	2.12284500	0.33420500
N	1.17030500	0.83408600	0.72757200
C	0.87207900	-0.06771900	-0.19246300
C	0.41281700	0.61858400	-1.42000000
C	1.04064100	-1.51547000	0.02341200
H	0.04912700	-1.97936700	0.05906200
H	1.58658400	-1.96393300	-0.81080600
H	1.56420200	-1.73168900	0.95547100
C	1.20676600	3.16891000	1.32650700
O	1.63440300	2.87408700	2.42738500
O	0.92664400	4.38264200	0.88928300
C	1.16184400	5.48067200	1.82121900
H	0.54794700	5.30068500	2.70606200
H	2.21211200	5.44494300	2.11748000
C	0.83135100	3.23186100	-2.00124700
H	0.39533100	4.16346200	-1.63266200
H	0.36392400	3.03231600	-2.96918800
C	0.74346900	0.03660400	-2.78223900
H	0.71131400	0.85097400	-3.51104600
H	1.77910500	-0.31887500	-2.77938900
C	2.34225600	3.37418200	-2.16186100
H	2.57705000	4.16719800	-2.87407400
H	2.82401600	3.63248900	-1.21478000
H	2.79459300	2.44950100	-2.53160800
C	-0.20913800	-1.07546800	-3.21802200
H	-1.24052200	-0.71603100	-3.26241500
H	0.06067500	-1.43757300	-4.21166700
H	-0.18514600	-1.93101400	-2.53896000
C	0.79921300	6.75831200	1.10851800
H	1.42013900	6.90456200	0.22199200
H	-0.25091000	6.75762900	0.80844400
H	0.96133100	7.60374300	1.78074100
C	-1.82972600	1.08808400	-0.12299100

O	-1.60838400	0.26948100	0.74874600
C	-0.88186600	1.38744100	-1.24147500
O	-2.91759500	1.83016700	-0.21797400
H	-1.37279700	1.66615100	-2.16832400
C	-3.92395300	1.68831900	0.82704500
H	-4.84309900	2.01773700	0.34483700
H	-4.00051000	0.63111300	1.08356200
C	-3.57070900	2.54121700	2.02484300
H	-3.45945000	3.58907400	1.73650800
H	-2.64882800	2.19545600	2.49759300
H	-4.37222600	2.47537700	2.76460900
H	1.44637900	0.63358200	1.68882000

IV-TS-pro3,4

C	-0.43270800	1.56206400	0.12347700
C	0.09655000	1.44308700	1.40621700
N	0.24341300	0.14517400	1.88460700
C	0.20308100	-0.82367600	0.98109300
C	-0.23241700	-0.36349100	-0.32477000
C	0.55247700	-2.22113000	1.31544400
H	-0.37128700	-2.80554100	1.39274900
H	1.15927500	-2.66241300	0.52142400
H	1.09367100	-2.28799900	2.26061000
C	0.62455300	2.46027300	2.34788100
O	1.03744800	2.11561100	3.43867300
O	0.55393200	3.69723700	1.90051300
C	1.03566200	4.74414100	2.79632900
H	0.43208900	4.70469900	3.70517900
H	2.06890100	4.50789300	3.05806000
C	-0.17302900	2.72702000	-0.79937400
H	-0.57117200	3.62793300	-0.32285800
H	-0.75751500	2.57973100	-1.71056500
C	0.31417500	-0.90839500	-1.60934600
H	0.31445100	-0.10229200	-2.34966800
H	1.35554600	-1.21518400	-1.48214700
C	1.30138200	2.92794100	-1.15057500

H	1.41382600	3.77326100	-1.83234200
H	1.89904400	3.13654800	-0.26141500
H	1.71664800	2.04395200	-1.64205300
C	-0.53687200	-2.07666500	-2.13300000
H	-1.57175000	-1.76851500	-2.29987100
H	-0.13157800	-2.43090800	-3.08273700
H	-0.54502200	-2.91691600	-1.43543100
C	0.90346000	6.05451200	2.06309800
H	1.50618200	6.05887300	1.15223200
H	-0.13726900	6.25542200	1.79972100
H	1.25414700	6.86413200	2.70687200
C	-2.53166000	0.13116300	0.68948500
O	-2.54359400	-0.90329500	1.32116200
C	-1.42705400	0.49004000	-0.28715300
O	-3.44218800	1.08555600	0.74149700
H	-1.83940800	0.71100300	-1.26997700
C	-4.55328600	0.90760500	1.66868900
H	-5.34269400	1.52830000	1.24737700
H	-4.85429900	-0.14019200	1.64070600
C	-4.15954000	1.35090700	3.05965200
H	-3.82254500	2.39021200	3.05647000
H	-3.37058800	0.71529100	3.46769200
H	-5.02535900	1.27704900	3.72215500
H	0.49471500	0.01339800	2.86195100

II-TS-pro2,3

C	-1.92009600	2.26644700	-0.08701800
C	-3.21672100	0.84631900	0.30526600
N	-4.29626900	1.33985000	-0.44563700
C	-4.10655200	2.50178700	-1.05445200
C	-2.72680400	2.92862900	-1.01455400
C	-5.22254100	3.19094100	-1.73786500
H	-5.58172600	3.99184300	-1.08161900
H	-4.89745900	3.64712500	-2.67320700
H	-6.05386000	2.51247400	-1.93800800
C	-2.99963900	-0.61387200	0.23054000

O	-3.78798400	-1.32465800	-0.35734300
O	-1.90779900	-0.99991700	0.86586600
C	-1.61684800	-2.43019300	0.85416200
H	-2.45113900	-2.94031300	1.33948800
H	-1.57521500	-2.75187800	-0.18844400
C	-0.42347300	2.12930100	-0.23179800
H	-0.00575900	1.74663600	0.70128100
H	-0.00536200	3.13460000	-0.35775300
C	-2.21739500	3.95747900	-1.97919300
H	-2.71971100	3.85744800	-2.94533500
H	-1.15666200	3.78636700	-2.16627100
C	-0.00019200	1.23061000	-1.39580500
H	1.08839400	1.19370000	-1.46938100
H	-0.36230800	0.21017400	-1.24869600
H	-0.39009300	1.59128300	-2.35065300
C	-2.41555700	5.38107900	-1.43065200
H	-3.46837600	5.59764300	-1.23787900
H	-1.87270700	5.51588400	-0.49254000
H	-2.04412200	6.11116500	-2.15223600
C	-0.31055800	-2.62783200	1.57952900
H	0.50216200	-2.09596100	1.07977800
H	-0.37749500	-2.28087500	2.61288100
H	-0.06359200	-3.69168000	1.59255800
C	-3.43366300	2.77824800	1.96474100
O	-4.09984000	3.63623600	1.42607700
C	-2.56273200	1.78826500	1.20909800
O	-3.36530800	2.55316900	3.26244100
H	-1.84945100	1.29396700	1.86097400
C	-4.18596500	3.38537200	4.13763600
H	-3.66519600	3.34016400	5.09291600
H	-4.16191900	4.40532500	3.75220500
C	-5.59326200	2.84096000	4.22785200
H	-5.58986900	1.80652300	4.57842400
H	-6.10054600	2.89373200	3.26228900
H	-6.16511800	3.43956500	4.94110200
H	-5.11723600	0.75840400	-0.58216100

pro2,3

C	2.58793700	0.66904300	-0.33372400
C	0.26641500	-0.21987000	-0.27472000
N	-0.06846900	0.81619600	-1.06248400
C	0.76550700	1.76987000	-1.48533300
C	2.15537700	1.67343000	-1.15795900
C	0.18873400	2.84252400	-2.34433800
H	0.49548300	3.82832000	-1.98868200
H	0.54848100	2.74260900	-3.37350900
H	-0.90165200	2.80315800	-2.35869900
C	-0.84703700	-1.13672300	0.01667300
O	-1.95084100	-0.95353700	-0.46314000
O	-0.48779100	-2.11222100	0.82852300
C	-1.51986500	-3.07500700	1.20138700
H	-2.33809800	-2.51786800	1.66209600
H	-1.88900200	-3.53470000	0.28247500
C	4.03131900	0.41195700	-0.02837600
H	4.11734900	0.02765600	0.99110200
H	4.60686000	1.33690700	-0.07495500
C	3.10308700	2.70051900	-1.73720500
H	2.73214000	3.04544900	-2.70458800
H	4.06357100	2.22727300	-1.94699100
C	4.61881600	-0.61786600	-1.01093700
H	5.65866000	-0.82180600	-0.75072800
H	4.07598500	-1.56603600	-0.97256500
H	4.58845800	-0.25156100	-2.03986200
C	3.30650600	3.89861800	-0.80273200
H	2.36338300	4.40962900	-0.58932300
H	3.73301600	3.58580900	0.15374200
H	3.98499900	4.62493900	-1.25406000
C	-0.88675300	-4.06952400	2.14024800
H	-0.06348700	-4.59904700	1.65623800
H	-0.50857800	-3.57770600	3.03909700
H	-1.63520200	-4.80546900	2.44225100
C	1.55298900	0.05304400	1.84797400

O	2.39075800	-0.30642000	2.63110800
C	1.61465200	-0.27814400	0.32808200
O	0.48957600	0.79656900	2.12337000
H	2.00546000	-1.30123800	0.29491400
C	0.30192800	1.17743500	3.51888400
H	0.28508300	0.26147000	4.11244100
H	1.16903800	1.76780300	3.82134900
C	-0.98934000	1.95081400	3.60254300
H	-1.83552000	1.34116100	3.27755000
H	-0.94888700	2.85368700	2.98897900
H	-1.16421000	2.25154900	4.63790400
H	-1.05245500	0.81841100	-1.34702000

pro3,4

C	-1.07665600	1.22758100	0.04773600
C	0.21945700	0.84989700	0.13657000
N	1.18942100	1.51232200	-0.59932800
C	1.00562700	2.59165500	-1.37683600
C	-0.28360300	3.08253500	-1.52035800
C	2.22294800	3.16672100	-2.03086100
H	2.14636300	4.24966800	-2.12525300
H	3.12330700	2.93866500	-1.45692400
H	2.34670600	2.75117100	-3.03518800
C	0.85968800	-0.23159900	0.95235900
O	2.05587900	-0.42731700	0.85418200
O	0.03774100	-0.89081400	1.73626400
C	0.62008000	-1.95874700	2.54650300
H	1.08234000	-2.67491300	1.86487200
H	1.40170300	-1.51421200	3.16526100
C	-2.21790300	0.64842100	0.82883100
H	-2.08528000	-0.42456200	0.95515800
H	-3.13985500	0.80181800	0.25984000
C	-0.59541900	4.29172000	-2.34790900
H	-1.57943300	4.66972100	-2.06104100
H	0.12507000	5.08414500	-2.12135300
C	-2.35586200	1.31996400	2.20444100

H	-3.23071000	0.92258200	2.72218500
H	-1.47703600	1.12663600	2.82220500
H	-2.48423200	2.40218400	2.11514300
C	-0.58148300	4.01579600	-3.86244800
H	-1.30601100	3.24682700	-4.13742700
H	-0.83764100	4.92963100	-4.40128300
H	0.40074700	3.68700600	-4.20674800
C	-0.49785500	-2.56081400	3.35865300
H	-0.95367300	-1.81630900	4.01508600
H	-1.27013200	-2.98494200	2.71320900
H	-0.09716500	-3.36357500	3.98150500
C	-2.27250100	1.73473600	-2.05535700
O	-1.94273500	0.73315400	-2.63877300
C	-1.42624900	2.33939200	-0.89928600
O	-3.34077400	2.47156300	-2.30529000
H	-2.08524900	3.05447100	-0.38935600
C	-4.19632000	2.02782600	-3.40203400
H	-3.58062900	1.97021200	-4.30228500
H	-4.54630000	1.02199800	-3.16214700
C	-5.32057100	3.02406500	-3.52646100
H	-4.93977100	4.02213800	-3.75387700
H	-5.90670100	3.07289100	-2.60640800
H	-5.98414700	2.71866500	-4.33854800
H	2.12459800	1.11729900	-0.46724200
